# TDP-43 promotes allergen-induced inflammation and influenza A virus replication in airway epithelium

**DOI:** 10.1038/s41467-026-75901-0

**Published:** 2026-07-23

**Authors:** Yanmei Yuan, Wenzhu Tang, Xixi Feng, Chunlei Zhang, Yingying Liao, Meiyan Tang, Jian He, Shengpeng Wan, Guihong Pan, Xiaojiang Lai, Qing Xue, Jun Wang

**Affiliations:** 1 Precision Research Center for Refractory Diseases, Shanghai Jiao Tong University Pioneer Research Institute for Molecular and Cell Therapies, Shanghai General Hospital, Shanghai Jiao Tong University School of Medicine, Shanghai, China; 2 State Key Laboratory of Innovative Immunotherapy, School of Pharmaceutical Sciences, Shanghai Jiao Tong University, Shanghai, China; 3 Department of Pediatric Surgery, Guangdong Provincial Key Laboratory of Research in Structural Birth Defect Disease, Guangdong Provincial Clinical Research Center for Child Health, Guangzhou Institute of Pediatrics, Guangzhou Women and Children’s Medical Center, Guangzhou Medical University, Guangzhou, China; 4 Guangzhou Institutes of Biomedicine and Health, Chinese Academy of Sciences, Guangzhou, China; 5 Guangzhou Medical University-Guangzhou Institute of Biomedicine and Health (GMU-GIBH) Joint School of Life Sciences, Guangzhou Medical University, Guangzhou, China; 6 Ningde Clinical Medical College of Fujian Medical University, Fuzhou, China; 7 Department of Respiratory and Critical Care Medicine, Ningde Municipal Hospital, Ningde, China

**Keywords:** Respiratory tract diseases, Mucosal immunology, Asthma, Asthma, Viral infection

## Abstract

Current targeted therapeutics for asthma focus on the T2 immune response, providing symptomatic management without addressing the issues of recurrence. Identifying key pathophysiological determinants in airway epithelium enables novel therapeutics to prevent asthma recurrence. Here we demonstrate that TDP-43, as the master regulator of RNA metabolism, is induced in airway epithelial cells by host allergen exposure and promotes asthmatic airway responses through dampening nonsense-mediated decay (NMD) by modulating UPF1 expression, resulting in accumulation of pathogenic and NMD-sensitive transcripts including *CHRM1* encoding receptor that transduces the signaling of vagally-derived neurotransmitter acetylcholine. TDP-43 also facilitates airway influenza A virus replication. Intranasal application of lipid nanoparticles carrying antisense oligonucleotides targeting TDP-43 effectively reduce asthma-like pathologies in mice. Our findings indicate that targeting airway TDP-43 is therapeutically possible for asthma and influenza A virus infection-complicated asthma exacerbations.

## Abstract (web summary)

Different co-factors are involved with the promotion of asthma and the replication of influenza virus in the respiratory tract. Here the authors propose that TAR DNA-binding protein 43 (TDP-43) is involved with the promotion of allergic airway responses involving UPF1-mediated nonsense-mediated decay (NMD) activity leading to accumulation of NMD-sensitive transcripts and the replication of influenza virus in mouse models.

## Introduction

Asthma is a major chronic non-communicable lung disease featured as airway hyperresponsiveness (AHR), reversible airflow obstruction, high mucus secretion, chronic inflammation, and airway remodeling^1^. Asthma affects approximately 334 million people globally, with a surging incidence contributing to over 400,000 deaths annually. The cornerstone management for asthma involves the using of glucocorticoids and bronchodilators^1^. The current targeted therapeutics for asthma predominantly target type II (T2) immune response, including anti-IL-4Rα (Dupilumab, Sanofi)^2^, anti-IgE (Omalizumab, Novartis)^3^, and anti-IL-5/5R (Depemokimab, GSK)^4^. However, this strategy is ineffective for non‑T2 asthmatics, addressing neither recurrence nor virus‑induced acute exacerbations, such as asthma exacerbations complicated by influenza A virus infection (IAV‑AEs)^5^.

Asthmatic response can be triggered by a wide array of allergens that share minimal structural or biological commonality, including those with protease activity, such as house dust mites (HDM) and cockroach antigen (CA), and those without^6^. It is widely acknowledged that allergen-induced T2 inflammation is the primary driver of airway pathology. At early phase of murine asthmatic modeling, animals are sensitized by intraperitoneal injection of allergens emulsified with adjuvant, resulting in the priming of host pro-Th2 immunity. During sensitization, airway epithelium abnormalities begin to develop, as circulating T2 cytokines from pro-Th2 can act on airway epithelial cells (AECs) to initiate pathological changes facilitating allergen penetration during the challenge phase^7,8^, resembling the intrinsic barrier defects in populations susceptible to allergen-induced asthma^9,10^. Abnormal airway epithelium triggers the release of alarmins such as IL-33, IL-25, and thymic stromal lymphopoietin (TSLP). These alarmins are essential to fully activate T2 effector cells, which secrete more T2 cytokines that boost airway eosinophilia and generation of bronchoconstrictor mediators, ultimately driving bronchoconstriction and AHR^11^. These T2 cytokines further impair the airway epithelium, creating a vicious cycle perpetuating allergen-induced airway disturbance upon repeated challenge, wherein the vagal neurotransmitter acetylcholine (Ach) promotes bronchoconstriction and mucus hypersecretion via muscarinic receptor activation^12,13^. Elucidating the molecular determinants in AECs that mediate epithelial injuries across disease course is therefore critically urgent to develop novel therapeutics for asthma.

Airway injuries induced by host allergen exposure are increasingly recognized to trigger cellular stress in AECs: Chen et al. demonstrated that host allergen exposure activates cellular stress in AECs, leading to stress granule formation and subsequent IL-33 maturation and secretion^14^; endoplasmic reticulum (ER) stress is closely associated with asthmatic airway response, as evidenced in both allergen-induced asthmatic murine models and airway epithelial brushing specimens from asthmatic patients^15,16^; preclinical studies also showed that suppression of ER stress significantly diminished asthmatic airway response^17^. In eukaryotic cells, aberrantly processed mRNAs are inspected and eliminated by RNA quality control (RQC) machineries, such as nonsense-mediated mRNA decay (NMD) triggered by premature termination codons (PTCs), thereby maintaining transcriptome integrity^18^. Deteriorated NMD activity contributes to the pathogenesis of ageing, genetic diseases and cancers^18^. While cellular stress frequently compromises NMD activity and causes the accumulation of stress-related and NMD-sensitive transcripts^19–21^, the mechanisms through which cellular stress impairs NMD remain elusive. We hypothesize that airway abnormalities and the accompanying cellular stress following host allergen exposure may disrupt NMD in AECs, playing a role in the onset of asthmatic reactions.

TAR DNA-Binding Protein-43 (TDP-43, encoded by the *TARDBP* gene) is a master regulator in RNA metabolism and performs a crucial role in RQC^22^. In motor neurons (MNs), TDP-43 represses cryptic exon splicing, ensuring correct processing of pre-mRNAs, such as *STMN2* and *UNC13A*, which are essential for neural development^23,24^. The nuclear clearance of TDP-43 in MNs is a defining characteristic of neurodegeneration in TDP-43 proteinopathies including amyotrophic lateral sclerosis and frontotemporal dementia^25^. While proper expression level and correct nuclear distribution of TDP-43 is required for its functions in RNA splicing, cellular stress frequently induces dysregulated TDP-43 expression, nuclear condensation and cytoplasmic aggregation, directly effecting RQC^26,27^. Ectopic overexpression of TDP-43 in murine neurons dampened NMD, leading to cytotoxicity and survival issues^28,29^.

Here, we show that host allergen exposure-induced TDP-43 expression in AECs promotes asthmatic airway inflammation by dampening UPF1-mediated NMD activity, leading to accumulation of NMD-sensitive transcripts encoding pathogenic molecules including cholinergic receptor muscarinic 1 (CHRM1). Mice with specific ablation of TDP-43 in airway epithelium are resistant to allergen-induced asthma, IAV infection and IAV-AEs. We also prove the draggability of TDP-43 by intranasal administering of lipid nanoparticles encapsulating antisense oligonucleotides (LNP-ASO) targeting TDP-43 in allergen-induced asthmatic mice. Our findings propose that TDP-43 can serve as a potential therapeutic target for asthma.

## Results

### NMD degeneration coexists with induced TDP-43 expression in asthmatic AECs

**Fig. 1:**
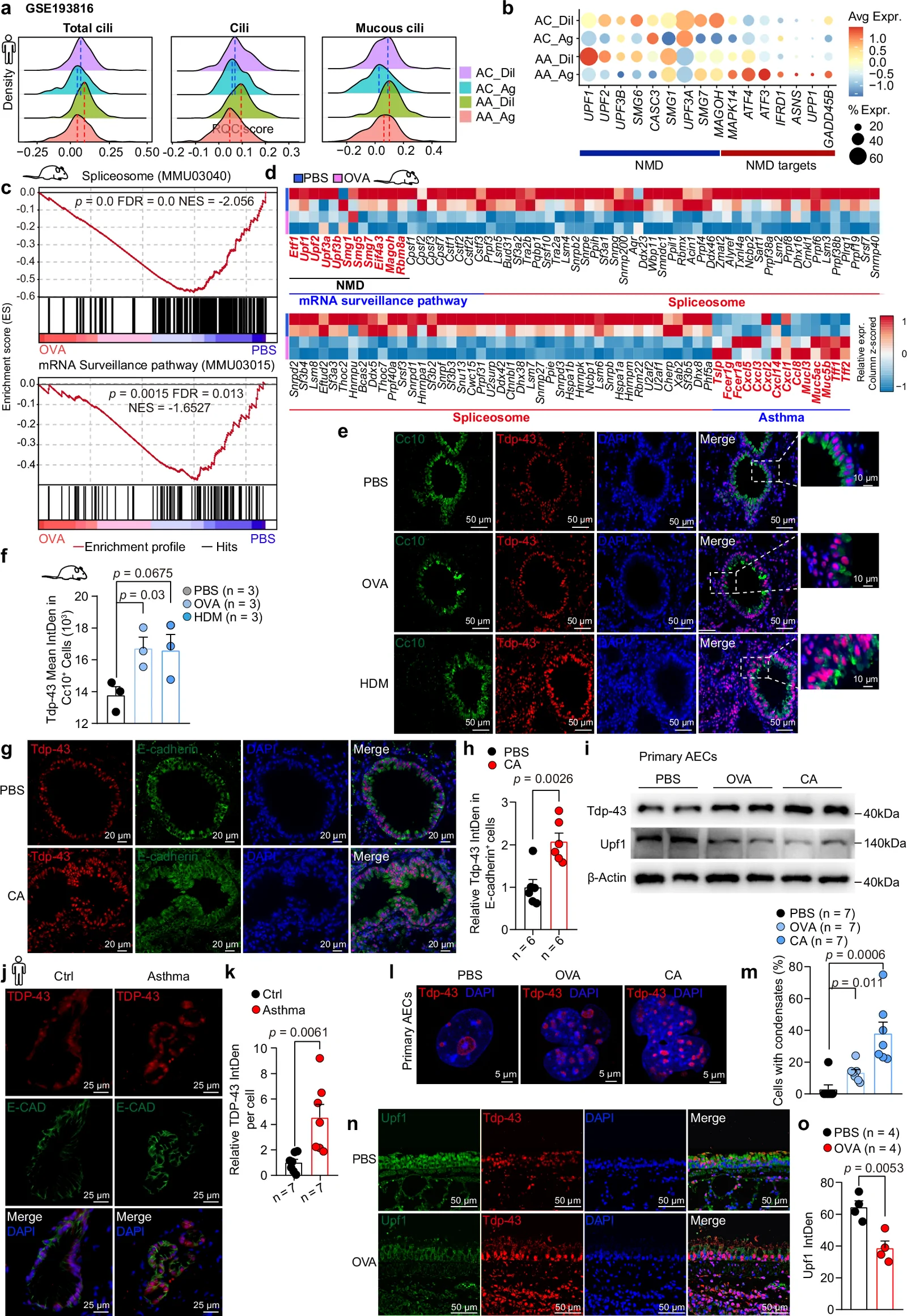
The expression of TDP-43 is dysregulated in the AECs from allergen-induced asthmatic mice and asthmatic patients. **a** Histogram plots comparing the RQC signature scores in ciliated cells from persons with exposure of allergen or diluent (GSE193816). The RQC signature scores were calculated with the AddModuleScore function in Seurat. **b** Dot plots comparing expressions of NMD-related genes and NMD targets across ciliated cell subpopulations from persons with allergen or diluent exposure. Ag: allergen, Dil: diluent. **c** GSEA plots of spliceosome and mRNA surveillance pathways in AECs under asthmatic and steady-state conditions. **d** Heatmap displaying expression of genes related to mRNA surveillance, spliceosome and asthma pathways in AECs under asthmatic and steady-state conditions. **e** Representative IF images of Tdp-43 in the airway epithelium from OVA/HDM-induced asthmatic mice. **f** Bar plots comparing Tdp-43 expression in Cc10^+^ cells in (**e**). **g** Representative IF images of Tdp-43 in the lungs from CA-induced asthmatic mice and control. **h** Bar plots comparing Tdp-43 expression in E-cadherin^+^ cells in (**g**). **i** Immunoblotting of Tdp-43 and Upf1 in the AECs isolated from CA/OVA-induced asthmatic mice and control. **j** Representative IF staining of TDP-43 and E-cadherin in the bronchoscopic mucosal specimens from patients with asthma and relatively healthy participants. **k** Quantitative results of TDP-43 IntDen per cell within E-Cadherin-positive regions in (**j**), each dot represents the mean value across fields for one patient, normalized to the mean of the control group. **l** Representative IF images of Tdp-43 in the AECs isolated from CA/OVA-induced mice and control mice. **m** Quantify the percentage of cells containing small Tdp-43 condensates in (**l**). **n** Representative IF staining of Upf1 and Tdp-43 in the lungs from OVA-induced asthmatic mice and control. **o** Quantitative IF results of Upf1 in (**n**). Data are presented as mean ± SEM. The data in **g**, **h**, **l**, **m** have at a minimum five independent biological replicates. The data in **e**, **i**, **n**, **o** represent two independent experiments. Significance was assessed by unpaired two-tailed Student’s *t*-test (**f**, **h**, **k**, **o**), and two-tailed Mann–Whitney *U* test (**m**). Source data are provided as a Source data file.

To assess RQC involvement in asthmatic responses, we inquired an RQC score based on RNA splicing/surveillance genes within a published single cell RNA sequencing (scRNA-seq) dataset of human airway mucosa (GSE193816) (Supplementary Table 1)^30^. The cohort included allergic asthmatics (AAs) and non-asthmatic allergic controls (ACs), all sensitized to standardized HDM or/and cat hair extract. Mucosal specimens were obtained 24 h after sequential segmental bronchial challenge with diluent and allergen (HDM or/and cat hair extract). Two clusters of ciliated cells (Cili) were identified and the mucous Cili cluster highly expressed *IL33*, *SCGB3A1*, *MUC5AC* and *SCGB1A1* (Supplementary Fig. 1a, b). Among both AAs and ACs, Cili and mucous Cili with allergen exposure displayed substantially decreased RQC scores compared to that of diluent exposure (Fig. 1a). Of note, compared to ciliated cells with diluent exposure, ciliated cells with allergen exposure under-expressed genes encoding components of NMD machinery (*UPF1*, *UPF2*, *UPF3B*, *SMG6* and *SMG1*) in both AC and AA individuals (Fig. 1b). Genes encoding components of spliceosome were also under-expressed in ciliated cells from individuals with allergen exposure (Supplementary Fig. 1c). However, accumulated expression of NMD targets were only observed in ciliated cells with allergen exposure from AA donors (Fig. 1b). These results suggest that AECs from AAs may possess compromised NMD activity.

To further confirm this finding, we established asthmatic mouse models induced by different allergens including ovalbumin (OVA), CA and HDM (Supplementary Fig. 1d, e). The flow cytometric results revealed that all the allergens induced Th2 infiltrations in the bronchoalveolar lavage fluid (BALF) (Supplementary Fig. 1f, g). Besides, distinct granulocyte inflammations were observed in mice exposed to different allergens: OVA and HDM induced eosinophilic inflammation, whereas CA led to neutrophil infiltration (Supplementary Fig. 1g). To investigate the expression patterns of RQC-related genes, we performed full-length transcript sequencing (smart-seq2) with purified AECs from OVA-induced asthmatic mice. Gene Set Enrichment Analysis (GSEA) results revealed that AECs from asthmatic mice had increased expression of genes enriched in asthma (MMU05310) (including genes encoding MHC class II components; inflammatory cytokines: *Tnf, Il10*; high affinity IgE receptors: *Fcer1g* and *Fcer1a*), mucin type O-glycan biosynthesis (MMU00512) and TNF signaling (MMU04668) pathways, but decreased expression of genes enriched in cilium movement (GO_0003341) (Supplementary Fig. 2a). Strikingly, asthmatic AECs under-expressed genes enriched in spliceosome (MMU03040) and mRNA surveillance pathway (MMU03015) compared to AECs under steady state (Fig. 1c). Of particular note, expressions of NMD-associated genes (*Etf1/eRF1*, *Upf1*, *Upf2*, *Upf3a*, *Upf3b*, *Smg1*, *Smg5* and *Smg7*) were significantly reduced in asthmatic AECs (Fig. 1d). AECs from asthmatic mice showed increased expression of genes involved in asthmatic response (*Tslp*, *Csf3*), encoding chemokines for granulocytes or monocytes (*Cxcl5*, *Cxcl3*, *Cxcl14*, *Cxcl1* and *Ccl8*) and mucin secretion (*Muc13*, *Muc5ac*, *Muc5b*, *Tff1* and *Tff2*) (Fig. 1d). These findings suggest that NMD is compromised in AECs from persons with asthma, and this phenomenon may be conserved in both murine and human.

Given the essential role of TDP-43 in RNA metabolism in MNs, we questioned whether TDP-43 expression is dysregulated in asthmatic airway epithelium. Under steady state, TDP-43 expression mainly existed in the nuclei of AECs from both mouse and human (Supplementary Fig. 2b, c). TDP-43 expression within the airway epithelium was significantly induced in mice post OVA or CA exposure (Fig. 1e–h). Elevated Tdp-43 expression was further confirmed in purified AECs from OVA or CA-induced asthmatic mice (Fig. 1i and Supplementary Fig. 2d, e). We further established an Air-Liquid Interface (ALI) culture with primary murine AECs. The ALIs were subjected to Il13 stimulation to recapitulate the effects of pro-Th2-derived cytokines on AECs in sensitization phase of allergen-induced asthma. Control group displayed well ciliated cell identity and integrity, as evidenced by Acetylated-Tubulin (Ac-Tubulin) staining, whereas the Il13 group displayed prominent mucus secretion demonstrated by Periodic Acid-Schiff (PAS) staining (Supplementary Fig. 2f). ALIs from Il13 group also had increased Tdp-43 expression compared with controls (Supplementary Fig. 2g, h). More importantly, TDP-43 expression was significantly upregulated in the airway epithelium of both asthmatic patients and asthma-COPD overlap syndrome (ACOS) persons (Fig. 1j, k, Supplementary Fig. 2i, j).

We further differentiated ALIs from AECs of asthmatic, COPD (which also have intrinsic airway epithelial abnormalities), and control donors, then treated them with CA or OVA (Supplementary Fig. 3a). Primary ALIs from asthmatic patients showed markedly higher *IL33* and *TSLP* expression than controls (Supplementary Fig. 3b), confirming inherent epithelial abnormalities in asthmatic individuals. TDP-43 expression was elevated in asthma/COPD ALIs compared with controls (Supplementary Fig. 3c). CA treatment further increased TDP-43 expression in ALIs from the asthma/COPD group but not from controls (Supplementary Fig. 3d, e), indicating that intrinsic AEC abnormality is prerequisite for allergen responsiveness. Direct OVA treatment on ALIs did not trigger TDP-43 upregulation (Supplementary Fig. 3d).

Interestingly, we found that in AECs from allergen-induced asthmatic mice, the nuclear distribution of Tdp-43 was dispersed into several smaller condensates (Fig. 1l–m), while Upf1, an essential component of NMD^28^, was markedly decreased (Fig. 1i, n, o). Therefore, these findings drove us to speculate that allergen-induced TDP-43 expression in AECs may drive asthmatic airway response through compromising UPF1-mediated NMD.

### *Tardbp* ablation in AECs reshapes the asthmatic airway microenvironment upon OVA stimulation

**Fig. 2:**
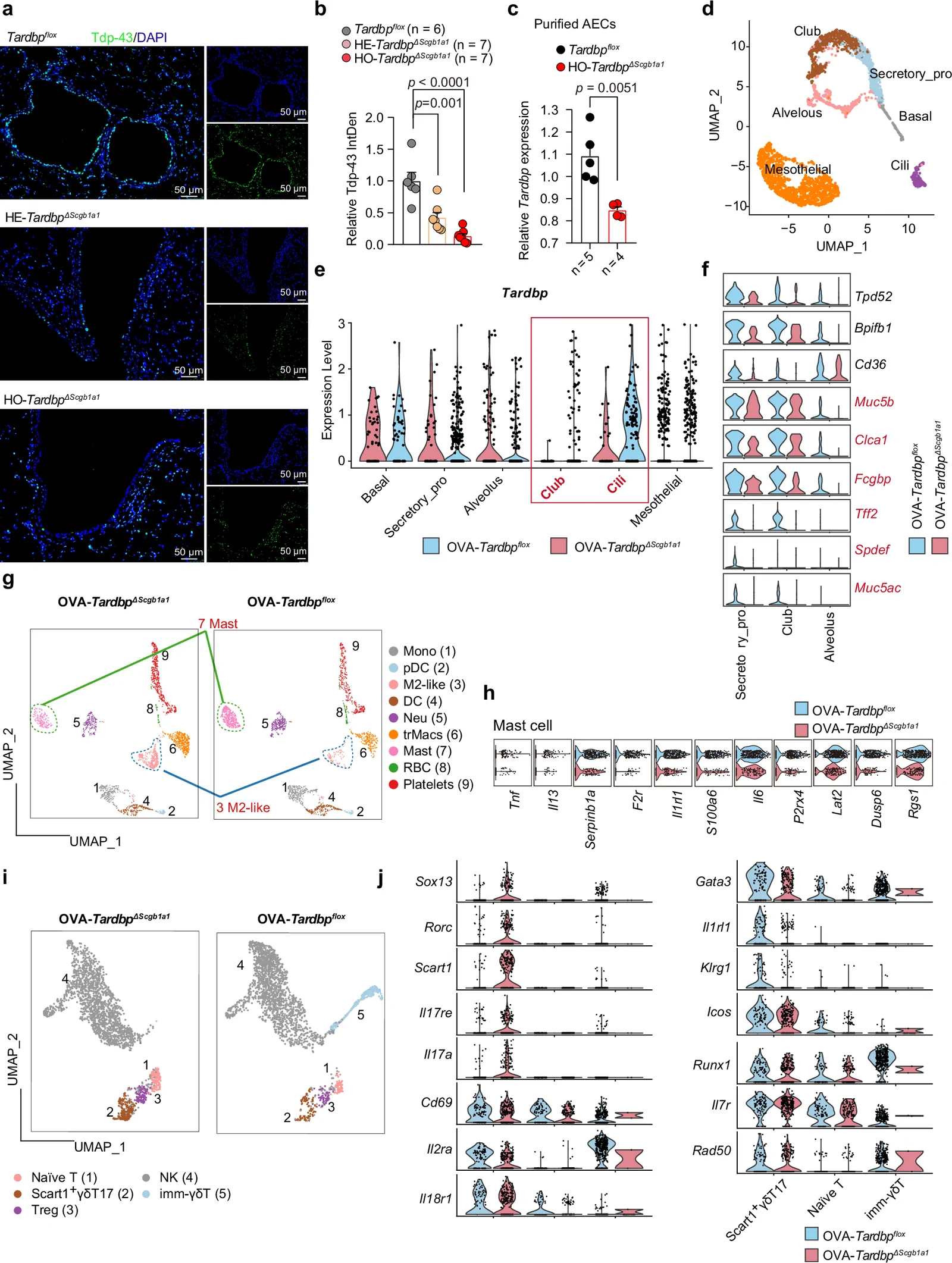
scRNA-seq analysis uncovers an alleviated asthmatic airway microenvironment in OVA-induced *Tardbp*^*ΔScgb1a1*^ mice. **a** Representative IF images of Tdp-43 in the lung tissues from *Tardbp*^*ΔScgb1a1*^ and *Tardbp*^*flox*^ mice. **b** Bar plots comparing the Integrated Density of Tdp-43 signals in (**a**), each dot represents an individual mouse. **c** mRNA level of *Tardbp* in primary AECs from *Tardbp*^*ΔScgb1a1*^ and *Tardbp*^*flox*^ mice. **d** UMAP plot showing the cell types of epithelium separately according to OVA-stimulated *Tardbp*^*ΔScgb1a1*^ and *Tardbp*^*flox*^ mice. **e** Violin plot showing the expression of *Tardbp* between OVA-stimulated *Tardbp*^*ΔScgb1a1*^ and *Tardbp*^*flox*^ mice in epithelial cell types. **f** Violin plot showing the expression of gene about mucin secretion in epithelial cell types. **g** UMAP plot showing the cell types of myeloid cells separately according to OVA-stimulated *Tardbp*^*ΔScgb1a1*^ and *Tardbp*^*flox*^ mice. **h** Violin plot showing the expression of gene related to granulocyte inflammation in mast cell. **i** UMAP plot showing the cell types of T cells separately according to OVA-stimulated *Tardbp*^*ΔScgb1a1*^ and *Tardbp*^*flox*^ mice. **j** Violin plot showing the expression of gene related to type 2 inflammatory stimulation in T cell types. Data are presented as mean ± SEM. The data in **a**, **b** have at a minimum 6 independent biological replicates. Experiments in **c** was performed in two independent replicates, and representative data from one replicate are presented. Significance was assessed by unpaired two-tailed Student’s *t*-test (**c**), and one-way ANOVA with Tukey’s multiple comparisons test (**b**). Source data are provided as a Source data file.

To investigate the in vivo function of Tdp-43 in AECs during the asthmatic airway response, we generated a transgenic mouse strain with specific *Tardbp* ablation in AECs by crossing the *Tardbp*^*flox*^ strain with *Scgb1a1*^*Cre*^ mice (*Tardbp*^*ΔScgb1a1*^) (Supplementary Fig. 4a). Scgb1a1/Cc10 marks club cells, which serve as the somatic stem cells responsible for the regeneration of airway epithelium^31^. Club cells replenish various types of epithelial cells within the airway epithelium, including ciliated and goblet cells^32^. IF results verified the substantial depletion of Tdp-43 within the bronchial epithelium in the homozygous *Tardbp*^*ΔScgb1a1*^ mice (HO-*Tardbp*^*ΔScgb1a1*^) (Fig. 2a, b). Decreased *Tardbp* expression was further validated in AECs from HO-*Tardbp*^*ΔScgb1a1*^ (Fig. 2c). No discernible pulmonary abnormalities were observed in HO-*Tardbp*^*ΔScgb1a1*^ mice raised in specific pathogen free (SPF) environment (Supplementary Fig. 4b).

We performed scRNA-seq analysis on enriched nonimmune cells harvested from trachea and lungs of both *Tardbp*^*ΔScgb1a1*^ and *Tardbp*^*flox*^ mice with OVA exposure. After stringent filtering, 30872 high quality cells were obtained. We were able to identify 6 epithelial, 6 endothelial, and 5 stromal subpopulations (Supplementary Fig. 4c, d and Supplementary Table 2). The epithelium compartment comprises of basal cells (*Krt19*, *Krt6a*, *Krt13*), secretory progenitors (secretory_pro) (*Lgr6*, *Msln*, *Muc16*, *Krt8*, *Wfdc2*), club cells (*Scgb1a1*, *Scgb3a2*, *Muc5b*), ciliated cells (cili) (*Foxj1*, *Cfap44*) and alveolar cells (*Ager*, *Sftpc*) (Fig. 2d and Supplementary Fig. 4e). In *Tardbp*^*ΔScgb1a1*^ mice, *Tardbp* expression was down-expressed specifically in club and ciliated cells but remained intact in basal cells and alveolar epithelium (Fig. 2e), confirming the targeted cell specificity of *Scgb1a1*^*cre*^. The secretory-pro, club, and alveolar cells from OVA-*Tardbp*^*ΔScgb1a1*^ mice exhibited a reduced capacity of mucin secretion compared to those from OVA-*Tardbp*^*flox*^ mice (lower expression of *Muc5ac*, *Spdef*, *Tff2*, *Fcgbp*, *Clca1*) (Fig. 2f). This observation suggests an alleviation of airway metaplasia in the OVA-*Tardbp*^*flox*^ group.

We also detected residual immune cells, including 5 natural killer cells (NK)/T cells, 4 B cells, and 9 myeloid cell subpopulations, attributed to non-specific binding in the magnetic enrichment process (Supplementary Fig. 4c, d and Supplementary Table 2). Nevertheless, notably reduced mast cell infiltrations (*Fcer1g* and *Mcpt8*) were observed in the lungs of OVA-*Tardbp*^*ΔScgb1a1*^ mice (Fig. 2g and Supplementary Fig. 4f). Furthermore, mast cells from OVA-*Tardbp*^*ΔScgb1a1*^ group also under-expressed genes related to granulocyte inflammation (*Tnf*, *Il13*, *Il1rl1*, *Il6*, *S100a6*, *Serpinb1a*, *F2r*, *P2rx4*) (Fig. 2h). Besides, the lungs of OVA-*Tardbp*^*ΔScgb1a1*^ mice exhibited an increased abundance of M2-like macrophages, which possess immunomodulatory and tissue-repair properties (*Mertk*, *Chil3*, *Arg1*, *Mrc1*) (Fig. 2g and Supplementary Fig. 4f).

Scart1^+^ γδT17 cells are mucosal-resident lymphocytes characterized by a Th17-like transcriptional signature that is essential for maintaining mucosal homeostasis^33^. In OVA-*Tardbp*^*flox*^ mice, this population underwent a trans-differentiation from its steady-state mucosal-γδT17 identity (marked by lower expression of *Sox13*, *Rorc*, *Scart1*, *Il17re*, and *Il17a*) toward an activated, Th2-like state, evidenced by elevated expression of *Cd69*, *Il2ra*, *Gata3*, *Il1rl1*, *Klrg1*, and *Runx1*(Fig. 2i, j and Supplementary Fig. 4g)^34^. Interestingly, a population of immature γδT cells (imm-γδT) (*Dntt*^+^*Rag1*^+^*Ptcra*^+^) was specifically detected in the lungs from OVA-*Tardbp*^*flox*^ mice (Fig. 2i and Supplementary Fig. 4g). Notably, both these imm-γδT cells and naïve T cells in OVA-*Tardbp*^*flox*^ mice similarly acquired a Th2-like program (Fig. 2j). In sharp contrast, the acquisition of the Th2-program, observed across Scart1^+^γδT17, imm-γδT and naïve T cells in OVA-*Tardbp*^*flox*^ mice, was blunted in the OVA-*Tardbp*^*ΔScgb1a1*^ mice (Fig. 2j). Together, the scRNA-seq analysis indicates that allergen exposure induced a less pronounced asthmatic airway microenvironment in *Tardbp*^*ΔScgb1a1*^ mice compared to *Tardbp*^*flox*^ mice.

### *Tardbp*^*ΔScgb1a1*^ mice are resistant to either OVA- or CA- induced asthmatic airway response

**Fig. 3:**
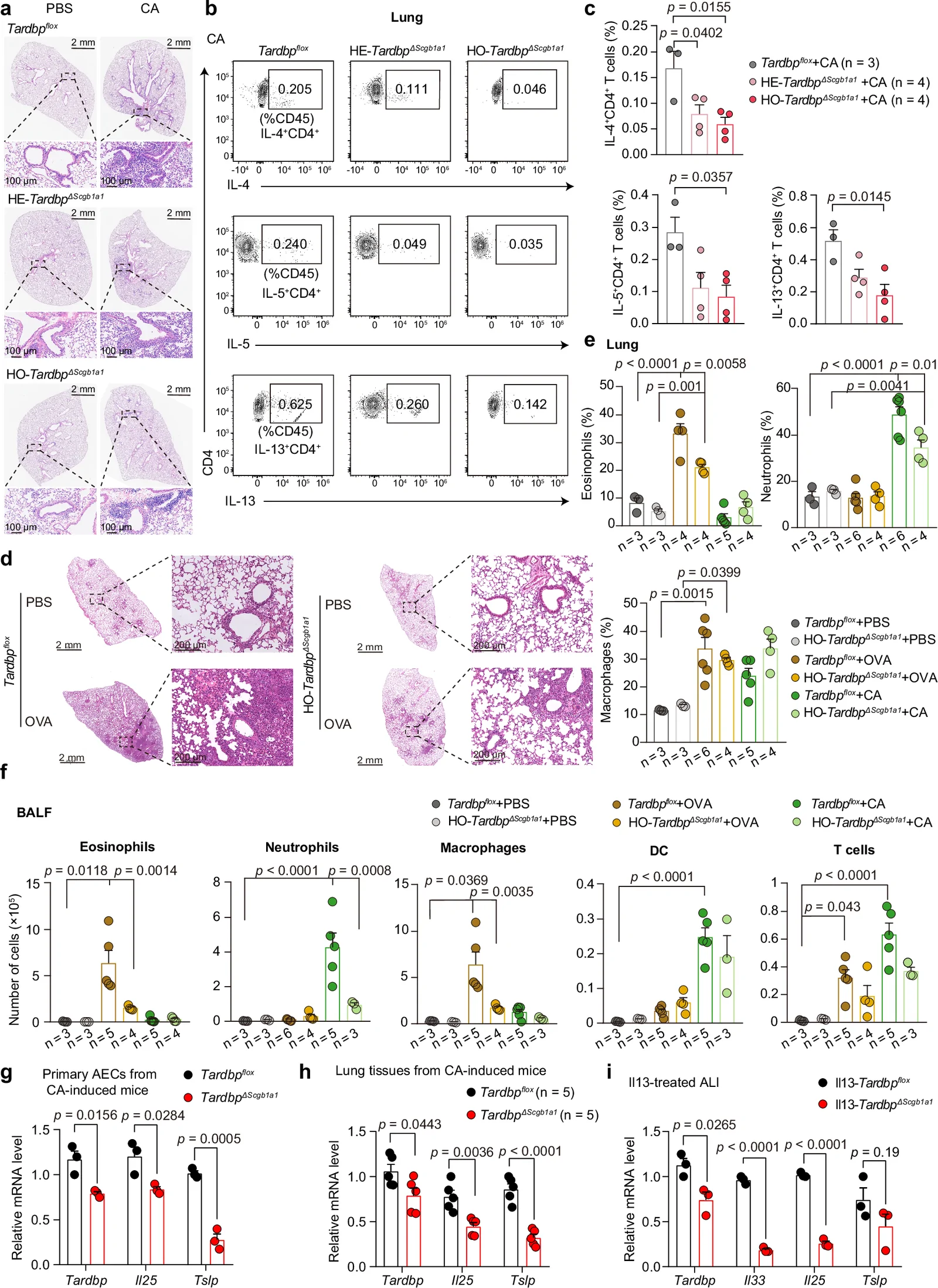
*Tardbp*^*ΔScgb1a1*^ mice are hyposensitive to allergen-induced asthma modeling. **a** H&E staining comparing pathologies between CA-induced *Tardbp*^*ΔScgb1a1*^ and *Tardbp*^*flox*^ mice. **b** Representative flow plots showing pulmonary infiltrations of Th2 across CA-induced HO-*Tardbp*^*ΔScgb1a1*^, HE-*Tardbp*^*ΔScgb1a1*^, and *Tardbp*^*flox*^ mice. **c** Bar plots summarizing the flow cytometric results in (**b**). **d** H&E staining comparing pathologies between OVA-induced *Tardbp*^*ΔScgb1a1*^ and *Tardbp*^*flox*^ mice. **e** Bar plots showing flow cytometric results of myeloid cell infiltrations in the lung tissues of *Tardbp*^*ΔScgb1a1*^ and *Tardbp*^*flox*^ mice. **f** Bar plots showing flow cytometric results of immune cell infiltrations in BALF specimens from *Tardbp*^*ΔScgb1a1*^ and *Tardbp*^*flox*^ mice. **g** mRNA levels of *Tardbp*, *Il25* and *Tslp* in primary AECs isolated from CA-induced *Tardbp*^*ΔScgb1a1*^ and *Tardbp*^*flox*^ mice. **h** mRNA levels of *Tardbp*, *Il25* and *Tslp* in lung tissues from CA-induced *Tardbp*^*ΔScgb1a1*^ and *Tardbp*^*flox*^ mice. **i** mRNA levels of *Tardbp*, *Il33*, *Il25* and *Tslp* in primary AECs isolated from CA-induced *Tardbp*^*ΔScgb1a1*^ and *Tardbp*^*flox*^ mice and differentiated under air-liquid interface conditions. Data are presented as mean ± SEM. Experiments in **b**,** c**,** e**–**i** were performed in two independent replicates, and representative data from one replicate are presented. Significance was assessed by unpaired two-tailed Student’s *t*-test (**g**, **h**, **i**), one-way ANOVA with Tukey’s multiple comparisons test (**c**, **e**, **f**), and Kruskal–Wallis test with Dunn’s multiple comparisons test (**c**,** f**). Source data are provided as a Source data file.

Consistent with the scRNA-seq results, *Tardbp*^*ΔScgb1a1*^ mice manifested significantly alleviated pulmonary pathologies upon CA-induced neutrophilic (non-T2) asthma modeling, which was dose-dependently correlated to the extent of Tdp-43 loss (Fig. 3a). Flow cytometric results revealed profoundly reduced infiltrations of Th2 in the lungs and significantly decreased neutrophil abundance in the lungs and BALF from CA-induced asthmatic HO-*Tardbp*^*ΔScgb1a1*^ mice (Fig. 3b, c, e, f). The *Tardbp*^*ΔScgb1a1*^ mice also exhibited ameliorated pathology upon OVA-induced eosinophilic (T2) asthma modeling (Fig. 3d). HO-*Tardbp*^*ΔScgb1a1*^ mice displayed substantially reduced eosinophil infiltrations in the lungs and markedly decreased infiltrations of eosinophils and macrophages in the BALF upon OVA-induced asthma modeling (Fig. 3e, f). Furthermore, in CA-induced *Tardbp*^*ΔScgb1a1*^ mice, expression levels of epithelial alarmin genes (*Il25* and *Tslp*) were significantly downregulated, both in isolated primary AECs and while lung tissues (Fig. 3g, h). These reductions were also recapitulated ex vivo, where ALI cultures derived from *Tardbp*^*ΔScgb1a1*^ mice with CA exposure showed diminished *Il25* and *Tslp* induction compared to ALIs derived from CA-induced *Tardbp*^*flox*^ mice (Fig. 3i). These results indicate that *Tardbp*^*ΔScgb1a1*^ mice were hyposensitive to both OVA-induced T2 and CA-induced non-T2 asthmatic airway response.

### Dysregulated TDP-43 expression contributes to a molecular signature of impaired NMD in asthmatic AECs

**Fig. 4:**
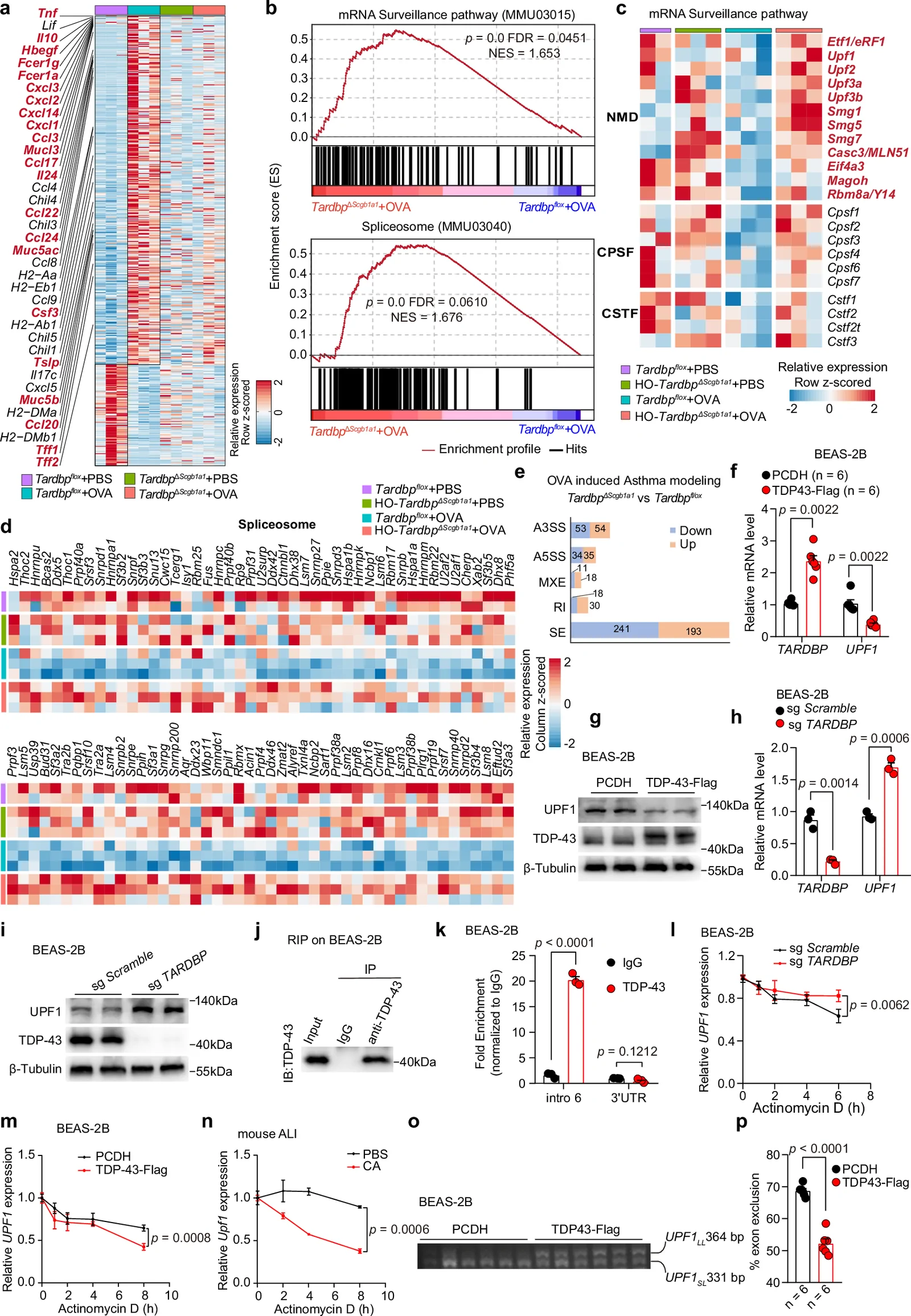
Allergen-induced TDP-43 expression dampens UPF1-mediated NMD in AECs. **a** Heatmap showing the expression of AHR-related genes in AECs from allergen-stimulated *Tardbp*^*ΔScgb1a1*^ and *Tardbp*^*flox*^ mice. **b** GSEA analysis showing gene sets of mRNA surveillance pathway and spliceosome in AECs from OVA-induced *Tardbp*^*ΔScgb1a1*^ and *Tardbp*^*flox*^ mice. Heatmap comparing expressions of genes related to mRNA surveillance pathway (**c**) and spliceosome (**d**) across AECs from *Tardbp*^*ΔScgb1a1*^ and *Tardbp*^*flox*^ mice with or without OVA stimulation. **e** Stacked histograms showing the occurrence of altered AS events between AECs isolated from OVA-stimulated *Tardbp*^*flox*^ and *Tardbp*^*ΔScgb1a1*^ mice. SE: skipped exon; A5SS: selective 5’ (donor) splicing site; A3SS: selective 3’ (acceptor) splicing site; RI: retained intron; MXE: mutually exclusive exon. mRNA (**f**) and protein (**g**) levels of TARDBP and UPF1 in TDP-43-overexpressed BEAS-2B cells. mRNA (**h**) and protein (**i**) levels of TARDBP and UPF1 in TDP-43-knockdown BEAS-2B cells. **j** Immunoblotting of TDP-43 after RIP assay with TDP-43 antibody in BEAS-2B cells. **k** qPCR analysis of RNA pulled down by TDP-43 antibody in BEAS-2B cells, using primers targeting the intron6 or 3’UTR of *UPF1*. mRNA level of *UPF1* in TDP-43-knockdown (**l**) or TDP-43-overexpressed (**m**) BEAS-2B cells treated with 10 μg/ml Actinomycin D for indicated time points. **n** mRNA level of *Upf1* in allergen (CA)-induced and control AECs under air-liquid interface conditions, followed by treated with 10 μg/ml Actinomycin D for indicated time points. **o** Agarose gel separation of long and short minigene splicing variants in RT-PCR products. **p** Quantitative results of long and short variants in (**o**). Data are presented as mean ± SEM. The data in **f**–**k**,** o**,** p** represent three independent experiments, and **l**, **m** represent two independent experiments. Representative data from one replicate are presented. Significance was assessed by unpaired two-tailed Student’s *t*-test (**h**, **k**,** p**), two-tailed Mann–Whitney *U* test (**f**), and two-way ANOVA (**l**–**n**). Source data are provided as a Source data file.

To gain mechanistic insights of TDP-43 involvement in allergen-induced asthmatic airway response, we performed smart-seq2-seq on AECs isolated from *Tardbp*^*ΔScgb1a1*^ and *Tardbp*^*flox*^ mice under OVA-induced asthmatic state (Supplementary Fig. 5a). AECs from *Tardbp*^*ΔScgb1a1*^ mice with OVA exposure failed to mount the asthmatic transcriptomic program as control mice did (Fig. 4a). GSEA and heatmap results showed that AECs from *Tardbp*^*ΔScgb1a1*^ mice with OVA exposure under-expressed genes enriched in asthma (MMU05310), mucin type O-glycan biosynthesis (MMU00512), TNF signaling pathway (MMU04668) pathways (Supplementary Fig. 5b), but over-expressed genes enriched in cilium assembly (GO_0060271) and cilium movement (GO_0003341) pathways (Supplementary Fig. 5c, d), indicating that *Tardbp*^*ΔScgb1a1*^ mice retained airway epithelium integrity under allergen exposure.

Of particular note, AECs from *Tardbp*^*ΔScgb1a1*^ mice with OVA challenge retained expressions of genes in spliceosome (MMU03040) and mRNA surveillance pathway (MMU03015) compared to AECs from *Tardbp*^*flox*^ mice (Fig. 4b–d). Moreover, NMD-related gene expressions in AECs from OVA-challenged *Tardbp*^*ΔScgb1a1*^ mice remained comparable to that in AECs from steady-state *Tardbp*^*flox*^ mice (Fig. 4c), indicating that TDP-43 determines allergen-induced NMD degeneration in AECs. Asthmatic AECs from *Tardbp*^*flox*^ mice had significant number of altered alternative splicing (AS) events and remarkably higher expression of transcripts related to asthmatic response (*Chrm1*, *Noxa1*, *Abcc8*, *Ecm1*, *Slc28a3*, *Pdzk1ip1*) (Fig. 4e)^35,36^, which also displayed different splicing patterns compared to that from *Tardbp*^*ΔScgb1a1*^ mice with OVA challenge (Supplementary Fig. 5e, f).

### TDP-43 modulates UPF1 stability in asthmatic AECs

To validate the RNA-seq results, we examined the expression of UPF1, encoding the RNA-dependent helicase UPF1 (the rate-limiting component of NMD). Strikingly, we observed that UPF1 was downregulated upon TDP-43 overexpression, while being significantly upregulated at both mRNA and protein level in TDP-43-knockdown BEAS-2B cells (Fig. 4f–i). In intron6 of *UPF1*, there are 4 tandem UG repeats (UG)_4_ for potential binding of TDP-43^37^ (Supplementary Fig. 5g), which was further verified by performing RNA immunoprecipitation coupled with quantitative real-time polymerase chain reaction (RIP-qPCR) with anti-TDP-43 antibody on BEAS-2B cells (Fig. 4j, k). Results of RNA decay assay showed that *UPF1* stability was enhanced in BEAS-2B cells with TDP-43 knockdown, conversely, it was reduced in BEAS-2B with TDP-43 overexpression (Fig. 4l, m). RNA decay assay on murine ALIs further demonstrated that CA stimulation significantly destabilized *Upf1* mRNAs (Fig. 4n).

In mammals, *UPF1* has two isoforms derived from conserved alternative 5’ splice site usage in exon7, generating *UPF1* with different regulatory loop lengths (*UPF1*_*SL*_ and *UPF1*_*LL*_ with 33 nt longer) (Supplementary Fig. 5h)^38^. Under steady state, *UPF1*_*LL*_ accounts for only 15–25% of total *UPF1* mRNA^39^. As the TDP-43-binding site in intron6 is in proximity to the alternative 5’ splice site in exon7, we wondered whether excess TDP-43 expression could regulate alternative splicing of *UPF1*. We performed minigene splicing assay by subcloning the partial intron6-exon7-intron7 region of *UPF1* into the pET01 construct, flanked by exons A and B (Supplementary Fig. 5g)^40^. The minigene construct was transfected into BEAS-2B cells for 24 h and the splicing patterns of the inserted *UPF1* genomic segment were analyzed by Reverse transcription-PCR (RT-PCR) using indicated primers depicted in Supplementary Fig. 5g. The minigene splicing analysis demonstrated that both the short (331 nt) and long (364 nt) isoforms were detectable in BEAS-2B cells, with the short isoform predominating (Fig. 4o). Overexpression of TDP-43 enhanced the inclusion of the 33 nt fragment, thereby increasing the proportion of the long isoform (Fig. 4o, p). Together, these findings indicate that allergen-induced TDP-43 expression in AECs modulates *UPF1* splicing and stability.

### CHRM1 transcripts accumulate in asthmatic AECs with dampened NMD

NMD perceives mRNAs either through PTCs in proximity to exon-junction complexes (EJCs) in the ORF, or via uORFs in 5’UTR, very long 3’UTR or 3’UTR-located EJCs, and triggers their degradation^41^. We speculated that compromised NMD in asthmatic AECs gives rise to the accumulation of NMD-sensitive transcripts driving asthmatic response, which shall be inspected and eliminated in AECs with sound NMD activity. We observed multiple genes with differential alternative splicing patterns between AECs from allergen-induced *Tardbp*^*ΔScgb1a1*^ and *Tardbp*^*flox*^ mice (Supplementary Fig. 5e). Among these candidate genes, prediction analyses (using the Ribo-uORF database, https://www.compgen.uni-muenster.de/tools/uorfdb) showed that the *CHRM1* transcripts in both human and mouse contain multiple uORFs in their 5’UTRs (Supplementary Table 3, 4). Besides, large-scale genome-wide association study revealed that polymorphisms in *CHRM1* confer susceptibility to asthma^35^. Therefore, we took *CHRM1* to dissect the underlying mechanisms.

**Fig. 5:**
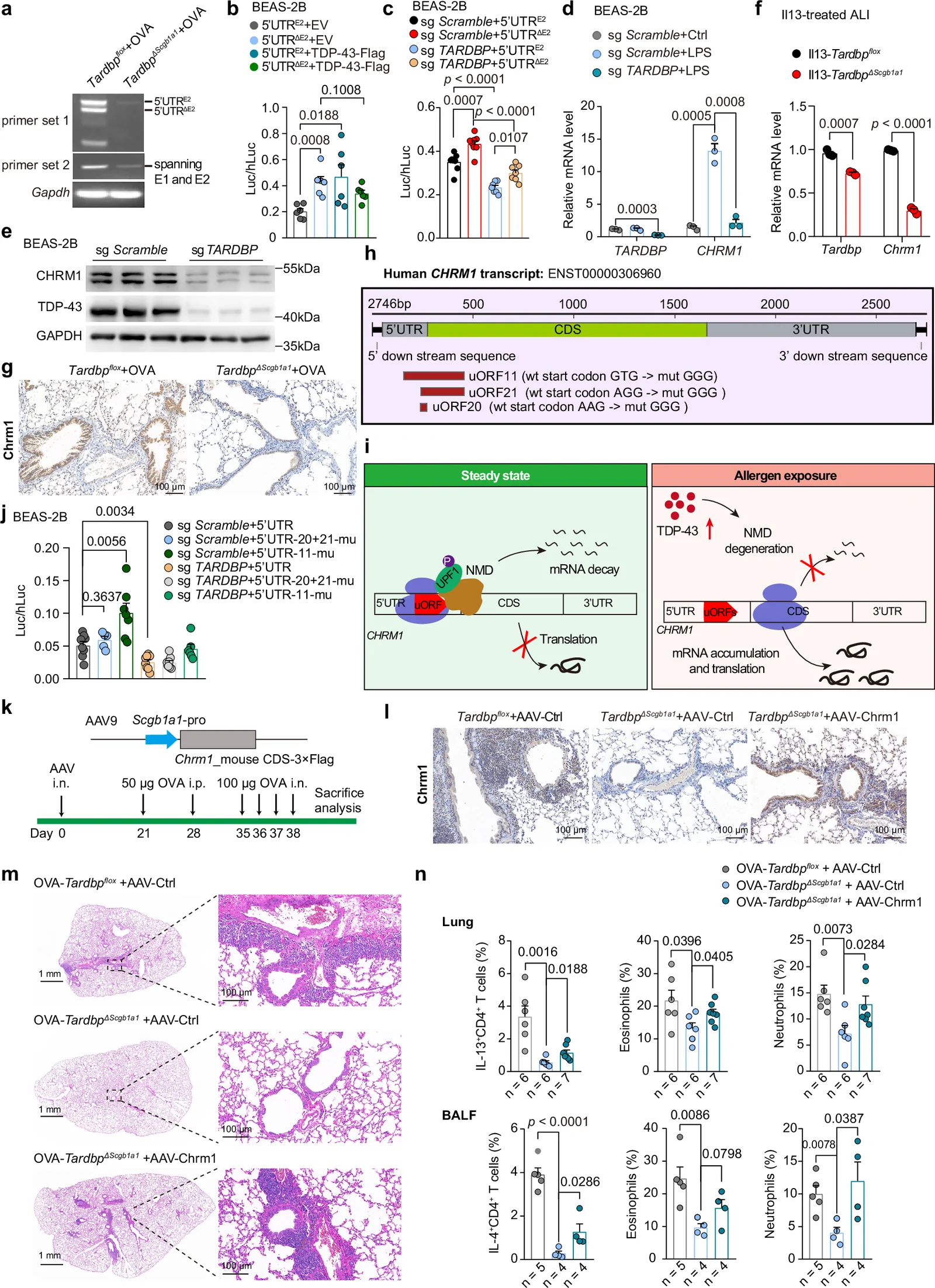
Allergen-induced TDP-43 expression leads to accumulation of NMD-sensitive *Chrm1* transcripts in AECs. **a** Alternatively spliced transcripts of *Chrm1* were examined by RT-PCR in purified AECs from OVA-induced *Tardbp*^*ΔScgb1a1*^ and *Tardbp*^*flox*^ mice. **b** Bar charts comparing relative Luc activities (Luc/hLuc) directed by 5’UTR^E2^ and 5’UTR^ΔE2^ of mouse *Chrm1* in BEAS-2B cells with or without TDP-43 overexpression. **c** Bar charts comparing relative Luc activities directed by 5’UTR^E2^ and 5’UTR^ΔE2^ of mouse *Chrm1* in BEAS-2B cells with or without TDP-43 ablation. mRNA (**d**) and protein (**e**) levels of CHRM1 and TARDBP in TDP-43-knockdown BEAS-2B cells with or without LPS treatment. **f** mRNA levels of *Chrm1* and *Tardbp* in primary AECs isolated from CA-induced *Tardbp*^*ΔScgb1a1*^ and *Tardbp*^*flox*^ mice and differentiated under air-liquid interface conditions. **g** Representative IHC images of Chrm1 in the airway epithelium from OVA-induced *Tardbp*^*ΔScgb1a1*^ and *Tardbp*^*flox*^ mice. **h** The locations of uORFs within the 5’UTR of human *CHRM1* transcript. **i** Sketch graphics proposing that allergen-induced NMD degeneration gives rise to *CHRM1* transcript accumulation and expression in asthmatic AECs. **j** Bar charts comparing relative Luc activities (Luc/hLuc) directed by 5’UTR between site-directed mutagenesis on the start codons of predicted uORFs of human CHRM1’s in BEAS-2B cells. **k** Schematic diagrams depicting the construct of AAV-Chrm1 and the complementation experiment on *Tardbp*^*ΔScgb1a1*^ with AAV-Chrm1. **l** Representative IHC images of Chrm1 in the airway epithelium from OVA-induced *Tardbp*^*ΔScgb1a1*^ and *Tardbp*^*flox*^ mice receiving i.n. infection of AAV-Ctrl or AAV-Chrm1. **m** H&E staining comparing pathologies of OVA-induced *Tardbp*^*flox*^ and *Tardbp*^*ΔScgb1a1*^ mice receiving i.n. infection of AAV-Ctrl or AAV-Chrm1. **n** Bar plots showing flow cytometric results of immune cell percentage in the lung tissues and BALF of OVA induced- *Tardbp*^*ΔScgb1a1*^ and *Tardbp*^*flox*^ mice receiving i.n. infection of AAV-Ctrl or AAV-Chrm1. Data are presented as mean ± SEM. The data in **a**–**f**, **j**, **n** represent two independent experiments, and representative data from one replicate are presented. Significance was assessed by unpaired two-tailed Student’s *t*-test (**b**, **d**, **f**, **j**, **n**), two-tailed Mann–Whitney *U* test (**n**), and one-way ANOVA with Tukey’s multiple comparisons test (**c**). Source data are provided as a Source data file.

*CHRM1* is a G protein-coupled receptor transducing cellular responses by recognizing vagal neurotransmitter Ach. It is well-acknowledged that Ach not only promotes contraction of airway smooth muscle cells (ASMCs), but also actively contributes to airway inflammation and mucus production through binding to muscarinic receptors expressed on AECs^12,13^. CHRM1 on AECs also regulates cell proliferation and chemokine production^42,43^. Mouse *Chrm1* has two variants with identical coding sequence (CDS) and 3’UTR, the only difference is that variant 1 has longer 5’UTR with inclusion of exon2 (5’UTR^E2^), which is absent from the 5’UTR of variant 2 (5’UTR^ΔE2^) (Supplementary Fig. 6a). RT-PCR detection with the primer sets depicted in Supplementary Fig. 6a showed that asthmatic AECs from *Tardbp*^*flox*^ mice had mixed pool of *Chrm1*−5’UTR^E2^ and −5’UTR^ΔE2^, whereas in AECs from *Tardbp*^*ΔScgb1a1*^ mice with OVA exposure, *Chrm1*−5’UTR^E2^ level was substantially low and −5’UTR^ΔE2^ was undetectable (Fig. 5a). We subcloned the 5’UTR^E2^ and 5’UTR^ΔE2^ of *Chrm1* into a luciferase (Luc) reporter vector to measure their capacities in driving Luc expression (Supplementary Table 5 and Supplementary Fig. 6b). Both 5’UTR^E2^ and 5’UTR^ΔE2^ could direct Luc expression in 293T and BEAS-2B cells, and 5’UTR^ΔE2^ exhibited superior activity than 5’UTR^E2^ (Fig. 5b and Supplementary Fig. 6c). Overexpression of TDP-43 enhanced Luc expression (Fig. 5b and Supplementary Fig. 6c, d), whereas TDP-43 knockdown suppressed Luc expression (Fig. 5c). Consistent with the luciferase assay results, we found that CHRM1 expression were substantially attenuated on TDP-43 knockdown in BEAS-2B (Fig. 5d, e and Supplementary Fig. 6e). Besides, ALI cultures derived from *Tardbp*^*ΔScgb1a1*^ mice had decreased *Chrm1* expression compared to those from *Tardbp*^*flox*^ mice (Fig. 5f). *Chrm1* expression was also downregulated in the lung tissues from *Tardbp*^*ΔScgb1a1*^ mice than in *Tardbp*^*flox*^ mice upon CA exposure (Supplementary Fig. 6f). IHC results confirmed diminished Chrm1 expression within airway epithelium from *Tardbp*^*ΔScgb1a1*^ mice with OVA exposure (Fig. 5g).

*CHRM1* transcripts in both human and mouse have long 3’UTRs (human ENST00000306960 3’UTR 1039 bp; mouse ENSMUST00000035444 3’UTR and ENSMUST00000163785 4291 bp) and multiple uORFs in their 5’UTRs, several of which possess adequate or strong Kozak strength (Fig. 5h, Supplementary Tables 3, 4, and Supplementary Fig. 6g), suggesting that in NMD-competent cells, *CHRM1* transcripts are inspected and degraded by NMD pathway, whereas in cells with compromised NMD activity, these transcripts can accumulate and be robustly expressed (Fig. 5i). Human *CHRM1* 5’UTR with start codon mutation in uORF11 exhibited significantly enhanced capacity to direct Luc expression in BEAS-2B cells (Fig. 5h, j), meanwhile, CHRM1 expression was substantially increased upon UPF1 knockdown (Supplementary Fig. 6h), confirming that *CHRM1* is a NMD target.

### Re-expression of Chrm1 on AECs restores OVA-induced asthmatic airway response in *Tardbp*^*ΔScgb1a1*^ mice

We proceeded to ask whether Chrm1 is a major downstream effector of TDP-43, so we conducted rescue experiments by intranasally (i.n.) administering adeno-associated virus serotype 9 (AAV9) carrying *Chrm1* CDS directed by the promotor of Scgb1a1/Cc10 and the C-terminal of Chrm1 was Flag tagged (AAV-Chrm1-Flag (Fig. 5k and Supplementary Table 6). mIF results validated that Chrm1-Flag expression was restricted to E-cadherin^+^ epithelium (Supplementary Fig. 6i, j). We observed that *Tardbp*^*flox*^ mice with prior AAV-Chrm1-Flag infection displayed severer lung pathologies and had markedly greater infiltrations of Th2 (IL-4^+^CD4^+^) and Th1 (IFN-γ^+^CD4^+^) cells in the BALF upon OVA stimulation when compared to *Tardbp*^*flox*^ mice with AAV-Ctrl infection, implying the potential of Chrm1 in promoting asthmatic response (Supplementary Fig. 6k, l). IHC staining demonstrated re-expression of Chrm1 in the airway epithelium of the *Tardbp*^*ΔScgb1a1*^ mice following AAV-Chrm1 infection (Fig. 5l). More importantly, restored expression of Chrm1 in AECs of *Tardbp*^*ΔScgb1a1*^ mice rescued asthmatic pathologies and reconstructed the pulmonary inflammatory microenvironment (Fig. 5m, n and Supplementary Fig. 6m), indicating that Chrm1 re-expression in AECs substantially rescued the sensitivity of *Tardbp*^*ΔScgb1a1*^ mice to OVA-induced asthmatic airway response.

In addition to *Chrm1*, we also examined another NMD-sensitive transcript *ATF4*. ATF4 is a master transcription factor in endoplasmic reticulum (ER) stress. Accumulating evidence indicates that ER stress is closely associated with asthmatic airway response, as evidenced in both allergen-induced murine models of asthma and airway mucosal brushing samples from asthmatic patients^15,16^. Inhibition of ATF4-involved ER stress could suppress asthmatic airway response^17^. *ATF4* is a well-documented NMD target and has several uORFs in the 5’UTR (Supplementary Fig. 7a)^44^, and we found that accumulated ATF4 expression was observed upon UPF1 knockdown in BEAS-2B cells (Supplementary Fig. 7b). In lung tissues from mice with CA exposure, we detected a significant upregulation of Atf4 signal compared to that from control mice (Supplementary Fig. 7c, d). Furthermore, *Tardbp*^*ΔScgb1a1*^ mouse lungs showed a marked downregulation of Atf4 relative to *Tardbp*^*flox*^ mice upon CA challenging (Supplementary Fig. 7e, f). In AECs isolated from CA-stimulated *Tardbp*^*ΔScgb1a1*^ mice, *Atf4* mRNA level was significantly downregulated compared to control mice (Supplementary Fig. 7g). Besides, *TARDBP*-knockdown in BEAS-2B cells markedly reduced ATF4 expression at both mRNA and protein levels (Supplementary Fig. 7h, i). Conversely, *TARDBP* overexpression significantly increased *ATF4* expression (Supplementary Fig. 7j). Taken together, these results demonstrate that allergen-exposure impaired NMD activity in AECs, leading to uncontrolled accumulation of NMD-sensitive transcripts including *CHRM1* and *ATF4,* which encode pathogenic molecules mediating asthmatic airway response to allergens.

### *Tardbp*^*ΔScgb1a1*^ mice are resistant to IAV infection and IAV-AEs

**Fig. 6:**
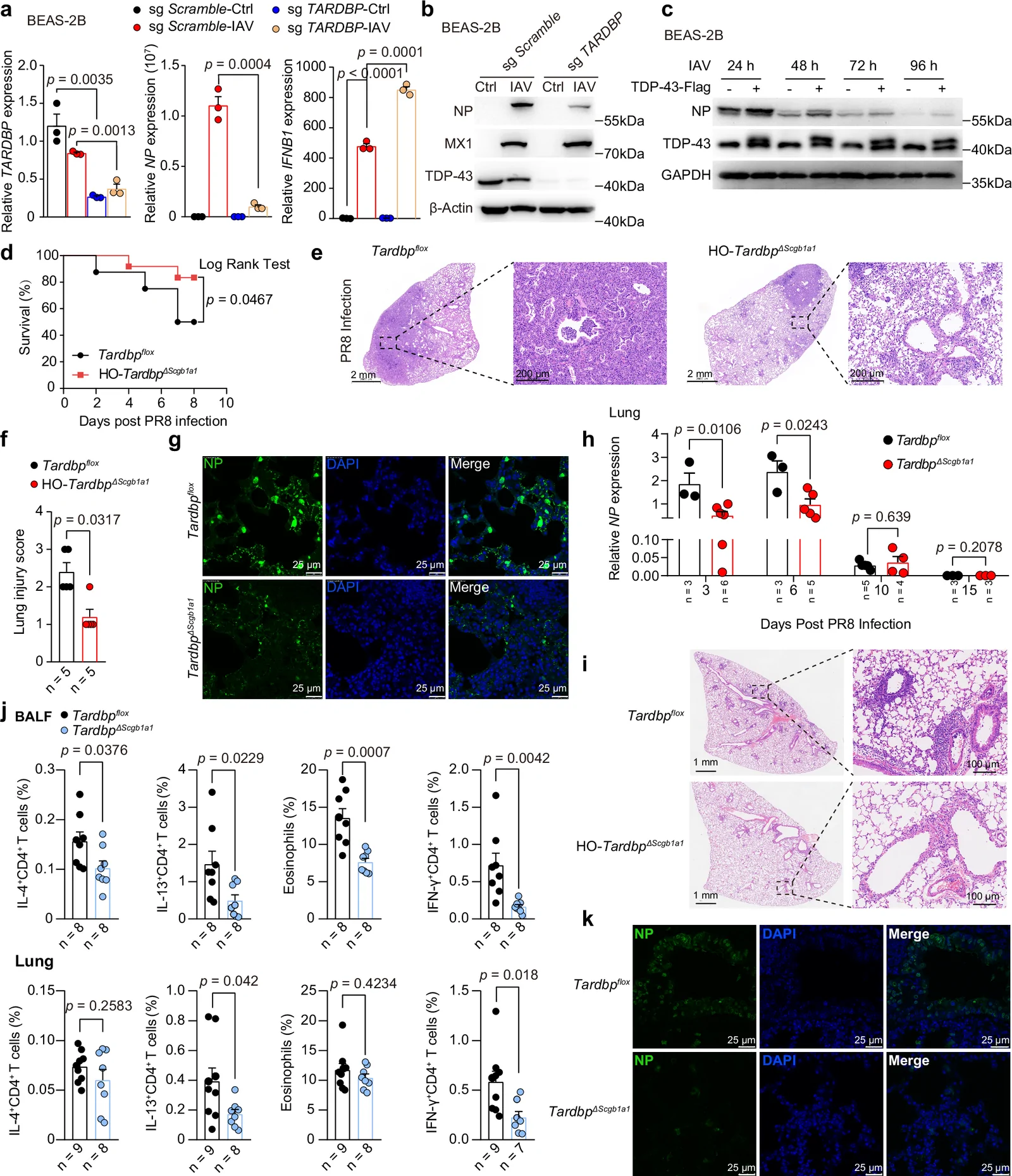
*Tardbp*^*ΔScgb1a1*^ mice are hypo-sensitive to IAV infection and IAV-AEs. **a** mRNA levels of *TARDBP*, *NP* and *IFNB1* in TDP-43-knockdown BEAS-2B cells with or without PR8 infection for 24 h. **b** Immunoblotting of NP, MX1 and TDP-43 in TDP-43-knockdown BEAS-2B cells with or without PR8 infection for 24 h. **c** Immunoblotting of NP and TDP-43 in TDP-43-overexpressing BEAS-2B cells infected with PR8 at different time. **d** Survival curves for *Tardbp*^*ΔScgb1a1*^ and *Tardbp*^*flox*^ mice post PR8 infection. **e** Representative H&E images comparing pulmonary pathologies between PR8-infected *Tardbp*^*ΔScgb1a1*^ and *Tardbp*^*flox*^ mice. **f** Bar plots displaying the quantified lung injury scores in (**e**). **g** Representative IF images comparing NP signals in the lung tissues between *Tardbp*^*ΔScgb1a1*^ and *Tardbp*^*flox*^ mice post PR8 infection. **h** qPCR results showing *NP* expression in lungs from *Tardbp*^*ΔScgb1a1*^ and *Tardbp*^*flox*^ mice at 3, 6, 10, and 15 days post-infection with PR8 virus. **i** H&E staining comparing pulmonary pathologies between *Tardbp*^*ΔScgb1a1*^ and *Tardbp*^*flox*^ mice under IAV-AE state. **j** Bar plots showing flow cytometric results of immune cell percentage in the lung tissues and BALF of *Tardbp*^*ΔScgb1a1*^ and *Tardbp*^*flox*^ mice with IAV-AEs. **k** Representative IF images comparing NP intensities in the lung tissues between *Tardbp*^*ΔScgb1a1*^ and *Tardbp*^*flox*^ mice with IAV-AEs. Data are presented as mean ± SEM. The data in **e**, **f**, **g**, **i**, **j**, **k** have at a minimum five independent biological replicates. Significance were assessed by unpaired two-tailed Student’s *t*-test (**a,** **h, j**), two-tailed Mann–Whitney *U* test (**f**, **j**), and Log Rank Test (**d**). Source data are provided as a Source data file.

Asthmatic individuals are susceptible to respiratory virus infections including IAV, causing exacerbations of existing asthma symptoms^45,46^. Wondering the potential roles of TDP-43 in AECs during respiratory virus infection, we infected the BEAS-2B-sg*TARDBP* line with IAV (PR8 strain). Results of qPCR revealed that upon IAV infection, BEAS-2B-sg*TARDBP* had significantly reduced expression of transcripts encoding viral *Nucleoprotein* (*NP*) but increased *IFNB1* expression compared to BEAS-2B-sg*Scramble* (Fig. 6a). Immunoblotting results confirmed that the expression of NP in BEAS-2B-sg*TARDBP* was remarkably reduced, while the expression of MX1 remained unchanged compared to BEAS-2B-sg*Scramble* (Fig. 6b). We then evaluated virus replication dynamics by infecting TDP-43-overexpressing and control BEAS-2B cells with PR8 and collecting samples at 24, 48, 72 and 96 hours post-infection. The results showed that TDP-43 overexpression significantly enhanced PR8 infection and delayed viral clearance (Fig. 6c and Supplementary Fig. 8a, b). These results indicate that TDP-43 facilitates IAV infection in AECs, in consistency with a recent study reporting that TDP-43 is recruited to the viral mRNAs by viral polymerase and facilitate IAV replication^47^. We further tested the susceptibility of *Tardbp*^*ΔScgb1a1*^ mice to IAV infection. HO-*Tardbp*^*ΔScgb1a1*^ mice displayed increased survival rate upon IAV infection when compared to *Tardbp*^*flox*^ mice (Fig. 6d). H&E staining showed that the HO-*Tardbp*^*ΔScgb1a1*^ mice exhibited significantly alleviated pulmonary pathologies following PR8 infection (Fig. 6e, f). Furthermore, lung tissues from *Tardbp*^*ΔScgb1a1*^ mice displayed substantially reduced viral NP signals (Fig. 6g). We also compared pulmonary NP signals between *Tardbp*^*ΔScgb1a1*^ and *Tardbp*^*flox*^ mice at 3, 6, 10, and 15 days post PR8 infection. In the lungs of *Tardbp*^*flox*^ mice, the NP signal increased from day 3 to day 6, peaked, then declined by day 10, with viral clearance achieved by day 15, whereas in the *Tardbp*^*ΔScgb1a1*^ mice, the lung viral NP levels remained substantially reduced at day 3 and day 6 (Fig. 6h and Supplementary Fig. 8c–e). These results collectively indicate that *Tardbp*^*ΔScgb1a1*^ mice were resistant to IAV infection.

Given the active involvements of TDP-43 in both allergen-induced airway inflammation and IAV infection, we proposed that TDP-43 could be a promising target for the treatment of IAV-AEs. We established a mouse IAV-AEs model by giving a single dose of RP8 infection on day 18 during the development of OVA-induced asthma (Supplementary Fig. 9a). The H&E results demonstrated that IAV infection aggravated pulmonary pathologies induced by OVA (Supplementary Fig. 9b). The flow cytometric results also revealed that OVA-induced asthmatic mice with IAV infection had increased pulmonary infiltrations of Th2 (IL-4^+^CD4^+^, IL-13^+^CD4^+^) and Th1 (IFN-γ^+^CD4^+^) in comparison with mice with OVA-stimulation alone (Supplementary Fig. 9c, d). Compared to the *Tardbp*^*flox*^ mice, the HO-*Tardbp*^*ΔScgb1a1*^ mice exhibited significantly diminished pulmonary pathologies and reduced infiltrations of Th2 (IL-4^+^CD4^+^, IL-13^+^CD4^+^), eosinophils, Th1 (IFN-γ^+^CD4^+^) and IFN-γ^+^CD8^+^ T cells (Fig. 6i, j and Supplementary Fig. 9e). IF staining of NP protein further demonstrated that TDP-43 ablation in AECs suppressed viral replication in lungs (Fig. 6k). These results indicate that TDP-43 is a promising therapeutic target for the treatment of IAV infection and IAV-AEs.

### I.n. application of ASO-LNP targeting TDP-43 ameliorates asthmatic response in mice

**Fig. 7:**
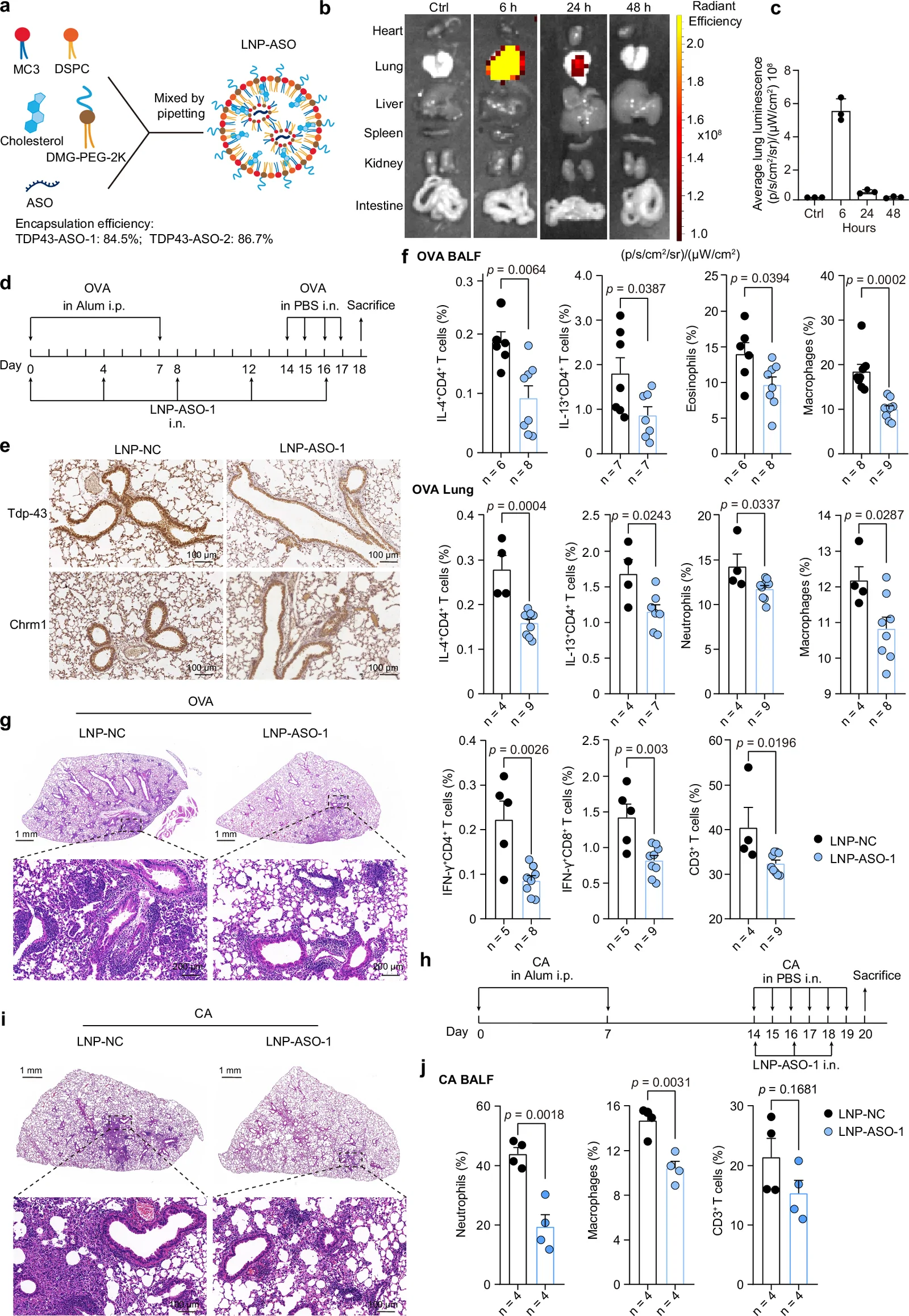
Therapeutic administering of LNP-ASO targeting TDP-43 in airway epithelium in asthmatic mice. **a** Scheme of the preparation of LNP-ASO and general lipid compositions of LNP-ASO formulation. **b** Ex vivo fluorescence images of the excised major organs at various timepoints after one-time i.n. administration of DiR-labeled LNP-ASO. **c** Bar plots showing mean fluorescence intensities of the excised lungs at different timepoints post DiR-labeled LNP-ASO administration. **d** Schematic diagram illustrating therapeutic strategy of LNP-ASO targeting Tdp-43 in OVA-induced asthmatic mice. **e** Representative images of IHC staining of Tdp-43 and Chrm1 in lung tissue from OVA-induced mice receiving i.n. LNP-ASO-1 intervention. **f** Bar plots showing flow cytometric results of immune cell percentage in the lung tissues and BALF of OVA-induced mice receiving i.n. LNP-ASO-1 intervention. **g** H&E staining of pulmonary pathologies in OVA-induced mice receiving i.n. LNP-ASO-1 intervention. **h** Schematic diagram illustrating therapeutic strategy of LNP-ASO targeting Tdp-43 in CA-induced asthmatic mice. **i** H&E staining of pulmonary pathologies in CA-induced mice receiving i.n. LNP-ASO-1 intervention. **j** Bar plots showing flow cytometric results of immune cell percentage in the BALF of CA-induced mice receiving i.n. LNP-ASO-1 intervention. Data are presented as mean ± SEM. The data in **e**,** g**,** i** have at a minimum five independent biological replicates. Significance was assessed by unpaired two-tailed Student’s *t*-test (**f**, **j**), and two-tailed Mann–Whitney *U* test (**f**). Source data are provided as a Source data file.

Lipid nanoparticles (LNPs), which can deliver therapeutic nucleic acids to specific organs, have gained increasing attention. Herein, to investigate the therapeutic potential of targeting TDP-43 in allergic asthma, we report an approach to boost i.n. ASO delivery through a modified standard four-component LNP formation^48^ (Fig. 7a). The encapsulation efficiencies of two distinct *Tardbp*-specific ASO sequences (TDP-43-ASO-1 and -2) resulting from pipetted mixing were 84.5% and 86.7%, respectively (Fig. 7a). Cryogenic transmission electron microscopy (cryo-TEM) imaging elucidated that the LNP-ASO had a dense spherical structure with a multi-lamellar shell and an amorphous core (Supplementary Fig. 10a). To examine biodistribution profiles, we conducted ex vivo organ fluorescence imaging on the excised major organs at various timepoints following i.n. administering of the indicated LNP formula carrying a fluorescent probe of DiR. Strong fluorescence signals were predominantly detected in the lung tissues within 24 h post-application (Fig. 7b, c), indicating that the LNP-ASO complexes traversed the airway mucosa. The other organs (heart, liver, spleen, and kidney) showed non-detectable fluorescence signals, indicating high lung-targeting specificity (Fig. 7b). H&E staining detected no pathological changes in multiple tissues following i.n. administering of LNP, implying that the i.n. of LNP was safe (Supplementary Fig. 10b). In vitro screening revealed that both LNP-ASO constructs effectively suppressed the expressions of Tdp-43 and Chrm1 in the mouse melanoma cell line B16-F10 (Supplementary Fig. 10c–e). LNP-ASO-1 with superior efficacy in suppressing Tdp-43 expression was chosen for subsequent in vivo therapeutic evaluations in allergen-induced murine asthma model. The therapeutic study was conducted in two distinct modes: a prophylactic regimen, in which LNP-ASO was administered throughout both the allergen sensitization and challenge phases (Fig. 7d–f) and a therapeutic regimen, in which LNP-ASO was initiated after two weeks of sensitization (Fig. 7h–j). This design allowed us to evaluate the protective effect prior to disease establishment and the therapeutic effect on established allergen-sensitized animals. IHC result exhibited that i.n. delivery of LNP-ASO-1 substantially intervened Tdp-43 expression in the airway epithelium, accompanied by a significant downregulation of Chrm1 (Fig. 7e). Moreover, the results of lung pathology and flow cytometry proved that LNP-ASO-1 administration in both prophylactic and therapeutic manner significantly ameliorated asthma-associated inflammations and pathologies (Fig. 7f, g, i, j). Collectively, our findings indicate that i.n. administering of LNP-ASO targeting TDP-43 represent a promising therapeutic approach for asthma.

## Discussion

Current pipelines of targeted therapies for asthma predominantly focus on T2 immune response. This approach provides symptomatic management without addressing the underlying pathophysiology, resulting in persistent recurrence. AECs harbor critical factors that mediate airway epithelial injuries induced by host allergen exposure, thereby driving the asthmatic response. In this study, we discovered that TDP-43 upregulation in AECs drives allergen-induced asthmatic airway inflammation through dampening UPF1-mediated NMD activity in asthmatic AECs, leading to accumulated expression of pathogenic NMD-sensitive transcripts, such as *CHRM1* that transduces signaling of the vagal neurotransmitter ACh. We also demonstrated that targeting TDP-43 in AECs is a potential strategy for treating asthma and IAV-AEs.

### TDP-43 may act as a stress sensor in AECs upon host allergen exposure

It becomes increasingly recognized that allergen exposure induces airway epithelium perturbations^6^. These can arise directly from the protease activity of allergens such as CA or HDM, or indirectly from Th2 immunity primed by allergens like OVA, which lacks protease activity and does not directly damage the epithelium. OVA disease models remain useful for studying airway barrier dysfunction, as OVA could indirectly disrupt the barrier via Th2 immunity^7,9^, facilitating further allergen penetration. Barrier abnormalities release epithelial alarmins, which fully activate T2 effector responses. The resulting T2 cytokines further impair the barrier, creating a vicious cycle^6,49^. Thus, allergen‑induced airway tissue disturbance is not confined to the early phase but persists throughout repeated allergen exposure. The persistent airway epithelium perturbations activate chronic cellular stress in AECs. This raises a key unanswered question: how tissue perturbation-induced cellular stress is sensed and converted into potent asthmatic responses. In this study, we identify TDP-43 as a key sensor for this cellular stress. Firstly, the nuclear distribution of TDP-43 may be disrupted by cellular stress, leading to the abnormal formation of its RNA-depleted nuclear condensates or cytoplasmic aggregation as reported in neuron cells^26,27^. Normally, TDP-43 maintains homeostasis by binding to its own 3’UTR to autoregulate its protein levels, however, under the circumstances of neurodegenerative disorders, nuclear mislocalization of TDP-43 breakdowns this autoregulation loop, leading to uncontrolled accumulation of TDP-43^22^. It shall be similar in asthmatic airway epithelium, wherein TDP-43 expression level is significantly increased. Secondly, dysregulated TDP-43 level further suppresses UPF1 expression by directly destabilizing *UPF1* transcripts, leading to dampened NMD activity and subsequent accumulation of NMD-sensitive transcripts, which encode pathogenic molecules, such as ATF4 and CHRM1. The final step is the execution of the type 2 innate inflammatory response: ATF4 initiates a transcriptional program that facilitates IL-33 maturation and release^14,50^, and CHRM1 transduces ACh signaling.

### TDP-43 restrains NMD activity by regulating alternative splicing and stability of UPF1 transcripts

In neuron cells with TDP-43 loss, NMD is activated to degrade RNAs containing PTCs introduced by aberrant cryptic exons^51^. These cryptic exons become fully detectable only when NMD is inhibited^52^. This indicates that UPF1 expression or activity is not reduced, and may even be enhanced, by TDP-43 loss. Meanwhile, wildtype TDP-43 overexpression in rodent neurons impair UPF1-mediated NMD and leads to cytotoxicity, which can be rescued by co-overexpression of UPF1^28,29^. In consistency, we also observed that TDP-43 overexpression suppresses UPF1 expression, and *UPF1* transcripts are destabilized in AECs with allergen exposure. To date, the underlying mechanism by which TDP-43 suppresses UPF1 expression is unknown. In our study, we revealed that TDP-43 directly binds to *UPF1* transcripts and regulates its alternative splicing. It can be speculated that under physiological conditions, AECs with proper expression level and correct nuclear distribution of TDP-43 may be recruited to the (UG)_4_ site and prevent the inclusion of the so-called 33-nt cryptic exon, thereby generating *UPF1*_*SL*_. In contrast, in allergen-induced AECs, dysregulated nuclear distribution and expression of TDP-43 might disrupt this splicing regulation, leading to the generation of *UPF1*_*LL*_. The regulatory mechanism between TDP-43 and *UPF1* in asthmatic airway response may also be relevant to the neuropathological field.

### CHRM1 transcripts are NMD-sensitive and accumulate in asthmatic AECs

The increased release of the neurotransmitter ACh acts on various type of muscarinic receptors expressed on ASMCs, airway neurons, immune cells, and submucosal glands to potentiate airway inflammation, bronchoconstriction, and mucus secretion in asthma^53^. CHRM1 plays significant roles in the pathophysiology of asthma by mediating bronchoconstriction^54–56^, modulating neural responses, enhancing inflammatory processes, and increasing mucus secretion. Polymorphisms in the CHRM1 confer susceptibility to asthmatic populations^35^. The selective CHRM1 antagonist pirenzepine suppressed vagally induced bronchoconstriction in human^56^. Here we uncovered that *CHRM1* is one of NMD targets that accumulate in asthmatic AECs with compromised NMD activity, advancing our understanding of the critical role of CHRM1 expression regulation in asthmatic response.

### TDP-43 plays pathogenic roles in both asthmatic airway response and respiratory viral infection

The complex interplay between respiratory viral infections and the asthma pathogenesis remains an area of active investigation. While the hygiene hypothesis posits that early-life microbial exposure shapes host immune system and may reduce allergy/asthma risk^57^. However, established asthmatic airway epithelium exhibits disrupted tight junctions that facilitate viral entry^58^, meanwhile, virus infection triggers IL-33 release, amplifying T2 inflammation and inhibiting type I/III interferon (IFN) production, thereby blunting antiviral immunity^59,60^. Substantial evidence confirms that respiratory viruses, including rhinovirus and IAV, drive acute asthma exacerbations, imposing significant clinical burdens during epidemics^5^. Asthmatic children infected with H1N1 experienced heightened symptom severity^45,61^. Consequently, IAV-complicated asthma frequently necessitates intensified clinical management due to exacerbated pathological manifestations^62^. While influenza vaccination provides seroprotection in asthma^63^, targeted therapies for IAV-AEs remain critically deficient. Here, we demonstrated that targeting TDP-43 in the airway epithelium could not only block asthmatic airway response but also restrain IAV replication, offering a promising therapeutic target for IAV-AEs. The ongoing drug development for the treatment of TDP-43 mutation-associated neurodegenerative diseases may be repurposed to treat asthma, IAV infection, and IAV-AEs.

In summary, we show that allergen-induced TDP-43 expression in AECs dampens UPF1-mediated NMD activity, leading to accumulation of NMD-sensitive transcripts encoding pathogenic molecules that mediating asthmatic response. We also provide preclinical therapeutic evidence demonstrating that LNP-ASO targeting TDP-43 in AECs is safe and efficacious for the treatment of asthma.

## Methods

### Human samples

Human lung biopsies were obtained from the pathology department of Guangzhou Women and Children’s Medical Center, State Key Laboratory of Respiratory Disease, Guangzhou Medical University under the approval of Ethics Review Committee (Approval no: 2023038B00). The lung tissue was obtained from either non-malignant adjacent tissue in lung cancer surgical specimens or from organ donors, sourced from individuals with or without ACOS. The bronchoscopic mucosal biopsy tissues or bronchial epithelial cell brushing specimens were collected from Ningde Municipal Hospital under the approval of Ethics Review Committee (Approval no: NSYKYLL-2025-152). The bronchoscopic mucosal biopsy tissues or bronchial epithelial cell brushing specimens were harvested from asthmatic patients, patients with COPD, or simple pneumonia undergoing bronchoscopic brushing as part of their diagnostic evaluation. All participants gave written, informed consent. Further use and additional analyses were permitted and consented. The gender, age, and peripheral blood eosinophil count of each individual were provided in Supplementary Table 7.

### Mouse models

*Tardbp*^*flox*^ mice (NM-CKO-2102528) and Scgb1a1-IRES-Cre mice (NM-KI-210120) were purchased from Shanghai Model Organisms Center, Inc., and airway epithelium specific *Tardbp* deficient (*Tardbp*^*ΔScgb1a1*^) mice were generated by breeding Scgb1a1-IRES-Cre mice with *Tardbp*^*flox*^ mice. Male C57BL/6J mice (6–8 weeks old) were obtained from Rise Mice Biotechnology Co., Ltd for the development of an asthmatic mouse model. The transgenic mouse strains used in this study were propagated and maintained by Cyagen Biosciences (Suzhou) Inc. All animal experiments were approved by the Animal Care and Use Committee of Shanghai Yishang Biotechnology Co., Ltd with Biosafety Level 2 (BSL-2) facilities (Approval no. IACUC-2023-Mi-209).

For construction of asthmatic mouse models, mice were intraperitoneally injected with 10–50 µg OVA, HDM, or CA dissolved in 100 μl PBS, emulsified with 100 µl adjuvant on days 0 and 7. During the challenge phase, mice were intranasally administered 100 μg OVA, HDM, and CA in 50 μl of PBS on days 14, 15, 16, and 17^64^. For the low-concentration CA-induced asthma model, mice were sensitized by intraperitoneally injecting of 5 µg CA in 100 µl CFA on day 0 and day 7. Mice were challenged with CA (5 µg in 50 µl PBS) i.n. from days 14 to 17^65^. In all experiments, PBS served as the control. Mice were euthanized 24 h after the last allergen challenge, and peripheral blood, bronchoalveolar lavage fluid (BALF), and lung tissues were collected for subsequent analysis.

For the Influenza A (PR8, H1N1) infection mouse models, the virus was originally procured from Dr. Hui Zhang (Zhongshan Medical School, Sun Yat-sen University). To assess survival rates, the *Tardbp*^*ΔScgb1a1*^ mice and the littermate controls were intranasally injected with the median lethal dose (LD_50_) of PR8 virus. After infection, the mortality of mice was continuously monitored. On day 8, the mice were euthanized, and lung tissues were collected for histological and immunofluorescence staining to assess viral infection and lymphocyte infiltration. For the allergen-induced asthma and IAV infection mouse model, mice were first subjected to OVA sensitization, followed by infecting with PR8 virus. After 24 h, the lung tissue and BALF of mice were collected for further analysis.

For AAV-mediated airway epithelial cell-specific Chrm1-overexpressing mouse model, we constructed the AAV-Chrm1 overexpression virus with Cc10 promoter-driven expression. The *Tardbp*^*ΔScgb1a1*^ mice and the littermate controls were intranasally administered with AAV9-Cc10-Chrm1 or AAV9-Cc10-Cre at a dose of 6 × 10^10^ vector genomes (vg) per mouse. Three weeks later, mice were subjected to OVA/CA-induced asthma modeling. The Mouse Scgb1a1 promoter and Chrm1 CDS for the generation of AAV9-Chrm1 were listed in Supplementary Table 6.

To generate Tardbp-knockdown mice via the LNP-ASO method, C57BL/6J mice received intranasal injections of ASO-LNPs at a dose of 0.4 nmol per administration, concurrent with the OVA and CA modeling procedure. Both male and female participants were included in this study without distinction, and data were analyzed without stratification by sex. The mice were euthanized by narcotic drug overdose in accordance with the recommendations for the euthanasia of experimental animals.

### Cell culture

BEAS-2B and 293T cell lines were purchased from ATCC (American Type Culture Collection), and routinely maintained in DMEM (Gibco) supplemented with 10% FBS (Gibco) and penicillin/streptomycin (Gibco). Cells were kept in a humidified cell culture incubator at 37 °C with 5% CO_2_ atmosphere. BEAS-2B cells were stimulated with LPS for indicated time, or infected with Influenza A for 24 h. Subsequently, the cells were harvested for qPCR analysis. ASO-LNPs were diluted in culture media to a total ASO dose of 50 nmol for in vitro knockdown of Tdp-43. BEAS-2B cells were infected with PR8 and then collected at 24 h, 48 h, 72 h, and 96 h post-infection for subsequent analyses.

Mouse bronchial epithelial cells were isolated as described previously^66^. Briefly, the trachea and hearts of mice were rinsed with PBS. The bronchi were carefully dissected as far as possible, cut into small pieces, and subsequently digested in DMEM/F12 medium containing antibiotics (penicillin-streptomycin- amphotericin B) and 0.15% pronase overnight at 4 °C. The digested tissue was terminated with DMEM/F12 supplemented with 20% FBS, and centrifuged at 500 × *g* for 10 min at 4 °C. Then, the precipitates were further digested with 1 ml of DNase I solution (consisting of 900 µl DMEM/F12 medium, 100 µl of 10 mg/ml BSA, and 0.5 mg DNase I) for 5 min, followed by centrifuging at 500 × *g* for 10 min at 4 °C. Red blood cells were lysed by incubating with 1 ml ACK for 2 min, and the lysis reaction was terminated by adding 10 ml of pre-cooled PBS. Subsequently, we used CD45 microbeads (130-052-301, Miltenyi) to removed CD45 positive cells and EpCAM microbeads (130-105-958, Miltenyi)) to collect EpCAM positive cells. Finally, the dead cells were removed with the Dead Cell Removal Kit (130-090-101, Miltenyi).

Primary Human Bronchial Epithelial cells (HBECs) were grown in PneumaCult-Ex Plus Medium (05040) and placed in 6 cm dish for maximum 10 days, or until 80–90% confluency.

The mouse bronchial epithelial cells and the primary Human Bronchial Epithelial cells (HBECs) were isolated from both sexes.

### Air–liquid interface (ALI) culture

The isolated mouse bronchial epithelial cells were resuspended with 8 mL MTEC medium containing 10% FBS, and plated on 10 cm dish to incubate for 5 h. The cell suspension was collected and centrifuged. The resulting pellet was resuspended in PneumaCult-Ex Plus Medium (05040) and seeded on transwell plates (0.4 μm pore size, 3470) until reaching apparent confluence. For HBECs, cells were trypsinized, resuspended in PneumaCult-Ex Plus Medium (05040), and seeded on transwell plates (0.4 μm pore size, 3470) until reaching apparent confluence.

Following this, the upper chamber PneumaCult-Ex Plus Medium was removed, and the cells were induced to differentiate by adding PneumaCult-ALI Medium to the lower chamber, with medium refreshed every two days for more than 14 days. On day 7 of ALI culture, cells were treated with BSA or 10 ng/mL Recombinant Murine Il13 cytokines (210-13-10 μg) for 7 days. For CA stimulation, 200 μg/ml CA was applied to the upper chamber for 24 h. For OVA stimulation, 400 μg/ml OVA was applied to the upper chamber for the same duration. The cells were then subjected to RT-PCR, western blot analysis, fixation, embedding, and further stained with H&E, PAS, and immunofluorescence staining.

### TARDBP and UPF1 stable knockdown cell line construction

The sgRNA sequences of TDP-43 and UPF1 were cloned into the LentiCRISPR V2 plasmids. To acquire the lentivirus, the plasmids were co-transfected with lentiviral package vectors (psPAX and pMD2.G) into 293T cells. At 48 h post-transfection, virus-containing supernatant of 293T cells was harvested and centrifugated at 20,000 × *g* for 2 h. After removing the supernatant, the precipitate containing lentivirus particles was resuspended with DMEM.

To establish the stable TDP-43-knockdown and UPF1-knockdown cell line, the packaged lentivirus was added to the culture medium of BEAS-2B cells and incubated for 8 h. At 24 h post-infection, the cells were selected with puromycin for 3 days or longer. The sgRNA sequences of TDP-43 and UPF1 were listed as following:

*TARDBP* Forward sequence: 5′-CACCGACATCCGATTTAATAGTGTT-3′;

*TARDBP* Reverse sequence: 5′-AAACAACACTATTAAATCGGATGTC-3′;

*UPF1* Forward sequence: 5′-CACCGCAAAGAGGTGACCCTGCACA-3′;

*UPF1* Reverse sequence: 5′-AAACTGTGCAGGGTCACCTCTTTGC-3′.

### RNA sequencing

Total RNA was isolated using the Trizol Reagent (Invitrogen Life Technologies), after which the concentration, quality, and integrity were determined using a NanoDrop spectrophotometer (Thermo Scientific). Three microgram RNA was used as input material for the RNA sample preparations. Sequencing libraries were generated according to the following steps. Firstly, mRNA was purified from total RNA using poly-T oligo-attached magnetic beads. Fragmentation was carried out using divalent cations under elevated temperature in an Illumina proprietary fragmentation buffer. First strand cDNA was synthesized using random oligonucleotides and Super Script II. Second strand cDNA synthesis was subsequently performed using DNA Polymerase I and RNase H. Remaining overhangs were converted into blunt ends via exonuclease/polymerase activities, and the enzymes were removed. After adenylation of the 3’ ends of the DNA fragments, Illumina PE adapter oligonucleotides were ligated to prepare for hybridization. To select cDNA fragments of the preferred 400–500 bp in length, the library fragments were purified using the AMPure XP system (Beckman Coulter, Beverly, CA, USA). DNA fragments with ligated adaptor molecules on both ends were selectively enriched using Illumina PCR Primer Cocktail in a 15 cycle PCR reaction. Products were purified (AMPure XP system) and quantified using the Agilent high sensitivity DNA assay on a Bioanalyzer 2100 system (Agilent). The sequencing library was then sequenced on NovaSeq 6000 platform (Illumina).

### RIP-QPCR

Fifty million BEAS-2B cells were washed twice with cold PBS. Cells were treated with 0.5% formaldehyde for 10 min, and the reaction was quenched with 125 mM glycine. The cells were then washed twice with cold PBS and subsequently lysed with Lysis buffer containing proteinase Inhibitor and 400 U/ml murine RNase Inhibitor (RL301-02). Samples were treated by sonication and centrifuged at 14,000 × *g* for 15 min. A portion of the cell lysate was set aside as the input, while the remainder was incubated overnight at 4 °C with anti-TDP-43 (10782-2-AP, 1:100 dilution) or Rabbit IgG. On the following day, pre-washed Protein A/G Magnetic beads were added and incubated at 4 °C for 3 h. Subsequently, the beads were washed five times with ice-cold washing buffer containing 200 U/ml RNase inhibitor. Afterward, the magnetic beads were digested with Proteinase K in washing buffer containing 1% SDS, 2 mg/mL Proteinase K, and 400 U/mL RNase inhibitor, and incubated at 55 °C for 1 h with continuous shaking. Finally, total RNA was purified from both the input and eluted samples using TRIzol reagent (5 volumes) followed by the RNA extraction step. The sequences of the two primer pairs targeting intron 6 and the 3′UTR were as follows:

intron 6 Forword sequence: 5′-TGCGGTGAAGCCTCCAGAC-3′;

intron 6 Reverse sequence: 5′-AGCCTCTGCCGTGAGAGC-3′;

3’UTR Forward sequence: 5′- CCACTGCTAAAACTGAAGCACC-3′;

3’UTR Reverse sequence: 5′-GCTACTCTGAGATGAGGGTCTTCC-3′.

### Actinomycin D assay

BEAS-2B cells and ALI differentiated AECs were treated with 10 μg/ml Actinomycin D (HY-17559-5 mg) for 0, 1, 2, 4, 6, or 8 h to block transcription. mRNA degradation was determined by RT-PCR.

### Luciferase assay

The 5’UTR sequence of human or mouse CHRM1 was synthesized and cloned into the pmirGLO vector by a commercial vendor. The start codons of uORFs 11, 20 and 21 in the human CHRM1 5’UTR pmirGLO vector were mutated to GGG using a site-directed mutagenesis kit. The Dual-Luciferase Reporter Assay was performed using the Dual-Luciferase Reporter Assay System (E1960, Promega) according to the manufacturer’s protocol. Briefly, BEAS-2B or 293T cells were seeded in 96-well plates. The next day, the cells were co-transfected with the CHRM1 5′UTR pmirGLO vectors and either TDP-43-overexpression or control plasmid. Twenty-four hours post-transfection, cells were lysed with 20 μl of lysis buffer for 15 min. Subsequently, 5 μl of the lysate was transferred to black 96-well microplate. 50 μl of the prepared Luciferase Assay Buffer Ⅱ was added, and the firefly luciferase activity was determined. Finally, 50 μl of the prepared Stop & Glo reaction solution was added, and the activity was measured again.

### Polymerase chain reaction

Total RNA was extracted using TRIzol reagent (R401-01, Vazyme). Reverse transcription was performed from RNA to cDNA with HiScript III RT SuperMix (R323-01, Vazyme), and the real-time PCR was operated with ChamQ Universal SYBR qPCR Master Mix (Q711-02, Vazyme) according to the manufacturer’s instructions. Data were collected and analyzed using a QuantStudio 7 Flex. The primer sequences were listed in Supplementary Table 8.

For analysis the splicing of CHRM1, total RNA was extracted and reverse-transcribed into cDNA from BEAS-2B or primary mouse bronchial epithelial cells, and the PCR was operated with a commercial PCR kit (SB-111, share-bio). The primer sequences were also listed in Supplementary Table 8.

### Western blot analysis

Western and IP cell lysis buffer (Beyotime) with proteinase and phosphatase inhibitor cocktail was used to lyse cells. Proteins were separated by SDS-PAGE gel and then transferred to PVDF membranes (Merck Millipore). After blocking, the membranes were incubated at 4 °C overnight with anti-TDP-43 (1:2000 dilution), anti-UPF1 (1:3000 dilution), anti-ATF4 (1:2000 dilution), anti-GAPDH (1:5000 dilution), anti-CHRM1 (1:1000 dilution), followed by incubating with secondary antibody. Immunoreactive bands were visualized using an Gel imaging analysis system. Primary antibody sources and working dilutions were listed in Supplementary Table 9.

### Pulmonary histopathologies

The left lung sections of mice were fixed in 4% paraformaldehyde for 24 h, paraffin-embedded, and sectioned into 5-μm-thick slices. Subsequently, the tissue sections were stained with hematoxylin and eosin (H&E), and photographed by light microscopy to evaluate the degree of airway inflammation.

Histologic lung injury severity was scored per lung lobe according to a standardized scoring system by evaluating the extension of pulmonary leukocyte infiltration, the amount of intra-alveolar leukocytes, and the amount of exudative debris and edema fluid: 0 = 0%, no lesions; 1 = 1%–25%, minimal; 2 = 26–50%, mild; 3 = 51–75%, marked; 4 = 76–100%, severe^67^.

For Tdp-43 and Chrm1 immunohistochemical staining, the sections were deparaffinized and subjected to antigen retrieval in 10 mM citrate buffer (pH = 6.0) with microwave heating. Next, the sections were blocked with serum for 30 min at room temperature and incubated overnight with antibodies against Tdp-43 (10782-2-AP, 1:400 dilution) or Chrm1 (abs121129, 1:200 dilution) in 5% BSA/PBS at 4 °C. On the following day, the sections were incubated with a biotinylated anti-rabbit IgG secondary antibody (1:200 dilution) for 1 h at room temperature. Subsequently, immunoreactivity was visualized using a DAB peroxidase substrate kit, followed by counterstaining with hematoxylin.

### Immunofluorescence (IF) staining

The paraffin-embedded lung sections were deparaffinized using dimethylbenzene. Multiplex immunofluorescence staining was conducted with the PANOVUE system for primary antibodies of identical host origin according to the manufacturer’s instructions. Briefly, the deparaffinated sections were immersed in sodium citrate buffer (10 mM citrate, pH 6.0) and boiled for 15 min for antigen retrieval. Subsequently, the sections were sequentially processed with 10% goat serum blocking for 30 min at room temperature, primary antibody incubating overnight at 4 °C, and the anti-rabbit IgG, HRP-linked antibody treatment for 1.5 h at room temperature, followed by staining with the tyramide signal amplification (TSA). All other primary antibodies staining was performed as the same procedure. The TissueFAXS platform (TissueGnostics) and Leica TCS SP8 were used to acquire multispectral images of the whole section at different magnification, and the fluorescent images were analyzed using Leica Application Suite X software. The TissueQuest software and Image J was utilized to analyze mean fluorescence intensity for each slide.

### Mouse lung tissue digestion and immune cell profiling with flowcytometry

The lung tissues of mice were cut with scissors and transferred to 5 ml centrifuge tube, addition with 0.5 mg/ml collagenase A to digest in a shaking table for 1 h at 37 °C. The digested tissue was filtered by a 70-μm screen and centrifuged at 500 × *g* for 5 min at 4 °C. 2 ml ACK was added to the cells in the bottom of the EP tube to crack the red blood cells for 5 min, followed by addition of 10 ml PBS to terminate the cleavage. After centrifugation again, the supernatant was discarded to obtain cell precipitation for use.

For BALF and lung single-cell suspension myeloid flow panel, cells were incubated with the flow antibodies to anti-mouse CD45 (103128, BioLegend), CD3 (100241, BioLegend), CD4 (100410, BioLegend), SiglecF (155504, BioLegend), CD11b (101228, BioLegend), F4/80 (123116, BioLegend), Ly6G (108441, BioLegend), CD19 (115530, BioLegend), CD11c (117318, BioLegend) and I-A/I-E (107639, BioLegend) for 30 min at 4 °C for surface staining.

For Th2 flow panel, the lung single-cell suspension was stimulated with PMA (50 ng/ml), ionomycin (1 μg/ml), and BFA (1x dilution) in 500–1000 μl complete 1640 medium (Gibco) supplemented with 10% FBS and penicillin/streptomycin and incubated at 37 °C for 4 h prior to staining. Then the cells were incubated with the flow antibodies to anti-mouse CD45 (103128, BioLegend), CD3 (100241, BioLegend), CD4 (100410, BioLegend), CD8 (100749, BioLegend) for 30 min at 4 °C for surface staining. Subsequently, dead cells were stained with Zombie Aqua Fixable Viability Kit (423101, BioLegend) in all flow panels. For intracellular staining, the cells were treated with Cyto-Fast Fix/Perm Buffer Set (426803, BioLegend) and stained with anti-mouse IL-4 (504126, BioLegend), IL-5 (504306, BioLegend), IL-13 (159403, BioLegend), GATA3 (566642, BD), IFN-γ (505829, BioLegend).

Flow cytometry was performed using a LSR Fortessa flow cytometer (BD). The data were analyzed with FlowJo 10.0 (Treestar). Different cell types were identified by the following gating strategies: macrophages (Zombie Aqua^-^CD45^+^F4/80^+^CD11c^+^), B cells (Zombie Aqua^-^CD45^+^CD19^+^), T cells (Zombie Aqua^-^CD45^+^CD3^+^), Th2 (Zombie Aqua^-^CD45^+^CD3^+^CD4^+^GATA3^+^), eosinophils (Zombie Aqua^-^CD45^+^CD11c^-^SiglecF^+^), neutrophils (Zombie Aqua^-^CD45^+^CD11b^+^Ly6G^+^) and dendritic cells (Zombie Aqua^-^CD45^+^F4/80^-^CD11c^+^I-A/I-E^+^) in the BALF and lung tissue cells.

### Single cell RNA sequencing

*Tardbp*^*ΔScgb1a1*^ and *Tardbp*^*flox*^ mice were subjected to OVA modeling. After modeling, the trachea and half of the lung tissue adjacent to the trachea were collected from the mice, then minced with scissors and digested in 0.5 mg/mL collagenase A in a shaking table for 1 h at 37 °C. Then, the digested tissue was filtered through a 70 μm cell strainer and centrifuged at 500 × *g* for 5 min at 4 °C. Red blood cells were lysed for 2 min with 2 ml ACK buffer, quenched with 10 ml PBS, and pelleted by centrifugation. The cell pellet was resuspended in 1640 medium supplemented with 10% FBS and 5 μM EDTA. CD45 positive immune cells were then partially removed using CD45 MicroBeads (130-052-301, Miltenyi) to obtain epithelial-enriched single-cell suspension for downstream applications.

Single-cell RNA sequencing was performed using the SURFSeq 5000 platform (GeneMind Biosciences LTD., China). Freshly isolated single-cell suspensions were loaded onto the SURFSeq 5000 microfluidic chip, where individual cells were encapsulated into nanoliter-scale droplets together with barcoded beads containing unique molecular identifiers (UMIs) and cell barcodes. Reverse transcription, cDNA amplification, and library construction were carried out following the manufacturer’s standard protocol. Sequencing reads were evaluated by FastQC and MultiQC. High-quality reads were aligned to the GRCm39-2024-A genome assembly and quantified using CellRanger (version 5.0.1, 10X Genomics). The gene expression matrix was analyzed in R using the Seurat package (4.3.0). Cell clusters were annotated based on canonical marker genes as defined in Supplementary Table 2.

Processed count matrixes of AECs on airway mucosa from allergic asthmatic individuals under exposure of allergen or diluent (GSE193816) were downloaded and analyzed with Seurat (v4.0.1)^68^. The ciliated cell subpopulations were annotated by the expressions of canonical marker genes (*FOXJ1* and *PIFO*). RQC signature score was calculated with the *AddModuleScore* function in Seurat. The genes used for RQC signature score determination are listed in Supplementary Table 1.

### Smart-seq2 analysis

Total RNA was extracted from isolated primary bronchial epithelial cells for library construction and quality inspection. The sequencing library was then sequenced on NovaSeq 6000 platform (Illumina). Reference genome and gene model annotation files were downloaded from the Ensemble database (v.108.39, mouse: GRCm39; human: GRCh38). The index of the reference genome was constructed using HISAT2 v2.0.5, and the paired end clean reads were compared with the reference genome using HISAT2 v2.0.5. HTSeq (0.9.1) was used to statistically compare the Read Count value of each gene as the original gene expression. In order to make the gene expression levels of different genes and different samples comparable, FPKM (Fragments Per Kilobase per Million fragments) was used to standardize the expression levels. For pair-end sequencing, there are two Reads per fragment. FPKM only counts the number of Fragments from two Reads that can be compared to the same transcript. Analysis of Alternative Splicing (AS) events were performed with rMATS (http://rnaseq-mats.sourceforge.net/index/html). Quantification of splicing event was based on the junction counts, which were calculated as $\phi=\left(\frac{I}{{{\rm{LI}}}}\right)/\left(\frac{I}{{{\rm{LI}}}}+\frac{S}{{{\rm{LS}}}}\right)$, where:

ϕ:exon inclusion level;

*I*: exon inclusion isoform;

LI: effective length of isoform with exon inclusion;

*S*: exon skipping isoform;

LS: effective length of isoform with exon skipped.

AS events with $\Delta \phi=\left|\phi 1-\phi 2\right|$ > 5% and FDR < = 1% were considered as statistically significant.

### GSEA

GSEA analysis was performed to functionally annotate the relevant genes and assessed the enriched signaling pathways using GSEA_Linux_4.1.0. Absolute NES > 1, *p* < 0.05 and FDR < 0.25 was recognized as a statistically significant pathway.

### minigene splicing

A splicing reporter construct was generated using the pSpliceExpress vector^37^, which contains two constitutively spliced rat insulin exons flanking a multiple cloning site, driven by an RSV LTR promoter.

The genomic fragment encompassing intron6 (286 bp), exon7 (118 bp), and intron7 (147 bp) of human UPF1 (GRCh38/hg38 coordinates: chr19: [18852701]-[18853251]) was amplified from human genomic DNA by PCR using primers fused with attB1 and attB2 recombination sites. The purified PCR product was recombined directly into the pSpliceExpress vector, and the vector was verified by Sanger sequencing. The fragment sequences were listed in Supplementary Table 10.

TDP-43-overexpressed BEAS-2B cells and control cells were seeded into 6-well plates, and transfected at 70% confluence with 1 μg of minigene plasmid per well using Lipofectamine 3000 Transfection Reagent (L3000015, Thermo Fisher Scientific). Total RNA was isolated 24 h post-transfection using the TRIzol reagent (R401-01, Vazyme). Splicing patterns of the inserted UPF1 genomic segment were analyzed by RT-PCR using primers specific to the upstream and downstream insulin exons (forward: 5′-GATCGATCCGCTTCCTGCCCC-3′; reverse: 5′-CTGCCGGGCCACCTCCAGTGCC-3′), and PCR products were resolved on 2.5% agarose gels. The band intensities of *UPF1*_*SL*_ and *UPF1*_*LL*_ were quantified using ImageJ software.

### Preparation of LNPs

The ASO sequences are Tdp-43-ASO-1 5′-TTTCAATGGGTTCATCGTTC-3′ and Tdp-43-ASO-2 5′-TACACTGAGACACGGGATTC-3′. All lipids with specified molar ratios were dissolved in ethanol, and ASO was dissolved in citric acid/sodium citrate buffer (20 mM, pH 4.0). The base four-component LNP formulation herein consists of MC3, DSPC, cholesterol, and DMG-PEG-2K at the molar ratio of 50/10/38.5/1.5, and ASO (total lipids/ASO = 40/1, w/w). The prepared lipid mixtures were rapidly mixed by pipette with the ASO aqueous phase at a volume ratio of 1:3, allowing for self-assembly of ASO-loaded LNPs. Before mixing, lipid solution was warmed at 60 °C for one minute to ensure homogenous solubilization of lipid components. Upon mixing, LNPs were immediately diluted in PBS to bring the ethanol concentration below 5% (v/v), and then concentrated through ultracentrifugation using an Amicon filter with the MWCO of 30 kDa (Millipore, MA, USA) and buffer exchange to PBS. To formulate DiR-labeled LNP-ASO, we mixed the LNP-ASO and DiR in a 100:1 (w/w) ratio. After pipette mixing and incubation at room temperature for 30 min, the mixture was dialyzed against PBS to remove free DiR and then concentrated by ultrafiltration. Samples were stored at 4 °C until needed. Aliquots of LNPs were diluted in PBS and then culture media to a total ASO dose of 50 nM for in vitro knockdown efficacy screenings.

The encapsulation efficiency (EE) was determined using a Quant-iTRiboGreen RNA Assay Kit. In brief, LNPs were diluted in Tris-EDTA (TE) buffer or 0.1% Triton-X 100 TE buffer and followed by the addition of the RiboGreen dye to quantify the unencapsulated and total ASO concentrations for determination of %EE. For Cryo-EM imaging, the LNP-ASO sample was dropped onto a hydrophilic-treated copper grid and then loaded with sample using Vitrobot Mark IV (FEI), resulting in vitreous ice. The grids were observed by Talos F200C G2 TEM (FEI, USA) with an acceleration voltage of 200 kV.

### Evaluation of LNP biodistribution upon intranasal administration in mice

The biodistribution of LNP-ASO was imaged using an IVIS Lumina system (Perkin Elmer) at an excitation of 740 nm and an emission of 790 nm. Fluorescent imaging of collected major organs was performed at various time points (6 h, 24 h, and 48 h) following intranasal delivery of DiR-labeled LNP-ASO. The Living Image 4.4 software’s region of interest (ROI) function was employed to quantify the fluorescence intensity of tissues. The dose of ASO per mouse was set as 4 nmol.

### Statistical analysis

All results are presented as mean ± standard error (SEM). Comparisons between two groups were performed using the Unpaired Student’s *t*-test (two-tailed) for normally distributed data or the Mann–Whitney *U* test (two-tailed) for non-normally distributed data. Comparison between paired samples was conducted using the paired Student’s *t*-test. We used post hoc Tukey multiple comparisons test in one-way ANOVA (normally distributed data), and Dunn’s multiple comparisons test in Kruskal–Wallis test (non-normally distributed data) for pairwise comparisons of difference between three or more groups. Log-rank test was applied to analyze survival curve. The Sharpiro–Wilk test and Kolmogorov-Smirnov test were applied to analyze the normality of the data. The data were analyzed using the Graphpad prism 8 software. The criterion of statistical significance was *p* < 0.05, and symbols indicate *p* values as follows: *p < 0.05; **p < 0.01; ***p < 0.001.

### Reporting summary

Further information on research design is available in the Nature Portfolio Reporting Summary linked to this article.

## Supplementary information

Supplementary Information

Reporting Summary

Transparent Peer Review file

## Source data

Source Data

## Data Availability

All data associated with this study are present in the paper or the Supplementary Materials. The raw RNA sequencing data generated in this study have been deposited in the Genome Sequence Archive in National Genomics Data Center, China National Center for Bioinformation/Beijing Institute of Genomics, Chinese Academy of Science under accession code CRA028702. The raw numbers for charts and graphs are available in the Source Data file whenever possible. All other data are available in the article and its Supplementary files or from the corresponding author upon request. Source data are provided with this paper.
