# Supplementary material for: TDP-43 promotes allergen-induced inflammation and influenza A virus replication in airway epithelium
Source: Nat Commun. 2026 Jul 23;17:8992. doi: 10.1038/s41467-026-75901-0 (PMC13507059; doi:10.1038/s41467-026-75901-0)

Supplementary Materials for

TDP-43 promotes allergen-induced inflammation and influenza A virus replication in airway epithelium

Yanmei Yuan[#], Wenzhu Tang[#], Xixi Feng[#], Chunlei Zhang[#], Yingying Liao[#], Meiyan Tang[#], Jian He[#], Shengpeng Wan, Guihong Pan, Xiaojiang Lai, Qing Xue*, Jun Wang*

[#] These authors contributed equally to this work.

* Correspondence: Email: surky@163.com (Q.X.), junwang2023@sjtu.edu.cn (J.W.)

Materials

Ovalbumins (OVA, A5503), Complete Freund's Adjuvant (CFA, F5881), 3% H_2O_2 (323381), Brefeldin A (BFA, B7651), Pronase (10165921001), DNase I (DN25), PMA (P8139) and Ionomycin (I0634) were obtained from Sigma-Aldrich. House Dust Mite (HDM, XPB70D3A2.5) and Cockroach antigen (CA, XPB46D3A4) were purchased from Greer. LPS (00-4976-93) was purchased from Invitrogen. Alum adjuvant (77161) was ordered from Thermo Fisher Scientific. Collagenase A (10103586001) and proteinase inhibitor (5892791001) were obtained from Roche. Lipids, including ionizable lipid DLin-MC3-DMA (MC3), cholesterol, DSPC (1,2-distearoyl-sn-glycero-3-phosphocholine), and PEG-lipids 1,2-dimyristoyl-rac-glycero-3-methoxy (poly(ethylene glycol-2000)) (DMG-PEG-2K) were obtained from AVT Pharmaceutical Co., Ltd. Fluorescent dye (1,1′-dioctadecyl-3,3,3′,3′-tetramethylindotricarbocyanine iodide (DiR) was purchased from Beyotime. The Tardbp-targeting 20-mer ASOs were synthesized by GenePharma. 4% Paraformaldehyde (BL539A) and ACK (BL503A) were purchased from Biosharp. Recombinant Murine Il13 cytokines (210-13-10 μg) was purchased from PeproTech. PneumaCult-ALI Medium Kit (05001), 0.2% Heparin Solution (07980), Hydrocortisone Stock Solution (07925) and PneumaCult-Ex Plus Medium (05040) were obtained from Stemcell. Transwell (0.4 μm pore size, 3470) was purchased from Corning. Collagen I, Rat Tail (354236) was bought from BioCoat. Murine RNase Inhibitor (RL301-02) was obtained from Vazyme. Proteinase K (10409ES03) was bought from Yeasen. Actinomycin D (HY-17559-5mg) was purchased from MedChemexpress. Anti-TDP-43 (ab109535) were bought from Abcam. Anti-CHRM1 (abs121129) was ordered from Absin. Anti-GAPDH (#5174S) was obtained from Cell signaling technology. Anti-E-cadherin (ET1607-75) was purchased from HUABIO. Anti-Cc10 (RD181022220-01) was ordered from BioVendor R&D. Anti-UPF1(66898-1-lg), anti-MX1 (13750-1-AP), anti-ATF4 (10835-1-AP), anti-Flag (80801-2-RR) and anti-TDP-43 (10782-2-AP) were purchased from Proteintech. Anti-Influenza A virus Nucleoprotein (GTX125989) was bought from GeneTex. Anti-β-tubulin (M20005) and the HRP Goat anti-Rabbit IgG (M21002L) were purchased from Abmart. The DAB peroxidase substrate kit (DA1010) was bought from Solarbio. CD45 microbeads (130-052-301), EpCAM microbeads (130-105-958) and Dead Cell Removal Kit (130-090-101) were obtained from Miltenyi.

Supplementary figures and figure legends

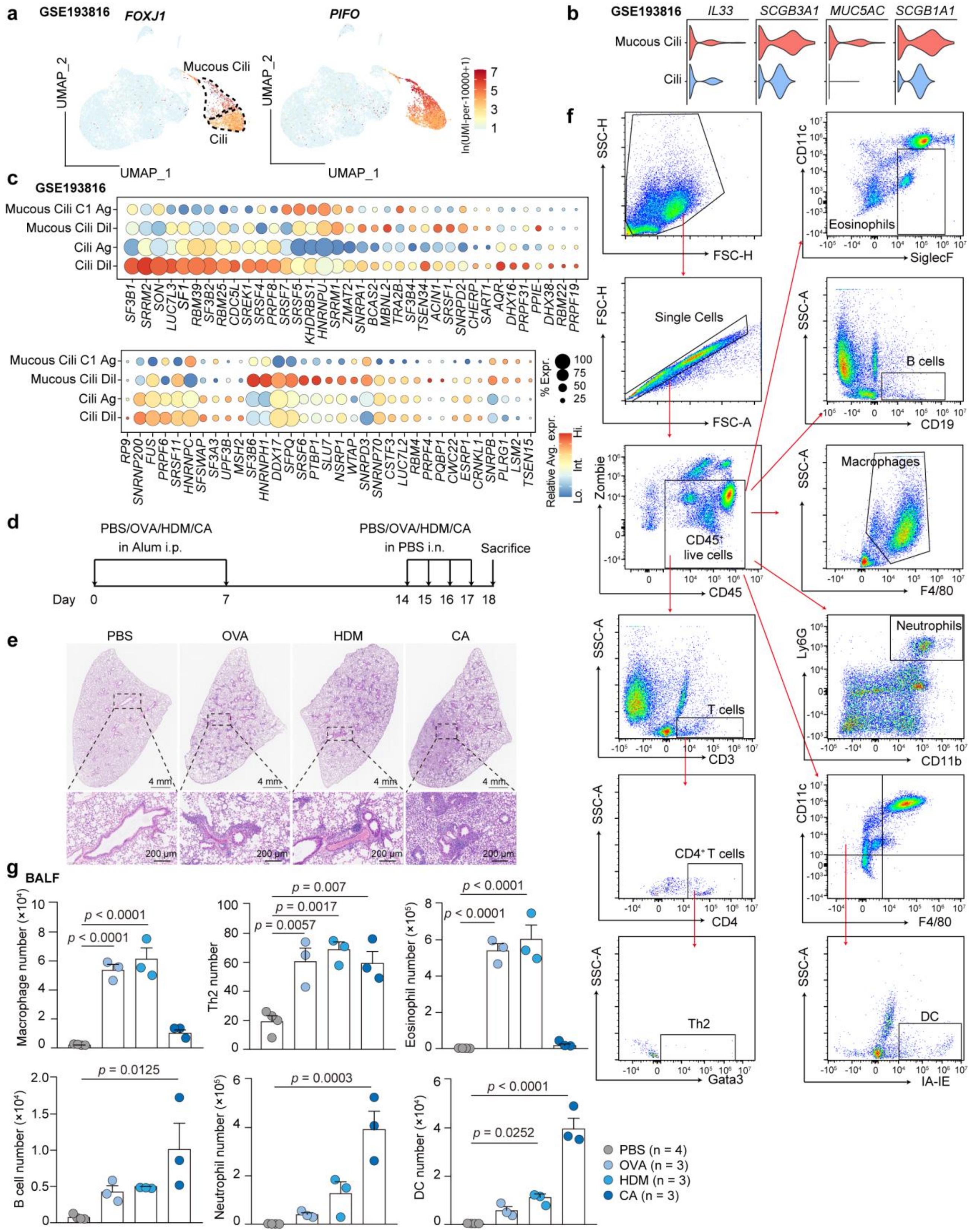

Supplementary Fig. 1 | Establishment of allergen-induced asthmatic mouse models. a, UMAP plots showing marker gene expressions of ciliated cells in scRNA-seq dataset of airway mucosa from allergic asthmatic individuals under allergen or diluent exposure (GSE193816). Color intensity was proportional to its normalized unique molecular identifier (UMI). **b,** Violin plots showing differential gene expressions between mucous Cili and Cili subpopulation. **c,** Dot plots comparing expressions of spliceosome-related genes across ciliated cell subpopulations from subjects with allergen or diluent exposure. Ag: allergen, Dil: diluent. **d,** Schematic diagram illustrating construction of allergen-induced asthmatic mouse models. **e,** H&E staining displaying pulmonary pathologies in asthmatic mice induced by various allergens. **f,** Flow cytometric gating strategy for different immune cell populations in BALF from control mice. **g,** Bar plots comparing abundance of different immune cells in BALF between asthmatic and control mice.

Data are presented as mean ± SEM. Experiments in **g** was performed in two independent replicates, and representative data from one replicate are presented. Significance was assessed by one-way ANOVA with Tukey's multiple comparisons test (**g**). Source data are provided as a Source Data file.

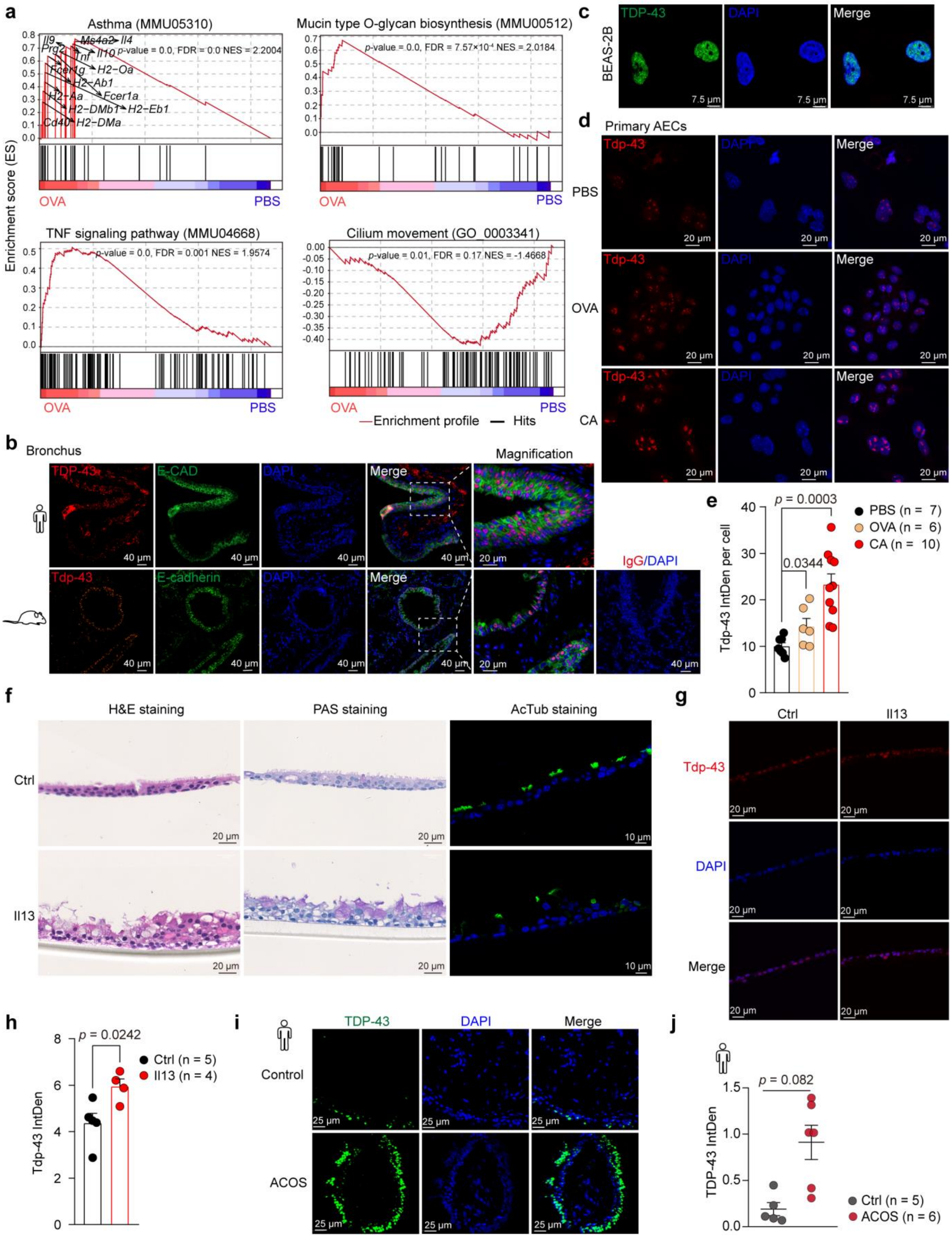
a
Asthma (MMU05310)
p-value = 0.0, FDR = 0.0 NES = 2.2004
Il9
Prg2
Ms4a2
Il4
Tnf
Il10
Fcer1g
H2-Oa
H2-Ab1
Fcer1a
H2-Aa
H2-DMb1
H2-Eb1
Cd40
H2-DMa
Mucin type O-glycan biosynthesis (MMU00512)
p-value = 0.0, FDR = 7.57×10-4 NES = 2.0184
TNF signaling pathway (MMU04668)
p-value = 0.0, FDR = 0.001 NES = 1.9574
Cilium movement (GO_0003341)
p-value = 0.01, FDR = 0.17 NES = -1.4668
Enrichment score (ES)
OVA
PBS
Enrichment profile
Hits
b
Bronchus
TDP-43
E-CAD
DAPI
Merge
Magnification
Tdp-43
E-cadherin
IgG/DAPI
40 μm
20 μm
c
BEAS-2B
TDP-43
DAPI
Merge
7.5 μm
d
Primary AECs
Tdp-43
DAPI
Merge
PBS
OVA
CA
20 μm
e
Tdp-43 IntDen per cell
p = 0.0003
0.0344
PBS (n = 7)
OVA (n = 6)
CA (n = 10)
f
H&E staining
PAS staining
AcTub staining
Ctrl
Il13
20 μm
10 μm
g
Ctrl
Il13
Tdp-43
DAPI
Merge
20 μm
h
p = 0.0242
Tdp-43 IntDen
Ctrl (n = 5)
Il13 (n = 4)
i
TDP-43
DAPI
Merge
Control
ACOS
25 μm
j
p = 0.082
TDP-43 IntDen
Ctrl (n = 5)
ACOS (n = 6)

Supplementary Fig. 2 | Dysregulated TDP-43 expressions in the AECs from allergen-induced asthmatic mice and ACOS patients. a, GSEA analysis of asthma (MMU05310), TNF signaling (MMU04668), mucin type O-glycan biosynthesis and cilium movement (GO_0003341) signaling in AECs from OVA-induced asthmatic mice. **b,** Representative IF images of Tdp-43 and E-Cadherin in the lung tissues from C57BL/6J mice and bronchoscopic mucosal specimens from healthy subjects, IgG was used as a negative control. **c,** Representative IF image of TDP-43 in BEAS-2B cells. **d,** Representative IF images of Tdp-43 in the AECs isolated from CA/OVA-induced mice and control mice. **e,** Quantitative IF results of Tdp-43 IntDen in (**d**). **f,** Representative images of H&E staining, PAS staining and AcTub IF staining in Il13-stimulated and control AECs under air-liquid interface conditions. **g,** Representative IF images of Tdp-43 in Il13-stimulated and control AECs under air-liquid interface conditions. **h,** Quantitative IF results of Tdp-43 IntDen in (**g**). **i,** Representative IF images of TDP-43 in the lungs from patients with ACOS. **j,** Bar plots comparing the Integrated Density of TDP-43 signals in (**i**), each dot represents an individual patient.

Data are presented as mean ± SEM. The data in **d**, **e** have at a minimum 5 independent biological replicates. The data in **b**, **c**, **f**-**h** represent two independent experiments. Significance was assessed by unpaired two-tailed Student's *t*-test (**e**, **h**, **j**). Source data are provided as a Source Data file.

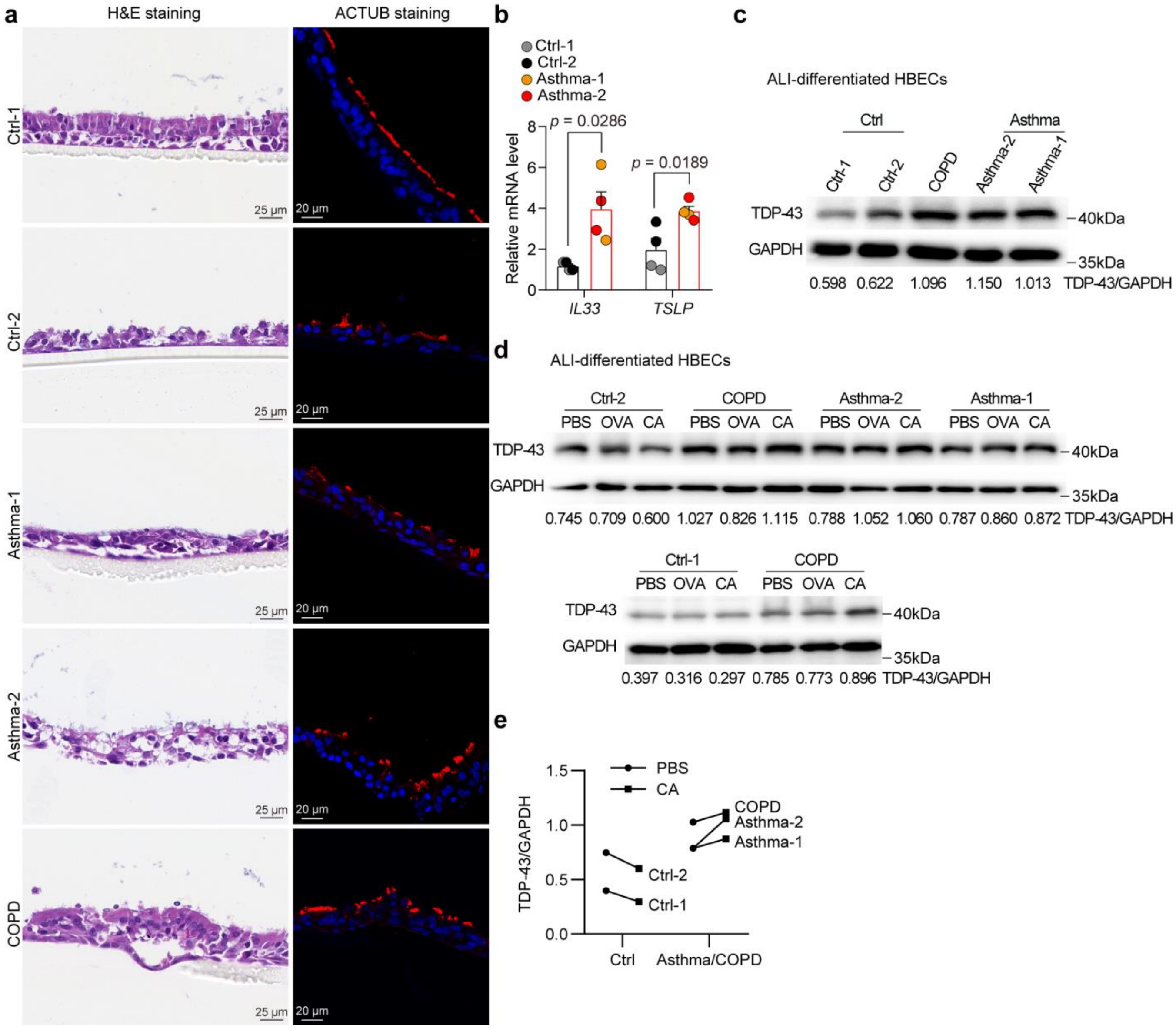

Supplementary Fig. 3 | TDP-43 expression is abnormally elevated in the ALIs differentiated from Asthma and COPD HBECs. a, Representative images of H&E staining and ACTUB IF staining in Asthma, COPD and control patient HBECs under ALI conditions. **b,** mRNA levels of *IL33* and *TSLP* in Asthma and control patient HBECs under ALI conditions, each dot denotes one independent ALI biological replicate. **c,** Immunoblotting of TDP-43 in Asthma, COPD and control patient HBECs under ALI conditions. **d,** Immunoblotting of TDP-43 in Asthma and control patient AECs under ALI conditions and treated with CA (200 μg/ml), OVA (400 μg/ml) or PBS for 24 h. **e,** Quantitative analysis of TDP-43 expression normalized to GAPDH in (**d**).

Data are presented as mean ± SEM. The data in **c**, **d** represent two independent experiments. Significance was assessed by unpaired two-tailed Student's *t*-test, and two-tailed Mann-Whitney *U* test (**b**). Source data are provided as a Source Data file.

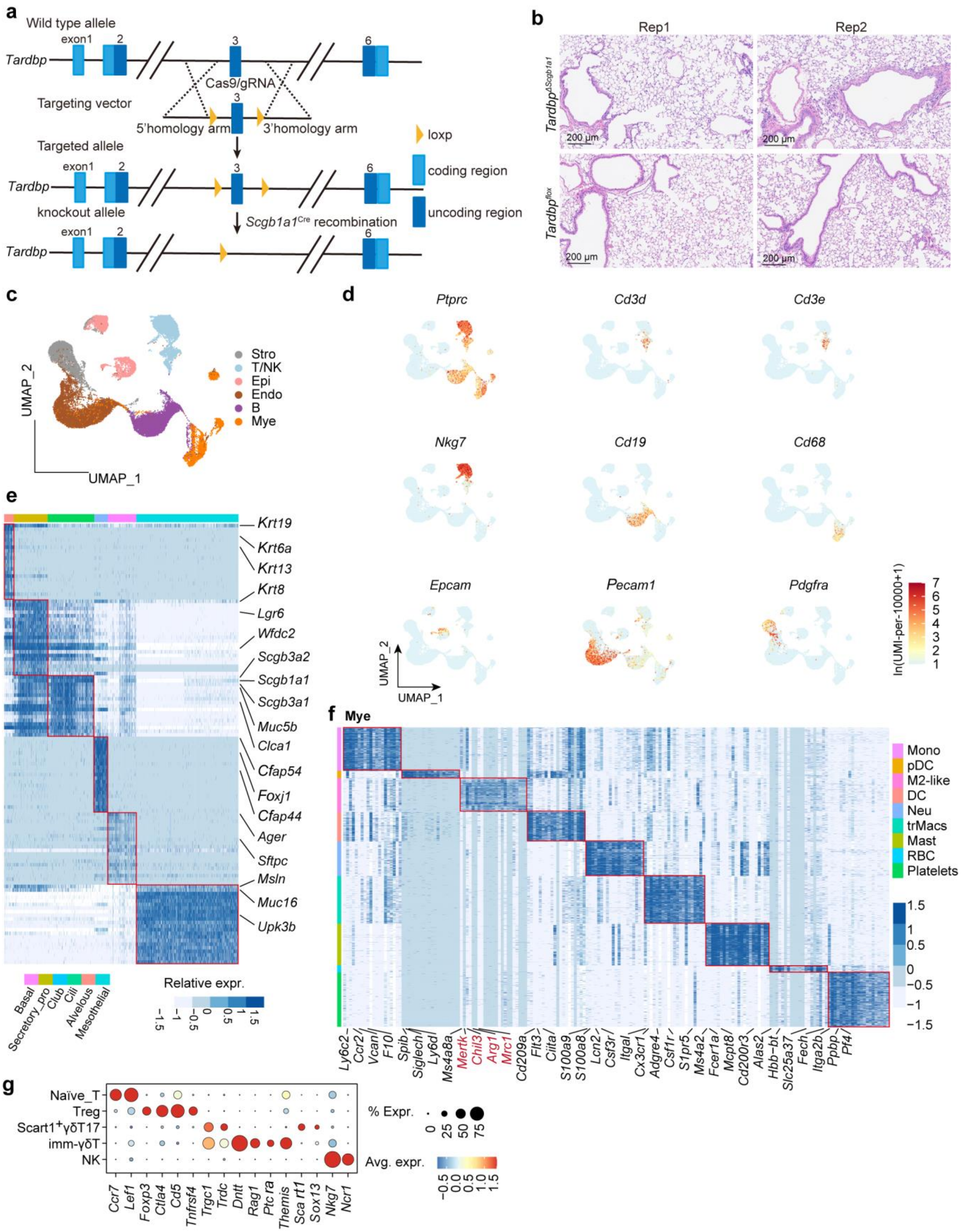

a
Wild type allele
exon1
2
3
6
Tardbp
Cas9/gRNA
Targeting vector
5'homology arm
3'homology arm
loxp
Targeted allele
coding region
knockout allele
Scgb1a1Cre recombination
uncoding region
b
Rep1
Rep2
TardbpΔScgb1a1
Tardbpflox
200 μm
c
Stro
T/NK
Epi
Endo
B
Mye
UMAP_2
UMAP_1
d
Ptprc
Cd3d
Cd3e
Nkg7
Cd19
Cd68
Epcam
Pecam1
Pdgfra
ln(UMI-per-10000+1)
e
Krt19
Krt6a
Krt13
Krt8
Lgr6
Wfdc2
Scgb3a2
Scgb1a1
Scgb3a1
Muc5b
Clca1
Cfap54
Foxj1
Cfap44
Ager
Sftpc
Msln
Muc16
Upk3b
Basal
Secretory_pro
Club
Cili
Alvelous
Mesothelial
Relative expr.
f
Mye
Mono
pDC
M2-like
DC
Neu
trMacs
Mast
RBC
Platelets
Ly6c2
Ccr2
Vcan
F10
Spib
Siglech
Ly6d
Ms4a8a
Mertk
Chil3
Arg1
Mrc1
Cd209a
Flt3
Ciita
S100a9
S100a8
Lcn2
Csf3r
Itgal
Cx3cr1
Adgre4
Csf1r
S1pr5
Ms4a2
Fcer1a
Mcpt8
Cd200r3
Alas2
Hbb-bt
Slc25a37
Fech
Itga2b
Ppbp
Pf4
g
Naïve_T
Treg
Scart1+γδT17
imm-γδT
NK
Ccr7
Lef1
Foxp3
Ctla4
Cd5
Tnfrsf4
Trgc1
Trdc
Dntt
Rag1
Ptcra
Themis
Scart1
Sox13
Nkg7
Ncr1
% Expr.
Avg. expr.

Supplementary Fig. 4 | Transcriptional profiling of airway mucosa by scRNA-seq in OVA-induced *Tardbp*$^{\Delta Scgb1a1}$ mice. a, Schematic diagram displaying the construction strategy of *Tardbp*$^{\Delta Scgb1a1}$ mice. **b,** H&E staining of lung tissues from *Tardbp*$^{\Delta Scgb1a1}$ and *Tardbp*^{flox} mice. **c,** UMAP plot showing the identified subsets of single cell transcriptomes from the lungs of OVA-stimulated *Tardbp*$^{\Delta Scgb1a1}$ and *Tardbp*^{flox} mice. **d,** Major cell types were defined by their marker genes. **e,** Heatmap showing expression of the top 20 differential genes in multiple distinct epithelial cell types. Each column represents an individual cell. **f,** Heatmap showing expression of the top 20 differential genes in different myeloid cell types. Each row represents an individual cell. **g,** Dotplots showing expression level of marker genes of various cell types in B cells and T cells.

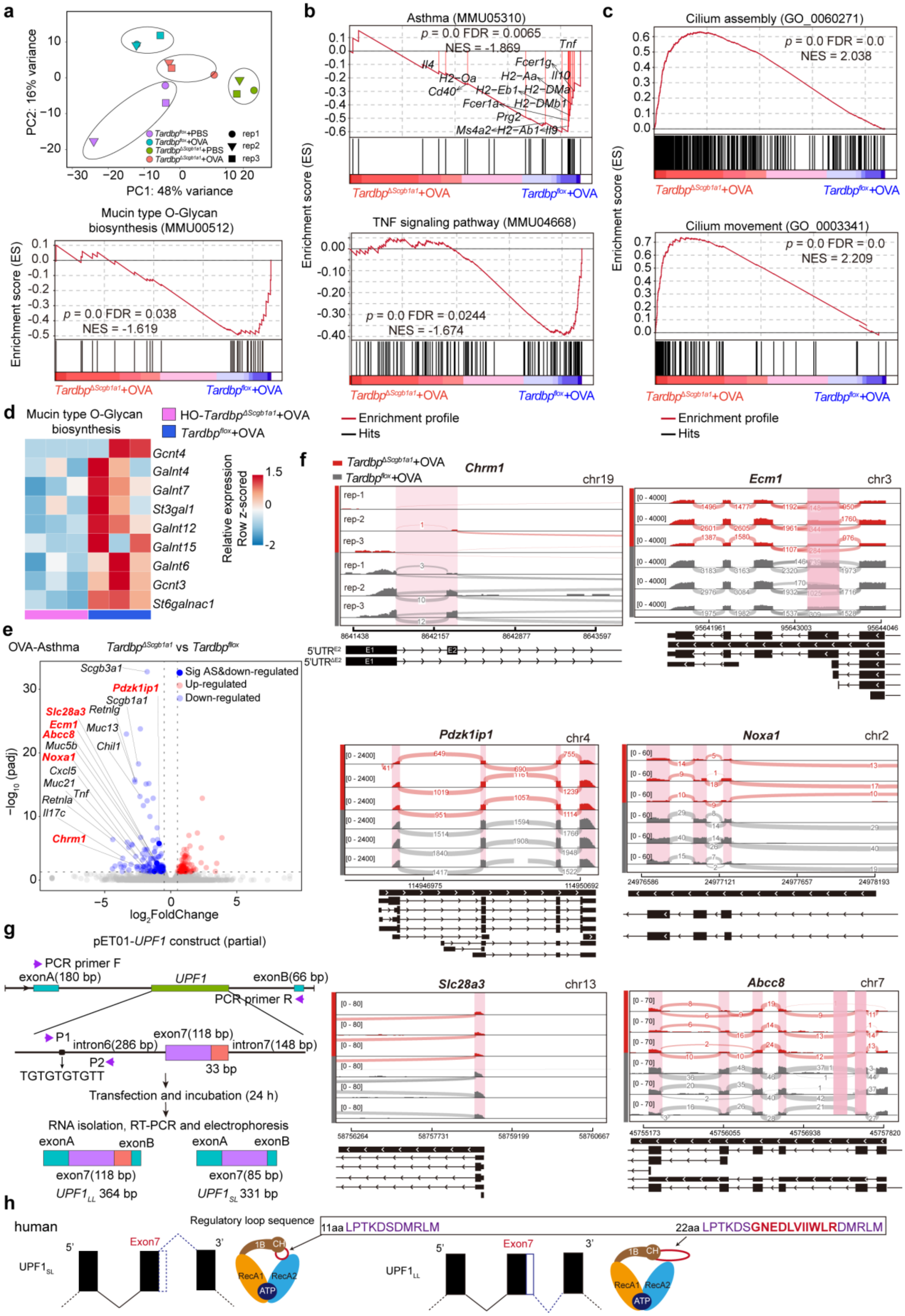
a
PC1: 48% variance
PC2: 16% variance
Tardbp^flox+PBS
Tardbp^flox+OVA
Tardbp^ΔScgb1a1+PBS
Tardbp^ΔScgb1a1+OVA
rep1
rep2
rep3
Mucin type O-Glycan biosynthesis (MMU00512)
p = 0.0 FDR = 0.038
NES = -1.619
Enrichment score (ES)
Tardbp^ΔScgb1a1+OVA
Tardbp^flox+OVA
b
Asthma (MMU05310)
p = 0.0 FDR = 0.0065
NES = -1.869
Tnf
Il4
Fcer1g
H2-Oa
H2-Aa
Il10
Cd40
H2-Eb1
H2-DMa
Fcer1a
H2-DMb1
Prg2
Ms4a2
H2-Ab1
Il9
TNF signaling pathway (MMU04668)
p = 0.0 FDR = 0.0244
NES = -1.674
Enrichment profile
Hits
c
Cilium assembly (GO_0060271)
p = 0.0 FDR = 0.0
NES = 2.038
Cilium movement (GO_0003341)
p = 0.0 FDR = 0.0
NES = 2.209
d
Mucin type O-Glycan biosynthesis
HO-Tardbp^ΔScgb1a1+OVA
Tardbp^flox+OVA
Gcnt4
Galnt4
Galnt7
St3gal1
Galnt12
Galnt15
Galnt6
Gcnt3
St6galnac1
Relative expression Row z-scored
e
OVA-Asthma
Tardbp^ΔScgb1a1 vs Tardbp^flox
Sig AS&down-regulated
Up-regulated
Down-regulated
Scgb3a1
Pdzk1ip1
Scgb1a1
Slc28a3
Retnlg
Ecm1
Muc13
Abcc8
Muc5b
Chil1
Noxa1
Cxcl5
Muc21
Retnla
Tnf
Il17c
Chrm1
−log10 (padj)
log2FoldChange
f
Tardbp^ΔScgb1a1+OVA
Tardbp^flox+OVA
Chrm1
chr19
Ecm1
chr3
Pdzk1ip1
chr4
Noxa1
chr2
Slc28a3
chr13
Abcc8
chr7
5'UTR^E2
5'UTR^ΔE2
g
pET01-UPF1 construct (partial)
PCR primer F
exonA(180 bp)
UPF1
exonB(66 bp)
PCR primer R
P1
intron6(286 bp)
exon7(118 bp)
intron7(148 bp)
P2
TGTGTGTGTT
33 bp
Transfection and incubation (24 h)
RNA isolation, RT-PCR and electrophoresis
exonA
exonB
exon7(118 bp)
UPF1LL 364 bp
exon7(85 bp)
UPF1SL 331 bp
h
human
Regulatory loop sequence
11aa LPTKDSDMRLM
22aa LPTKDSGNEDLVIIWLRDMRLM
UPF1SL
UPF1LL
Exon7
RecA1
RecA2
ATP

Supplementary Fig. 5 | Smart-seq2 analysis of AECs from OVA-induced *Tardbp*^{flox} or *Tardbp*$^{\Delta Scgb1a1}$ mice. **a,** PCA plot showing the distribution of Smart-seq2 samples from various groups. **b, c,** GESA analysis revealing that AECs from asthmatic *Tardbp*$^{\Delta Scgb1a1}$ mice had decreased expressions of genes enriched in asthma, mucin type O-glycan biosynthesis and TNF signaling pathway (**b**) and increased expressions of genes enriched in cilium assembly and movement (**c**). **d,** Heatmap displaying the expressions of genes encoding enzymes involved in mucin O-Glycan biosynthesis in the AECs from *Tardbp*$^{\Delta Scgb1a1}$ and *Tardbp*^{flox} mice with OVA exposure. **e,** Volcano plot exhibiting differentially expressed genes between AECs from OVA-stimulated *Tardbp*$^{\Delta Scgb1a1}$ and *Tardbp*^{flox} mice. Genes labeled in red were also differentially spliced. **f,** Sashimi plots depicting altered splicing patterns in various transcripts within AECs between OVA-induced *Tardbp*^{flox} and *Tardbp*$^{\Delta Scgb1a1}$ mice. **g,** Schematic diagram of the assay for UPF1 minigene splicing. **h,** A schematic diagram comparing the differences between the two transcript isoforms of gene UPF1.

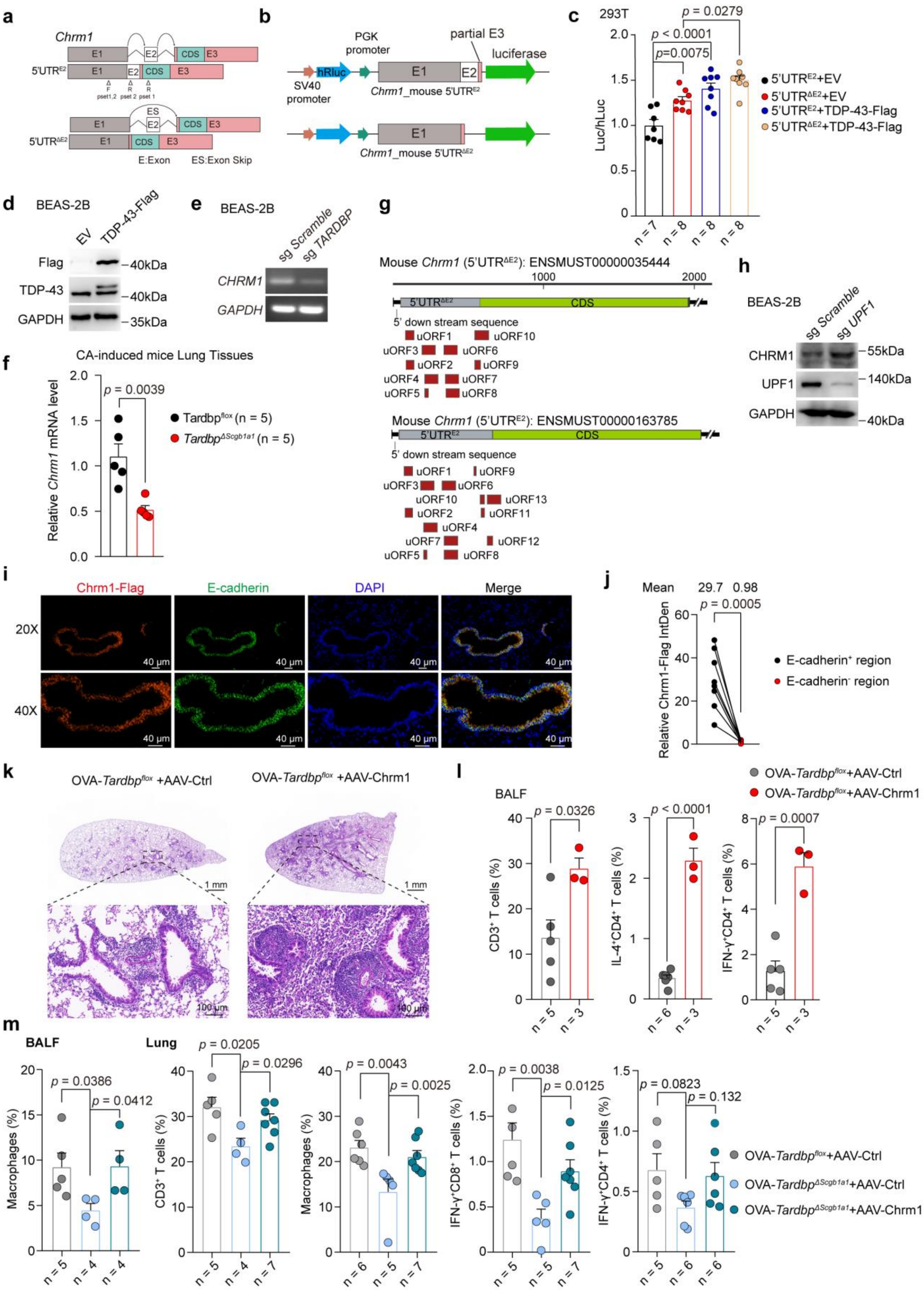
a
Chrm1
5'UTR^E2
5'UTR^ΔE2
E:Exon
ES:Exon Skip
b
PGK promoter
partial E3
luciferase
SV40 promoter
hRluc
Chrm1_mouse 5'UTR^E2
Chrm1_mouse 5'UTR^ΔE2
c
293T
Luc/hLuc
5'UTR^E2+EV
5'UTR^ΔE2+EV
5'UTR^E2+TDP-43-Flag
5'UTR^ΔE2+TDP-43-Flag
d
BEAS-2B
e
BEAS-2B
f
CA-induced mice Lung Tissues
Relative Chrm1 mRNA level
Tardbp^flox (n = 5)
Tardbp^ΔScgb1a1 (n = 5)
g
Mouse Chrm1 (5'UTR^ΔE2): ENSMUST00000035444
Mouse Chrm1 (5'UTR^E2): ENSMUST00000163785
h
BEAS-2B
i
Chrm1-Flag
E-cadherin
DAPI
Merge
j
Relative Chrm1-Flag IntDen
E-cadherin⁺ region
E-cadherin⁻ region
k
OVA-Tardbp^flox +AAV-Ctrl
OVA-Tardbp^flox +AAV-Chrm1
l
BALF
OVA-Tardbp^flox+AAV-Ctrl
OVA-Tardbp^flox+AAV-Chrm1
m
BALF
Lung
OVA-Tardbp^flox+AAV-Ctrl
OVA-Tardbp^ΔScgb1a1+AAV-Ctrl
OVA-Tardbp^ΔScgb1a1+AAV-Chrm1

Supplementary Fig. 6 | TDP-43 regulates the expression of Chrm1 in AECs. a, Schematic diagram of splicing variants of *Chrm1* transcripts and the primers for PCR detection of exon 1-3 (primer set 1) and exons 1-2 (primer set 2) inclusion/exclusion in mice. **b,** Schematic diagram showing the constructs carrying the Luc reporter driven by the 5'UTR^{E2} and 5'UTR$^{\Delta E2}$ of mouse *Chrm1*. **c,** Bar charts comparing relative Luc activities (Luc/hLuc) directed by 5'UTR^{E2} and 5'UTR$^{\Delta E2}$ of mouse *Chrm1* in 293T cells with or without TDP-43 overexpression. **d,** Immunoblotting of TDP-43 and Flag in TDP-43-overexpressed BEAS-2B cells. GAPDH was used as a loading control. **e,** PCR detecting the mRNA level of *CHRM1* in TDP-43-knockdown BEAS-2B cells. **f,** mRNA level of *Chrm1* in lung tissues from CA-induced *Tardbp*^{flox} or *Tardbp*$^{\Delta Scgb1a1}$ mice. **g,** The locations of potential uORFs with strong Kozak strength in the two mouse *Chrm1* transcripts. **h,** Immunoblotting of CHRM1 and UPF1 in UPF1-knockdown BEAS-2B cells. GAPDH was used as a loading control. **i,** Representative IF images of Flag and E-cadherin in lungs from OVA-induced *Tardbp*$^{\Delta Scgb1a1}$ and *Tardbp*^{flox} mice receiving i.n. infection of AAV-Chrm1. **j,** Bar plots comparing the IntDen of Flag signals between E-cadherin positive regions and E-cadherin negative regions. **k,** H&E staining comparing pathologies of OVA-induced *Tardbp*^{flox} mice receiving i.n. infection of AAV-Ctrl or AAV-Chrm1. **l,** Bar charts showing the infiltrations of Th2 (IL-4^{+}CD4^{+}) and Th1 (IFN-γ^{+}CD4^{+}) cells in BALF from OVA-induced asthmatic mice with prior infection of AAV-Chrm1. **m,** Bar plots showing flow cytometric results of BALF and pulmonary immune cell infiltrations in OVA induced-*Tardbp*$^{\Delta Scgb1a1}$ and *Tardbp*^{flox} mice receiving i.n. infection of AAV-Ctrl or AAV-Chrm1.

Data are presented as mean ± SEM. The data in **c, f**, **i**, **j** have at a minimum 5 independent biological replicates. Experiments in **d**, **e**, **h, l, m** were performed in two independent replicates, and representative data from one replicate are presented. Significance was assessed by unpaired two-tailed Student's t-test (**f**, **l**, **m**), two-tailed Mann-Whitney *U* test (**m**), one-way ANOVA with Tukey's multiple comparisons test (**c**), and paired two-tailed Student's *t*-test (**j**). Source data are provided as a Source Data file.

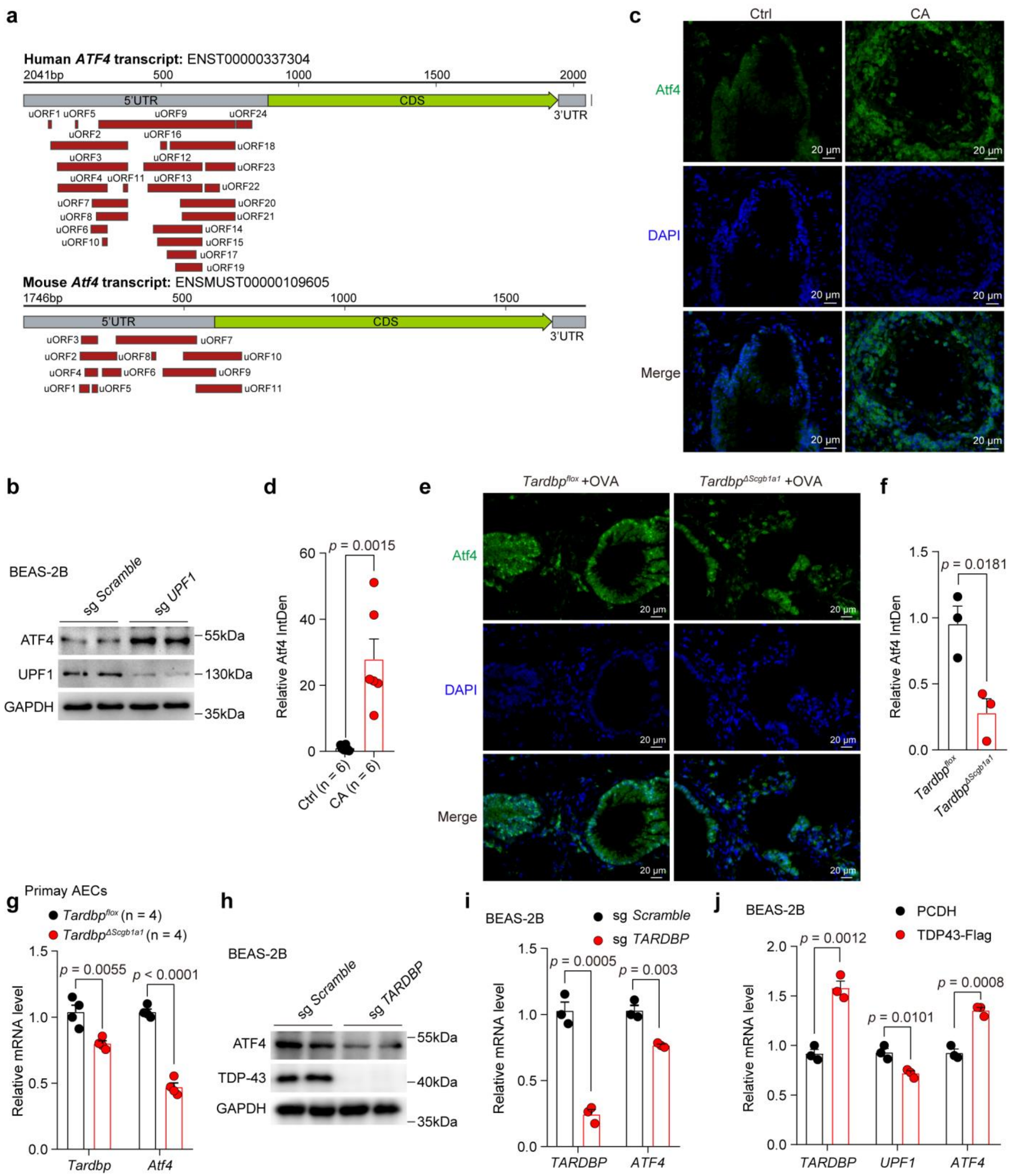

Supplementary Fig. 7 | TDP-43 regulates the expression of Atf4 in AECs. a, The locations of potential uORFs with strong Kozak strength in human and mouse *ATF4* transcripts. **b,** Immunoblotting of ATF4 and UPF1 in UPF1-knockdown BEAS-2B cells. GAPDH was used as a loading control. **c,** Representative

IF images of Atf4 in CA-stimulated mouse lung tissues and control. **d,** Bar plots comparing the IntDen of Atf4 signals in (**c**), each dot represents an individual mouse. **e,** Representative IF images of Atf4 in OVA-induced $Tardbp^{\Delta Scgb1a1}$ and $Tardbp^{flox}$ mouse lung tissues. **f,** Bar plots comparing the IntDen of Atf4 signals in (**e**), each dot represents an individual mouse. **g,** mRNA levels of *Tardbp* and *Atf4* in primary AECs isolated from CA-induced $Tardbp^{\Delta Scgb1a1}$ and $Tardbp^{flox}$ mice. **h,** Immunoblotting of ATF4 and TDP-43 in TDP-43-knockdown BEAS-2B cells. GAPDH was used as a loading control. **i,** mRNA levels of *TARDBP* and *ATF4* in TDP-43-knockdown BEAS-2B cells. **j,** mRNA levels of *TARDBP*, *UPF1* and *ATF4* in TDP-43-overexpressed BEAS-2B cells.

Data are presented as mean ± SEM. The data in **c**, **d** have at a minimum 6 independent biological replicates. Experiments in **b**, **e-j** were performed in two independent replicates, and representative data from one replicate are presented. Significance was assessed by unpaired two-tailed Student's *t*-test (**d**, **f**, **g**, **i**, **j**). Source data are provided as a Source Data file.

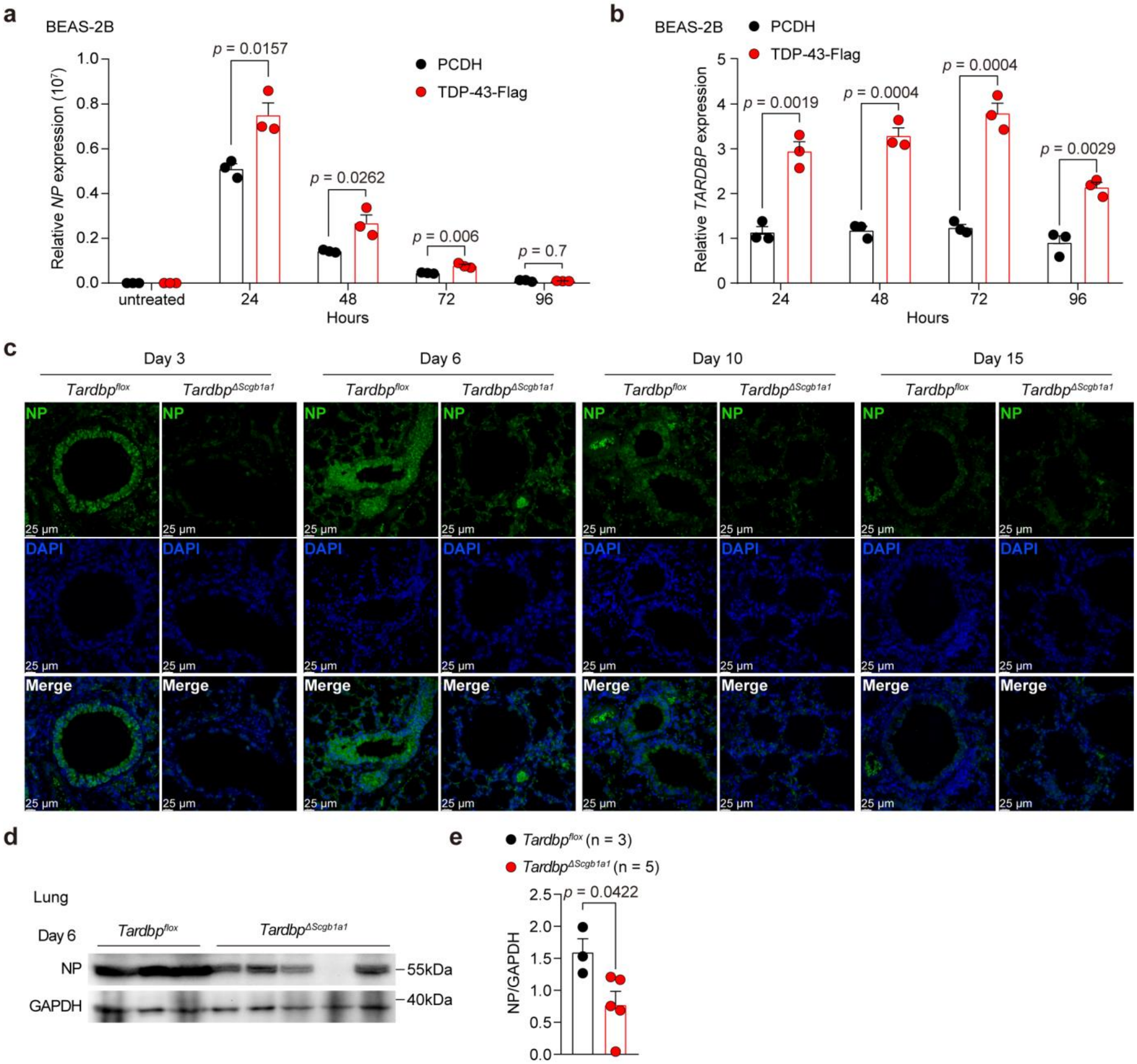

Supplementary Fig. 8 | *Tardbp$^{\Delta Scgb1a1}$* mice are hypo-sensitive to IAV infection. a, mRNA level of *NP* in TDP-43-overexpressed BEAS-2B cells infected with PR8 at different time points, untreated indicates no PR8 infection. **b,** mRNA level of *TARDBP* in TDP-43-overexpressed BEAS-2B cells infected with PR8 at different time points. **c,** Representative IF images of NP in lungs from *Tardbp$^{\Delta Scgb1a1}$* and *Tardbp^{flox}* mice infected with the PR8 at different time points. **d**, Immunoblotting of NP in lungs from *Tardbp$^{\Delta Scgb1a1}$* and *Tardbp^{flox}* mice infected with the PR8 for 6 days. **e**, Quantitative analysis of NP expression normalized to GAPDH in (**d**), each dot represents an individual mouse.

Data are presented as mean ± SEM. Experiments in **a, b, d, e** were performed in two independent replicates, and representative data from one replicate are presented. Significance was assessed by unpaired two-tailed Student's *t*-test (**a, b, e**), and two-tailed Mann-Whitney *U* test (**a**). Source data are provided as a Source Data file.

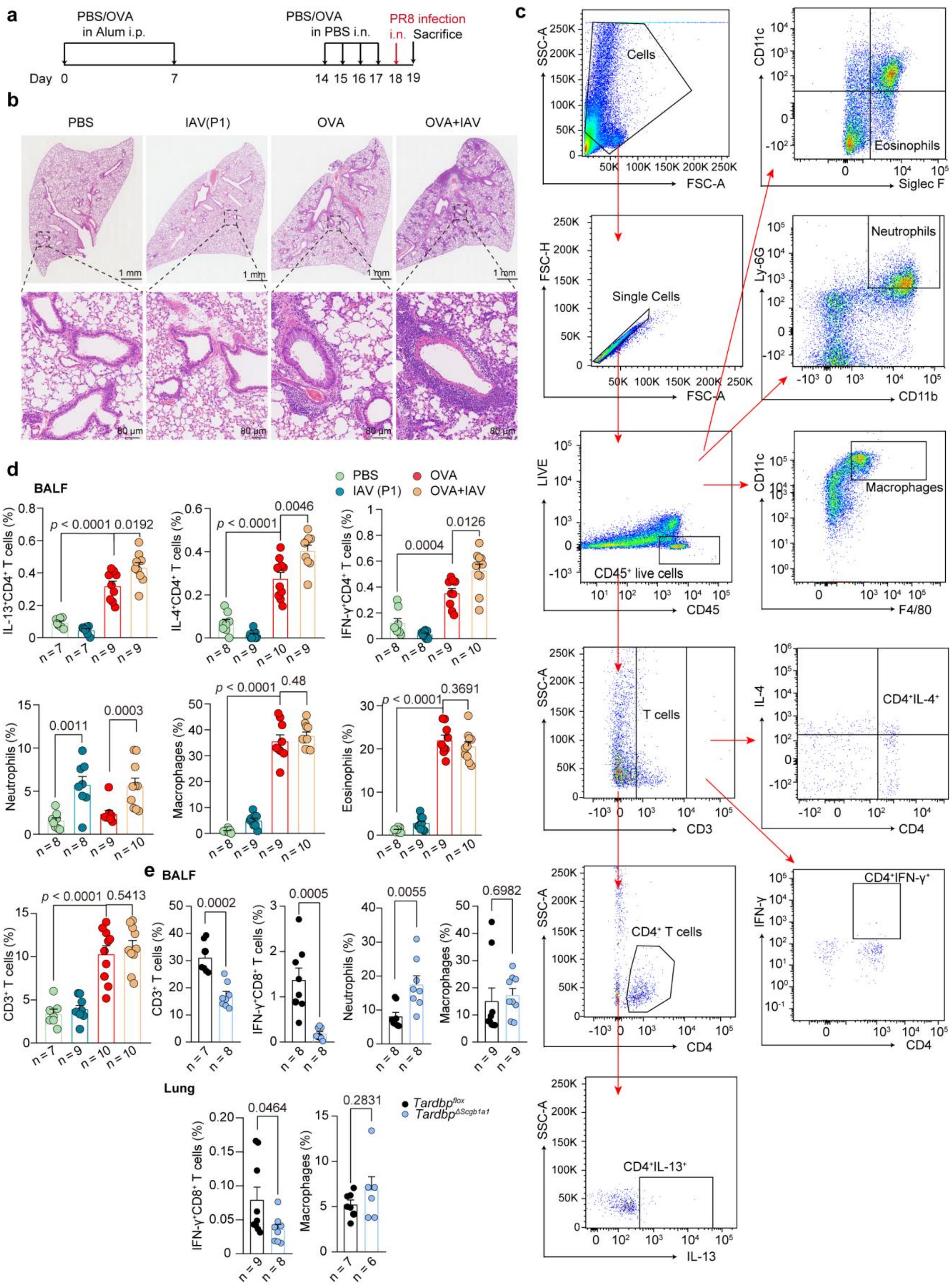
a
PBS/OVA
in Alum i.p.
PBS/OVA
in PBS i.n.
PR8 infection
i.n.
Sacrifice
Day 0 7 14 15 16 17 18 19
b
PBS
IAV(P1)
OVA
OVA+IAV
1 mm
80 μm
c
Cells
Single Cells
CD45+ live cells
T cells
CD4+ T cells
CD4+IL-13+
Eosinophils
Neutrophils
Macrophages
CD4+IL-4+
CD4+IFN-γ+
SSC-A
FSC-A
FSC-H
LIVE
CD45
CD3
CD4
IL-13
CD11c
Siglec F
Ly-6G
CD11b
F4/80
IL-4
IFN-γ
d
BALF
PBS
IAV (P1)
OVA
OVA+IAV
IL-13+CD4+ T cells (%)
IL-4+CD4+ T cells (%)
IFN-γ+CD4+ T cells (%)
Neutrophils (%)
Macrophages (%)
Eosinophils (%)
CD3+ T cells (%)
p < 0.0001
0.0192
0.0046
0.0004
0.0126
0.0011
0.0003
0.48
0.3691
0.5413
e
BALF
Lung
CD3+ T cells (%)
IFN-γ+CD8+ T cells (%)
Neutrophils (%)
Macrophages (%)
0.0002
0.0005
0.0055
0.6982
0.0464
0.2831
Tardbp^flox
Tardbp^ΔScgb1a1

Supplementary Fig. 9 | Establishment of murine IAV-AE model. a, Schematic diagram depicting construction of OVA-induced and virus infection exacerbated asthmatic mouse models. **b,** H&E staining of lung tissues from different groups of asthmatic mice. **c,** Flow cytometric gating strategy for different immune cell populations in BALF from asthmatic mice. **d,** Bar plots comparing infiltrations of immune cell populations in BALF across different groups of mice. **e,** Bar plots showing flow cytometric results of immune cell infiltrations in the lungs and BALF of *Tardbp*$^{\Delta Scgb1a1}$ and *Tardbp*^{flox} mice with IAV-AEs. Data are presented as mean ± SEM. The data in **d**, **e** have at a minimum 6 independent biological replicates. Significance was assessed by unpaired two-tailed Student's *t*-test (**d**, **e**), and two-tailed Mann-Whitney *U* test (**d**, **e**). Source data are provided as a Source Data file.

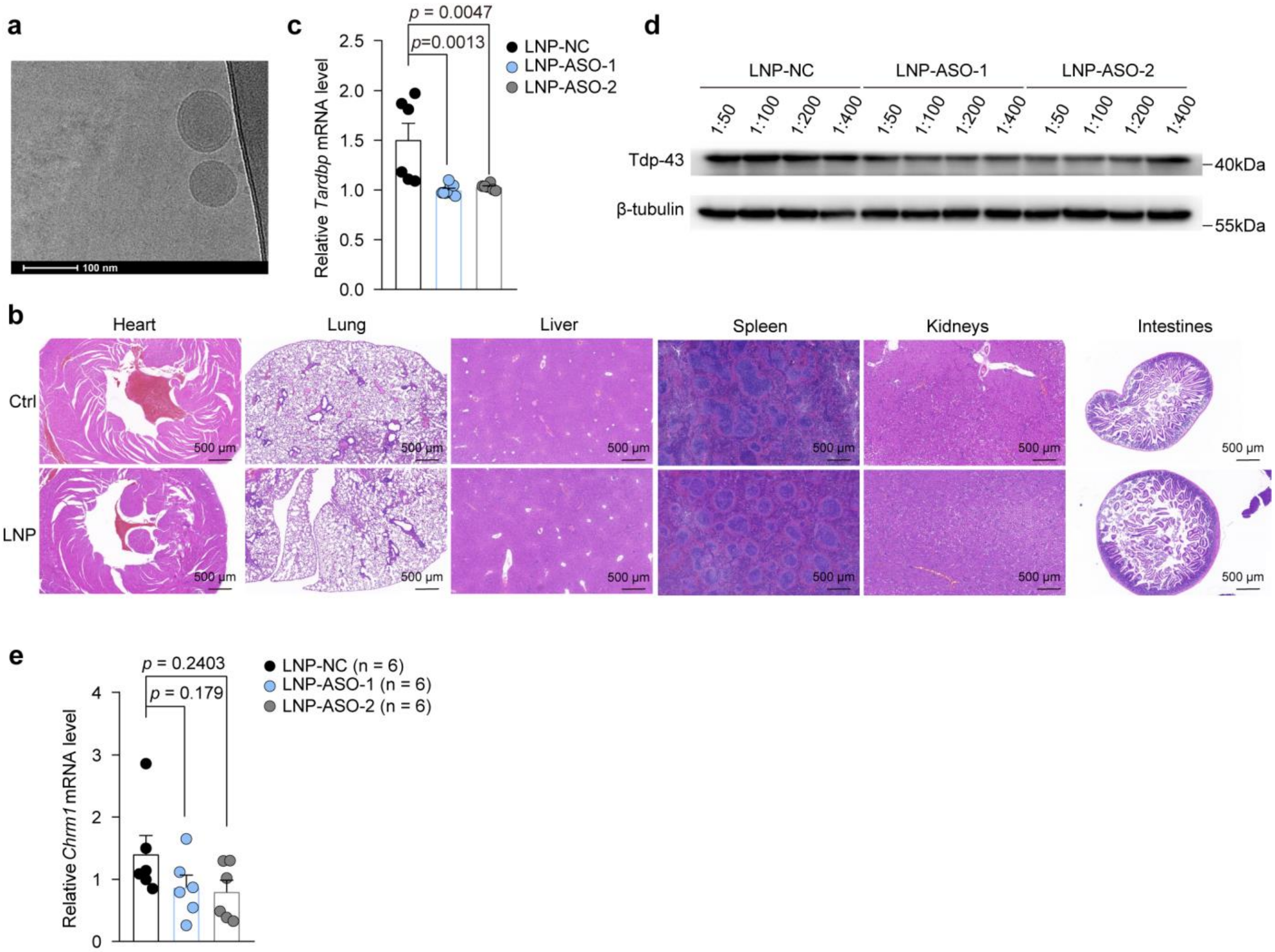

Supplementary Fig. 10 | Preparation of LNP-ASO targeting Tdp-43. a, Representative cryo-TEM image of LNP-ASO. Scale bar: 100 nm. **b,** Representative H&E images showing no obvious pathologies in multiple tissues of mice with LNP treatment. **c,** Bar plots showing the *Tardbp* expression in B16 cells treated with LNP-ASO. **d,** Immunoblots of Tdp-43 in B16 cells treated with LNP-ASO. β-tubulin was used as a loading control. **e,** Bar plots showing the *Chrm1* mRNA level in B16 treated with LNP-ASO. Data are presented as mean ± SEM. Data are representative of two independent experiments. Significance was assessed by one-way ANOVA with Tukey's multiple comparisons test (**c**), and two-tailed Mann-Whitney *U* test (**e**). Source data are provided as a Source Data file.

Supplementary tables

Supplementary Table 1. The list of RQC signature genes.

**RQC signature genes**
SMG9, *SMG6*, *LUC7L3*, *U2AF1*, *RBM22*, *SF3B1*, *SRSF1*, *SRSF6*, *SRSF7*, *SRSF5*, *SLU7*, *SFPQ*, *SF3B4*, *SF3B2*, *SRSF4*, *SF3A3*, *SRSF11*, *SF3B6*, *TSEN34*, *SF1*, *TSEN15*, *SFSWAP*, *SREK1*, *SRSF8*, *CRNKL1*, *ESRP1*, *U2AF1*, *PRPF4*, *PRPF8*, *NSRP1*, *MBNL2*, *TRA2B*, *MBNL3*, *PTBP1*, *YJU2*, *PRPF19*, *RBM39*, *LUC7L2*, *RP9*, *PRPF6*, *SNRNP70*, *DHX16*, *CDC5L*, *DHX38*, *ERH*, *SRRM1*, *PRPF31*, *KHDRBS1*, *SRRM2*, *CWC22, SNRPB*, *SNRNP200*, *DHX15*, *RBM22*, *FUS*, *NF1*, *SNRPD3*, *BCAS2*, *RBM25*, *SNRPD2*, *ACIN1*, *WTAP*, *HNRNPA2B1*, *HNRNPH1*, *DDX17*, *PLRG1*, *PQBP1*, *HNRNPC*, *SON, MSH2*, *HNRNPU*, *LSM2*, *SNRPA1*, *RBM4*, *UPF3B*, *UPF2*, *UPF1*, *ETF1*, *SMG1*, *SMG7*, *CASC3*, *MAGOH*, *SMG5*, *CSTF3*, *AQR*, *SNRNP200*, *ZMAT2*, *SART1*, *DHX16*, *CRNKL1*, *PRPF6*, *SRSF7*, *HNRNPC*, *PRPF31*, *DHX38*, *PRPF8*, *UPF1*, *CHERP*, *PRPF19*, *SNRPD2*, *SF3B4*, *PPIE*, *UPF3B*, *UPF2*, *UPF1*, *ETF1*, *SMG1*, *SMG7*, *CASC3*, *MAGOH*.

Supplementary Table 2. The canonical marker genes for each cell subpopulation.

Lineage	Pan-group marker	Cluster_name	In-group marker
Epithelium	*Epcam*	Basal	*Krt19, Krt6a, Krt13*
		Secretory_pro	*Lgr6, Msln, Muc16, Krt8, Wfdc2*
		Club	*Scgb3a2, Scgb1a1, Scgb3a1, Muc5b, Clca1*
		Cili	*Foxj1, Cfap44, Cfap54*
		Alvelous	*Ager, Sftpc*
		Mesothelial	*Msln, Upk3b, Muc16*
Endothelium	*Pecam1*	Arterial	*Calcrl, Ace, Epas1, Bmpr2*
		Venous	*Pltp, Gja4, Kit, Glp1r*
		aCap	*Car4, Apln, Igfbp7*
		aVenous	*Vwf, Bst1, Selp, Ptgs2, Ch25h*
		LEC	*Flt4, Prox1, Pdpn, Ccl21a*
		Arteriolar	*Fabp4, Aqp7, C1qtnf9, Pde2a, Kcna5, Rgcc*
Stromal cells	*Pdgfra*	AlvF	*Lum, Dpt, Gsn*
		Pericytes	*Mfap4, Itga8, Npnt, Limch1*
		MPC	*Pi16, Ackr3, Islr, Pcsk6, Ebf1*
		LipoF	*Gatm, Wnt4, Fgfr2, Il34*
		Mitotic-stro	*Mki67, Top2a, Cenpf*
T/NK	*Cd3d, Cd3e* or *Nkg7*	Naïve T	*Ccr7, Lef1, Tcf7*
		Treg	*Foxp3, Ctla4, Cd5, Tnfrsf4*
		Scart1_γδT17	*Trgc1, Trdc, Trgc2, Scart1, Sox13*
		imm-γδT	*Dntt, Rag1, Ptcra, Themis*
		NK	*Nkg7, Ncr1*
B cells	*Cd19*	Naïve B	*Ighd, Fcer2a, Cr2*
		Act_B	*Ccr7, Klf2, Cd69, Sell*
		MBC	*Spib, Cxcr5, Prdm1, Zbtb32, Cd80*
		PB	*Mki67, Sdc1, Jchain, Spi1*
Myeloid cells	*Cd68*	trMacs	*Csf1r, Adgre4, Cx3cr1, Itgal, S1pr5*
		Mast	*Fcer1a, Ms4a2, Mcpt8, Cd200r3*
		Mono	*Ly6c2, Ccr2, F10, Vcan*
		Neu	*S100a8, S100a9, Csf3r, Lcn2*
		DC	*Cd209a, Flt3, Ciita*
		M2-like	*Mrc1, Arg1, Chil3, Mertk, Ms4a8a*
		pDC	*Siglech, Spib, Ly6d*
		RBC	*Alas2, Fech, Slc25a37, Hbb-bt*
		Platelets	*Pf4, Ppbp, Itga2b*

Supplementary Table 3. uORF information of genes with differential alternative splicing patterns between *Tardbp*$^{\Delta Scgb1a1}$ and *Tardbp*^{flox} groups.

Symbol	Species	NCBI ID	5'UTR length (bp)	uORFs
CHRM1	Human	NM_000738.3	224	29
Chrm1-5'UTR$^{\Delta E2}$	Mouse	NM_007698.3	524	68
Chrm1-5'UTR^{E2}	Mouse	NM_001112697.1	616	81
NOXA1	Human	NM_001256068.2	137	7
NOXA1	Human	NM_001256067.2	137	7
NOXA1	Human	NM_006647.2	137	7
Noxa1	Mouse	NM_172204.4	281	32
Noxa1	Mouse	NM_001163626.1	281	32
ECM1	Human	NM_001202858.2	101	17
ECM1	Human	NM_022664.3	101	17
ECM1	Human	NM_004425.4	101	17
Ecm1	Mouse	NM_007899.4	127	11
Ecm1	Mouse	NM_001252653.3	127	11
Ecm1	Mouse	NM_001355070.2	127	11
SLC28A3	Human	NM_001199633.2	125	17
SLC28A3	Human	NM_022127.3	321	43
Slc28a3	Mouse	NM_022317.3	70	12

Supplementary Table 4. Predicted uORFs in the 5'UTRs of human and mouse *CHRM1* transcripts.

Name	uORF ID	Kozak strength	Exonic sequence
**Human *CHRM1* 5'UTR**	uORF1	adequate	CTGGCTGTGCCTCTCCCGCTGCCAGCACAGGGCG<br>CAGCTCCCCCTGGGAGCCAGGTGTTTGGGTCCCT<br>GGAGACGCCGCAGGCCCCCAGGGAGGCAGTGGG<br>GCTGAGGACCCTACAGACCCCTCTTCAGCCCCGT<br>GGTGATGACTTTCCCCTGAGGAAGCCCTGTAGCG<br>TGCCTGGAGGAAGGGGCTCTCCAACCCCAGCCCC<br>ACCTAGCCACCATGAACACTTCAGCCCCACCTGC<br>TGTCAGCCCCAACATCACCGTCCTGGCACCAGGA<br>AAGGGTCCCTGGCAAGTGGCCTTCATTGGGATCA<br>CCACGGGCCTCCTGTCGCTAGCCACAGTGACAGG<br>CAACCTGCTGGTACTCATCTCTTTCAAGGTCAACA<br>CGGAGCTCAAGACAGTCAATAA
	uORF2	adequate	GTGCCTCTCCCGCTGCCAGCACAGGGCGCAGCTC<br>CCCCTGGGAGCCAGGTGTTTGGGTCCCTGGAGAC<br>GCCGCAGGCCCCCAGGGAGGCAGTGGGGCTGAG<br>GACCCTACAGACCCCTCTTCAGCCCCGTGGTGAT<br>GACTTTCCCCTGAGGAAGCCCTGTAGCGTGCCTG<br>GAGGAAGGGGCTCTCCAACCCCAGCCCCACCTAG<br>CCACCATGAACACTTCAGCCCCACCTGCTGTCAG<br>CCCCAACATCACCGTCCTGGCACCAGGAAAGGGT<br>CCCTGGCAAGTGGCCTTCATTGGGATCACCACGG<br>GCCTCCTGTCGCTAGCCACAGTGACAGGCAACCT<br>GCTGGTACTCATCTCTTTCAAGGTCAACACGGAG<br>CTCAAGACAGTCAATAA
	uORF3	adequate	AGGGCGCAGCTCCCCCTGGGAGCCAGGTGTTTGG<br>GTCCCTGGAGACGCCGCAGGCCCCCAGGGAGGCA<br>GTGGGGCTGA
	uORF4	adequate	CTGGGAGCCAGGTGTTTGGGTCCCTGGAGACGCC<br>GCAGGCCCCCAGGGAGGCAGTGGGGCTGA
	uORF5	adequate	AGGTGTTTGGGTCCCTGGAGACGCCGCAGGCCCC<br>CAGGGAGGCAGTGGGGCTGA
	uORF6	adequate	TTGGGTCCCTGGAGACGCCGCAGGCCCCCAGGGA<br>GGCAGTGGGGCTGA
	uORF7	adequate	CTGGAGACGCCGCAGGCCCCCAGGGAGGCAGTG<br>GGGCTGAGGACCCTACAGACCCCTCTTCAGCCCC

			GTGGTGATGACTTTCCCCTGAGGAAGCCCTGTAG<br>CGTGCCTGGAGGAAGGGGCTCTCCAACCCCAGCC<br>CCACCTAGCCACCATGAACACTTCAGCCCCACCT<br>GCTGTCAGCCCCAACATCACCGTCCTGGCACCAG<br>GAAAGGGTCCCTGGCAAGTGGCCTTCATTGGGAT<br>CACCACGGGCCTCCTGTCGCTAGCCACAGTGACA<br>GGCAACCTGCTGGTACTCATCTCTTTCAAGGTCAA<br>CACGGAGCTCAAGACAGTCAATAA
	uORF8	adequate	ACGCCGCAGGCCCCCAGGGAGGCAGTGGGGCTG<br>AGGACCCTACAGACCCCTCTTCAGCCCCGTGGTG<br>ATGACTTTCCCCTGAGGAAGCCCTGTAGCGTGCC<br>TGGAGGAAGGGGCTCTCCAACCCCAGCCCCACCT<br>AGCCACCATGAACACTTCAGCCCCACCTGCTGTC<br>AGCCCCAACATCACCGTCCTGGCACCAGGAAAGG<br>GTCCCTGGCAAGTGGCCTTCATTGGGATCACCAC<br>GGGCCTCCTGTCGCTAGCCACAGTGACAGGCAAC<br>CTGCTGGTACTCATCTCTTTCAAGGTCAACACGGA<br>GCTCAAGACAGTCAATAA
	uORF9	adequate	AGGGAGGCAGTGGGGCTGAGGACCCTACAGACC<br>CCTCTTCAGCCCCGTGGTGATGACTTTCCCCTGAG<br>GAAGCCCTGTAGCGTGCCTGGAGGAAGGGGCTCT<br>CCAACCCCAGCCCCACCTAGCCACCATGAACACT<br>TCAGCCCCACCTGCTGTCAGCCCCAACATCACCG<br>TCCTGGCACCAGGAAAGGGTCCCTGGCAAGTGGC<br>CTTCATTGGGATCACCACGGGCCTCCTGTCGCTAG<br>CCACAGTGACAGGCAACCTGCTGGTACTCATCTC<br>TTTCAAGGTCAACACGGAGCTCAAGACAGTCAAT<br>AA
	uORF10	adequate	AGGCAGTGGGGCTGA
	uORF11	strong	GTG（GUG）<br>GGGCTGAGGACCCTACAGACCCCTCTTCAGCCCC<br>GTGGTGATGACTTTCCCCTGAGGAAGCCCTGTAG<br>CGTGCCTGGAGGAAGGGGCTCTCCAACCCCAGCC<br>CCACCTAGCCACCATGAACACTTCAGCCCCACCT<br>GCTGTCAGCCCCAACATCACCGTCCTGGCACCAG<br>GAAAGGGTCCCTGGCAAGTGGCCTTCATTGGGAT<br>CACCACGGGCCTCCTGTCGCTAGCCACAGTGACA<br>GGCAACCTGCTGGTACTCATCTCTTTCAAGGTCAA<br>CACGGAGCTCAAGACAGTCAATAA

	uORF12	adequate	CTGAGGACCCTACAGACCCCTCTTCAGCCCCGTGGTGATGACTTTCCCCTGAGGAAGCCCTGTAGCGTGCCTGGAGGAAGGGGCTCTCCAACCCCAGCCCCACCTAGCCACCATGAACACTTCAGCCCCACCTGCTGTCAGCCCCAACATCACCGTCCTGGCACCAGGAAAGGGTCCCTGGCAAGTGGCCTTCATTGGGATCACCACGGGCCTCCTGTCGCTAGCCACAGTGACAGGCAACCTGCTGGTACTCATCTCTTTCAAGGTCAACACGGAGCTCAAGACAGTCAATAA
	uORF13	adequate	GTGGTGATGACTTTCCCCTGA
	uORF14	adequate	GTGATGACTTTCCCCTGA
	uORF15	adequate	ATGACTTTCCCCTGA
	uORF16	adequate	AAGCCCTGTAGCGTGCCTGGAGGAAGGGGCTCTCCAACCCCAGCCCCACCTAGCCACCATGAACACTTCAGCCCCACCTGCTGTCAGCCCCAACATCACCGTCCTGGCACCAGGAAAGGGTCCCTGGCAAGTGGCCTTCATTGGGATCACCACGGGCCTCCTGTCGCTAGCCACAGTGACAGGCAACCTGCTGGTACTCATCTCTTTCAAGGTCAACACGGAGCTCAAGACAGTCAATAA
	uORF17	adequate	CTGTAG
	uORF18	adequate	GTGCCTGGAGGAAGGGGCTCTCCAACCCCAGCCCCACCTAGCCACCATGAACACTTCAGCCCCACCTGCTGTCAGCCCCAACATCACCGTCCTGGCACCAGGAAAGGGTCCCTGGCAAGTGGCCTTCATTGGGATCACCACGGGCCTCCTGTCGCTAGCCACAGTGACAGGCAACCTGCTGGTACTCATCTCTTTCAAGGTCAACACGGAGCTCAAGACAGTCAATAA
	uORF19	adequate	CTGGAGGAAGGGGCTCTCCAACCCCAGCCCCACCTAGCCACCATGAACACTTCAGCCCCACCTGCTGTCAGCCCCAACATCACCGTCCTGGCACCAGGAAAGGGTCCCTGGCAAGTGGCCTTCATTGGGATCACCACGGGCCTCCTGTCGCTAGCCACAGTGACAGGCAACCTGCTGGTACTCATCTCTTTCAAGGTCAACACGGAGCTCAAGACAGTCAATAACTACTTCCTGCTGAGCCTGGCCTGTGCTGACCTCATCATCGGTACCTTCTCCATGAACCTCTATACCACGTACCTGCTCATGGGCCACTGGGCTCTGGGCACGCTGGCTTGTGACCTCTGGCTGGCCCTGGACTATGTGGCCAGCAATGCCTCCGTCATGAATCTGCTGCTCATCAGCTTTGACCGCT

			ACTTCTCCGTGACTCGGCCCCTGAGCTACCGTGCC AAGCGCACACCCCGCCGGGCAGCTCTGATGATCG GCCTGGCCTGGCTGGTTTCCTTTGTGCTCTGGGCC CCAGCCATCCTCTTCTGGCAGTACCTGGTAGGGG AGCGGACAGTGCTAGCTGGGCAGTGCTACATCCA GTTCCTCTCCCAGCCCATCATCACCTTTGGCACAG CCATGGCTGCCTTCTACCTCCCTGTCACAGTCATG TGCACGCTCTACTGGCGCATCTACCGGGAGACAG AGAACCGAGCACGGGAGCTGGCAGCCCTTCAGG GCTCCGAGACGCCAGGCAAAGGGGGTGGCAGCA GCAGCAGCTCAGAGAGGTCTCAGCCAGGGGCTGA GGGCTCACCAGAGACTCCTCCAGGCCGCTGCTGT CGCTGCTGCCGGGCCCCCAGGCTGCTGCAGGCCT ACAGCTGGAAGGAAGAAGAGGAAGAGGACGAAG GCTCCATGGAGTCCCTCACATCCTCAGAGGGAGA GGAGCCTGGCTCCGAAGTGGTGATCAAGATGCCA ATGGTGGACCCCGAGGCACAGGCCCCCACCAAGC AGCCCCCACGGAGCTCCCCAAATACAGTCAAGAG GCCGACTAAGAAAGGGCGTGATCGAGCTGGCAA GGGCCAGAAGCCCCGTGGAAAGGAGCAGCTGGC CAAGCGGAAGACCTTCTCGCTGGTCAAGGAGAAG AAGGCGGCTCGGACCCTGAGTGCCATCCTCCTGG CCTTCATCCTCACCTGGACACCGTACAACATCATG GTGCTGGTGTCCACCTTCTGCAAGGACTGTGTTCC CGAGACCCTGTGGGAGCTGGGCTACTGGCTGTGC TACGTCAACAGCACCATCAACCCCATGTGCTACG CACTCTGCAACAAAGCCTTCCGGGACACCTTTCG CCTGCTGCTGCTTTGCCGCTGGGACAAGAGACGC TGGCGCAAGATCCCCAAGCGCCCTGGCTCCGTGC ACCGCACTCCCTCCCGCCAATGCTGA
	uORF20	strong	AAGGGGCTCTCCAACCCCAGCCCCACCTAG
	uORF21	strong	AGGGGCTCTCCAACCCCAGCCCCACCTAGCCACC ATGAACACTTCAGCCCCACCTGCTGTCAGCCCCA ACATCACCGTCCTGGCACCAGGAAAGGGTCCCTG GCAAGTGGCCTTCATTGGGATCACCACGGGCCTC CTGTCGCTAGCCACAGTGACAGGCAACCTGCTGG TACTCATCTCTTTCAAGGTCAACACGGAGCTCAA GACAGTCAATAA
	uORF1	strong	GTGGGTGTGGGTGTTTGTCTCCCTGTATCCGCCTT CTCTCTCCTCTTGGACTTTTAG

**Mouse *Chrm1* 5'UTR$^{\triangle E2}$**	uORF2	strong	GTGGGTGTTTGTCTCCCTGTATCCGCCTTCTCTCTCCTCTTGGACTTTTAG
	uORF3	strong	GTGGGTGCCCTTGTGGACAAGGGGGGCACACTCCAGGACTTAGTCCAACCCCAGATGCTGCCTGAGGCTTCCCTCCTGCTCCCCCTCCCTTTCTTTCCTCCCTTTCCTCCCTCCCTCTCTTTCCCTTCCTCCCTCCCCCCCAAGGCTGGCGTGCCAGGGGGTGGGACATGCCAATCACTGGCTGTGCCTCTCCGGCTGCCAGCACAGGGCGCAGCTCCCCCCGGGAGCCAGGTGTTTGGGTCCCAGGAGACACTGCAGGCCCCCGGAGAAGCACTGAAGCTGAGGACCCTACAGACCCCTCTCCGGTCCCATGGTGATGCCCTTACTCTGAGAAAAATCTTGTGA
	uORF4	strong	AAGGGGGGCACACTCCAGGACTTAGTCCAACCCCAGATGCTGCCTGAGGCTTCCCTCCTGCTCCCCCTCCCTTTCTTTCCTCCCTTTCCTCCCTCCCTCTCTTTCCCTTCCTCCCTCCCCCCCAAGGCTGGCGTGCCAGGGGGTGGGACATGCCAATCACTGGCTGTGCCTCTCCGGCTGCCAGCACAGGGCGCAGCTCCCCCCGGGAGCCAGGTGTTTGGGTCCCAGGAGACACTGCAGGCCCCCGGAGAAGCACTGAAGCTGAGGACCCTACAGACCCCTCTCCGGTCCCATGGTGATGCCCTTACTCTGAGAAAAATCTTGTGA
	uORF5	strong	AGGGGGGCACACTCCAGGACTTAG
	uORF6	strong	CTGGCGTGCCAGGGGGTGGGACATGCCAATCACTGGCTGTGCCTCTCCGGCTGCCAGCACAGGGCGCAGCTCCCCCCGGGAGCCAGGTGTTTGGGTCCCAGGAGACACTGCAGGCCCCCGGAGAAGCACTGA
	uORF7	strong	AGGGGGTGGGACATGCCAATCACTGGCTGTGCCTCTCCGGCTGCCAGCACAGGGCGCAGCTCCCCCCGGGAGCCAGGTGTTTGGGTCCCAGGAGACACTGCAGGCCCCCGGAGAAGCACTGAAGCTGAGGACCCTACAGACCCCTCTCCGGTCCCATGGTGA
	uORF8	strong	GTGGGACATGCCAATCACTGGCTGTGCCTCTCCGGCTGCCAGCACAGGGCGCAGCTCCCCCCGGGAGCCAGGTGTTTGGGTCCCAGGAGACACTGCAGGCCCCCGGAGAAGCACTGA
	uORF9	strong	AAGGGGCTGCCCCTTCATTCCACCTGGCTGCAATGA

	uORF10	strong	AGGGGCTGCCCCTTCATTCCACCTGGCTGCAATG<br>AACACCTCAGTGCCCCCTGCTGTCAGTCCCAACA<br>TCACCGTCTTGGCACCAGGAAAGGGTCCCTGGCA<br>AGTGGCATTCATCGGGATCACCACAGGCCTCCTG<br>TCTCTGGCTACAGTGACAGGCAACCTGCTGGTGC<br>TCATCTCCTTCAAGGTCAACACAGAGCTCAAGAC<br>AGTCAACAACTACTTCCTGCTGAGCCTGGCCTGT<br>GCTGA
**Mouse *Chrm1* 5'UTR^{E2}**	uORF1	strong	GTGGGTGTGGGTGTTTGTCTCCCTGTATCCGCCTT<br>CTCTCTCCTCTTGGACTTTTAG
	uORF2	strong	GTGGGTGTTTGTCTCCCTGTATCCGCCTTCTCTCTC<br>CTCTTGGACTTTTAG
	uORF3	strong	GTGGGTGCCCTTGTGGACAAGGGGGGCACACTCC<br>AGGACTTAGTCCAACCCCAGATGCTGCCTGAGGC<br>TTCCCTCCTGCTCCCCCTCCCTTTCTTTCCTCCCTT<br>TCCTCCCTCCCTCTCTTTCCCTTCCTCCCTCCCCCC<br>CAAGGCTGGCGTGCCAGGGGGTGGGACATGCCA<br>ATCACTGGCTGTGCCTCTCCGGCTGCCAGCACAG<br>GGCGCAGCTCCCCCCGGGAGCCAGGTGTTTGGGT<br>CCCAGGAGACACTGCAGGCCCCCGGAGAAGCACT<br>GAAGCTGAGGACCCTACAGACCCCTCTCCGGTCC<br>CATGGAAACCCTGAATCCTACCTCCCCTATCCACC<br>TTCAGCCCCCCAAGATGGATTGAATGAGGCTGCT<br>GGCCAGATGGACAAGCTGGATGCCAAGGTGATGC<br>CCTTACTCTGA
	uORF4	strong	AAGGGGGGCACACTCCAGGACTTAGTCCAACCCC<br>AGATGCTGCCTGAGGCTTCCCTCCTGCTCCCCCTC<br>CCTTTCTTTCCTCCCTTTCCTCCCTCCCTCTCTTTC<br>CCTTCCTCCCTCCCCCCCAAGGCTGGCGTGCCAGG<br>GGGTGGGACATGCCAATCACTGGCTGTGCCTCTC<br>CGGCTGCCAGCACAGGGCGCAGCTCCCCCCGGGA<br>GCCAGGTGTTTGGGTCCCAGGAGACACTGCAGGC<br>CCCCGGAGAAGCACTGAAGCTGAGGACCCTACAG<br>ACCCCTCTCCGGTCCCATGGAAACCCTGAATCCT<br>ACCTCCCCTATCCACCTTCAGCCCCCCAAGATGG<br>ATTGAATGAGGCTGCTGGCCAGATGGACAAGCTG<br>GATGCCAAGGTGATGCCCTTACTCTGA
	uORF5	strong	AGGGGGGCACACTCCAGGACTTAG
	uORF6	strong	CTGGCGTGCCAGGGGGTGGGACATGCCAATCACT<br>GGCTGTGCCTCTCCGGCTGCCAGCACAGGGCGCA

			GCTCCCCCCGGGAGCCAGGTGTTTGGGTCCCAGGAGACACTGCAGGCCCCCGGAGAAGCACTGA
	uORF7	strong	AGGGGGTGGGACATGCCAATCACTGGCTGTGCCTCTCCGGCTGCCAGCACAGGGCGCAGCTCCCCCCGGGAGCCAGGTGTTTGGGTCCCAGGAGACACTGCAGGCCCCCGGAGAAGCACTGAAGCTGAGGACCCTACAGACCCCTCTCCGGTCCCATGGAAACCCTGA
	uORF8	strong	GTGGGACATGCCAATCACTGGCTGTGCCTCTCCGGCTGCCAGCACAGGGCGCAGCTCCCCCCGGGAGCCAGGTGTTTGGGTCCCAGGAGACACTGCAGGCCCCCGGAGAAGCACTGA
	uORF9	strong	ATGGATTGA
	uORF10	strong	CTGGATGCCAAGGTGATGCCCTTACTCTGA
	uORF11	strong	AAGGTGATGCCCTTACTCTGA
	uORF12	strong	AAGGGGCTGCCCCTTCATTCCACCTGGCTGCAATGA
	uORF13	strong	AGGGGCTGCCCCTTCATTCCACCTGGCTGCAATGAACACCTCAGTGCCCCCTGCTGTCAGTCCCAACATCACCGTCTTGGCACCAGGAAAGGGTCCCTGGCAAGTGGCATTCATCGGGATCACCACAGGCCTCCTGTCTCTGGCTACAGTGACAGGCAACCTGCTGGTGCTCATCTCCTTCAAGGTCAACACAGAGCTCAAGACAGTCAACAACTACTTCCTGCTGAGCCTGGCCTGTGCTGA

Supplementary Table 5. Mouse and human *CHRM1* 5'UTR sequences.

Name	Sequence
**Mouse *Chrm1* 5'UTR^{E2}**	GTGTGTGTGTGTGCCGCCAGTCTGTGTGCAGTGGGTGTGGGTGTTTGT<br>CTCCCTGTATCCGCCTTCTCTCTCCTCTTGGACTTTTAGCTTTCCTGGG<br>CTCTCTTTCCTCATGTCCTGCCTCCTCCCTCTCGAGGCTCAAAGTGGGT<br>GCCCTTGTGGACAAGGGGGGCACACTCCAGGACTTAGTCCAACCCCA<br>GATGCTGCCTGAGGCTTCCCTCCTGCTCCCCCTCCCTTTCTTTCCTCCC<br>TTTCCTCCCTCCCTCTCTTTCCCTTCCTCCCTCCCCCCCAAGGCTGGCG<br>TGCCAGGGGGTGGGACATGCCAATCACTGGCTGTGCCTCTCCGGCTG<br>CCAGCACAGGGCGCAGCTCCCCCCGGGAGCCAGGTGTTTGGGTCCCA<br>GGAGACACTGCAGGCCCCCGGAGAAGCACTGAAGCTGAGGACCCTA<br>CAGACCCCTCTCCGGTCCCATGGAAACCCTGAATCCTACCTCCCCTAT<br>CCACCTTCAGCCCCCCAAGATGGATTGAATGAGGCTGCTGGCCAGAT<br>GGACAAGCTGGATGCCAAGGTGATGCCCTTACTCTGAGAAAAATCTT<br>GTGACTGGAGGAAGGGGCTGCCCCTTCATTCCACCTGGCTGCA
**Mouse *Chrm1* 5'UTR$^{\triangle E2}$**	GTGTGTGTGTGTGCCGCCAGTCTGTGTGCAGTGGGTGTGGGTGTTTGT<br>CTCCCTGTATCCGCCTTCTCTCTCCTCTTGGACTTTTAGCTTTCCTGGG<br>CTCTCTTTCCTCATGTCCTGCCTCCTCCCTCTCGAGGCTCAAAGTGGGT<br>GCCCTTGTGGACAAGGGGGGCACACTCCAGGACTTAGTCCAACCCCA<br>GATGCTGCCTGAGGCTTCCCTCCTGCTCCCCCTCCCTTTCTTTCCTCCC<br>TTTCCTCCCTCCCTCTCTTTCCCTTCCTCCCTCCCCCCCAAGGCTGGCG<br>TGCCAGGGGGTGGGACATGCCAATCACTGGCTGTGCCTCTCCGGCTG<br>CCAGCACAGGGCGCAGCTCCCCCCGGGAGCCAGGTGTTTGGGTCCCA<br>GGAGACACTGCAGGCCCCCGGAGAAGCACTGAAGCTGAGGACCCTA<br>CAGACCCCTCTCCGGTCCCATGGTGATGCCCTTACTCTGAGAAAAATC<br>TTGTGACTGGAGGAAGGGGCTGCCCCTTCATTCCACCTGGCTGCA
**Human *CHRM1* 5'UTR**	ATGCCAATCACTGGCTGTGCCTCTCCCGCTGCCAGCACAGGGCGCAG<br>CTCCCCCTGGGAGCCAGGTGTTTGGGTCCCTGGAGACGCCGCAGGCC<br>CCCAGGGAGGCAGTGGGGCTGAGGACCCTACAGACCCCTCTTCAGCC<br>CCGTGGTGATGACTTTCCCCTGAGGAAGCCCTGTAGCGTGCCTGGAG<br>GAAGGGGCTCTCCAACCCCAGCCCCACCTAGCCACC

Supplementary Table 6. Mouse *Scgb1a1* promoter and *Chrm1* CDS for the generation of AAV9-Chrm1.

Name	Sequence
Mouse *Scgb1a1* promoter	TTAAAAGTTGGTTTGGAATTTGGAAATGGGAAAATATGTAAGGACAATCT CACTCTAGGACTCAAAGACAGAGATAAAATGTTCCTTACAAACCTATTCT GGGGGAACAACAGCATCAGAACTCTTTCTATATCAGCCTCTGCACTAGGG TCAGGCCAGAGACACAGAAATGAGCTAAGCCTACAATGAGAACGGAGGA ACCATGGGAATGTGCATCCTTAGGGCCCAGGGCCACTAGGAAAAGGCAA CCCAAATGCCAGGACTTTAAATCCCCTTCCTGTTGTGTCATTAGATGCAA ACAGCCCTGGAAAGGATGTGACCATAAGCAAGGCAAGGTTCTGTAGCGG GGGCAGATCCTGAAGTTACTAGAAGCTGAACGTTCTAGGCTGACCATGCT TCTCACAGTCACTAGATAGGATGCTCATGAAATGAACTCTTGAAATGCCT CTCCAGGTCTTCCCCAATTTCCAGTCCCACCAGCACCATAGTAGGGACTG GGCATCTATTGTCTGGGTGGATGGATGAACATTTGAGACAACCTGGAAGT TTGAATAGGATTTGTGGAAATGGAGAAGGTGTTGGTCCTAAGACACAAG AGCACTGAAGAAGTAAGAGTTAACACTAAACATGGCCAAGAGAAGCAAG GAGACTAAGGTAAGGCCTGGGAATGGCTAACACTTGAGAACTGTCAACA TCGTGAAAGAATAAGAAAGAATGACCAAGGAAAGAAAACAGGAAAGAG CTAAGCGTGGGAGAGTCCTGGAGAGAATGGAGCAAGAAGGGGGAGGTTA TAGGGTAAAGGCCAGGGAGAGGTCAGGTCAGATGAAGACTGATGGTGCT TTCAATTGGCAGGACTCAAGGGCTGCTCTGCGTAGGAACAGGCCAAGCCT GCCTGATCTAGGCCCTGGGTCTCTGATGTGTACTATGGAGAAGTCTTTCTA TGTTCACGTCTACTGTATGTAGGATCGAGCCTGTCTAACAATGCCCAAGA ATCGAGTGACCTTGTGGCTTGAAGTCTAGCCACGTTCGTTGGAGGGAGGC AATAGAAGGAGTCTAGTGACATCTCAGAGTCCTGTGTCTTTGTCCTTCCCT GTGATTCCTGAAGGGTCTCCGGCCTCTGGTTCTCCAGGGTTGGCAAGTCT ACAGTTGCTTCCTGGAACCTGGAGTGCTCAGTGCTTGACTTCCAAGAGAG GACACAGTTGTCTTCTACAGTTCCACGACCTCTGACTTGGGTCCTCCACTG CCTGAATACTCCACAAGTGGCCTATTGTGTGAGTGAGCTCAGTTTCAATG GGAACAGAAACTGGGTTTATGAAAAGAGATTATTTGCTTATTCCACGGAG AAGATGACCAAGTAAATAATGCAATCTCCTAAGTGGAGCGCAATCACTG CCCTCTACCTCTTGTGGGCTGCAAGGAACATATAAAAAGCCACACACCCA CACATA**[CCCACACATTACAACATCACCCCACATCTACAGACACCAAA GCCTCCAACCTCTACC]**
Mouse *Chrm1* CDS	**Kozak[GCCACC]ATG**AACACCTCAGTGCCCCCTGCTGTCAGTCCCAACATC ACCGTCTTGGCACCAGGAAAGGTCCCTGGCAAGTGGCATTCATCGGGAT CACCACAGGCCTCCTGTCTCTGGCTACAGTGACAGGCAACCTGCTGGTGC TCATCTCCTTCAAGGTCAACACAGAGCTCAAGACAGTCAACAACTACTTC CTGCTGAGCCTGGCCTGTGCTGATCTCATCATTGGCACTTTCTCCATGAAC

	CTCTATACCACATACCTGCTCATGGGCCACTGGGCTCTGGGCACACTGGC<br>CTGTGACCTCTGGCTGGCCCTGGACTATGTGGCCAGCAACGCCTCTGTCA<br>TGAATCTTCTGCTCATCAGCTTTGACCGTTACTTCTCAGTGACCCGACCCC<br>TGAGCTACCGAGCCAAGCGCACTCCCCGCAGGGCAGCTCTGATGATTGGC<br>CTCGCGTGGTTGGTTTCCTTCGTTCTCTGGGCCCCAGCCATCCTCTTCTGG<br>CAATACCTAGTTGGGGAGCGGACAGTGCTGGCTGGGCAGTGCTACATCCA<br>GTTCCTCTCCCAACCCATCATCACTTTTGGCACAGCCATGGCCGCCTTCTA<br>CCTCCCTGTCACAGTCATGTGTACGCTGTACTGGCGCATCTACCGGGAGA<br>CAGAAAACCGAGCCCGGGAGCTAGCAGCCCTACAGGGCTCTGAGACACC<br>AGGCAAAGGTGGTGGCAGCAGCAGCAGCTCAGAGAGGTCACAGCCAGGA<br>GCTGAAGGCTCACCCGAGTCACCTCCAGGCCGCTGCTGTCGCTGTTGCCG<br>GGCACCCAGACTTCTGCAGGCCTACAGCTGGAAAGAAGAAGAGGAAGAG<br>GATGAAGGCTCCATGGAGTCCCTCACATCCTCTGAAGGTGAGGAGCCTGG<br>CTCAGAAGTGGTGATCAAGATGCCTATGGTAGATCCTGAGGCACAGGCA<br>CCCACCAAGCAGCCTCCCAAAAGCTCCCCAAATACAGTCAAGAGGCCCA<br>CCAAGAAAGGCCGAGACCGAGGCGGCAAAGGCCAAAAACCCCGAGGGA<br>AGGAACAACTGGCCAAGAGAAAGACCTTCTCACTGGTCAAGGAGAAGAA<br>GGCAGCTCGGACCCTGAGTGCCATCCTGCTGGCCTTCATCCTCACCTGGA<br>CACCATATAACATCATGGTGCTGGTGTCTACATTCTGCAAGGACTGTGTT<br>CCAGAAACCCTATGGGAGCTGGGCTACTGGCTTTGCTACGTCAACAGCAC<br>TGTCAACCCCATGTGCTACGCACTCTGCAACAAAGCCTTCCGGGACACTT<br>TCCGCCTGCTGTTGCTCTGCCGCTGGGACAAGCGGCGCTGGCGCAAAATC<br>CCCAAGCGCCCTGGCTCTGTGCACCGCACCCCCTCCCGCCAATGC**3×Flag**<br>**[GACTACAAGGATGACGATGACAAGGATTACAAAGACGACGATGATA**<br>**AGGACTATAAGGATGATGACGACAAA]TAA**

Supplementary Table 7. Information of Human Sample.

Bronchoscopic mucosal biopsy tissues

Sample	Gender	Age (years)	FeNO (ppb)	Peripheral blood eosinophil count ($\times 10^9$/L)	Asthma
Ctrl-1	Female	59	13	0.21	N.A.
Ctrl-2	male	44	-	0.03	N.A.
Ctrl-3	Female	73	-	0	N.A.
Ctrl-4	Female	52	-	0.1	N.A.
Ctrl-5	male	61	-	0.06	N.A.
Ctrl-6	male	63	-	0.02	N.A.
Ctrl-7	male	48	6.9	0.06	N.A.
Asthma-1	Female	37	23.9	0.87	Yes
Asthma-2	Female	67	89	0.66	Yes
Asthma-3	male	68	12	0.56	Yes
Asthma-4	male	63	24.8	0.59	Yes
Asthma-5	male	60	-	0.33	Yes
Asthma-6	male	49	39	0.68	Yes
Asthma-7	Female	17	12	0.23	Yes

Bronchial epithelial cell brushing specimens

Sample	Gender	Age (years)	FeNO (ppb)	Peripheral blood eosinophil count ($\times 10^9$/L)	Asthma /COPD
Ctrl-1	male	70	-	0.11	N.A.
Ctrl-2	male	53	-	0.05	N.A.
Asthma-1	Female	58	-	0.11	Yes
Asthma-2	male	73	-	0.32	Yes
COPD	male	78	-	0.1	Yes

Individuals with or without ACOS lung biopsies

Sample	Gender	Age (years)	GOLD stage	COPD duration (years)	Asthma comorbidity	CAT score
ACOS-1	Female	58.8	IV	33	Yes	16
ACOS-2	Female	66.7	IV	11	Yes	31
ACOS-3	Male	60	III	6	Yes	27
ACOS-4	Male	75.1	III	8	Yes	15
ACOS-5	Male	74.3	IV	6	Yes	21
ACOS-6	Male	60.4	II	2	Yes	21
Ctrl-1	Female	63.4	N.A.	N.A.	N.A.	N.A.
Ctrl-2	Male	59.1	N.A.	N.A.	N.A.	N.A.
Ctrl-3	Male	62.3	N.A.	N.A.	N.A.	N.A.
Ctrl-4	Male	66.7	N.A.	N.A.	N.A.	N.A.
Ctrl-5	Male	78	N.A.	N.A.	N.A.	N.A.

CAT: COPD Assessment Test; -: not measured.

Supplementary Table 8. The primer sequences for PCR.

**Primer name**	**Sequence**
GAPDH (human)-F	5’-CGGAGTCAACGGATTTGGTC-3’
GAPDH (human)-R	5’-GACAAGCTTCCCGTTCTCAG-3’
ACTIN (human)-F	5’-CATGTACGTTGCTATCCAGGC-3’
ACTIN (human)-R	5’-CTCCTTAATGTCACGCACGAT-3’
TARDBP (human)-F	5'-ATCTGGTGTATGTTGTCAACTATCC-3'
TARDBP (human)-R	5'-GAACTTCTCCAAAGGTACTAAAATACTC-3'
UPF1 (human)-F	5'-AACGAGCACCAAGGCATTGGCT-3'
UPF1 (human)-R	5'-GGCTGCTTTGATAGTGCCTTCG-3'
CHRM1 (human)-F	5'-CTACAGACCCCTCTTCAGCC-3'
CHRM1 (human)-R	5'-TGATCCCAATGAAGGCCACT-3'
ATF4 (human)-F	5'-CCAAGCACTTCAAACCTCATG-3'
ATF4 (human)-R	5'-ATCCATTTTCTCCAACATCCAATC-3'
IFNB1 (human)-F	5’-GATGAACTTTGACATCCCTGAG-3’
IFNB1 (human)-R	5’-TCAACAATAGTCTCATTCCAGC-3’
IL33 (human)-F	5’-GTGACGGTGTTGATGGTAAGAT-3’
IL33 (human)-R	5’-AGCTCCACAGAGTGTTCCTTG-3’
TSLP (human)-F	5’-ATGTTCGCCATGAAAACTAAGGC-3’
TSLP (human)-R	5’-GCGACGCCACAATCCTTGTA-3’
Chrm1 (mouse)-primer set F	5'-GGGCACACTCCAGGACTTAG-3'
Chrm1 (mouse)-primer set2 R	5'-GGGGAGGTAGGATTCAGGGT-3'
Chrm1 (mouse)-primer set1 R	5'-CCAGGCTCAGCAGGAAGTAG-3'
Il33 (mouse)-F	5'-TCCAACTCCAAGATTTCCCCG-3'
Il33 (mouse)-R	5'-CATGCAGTAGACATGGCAGAA-3'
Tslp (mouse)-F	5'-ACGGATGGGGCTAACTTACAA-3'
Tslp (mouse)-R	5'-AGTCCTCGATTTGCTCGAACT-3'
Il25 (mouse)-F	5'-ACAGGGACTTGAATCGGGTC-3'
Il25 (mouse)-R	5'-TGGTAAAGTGGGACGGAGTTG-3'
Atf4 (mouse)-F	5'-CCTGAACAGCGAAGTGTTGG-3'
Atf4 (mouse)-R	5'-TGGAGAACCCATGAGGTTTCAA-3'
Tardbp (mouse)-F	5'-AATCAGGGTGGGTTTGGTAACA-3'
Tardbp (mouse)-R	5’-GCTGGGTTAATGCTAAAAGCAC-3’
NP-F	5’-TGTGTATGGACCTGCCGTAGC-3’
NP-R	5’-CCATCCACACCAGTTGACTCTTG-3’

Supplementary Table 9. Antibody information.

Antibody for Flow cytometry				
Name	Catalog number	Clone name	Suppliers	Dilution
AF700 anti-mouse CD45	103128	30-F11	BioLegend	1:200
BV711 anti-mouse CD3	100241	17A2	BioLegend	1:200
PE-Cy5 anti-mouse CD4	100410	GK1.5	BioLegend	1:200
FITC anti-mouse SiglecF	155504	S17007L	BioLegend	1:200
PerCP-Cy5.5 anti-mouse CD11b	101228	M1/70	BioLegend	1:200
APC anti-mouse F4/80	123116	BM8	BioLegend	1:200
BV650 anti-mouse Ly6G	108441	RB6-8C5	BioLegend	1:200
PE-Cy7 anti-mouse CD11c	117318	N418	BioLegend	1:200
BV605 anti-mouse IL-4	504126	11B11	BioLegend	1:200
APC anti-mouse IL-5	504306	TRFK5	BioLegend	1:200
PE anti-mouse IL-13	159403	W17010B	BioLegend	1:200
BV421 anti-mouse IFN-γ	505829	XMG1.2	BioLegend	1:200

Antibody for Immunofluorescence and immunohistochemical staining			
Name	Catalog number	Suppliers	Dilution
Anti-TDP-43	ab109535	Abcam	1:50
Anti-TDP-43	10782-2-AP	Proteintech	1:400
Anti-CHRM1	abs121129	Absin	1:200
Anti-Cc10	RD181022220-01	BioVendor	N/A
anti-Influenza A virus Nucleoprotein	GTX125989	GeneTex	1:300
anti-ATF4	10835-1-AP	Proteintech	1:100
anti-Acetylated Tubulin	T7451	Sigma-Aldrich	1:100
anti-E-cadherin	ET1607-75	HuaBio	1:200
anti-Flag	80801-2-RR	Proteintech	1:300

Antibody for Immunofluorescence and immunohistochemical staining			
Name	Catalog number	Suppliers	Dilution
anti-GAPDH	#5174S	CST	1:5000
Anti-TDP-43	10782-2-AP	Proteintech	1:2000
Anti-CHRM1	abs121129	Absin	1:1000
Anti-β-tubulin	M20005	Abmart	1:5000
anti-Influenza A virus Nucleoprotein	GTX125989	GeneTex	1:3000
anti-UPF1	66898-1-Ig	Proteintech	1:3000
anti-MX1	13750-1-AP	Proteintech	1:1000
anti-ATF4	10835-1-AP	Proteintech	1:2000

Supplementary Table 10. The fragment sequences of *UPF1* were inserted in pSpliceExpress vector.

***UPF1* sequence**
Intro 6:<br>CTCTCTCTTCCTCCCTCCCACAGCAGCTGCTCCCTGGCCACGGAGGCCTCTGGTGTGCGGT<br>GAAGCCTCCAGACGGGATTGGCCTGTGTGTGTTCCGGGGACTCCTAATGGCTTCTCTTTC<br>AGATGTGCCCTTGGTGGCATTTGGGGCTGCAGGTGCCACTCACGGCGCCTGCTCTCACGG<br>CAGAGGCTTGCTGTAGGGCCCGCCTCATGGGGCCTCGGGCATGTGGAGGCCAGGCCCGG<br>GCCTGTGCTGGAGGCTAACCGGGGCTCTTGTTTTTCATGTGCTCAG<br>Exon 7:<br>ACTCAAGATAACATCACTGTCAGGTGGGACCTGGGCCTTAACAAGAAGAGAATCGCCTA<br>CTTCACTTTGCCCAAGACTGACTCTGGTAATGAGGATTTAGTCATAATTTGGTTAAGAG<br>Intron 7:<br>GTGATTTTAAGTTTTAAAATATTTGTGACCATAAGTAGCATAAATTCCTAGTTCCACCCTT<br>GTAAAGTGCCCCTTAATTTGAACTCTCCCTGGTGGAAGCGACGGCGTGGGTTAAAATGGC<br>CACCTCTCTCACTTTTTTACCTCAAG

Uncropped scans of all blots in Supplementary Figures.

Supplementary Fig. 3

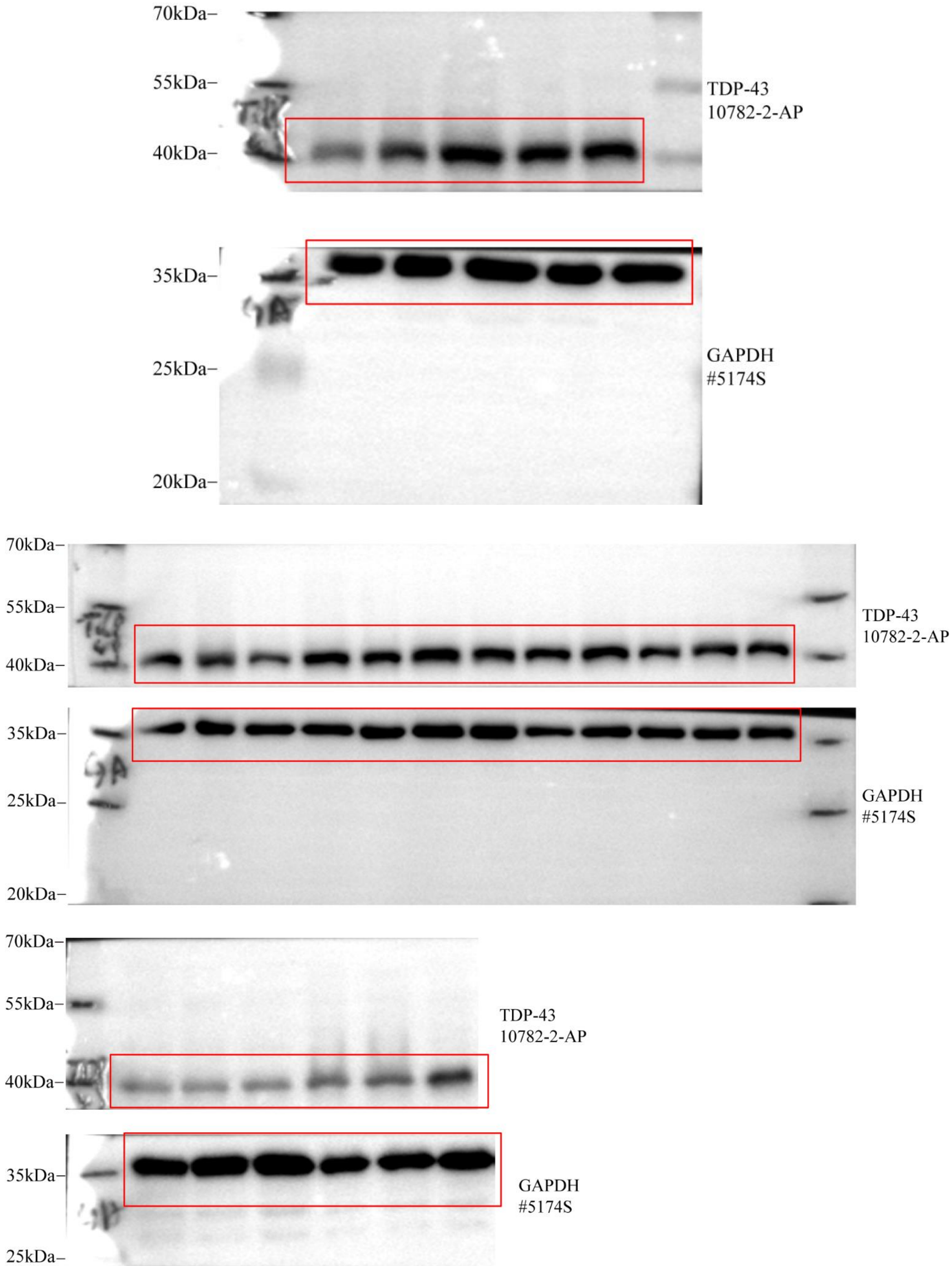

Supplementary Fig. 6

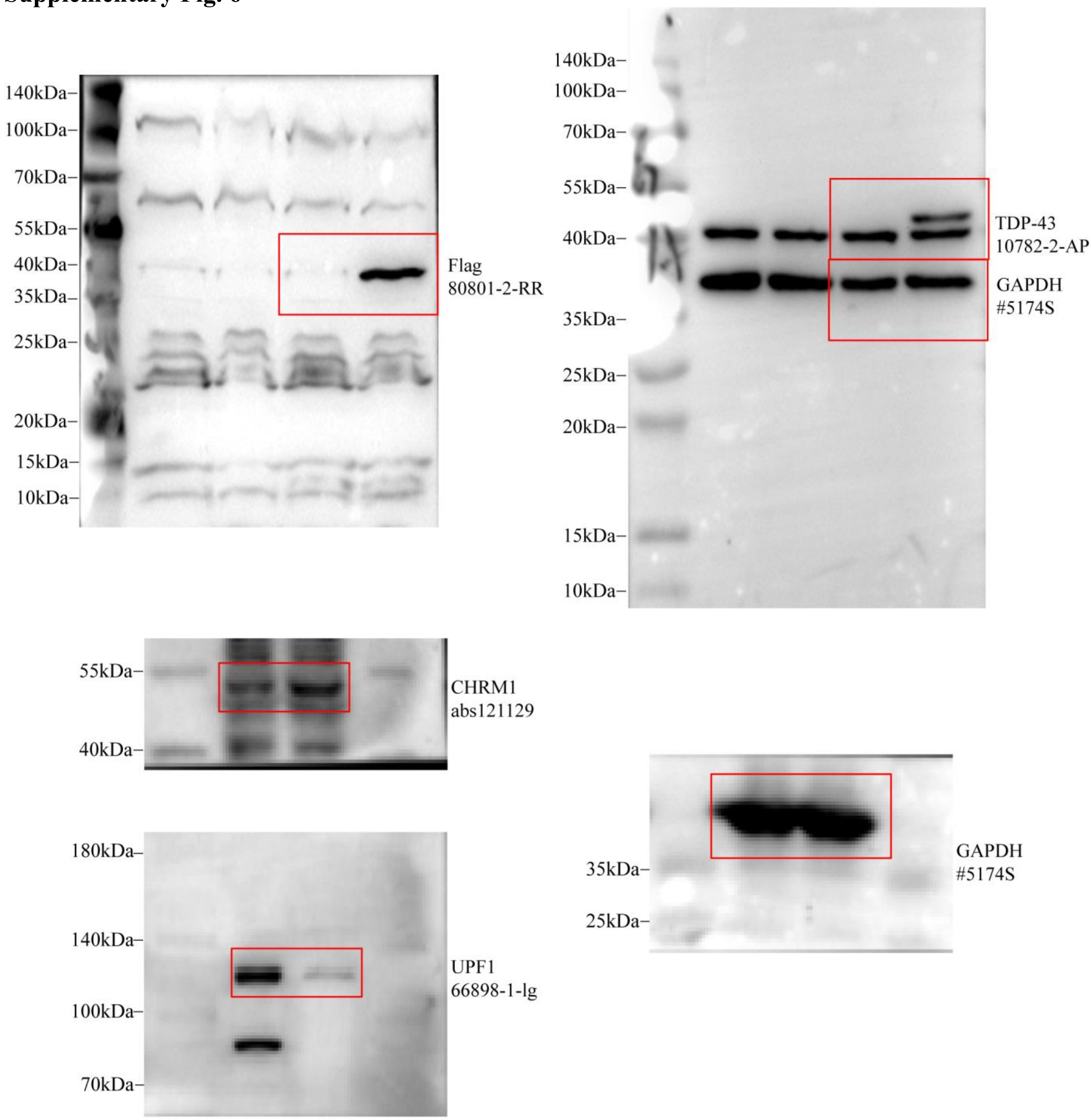

Supplementary Fig. 7

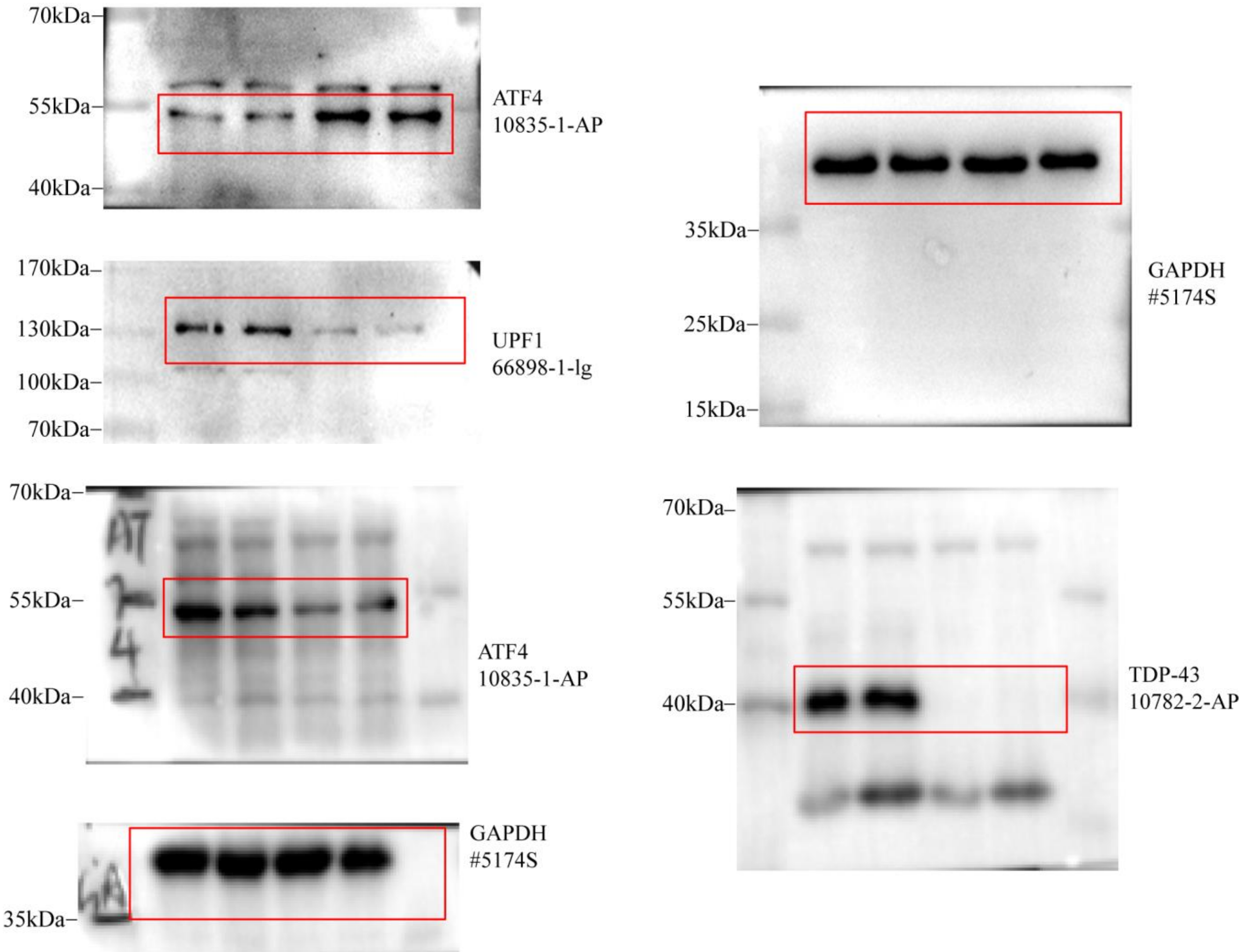

Supplementary Fig. 8

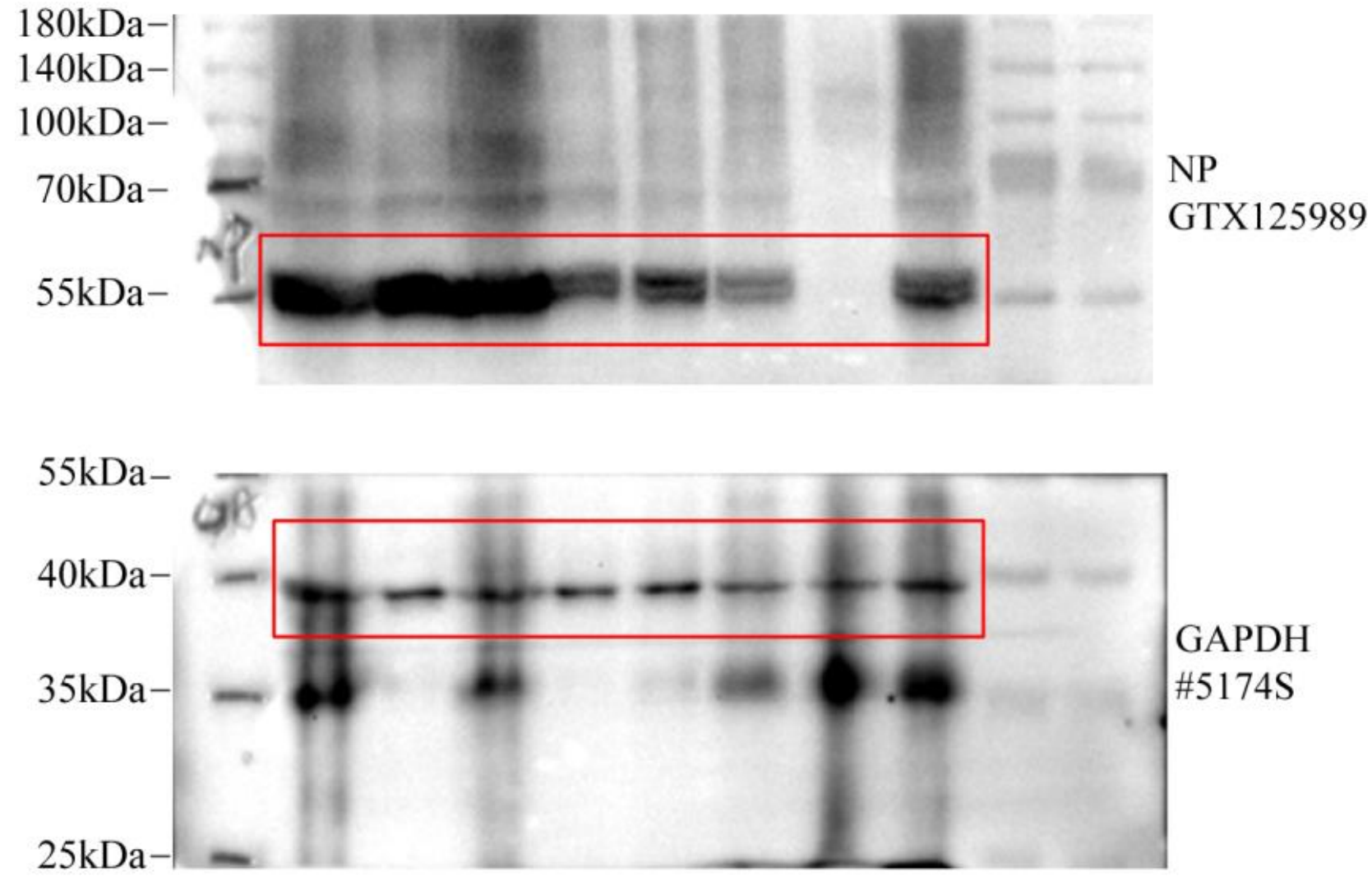

Supplementary Fig. 10

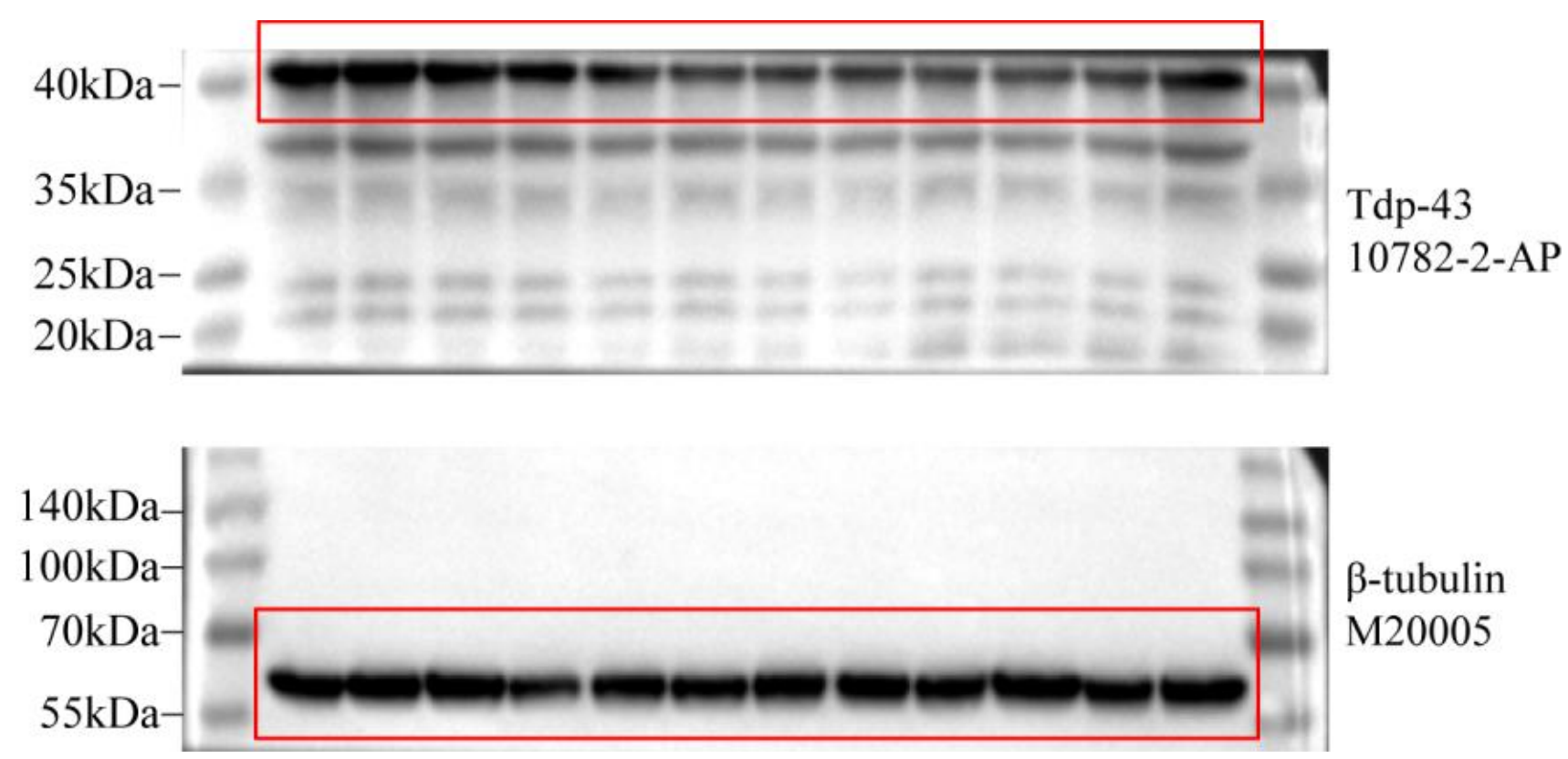

Supplement: Supplementary file 1 [file 41467_2026_75901_MOESM1_ESM.pdf]
