# Supplementary material for: TDP-43 promotes allergen-induced inflammation and influenza A virus replication in airway epithelium
Source: Nat Commun. 2026 Jul 23;17:8992. doi: 10.1038/s41467-026-75901-0 (PMC13507059; doi:10.1038/s41467-026-75901-0)
Supplement: Supplementary file 2 [file 41467_2026_75901_MOESM2_ESM.pdf]

nature portfolio

Corresponding author(s): Jun Wang

Last updated by author(s): July 5th, 2026

Reporting Summary

Nature Portfolio wishes to improve the reproducibility of the work that we publish. This form provides structure for consistency and transparency in reporting. For further information on Nature Portfolio policies, see our Editorial Policies and the Editorial Policy Checklist.

Statistics

For all statistical analyses, confirm that the following items are present in the figure legend, table legend, main text, or Methods section.

n/a	Confirmed	
☐	☒	The exact sample size (*n*) for each experimental group/condition, given as a discrete number and unit of measurement
☐	☒	A statement on whether measurements were taken from distinct samples or whether the same sample was measured repeatedly
☐	☒	The statistical test(s) used AND whether they are one- or two-sided<br>*Only common tests should be described solely by name; describe more complex techniques in the Methods section.*
☒	☐	A description of all covariates tested
☐	☒	A description of any assumptions or corrections, such as tests of normality and adjustment for multiple comparisons
☐	☒	A full description of the statistical parameters including central tendency (e.g. means) or other basic estimates (e.g. regression coefficient) AND variation (e.g. standard deviation) or associated estimates of uncertainty (e.g. confidence intervals)
☐	☒	For null hypothesis testing, the test statistic (e.g. *F*, *t*, *r*) with confidence intervals, effect sizes, degrees of freedom and *P* value noted<br>*Give P values as exact values whenever suitable.*
☒	☐	For Bayesian analysis, information on the choice of priors and Markov chain Monte Carlo settings
☒	☐	For hierarchical and complex designs, identification of the appropriate level for tests and full reporting of outcomes
☐	☒	Estimates of effect sizes (e.g. Cohen's *d*, Pearson's *r*), indicating how they were calculated

Our web collection on statistics for biologists contains articles on many of the points above.

Software and code

Policy information about availability of computer code

Data collection	Fluorescent images were obtained using Lecia TCS SP8 and flow cytometry data were collected using LSR Fortessa flow cytometer (BD). The Cryo-EM imaging was observed by Talos F200C G2 TEM. The biodistribution of LNP-ASO was imaged using an IVIS Lumina system (Perkin Elmer). real-time PCR data were collected using a Quantstudio 7 flex.
Data analysis	Statistical analyses were performed using Graphpad prism 8 software. The TissueQuest software and Image J were utilized to analyze mean fluorescence intensity for each slid. Flow cytometry data was analysed using FlowJo v10.8.1. The fluorescence intensity of tissues were quantified with the Living Image 4.4 software's region of interest (ROI) function.

For manuscripts utilizing custom algorithms or software that are central to the research but not yet described in published literature, software must be made available to editors and reviewers. We strongly encourage code deposition in a community repository (e.g. GitHub). See the Nature Portfolio guidelines for submitting code & software for further information.

Data

Policy information about availability of data

All manuscripts must include a data availability statement. This statement should provide the following information, where applicable:

- Accession codes, unique identifiers, or web links for publicly available datasets
- A description of any restrictions on data availability
- For clinical datasets or third party data, please ensure that the statement adheres to our policy

All data associated with this study are present in the paper or the Supplementary Materials. The raw RNA sequencing data generated in this study have been

deposited in the Genome Sequence Archive in National Genomics Data Center, China National Center for Bioinformation/Beijing Institute of Genomics, Chinese Academy of Science under accession code CRA028702 [https://ngdc.cncb.ac.cn/gsa/browse/CRA028702]. The raw numbers for charts and graphs are available in the Source Data file whenever possible. Source data are provided with this paper.

Research involving human participants, their data, or biological material

Policy information about studies with human participants or human data. See also policy information about sex, gender (identity/presentation), and sexual orientation and race, ethnicity and racism.

Reporting on sex and gender	Both male and female participants were included in this study without distinction, and data were analyzed without stratification by sex. Gender data for all human participants were compiled in Supplementary Table 7.
Reporting on race, ethnicity, or other socially relevant groupings	N/A
Population characteristics	N/A
Recruitment	Asthma patients and healthy controls were recruited by the Ningde Municipal Hospital, and the individuals with or without ACOS were recruited by the Guangzhou Women and Children's Medical Center.
Ethics oversight	All experimental procedures were reviewed and approved by the Ethics Review Committee.

Note that full information on the approval of the study protocol must also be provided in the manuscript.

Field-specific reporting

Please select the one below that is the best fit for your research. If you are not sure, read the appropriate sections before making your selection.

[x] Life sciences   [ ] Behavioural & social sciences   [ ] Ecological, evolutionary & environmental sciences

For a reference copy of the document with all sections, see nature.com/documents/nr-reporting-summary-flat.pdf

Life sciences study design

All studies must disclose on these points even when the disclosure is negative.

Sample size	Sample size was described in the legends of each figure. For in vivo studies, sample sizes of more than five mice per group were chosen to detect meaningful biological differences. For the majority of tissue staining analyses, a minimum of six samples were included in each group to guarantee the reliability of the findings. For qPCR assays, we included a minimum of three independent biological replicates per group.
Data exclusions	Outliers were excluded prior to statistical analysis.
Replication	Experiments described in the manuscript were performed at least twice and the number of experimental replicates is provided in the figure legends.
Randomization	For in vivo assays, mice were first grouped based on their genotypes. Subsequently, animals with the same genotype were randomly assigned to different treatment groups.
Blinding	The data collection and analysis of the key experiments including in vitro and in vivo treatments were done by a third investigator (blinding).

Reporting for specific materials, systems and methods

We require information from authors about some types of materials, experimental systems and methods used in many studies. Here, indicate whether each material, system or method listed is relevant to your study. If you are not sure if a list item applies to your research, read the appropriate section before selecting a response.

Materials & experimental systems

n/a	Involved in the study	
[ ]	[x]	Antibodies
[ ]	[x]	Eukaryotic cell lines
[x]	[ ]	Palaeontology and archaeology
[ ]	[x]	Animals and other organisms
[x]	[ ]	Clinical data
[x]	[ ]	Dual use research of concern
[x]	[ ]	Plants

Methods

n/a	Involved in the study	
[x]	[ ]	ChIP-seq
[ ]	[x]	Flow cytometry
[x]	[ ]	MRI-based neuroimaging

Antibodies

Antibodies used

Antibody for Flow cytometry

Name	Catalog number	Clone name	Suppliers	Dilution
AF700 anti-mouse CD45	103128	30-F11	BioLegend	1:200
BV711 anti-mouse CD3	100241	17A2	BioLegend	1:200
PE-Cy5 anti-mouse CD4	100410	GK1.5	BioLegend	1:200
FITC anti-mouse SiglecF	155504	S17007L	BioLegend	1:200
PerCP-Cy5.5 anti-mouse CD11b	101228	M1/70	BioLegend	1:200
APC anti-mouse F4/80	123116	BM8	BioLegend	1:200
BV650 anti-mouse Ly6G	108441	RB6-8C5	BioLegend	1:200
PE-Cy7 anti-mouse CD11c	117318	N418	BioLegend	1:200
BV605 anti-mouse IL-4	504126	11B11	BioLegend	1:200
APC anti-mouse IL-5	504306	TRFK5	BioLegend	1:200
PE anti-mouse IL-13	159403	W17010B	BioLegend	1:200
BV421 anti-mouse IFN-γ	505829	XMG1.2	BioLegend	1:200

Antibody for Immunofluorescence and immunohistochemical staining

Name	Catalog number	Suppliers	Dilution
Anti-TDP-43	ab109535	Abcam	1:50
Anti-TDP-43	10782-2-AP	Proteintech	1:400
Anti-CHRM1	abs121129	Absin	1:200
Anti-Cc10	RD181022220-01	BioVendor	
anti-Influenza A virus Nucleoprotein	GTX125989	GeneTex	1:300
anti-ATF4	10835-1-AP	Proteintech	1:100
anti-Acetylated Tubulin	T7451	Sigma-Aldrich	1:100
anti-E-cadherin	ET1607-75	HuaBio	1:200
anti-Flag	80801-2-RR	Proteintech	1:300

Antibody for western blot

Name	Catalog number	Suppliers	Dilution
anti-GAPDH	#5174S	Cell signaling technology	1:5000
Anti-TDP-43	10782-2-AP	Proteintech	1:2000
Anti-CHRM1	abs121129	Absin	1:1000
Anti-β-tubulin	M20005	Abmart	1:5000
anti-Influenza A virus Nucleoprotein	GTX125989	GeneTex	1:3000
anti-UPF1	66898-1-Ig	Proteintech	1:3000
anti-MX1	13750-1-AP	Proteintech	1:1000
anti-ATF4	10835-1-AP	Proteintech	1:2000

Validation

Antibodies were purchased from reputable manufacturers, and selected in part based on usage in peer-reviewed publications:
CD45
Komuczki J, et al. 2019. Immunity. 50:1289.;Saha D et al. 2017. Cancer cell. 32(2):253-267.
CD4
Palakurthi B, et al. 2023. Nat Commun. 14:2109. ; Schönberger K, et al. 2022. Cell Stem Cell. 29:131.
CD3
Xu W et al. 2019. Immunity. 50(4):1054-1068 .;Delacher M, et al. 2021. Immunity. 54(4):702-720.e17.
SiglecF
She L, et al. 2021. JCI Insight. 6:e143509.; Senatus L, et al. 2023. Commun Biol. 6:280.
CD11b
Li H, et al. 2022. Cancer Cell. 40:36.; Feng X, et al. 2023. Nat Commun. 14:3208.
F4/80
Amodio V, et al. 2023. Cancer Cell. 41:196. ; Czimmerer Z, et al. 2022. Immunity. 55:2006.
Ly6G
Brestoff JR, et al. 2021. Cell Metab. 33:270.; Mogilenko DA, et al. 2020. Immunity. 54(1):99-115.e12.
CD11c
Kedmi R, et al. 2022. Nature. 610:737.; Komuczki J, et al. 2019. Immunity. 50:1289.
IL-4
Fang D, et al. 2022. Immunity. 55:639.; Lorentsen KJ, et al. 2018. Nat Commun. 9:1679.
IL-5
Boothby IC, et al. 2021. Nature. 599:667. ;Li Q et al. 2018. Immunity. 48(2):258-270 .
IL-13
Glaubitz J, et al. 2022. Nat Commun. 13:4502. ;Fujimoto M, et al. 2022. Nat Commun. 13:5408.
IFN-γ
Ozga AJ, et al. 2022. Immunity. 55:82.; Kim E, et al. 2022. Immunity. 55:145.
TDP-43
Suitable for: ICC/IF, IHC-Fr, WB, IHC-P, Flow Cyt (Intra); Reacts with: Mouse, Rat, Human.
Zhao L et al. Nat Commun 11:341 (2020). TDP-43 facilitates milk lipid secretion by post-transcriptional regulation of Btn1a1 and Xdh.
Influenza A virus Nucleoprotein
Suitable for: WB, ICC/IF, IHC-P, IP, ELISA, Sandwich ELISA

AL Baker et al. Nature 2025; 637 (8047) : 913-920. Dairy cows inoculated with highly pathogenic avian influenza virus H5N1.
MX1
Suitable for: WB, IHC, IF/ICC, FC (Intra), IP, ELISA; Reacts with: human, mouse
Aaron R. Everitt et al. Nature. ; 484(7395): 519–523: IFITM3 restricts the morbidity and mortality associated with influenza.
Cc10
Wuetzler S et al. J Trauma Acute Care Surgery 73(4): 838–842 (2012). Clara cell protein16: A biomarker for detecting secondary respiratory complications in patients with multiple injuries.
anti-Acetylated Tubulin
Suitable for: quantitative dot blot, IF/ICC, WB, ELISA, RIA, electron microscopy; Reacts with: bovine, frog, invertebrates, human, hamster, mouse, protista, pig, monkey, chicken, rat, plant.
Caitlin Collins et al. Cell Rep. 2021 Aug 17;36(7):109556. Tubulin acetylation promotes penetrative capacity of cells undergoing radial intercalation.
ATF4
Suitable for: WB, IHC, IF, IP, CoIP, ChIP; Reacts with: human, mouse, rat.
Qiyi Zhao et al. Cell. 2020 Oct 1;183(1):76-93.e22. Targeting Mitochondria-Located circRNA SCAR Alleviates NASH via Reducing mROS Output.
UPF1
Suitable for: WB, IHC, IF/ICC, FC (Intra), ELISA; Reacts with: human, mouse, rat.
Liu Y et al. EMBO Rep. 2023 Jul 5;24(7):e56458. O-GlcNAcylation promotes topoisomerase IIα catalytic activity in breast cancer chemoresistance.
E-cadherin
Suitable for: WB, IF-Cell, IF-Tissue, IHC-P, FC, IP, mIHC; Reacts with: human.
Tao Huang et al. Adv Sci (Weinh). 2025 Sep;12(33):e06529.WTAP Mediated m6A Modification Stabilizes PDIA3P1 and Promotes Tumor Progression Driven by Histone Lactylation in Esophageal Squamous Cell Carcinoma.
Flag
Suitable for: WB, IHC, IF/ICC, FC (Intra), IP, CoIP, ELISA.
Shan Xu et al. FASEB J. 2024 Dec 15;38(23):e70213. The SLE-associated TREX1-P212fs mutation disrupts ER association leading to type I interferonopathy.

Eukaryotic cell lines

Policy information about cell lines and Sex and Gender in Research

Cell line source(s)	BEAS-2B and 293T cell lines were purchased from ATCC. The mouse bronchial epithelial cells and the primary Human Bronchial Epithelial cells (HBECs) were isolated from both sexes.
Authentication	The BEAS-2B and 293T cell line were authenticated using short tandem repeat (STR) profiling.
Mycoplasma contamination	All cell lines used in this study were tested for mycoplasma contamination and confirmed to be negative.
Commonly misidentified lines (See ICLAC register)	There is no commonly misidentified cell lines were used in our study.

Animals and other research organisms

Policy information about studies involving animals; ARRIVE guidelines recommended for reporting animal research, and Sex and Gender in Research

Laboratory animals	Tardbpflox mice (NM-CKO-2102528) and Scgb1a1-IRES-Cre mice (NM-KI-210120) were purchased from Shanghai Model Organisms Center, Inc., and airway epithelium specific Tardbp deficient (TardbpΔScgb1a1) mice were generated by breeding Scgb1a1-IRES-Cre mice with Tardbpflox mice. Male C57BL/6J mice (6–8 weeks old) were obtained from Rise Mice Biotechnology Co., Ltd for the development of an asthmatic mouse model.
Wild animals	No wild animals were used.
Reporting on sex	Both male and female participants were included in this study without distinction, and data were analyzed without stratification by sex. The findings are expected to be broadly applicable regardless of sex.
Field-collected samples	No field collected samples were used in the study.
Ethics oversight	All animal experiments were approved by the Animal Care and Use Committee of Shanghai Yishang Biotechnology Co., Ltd with Biosafety Level 2 (BSL-2) facilities (Approval no. IACUC-2023-Mi-209).

Note that full information on the approval of the study protocol must also be provided in the manuscript.

Plants

Seed stocks	N/A
Novel plant genotypes	N/A
Authentication	N/A

Flow Cytometry

Plots

Confirm that:

- [x] The axis labels state the marker and fluorochrome used (e.g. CD4-FITC).
- [x] The axis scales are clearly visible. Include numbers along axes only for bottom left plot of group (a 'group' is an analysis of identical markers).
- [x] All plots are contour plots with outliers or pseudocolor plots.
- [x] A numerical value for number of cells or percentage (with statistics) is provided.

Methodology

Sample preparation

The lung tissues of mice were cut with scissors and transferred to 5 ml centrifuge tube, addition with 0.5 mg ml-1 collagenase A to digest in a shaking table at 200 rpm for 1 h at 37 ℃. The digested tissue was filtered by a 70 μm screen and centrifuged at 500 rcf for 5 min at 4 ℃. 2 ml ACK was added to the cells in the bottom of the EP tube to crack the red blood cells for 5 min, followed by addition of 10 ml PBS to terminate the cleavage. After centrifugation again, the supernatant was discarded to obtain cell precipitation for use.

For BALF and lung single-cell suspension myeloid flow panel, cells were incubated with the flow antibodies to anti-mouse CD45 (103128, BioLegend), CD3 (100241, BioLegend), CD4 (100456, BioLegend), SiglecF (155504, BioLegend), CD11b (101263, BioLegend), F4/80 (123116, BioLegend),Ly6G (127645, BioLegend), CD19 (115530, BioLegend), CD11c (117318, BioLegend) and I-A/I-E (107639, BioLegend) for 30 min at 4 ℃ for surface staining.

For Th2 flow panel, the lung single-cell suspension was stimulated with PMA (50 ng ml-1), ionomycin (1 μg ml-1), and BFA (1x dilution) in 500-1000 μl complete RPMI-1640 medium (Gibco) supplemented with10% FBS and penicillin/streptomycin and incubated at 37 ℃ for 4 h prior to staining. Then the cells were incubated with the flow antibodies to anti-mouse CD45 (103128, BioLegend), CD3 (100241, BioLegend), CD4 (317408, BioLegend), CD8 (140416, BioLegend) for 30 min at 4 ℃ for surface staining. Subsequently, dead cells were stained with Zombie Aqua™ Fixable Viability Kit (423101, BioLegend) in all flow panels. For intracellular staining, the cells were treated with Cyto-Fast™ Fix/Perm Buffer Set (426803, BioLegend) and stained with anti-mouse IL-4 (504126, BioLegend), IL-5 (504306, BioLegend), IL-13 (12-7133-82, eBioscience), IFN-γ (505829, BioLegend).

Instrument	Cells were analyzed on a BD LSR Fortessa instrument.
Software	BD LSR Fortessa software (BD Biosciences) was used to collect the data, and the data was analyzed by FlowJo v10.6.2 software.
Cell population abundance	The purity of cells after sorting was confirmed by post-sort analyses and was usually > 90%.
Gating strategy	Gating strategies were described in Supplementary Fig. 1 and 9.

- [x] Tick this box to confirm that a figure exemplifying the gating strategy is provided in the Supplementary Information.
