# Supplementary material for: TDP-43 promotes allergen-induced inflammation and influenza A virus replication in airway epithelium
Source: Nat Commun. 2026 Jul 23;17:8992. doi: 10.1038/s41467-026-75901-0 (PMC13507059; doi:10.1038/s41467-026-75901-0)
Supplement: Supplementary file 3 [file 41467_2026_75901_MOESM3_ESM.pdf]

nature portfolio

Peer Review File

TDP-43 promotes allergen-induced inflammation and influenza A virus replication in airway epithelium

Corresponding Author: Professor Jun Wang

This file contains all reviewer reports in order by version, followed by all author rebuttals in order by version.

Version 0:

Reviewer comments:

Reviewer #1

(Remarks to the Author)
This manuscript by Yuan and colleagues identifies the expression of TDP-43, a regulator of RNA metabolism, particularly nonsense-mediated RNA decay (NMD), can be increased in airway epithelial cells by allergen challenge to the airways. This is a noteworthy finding as TDP-43 has not been associated with asthma and NMD has been speculated to play a role in asthma pathogenesis. Linking aberrant RNA quality control to asthma is a novel concept, whereas aberrant alternative RNA splicing has been described in COPD. The authors show further convincingly in three allergen asthma mouse models (OVA, dust mite and cockroach sensitization) that the decreased UPF1-mediated NMD leads to accumulation of the acetylcholine receptor CHRM1 that is involved in inflammatory epithelial cell responses. Further, they show that mucosal administration of lipid nanoparticles containing TDP-43 antisense can dampen allergic asthma responses in the mice. They further also show a role of TDP-43 in facilitation replication of influenza A. This is relevant as influenza can trigger severe asthma exacerbations, although influenza is not the most common respiratory virus that triggers asthma.

The methodology for the animal studies is sound and the methods are detailed to allow reproduction of the experiments. However, the data for human airway epithelial cells is less specific for asthma.

The are several points that should be addressed:

- The data on the human epithelial cells is limited to BEAS-2B cells. No ALI cultures were used to characterize the expression of TDP-43.

- It would be helpful if the RNAseq dataset of human airway mucosa from allergic individuals exposed to allergen is described in more detail.

- In the introduction non-T2 asthma is identified as a target. However the animal models used are allergic models TDP-43 is only significantly induced by the eosinophilic models OVA and HDM, but not by the neutrophilic cockroach model.

- Figure 1 F would benefit from staining controls (epithelial cell marker, isotype controls for TDP-43 mouse and human tissue.

- The human data is on increased TDP-43 expression associated with asthma is only shown for lung samples from patients with asthma-COPD overlap syndrome and not asthma alone.

Reviewer #2

(Remarks to the Author)
Yuan and colleagues have performed a series of experiments in transgenic mice and the human bronchial epithelial cell line BEAS2B. This is linked to published transcriptomic data from a human allergen challenge clinical study to assess a role for TDP-43 in asthma pathogenesis. There is also an assessment of the role of TDP-43 in influenza virus infection.
Background
Two or the 3 epithelial alarmins are mentioned (IL-33 and TSLP) – why not also mention IL-25?

In transcripts from asthma patients, specific AS events in related to T2 cytokines have been reported, including IL-33ΔE3&4, IL-4ΔE2 and IL-5ΔE2 16-18, which are closely associated with asthma severity . What does closely associated to asthma severity mean – closely is subjective and how was asthma severity measured?
Not clear why you think TDP-43 is potentially involved..? How do you speculate the preceding evidence in neurodegenerative diseases leads to question in asthma specifically? Do you have a hypothesis related to role of TDP-43 in asthma?
Here we reported that allergen-induced TDP-43 expression in AECs drives asthma pathogenesis by inducing UPF1-mediated NMD impairment, leading to accumulation of pathogenic transcripts containing upstream open reading frames (uORFs) in their 5' untranslated regions (5'UTRs) and long 3'UTRs, such as the transcripts encoding cholinergic receptor muscarinic 1 (CHRM1), whose expression on AECs plays essential role in asthmatic response through recognition of the vagal neurotransmitter acetylcholine (Ach).
This long sentence ties together disparate findings and in doing so loses accuracy. There is no data on asthma pathogenesis - and what does asthma pathogenesis mean? Inception of disease, progression, exacerbation frequency, disease severity, responsiveness to therapy etc this can only be assessed by longitudinal clinical study and cannot be deduced from mouse experiments.
General comment for background – the description of asthma is incomplete and inaccurate in places. Asthma is a clinical umbrella term covering multiple endotypes and when referring to pathogenesis this should be acknowledged and specific pathogenic mechanisms identified.

Results
Figure 1 What allergic individuals? Do they have asthma - this is not clear. What allergen are they sensitive to/challenged with? Were all subjects exposed to the same allergen? What is meant by pathogenic asthma state?
Why were 3 different models initially assessed and the OVA only examined further - could argue that OVA is least clinically informative. OVA is a 'clean' model of type 2 allergic inflammation – allergic airways disease.
To investigate the expression patterns of RQC-related genes, we performed full-length transcript sequencing (smart-seq2) with purified AECs from OVA-induced asthmatic mice . Gene Set Enrichment Analysis (GSEA) results revealed that AECs from asthmatic mice had increased expression of genes enriched in asthma Such as…? Were they consistent with allergen challenged people?
AECs from asthmatic mice showed increased expression of genes involved in asthmatic response, such as Mucl3, Ccl22, Rnase2a, Retnla, etc . These are not commonly reported 'asthma signature' genes reported in humans. This reinforces that mice do not have asthma and AECs from mice acutely sensitised and challenged with allergen will not recapitulate the intrinsic disease phenotype of AECs from adult asthmatics. As a result the analysis of mouse AECs in this context is of limited value here.
Fig 1J/K the data is not convincing – the images for healthy vs ACOS do not look comparable to me.
Fig 2 The data generated from silenced TARDBP in cell line BEAS-2B do NOT indicate that TDP-43 plays a crucial role in mediating inflammatory responses in AECs. This is a non-differentiated cell line so provides little insight in AEC biology.
Characterises TDP-43 knockout mice - comparing parental (flox), heterozygous ko (HE) and homozygous ko (HO). Please indicate how the data was confirmed – repeat experiments, in the figure legend. The description of the mouse data is very superficial – what day post allergen challenge is assessed?
Figure 3 To gain mechanistic insights of TDP-43 involvement in asthma pathogenesis, we performed smart-seq2-seq on AECs isolated from TardbpΔScgb1a1 and Tardbpflox mice under OVA-induced asthmatic state
This approach does not give insight into asthma pathogenesis – this will give insight into the role of TDP-43 in AEC acute response to allergen. The data is this figure is qualitative so difficult to discern the value.
Figure 4 The link between TDP-43 and CHRM1 mRNA expression is interesting. At a molecular level in this synthetic system it is a compelling narrative relevant to asthma. However caution is needed with over interpreting the mechanistic link to asthma in the absence of any data that actually does so.
Figure 5 I wonder why influenza was chosen as rhinovirus is the most common cause of asthma exacerbations. The BEAS-2B data is limited – a timecourse of virus replication would be more informative – these are cell lines so presumably you are not limited by cell number. As such the data related to control of viral replication and anti-viral interferon is very limited.
Regarding mouse influenza infection model – again a timecourse tracking lung viral load would be more informative. As it is the only data to support the conclusion is NP immunofluorescence (which is quantified) at a single timepoint.
Figure 6 The title of this section really highlights misunderstanding of asthma - application of ASO-LNP targeting TDP-43 ameliorates asthmatic symptoms
There is no assessment of anything related to symptoms. LNP ASO is delivered intranasally. This is done during the allergen sensitisation and challenge phase of the experiment – so cannot conclude if the treatment is preventing sensitisation or response to allergen in established sensitisation. This relevant to the research question – do the authors hypothesises that TDP-43 plays a role in asthma development, or allergen induced inflammation in established disease?

Reviewer #3

(Remarks to the Author)
This manuscript identifies TDP-43 as a novel epithelial-intrinsic regulator of asthma pathogenesis via suppression of nonsense-mediated decay (NMD). The authors propose that allergen-induced TDP-43 expression in airway epithelial cells inhibits UPF1-mediated RNA quality control, leading to accumulation of pathogenic transcripts such as CHRM1 and promoting airway inflammation. They further show that TDP-43 facilitates influenza A virus (IAV) replication, and that intranasal delivery of antisense oligonucleotides (ASO) encapsulated in lipid nanoparticles (LNPs) reduces asthma-like pathology in mice.
The study is mechanistically intriguing, linking RNA surveillance to airway mucosal biology. However, several key

mechanistic and interpretive issues need to be addressed.

Major Comments
1. Mechanistic link between TDP-43 and UPF1 repression is not established. Please provide direct evidence of regulation (e.g., RIP-qPCR, ChIP, or promoter assay).
2. Specificity of airway epithelial TDP-43 deletion needs further validation. Validation with FoxJ1-Cre (ciliated cell–specific) or Krt5-CreER (basal) models would strengthen the claim that epithelial TDP-43 is the key pathogenic driver. Or Single-cell RNA-seq demonstrating TDP-43 ablation across epithelial subsets would also be convincing.
3. The AAV–Chrm1 rescue experiment may not be epithelium-specific. Quantify transgene expression in airway vs non-airway tissues and test pharmacologic antagonists.
4. Link between epithelial TDP-43/CHRM1 and Th2 activation is unclear. Examine whether TDP-43 or CHRM1 alters epithelial alarmins (IL-33, TSLP, IL-25) that drive Th2/ILC2 responses.
5. NMD dysregulation beyond CHRM1 is not explored. Analyze other NMD-sensitive transcripts to broaden mechanistic insight.
6. Analyze other NMD-sensitive transcripts to broaden mechanistic insight.
7. Discussion lacks mechanistic integration. Expand on how TDP-43–NMD suppression fits within epithelial stress or immune-regulatory pathways.
8. Broader context could be strengthened. Consider linking epithelial TDP-43 biology to its known functions in neurodegeneration or systemic RNA surveillance.

Minor Comments
• The Abstract slightly overstates therapeutic claims; suggest changing to “ameliorated asthma-like pathology in mice.”
• Indicate sex and age of animals in all experiments.
• Specify statistical tests and adjustments for multiple comparisons.
• Include verification of Cre recombination efficiency and Tardbp deletion at the RNA level.

Version 1:

Reviewer comments:

Reviewer #1

(Remarks to the Author)
The authors have addressed the issues that were identified in the prior review and have added supporting data and information to the revised manuscript.

Minor comment:
It should be noted that the data with the human epithelial cells was done with BEAS-2b cells and not with epithelial cells differentiated at ALI.

Reviewer #2

(Remarks to the Author)
I would like to thank the authors for the additional work undertaken and data provided to address this reviewer's questions. For me there does remain several outstanding concerns:
Response figure 5 (updated figure 1) – the representative image does not appear to be relate to the quantification. What is ‘5 independent biological repeats’? You should indicate how many donors and how many sections/fields per section were analyzed.
I am not convinced (in the absence of studying this process using differentiated BECs from patients with asthma) allergen induced tissue perturbations – unclear what this means. The primary driver of airway pathology is allergen induced inflammation caused by T2 immunity. Direct epithelial damage by allergen is a less important secondary response and relies on inherent abnormality of asthmatic airway epithelial cells (otherwise everyone would be susceptible to allergen induced tissue perturbation).
Only some allergens (with protease activity for example) have been documented to directly damage epithelium – and this is mostly in mouse models with high level of allergen exposure. OVA as allergen is used to study T2 immunity in mouse models of allergic airways disease. OVA does not possess protease activity and does not damage epithelium. Therefore the use of OVA to understand how allergen induced epithelial damage drives airway inflammation is not appropriate.
The authors state “Importantly, in both CA and OVA mouse models, we consistently observed that Tdp-43 inhibition alleviated asthmatic airway response”. I think this data undermines the main conclusion. OVA does not exhibit protease activity, and it is fundamentally different from protease containing allergens such as house dust mite (HDM; e.g., Der p 1) or cockroach allergens.
Fundamental to supporting the conclusions of this research is the use of ALI-differentiated BECs from patients with asthma. Using mouse ALI-BECs treated with IL-13 does not recapitulate the inherent abnormality of asthmatic BECs necessary to recapitulate the allergen-induced tissue perturbations and signaling pathway central the hypothesis:
Allergen exposure triggered tissue perturbations cellular stress response in AECs dysregulated TDP-43 expression compromised NMD activity asthmatic airway inflammation, this study aims to investigate the potential role of TDP 43 dysregulation in allergen induced airway asthmatic inflammatory response.

Figure 6c – I see no evidence of infection and replication of IAV – there is no increase (above input) of NP – this data does not add anything to the main conclusions of the study. Similarly the assessment of influenza infection and replication time-course in vivo is qualitative (IF). The response states significantly low NP in KO mice. A quantitative measure of viral load (qPCR, infectivity) would be more convincing.

Reviewer #3

(Remarks to the Author)
The authors have adequately addressed all of my concerns. Specifically, the addition of scRNA-seq and mechanistic data (RIP-qPCR/minigene assay) has significantly strengthened the manuscript. I have no further comments and recommend the paper for publication.

Version 2:

Reviewer comments:

Reviewer #2

(Remarks to the Author)
I thank the authors for the substantial additional data. All outstanding concerns have been addressed.

Open Access This Peer Review File is licensed under a Creative Commons Attribution 4.0 International License, which permits use, sharing, adaptation, distribution and reproduction in any medium or format, as long as you give appropriate credit to the original author(s) and the source, provide a link to the Creative Commons license, and indicate if changes were made.
In cases where reviewers are anonymous, credit should be given to 'Anonymous Referee' and the source.
The images or other third party material in this Peer Review File are included in the article’s Creative Commons license, unless indicated otherwise in a credit line to the material. If material is not included in the article’s Creative Commons license and your intended use is not permitted by statutory regulation or exceeds the permitted use, you will need to obtain permission directly from the copyright holder.
To view a copy of this license, visit https://creativecommons.org/licenses/by/4.0/

REVIEWER COMMENTS

Response: We are grateful to the reviewers for their constructive comments and thoughtful suggestions. The comments were very helpful as we revised and improved our manuscript. Based on these comments and suggestions, we have made careful modifications to the original manuscript.

Reviewer #1 (Remarks to the Author):

This manuscript by Yuan and colleagues identifies the expression of TDP-43, a regulator of RNA metabolism, particularly nonsense-mediated RNA decay (NMD), can be increased in airway epithelial cells by allergen challenge to the airways. This is a noteworthy finding as TDP-43 has not been associated with asthma and NMD has been speculated to play a role in asthma pathogenesis. Linking aberrant RNA quality control to asthma is a novel concept, whereas aberrant alternative RNA splicing has been described in COPD. The authors show further convincingly in three allergen asthma mouse models (OVA, dust mite and cockroach sensitization) that the decreased UPF1-mediated NMD leads to accumulation of the acetylcholine receptor CHRM1 that is involved in inflammatory epithelial cell responses. Further, they show that mucosal administration of lipid nanoparticles containing TDP-43 antisense can dampen allergic asthma responses in the mice.

They further also show a role of TDP-43 in facilitation replication of influenza A. This is relevant as influenza can trigger severe asthma exacerbations, although influenza is not the most common respiratory virus that triggers asthma.

The methodology for the animal studies is sound and the methods are detailed to allow reproduction of the experiments. However, the data for human airway epithelial cells is less specific for asthma.

Response: We are grateful to the reviewer for their encouraging comments on the significance of our study, as well as the insightful recommendations for enhancing it.

The are several points that should be addressed:

- The data on the human epithelial cells is limited to BEAS-2B cells. No ALI cultures were used to characterize the expression of TDP-43.

Response: Thanks for the constructive advice. As your suggestion, we isolated murine primary airway epithelial cells (AECs) and established Air-Liquid Interface (ALI) cultures. Following differentiation, these ALI cultures were subjected to Il13 stimulation to recapitulate hallmark features of an asthmatic airway response.

As the result shown in Response Figure 1: S2f, the control (CTRL) group exhibited well-differentiated ciliated cells, as was also evidenced by strong immunofluorescence (IF) staining for Acetylated α-Tubulin (Ac-Tubulin), a marker for ciliated cells. In contrast, the Il13 stimulated group showed significantly enhanced mucus secretion, as demonstrated by Periodic Acid-Schiff (PAS) staining. These results confirmed the successful differentiation of our mouse primary airway epithelial cells.

Using ALI cultures, we obtained following experimental results:

- We performed IF staining for Tdp-43 on Il13-treated ALIs. The results indicated a higher expression level of Tdp-43 in the Il13 treated group compared to control group (Response Figure 1: S2g, h).
- In Il13-challenged ALIs derived from *Tardbp*$^{\Delta Scgb1a1}$ mice, expressions of alarmin genes were significantly reduced compared with those from *Tardbp*^{flox} mice (Response Figure 1: 3i).
- We treated ALI-cultured cells with cockroach allergen (CA) and, in parallel, blocked transcription with actinomycin D for varying durations. The results showed that the stability of *Upf1* transcripts was comparatively declined in the CA group (Response Figure 1: 4n).
- In addition, we also found that *Chrm1* expression was markedly decreased in Il13-challenged ALIs derived from *Tardbp*$^{\Delta Scgb1a1}$ mice compared to ALIs derived from *Tardbp*^{flox} mice (Response Figure 1: 5f).

Response Figure 1

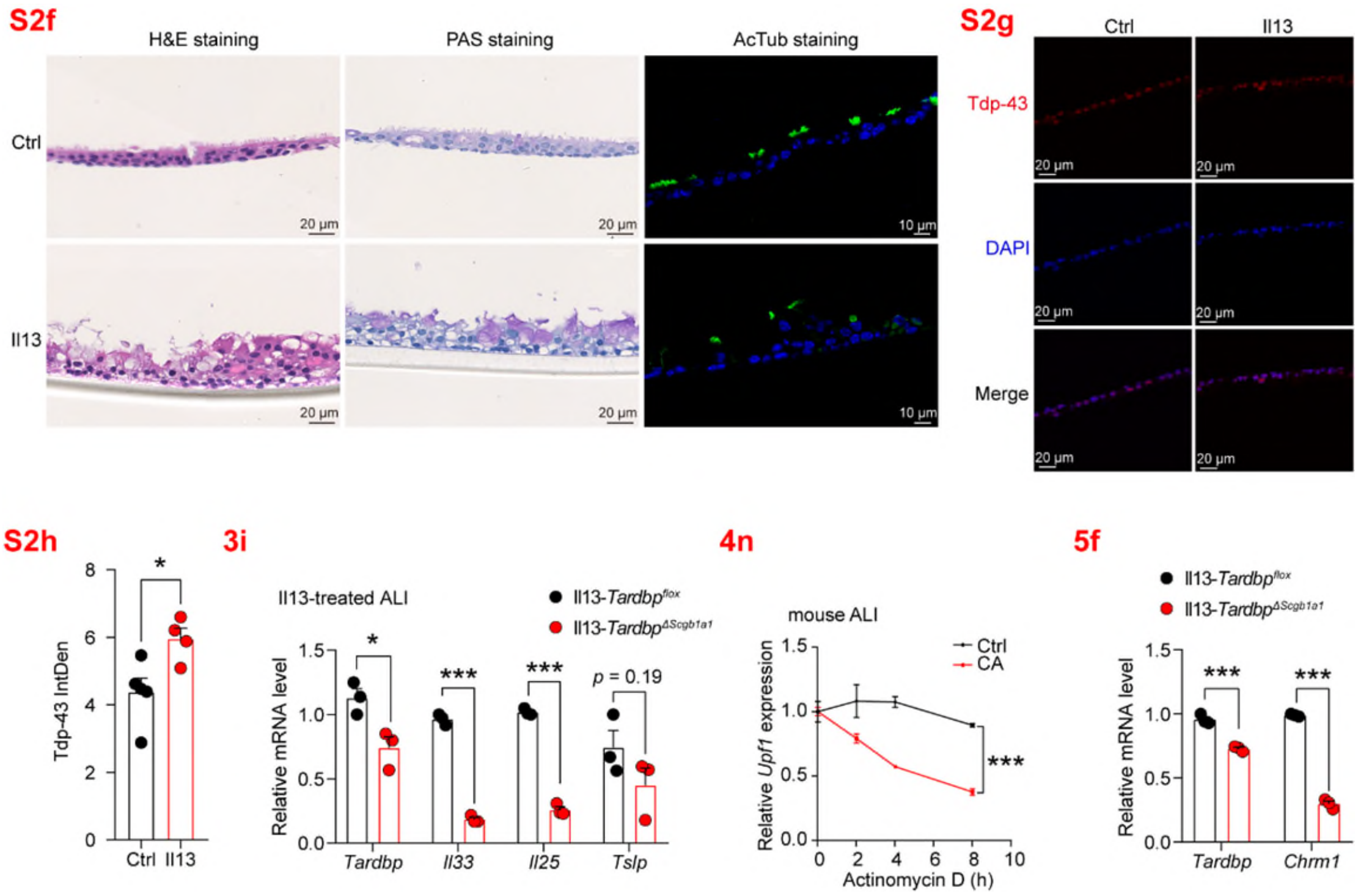

Response Figure 1 | **S2f**, Representative images of H&E staining, PAS staining and AcTub IF staining in Il13-stimulated and control AECs under ALI conditions. **S2g,** Representative IF images of Tdp-43 in Il13-stimulated and control AECs under air-liquid interface conditions. **S2h,** Quantitative IF results of Tdp-43 in (**S2g**). 3**i,** mRNA levels of *Tardbp*, *Il33*, *Il25* and *Tslp* in primary AECs isolated from CA-induced *Tardbp*$^{\Delta Scgb1a1}$ and *Tardbp*^{flox} mice and differentiated under air-liquid interface conditions. **4n,** mRNA expression of *Upf1* in allergen (CA)-induced and control AECs under air-liquid interface conditions, followed by treated with 10 μg/ml Actinomycin D for 2 h, 4 h and 8 h. **5f,** mRNA level of *Chrm1* and *Tardbp* in in primary AECs isolated from CA-induced *Tardbp*$^{\Delta Scgb1a1}$ and *Tardbp*^{flox} mice and differentiated under air-liquid interface conditions.

- It would be helpful if the RNAseq dataset of human airway mucosa from allergic

individuals exposed to allergen is described in more detail.

Response: Thank you for this insightful comment, which is valuable for highlighting the clinical relevance of our study.

As you suggested, we gave more details about the scRNA-seq dataset of human airway mucosa from allergic individuals as below:

Participant characteristics:

	Allergic controls (**AC**)	Allergic Asthmatics (**AA**)
History of asthma	×	✓
Allergic symptoms to HDM and/or cat	✓	✓
Skin prick test (**SPT**)	Positive	Positive
Lung function	Baseline FEV_1 and FVC normal	Baseline FEV1≥75% predicted
Methacholine challenge	Negative	Positive

The same stock of allergen was used for both SPT and segmental allergen challenge (SAC): standardized **HDM** [Dermatophagoides pteronyssinus; Greer Laboratories, 10,000 allergy units (AU) per ml] and/or **cat hair extract** [Felis catis; Greer Laboratories, 10,000 bioequivalent allergy units (BAU) per ml].

For the SAC procedures:

1) Diluent (**Dil**) was then administered to the anterior segment of the right upper lobe followed by allergen (**Ag**) to the lateral segment of the right middle lobe.

2) 24 hours later, endobronchial brush samples were obtained from the Dil- and Ag-challenged segmental airways for scRNA-seq.

Two subpopulations of ciliated cells (Cili) were identified (Response Figure 2: S1a) and the mucous Cili subpopulation highly expressed *IL33*, *SCGB3A1*, *MUC5AC* and *SCGB1A1* (Response Figure 2: S1b). To infer the potential involvement of RQC in allergen-induced asthmatic response, we inquired a RQC signature based on RNA splicing and surveillance-related genes in ciliated cells. Among both allergic controls (AC) and allergic asthmatics (AA) subjects, Cili and mucous Cili with allergen exposure (Ag) displayed substantially decreased RQC scores compared to that of diluent exposure (Dil) (Response Figure 2: 1a). Of note, compared to Dil-Cili, Ag-Cili under-expressed genes encoding components of NMD machinery (*UPF1*, *UPF2*, *UPF3B*, *SMG6* and *SMG1*) in both AC and AA individuals (Response Figure 2: 1b). However, accumulated expressions of NMD targets were only observed in Ag-Cili from AA subjects (Response Figure 2: 1b).

These results suggest that AECs from allergic asthmatics may possess compromised NMD activity.

Response Figure 2

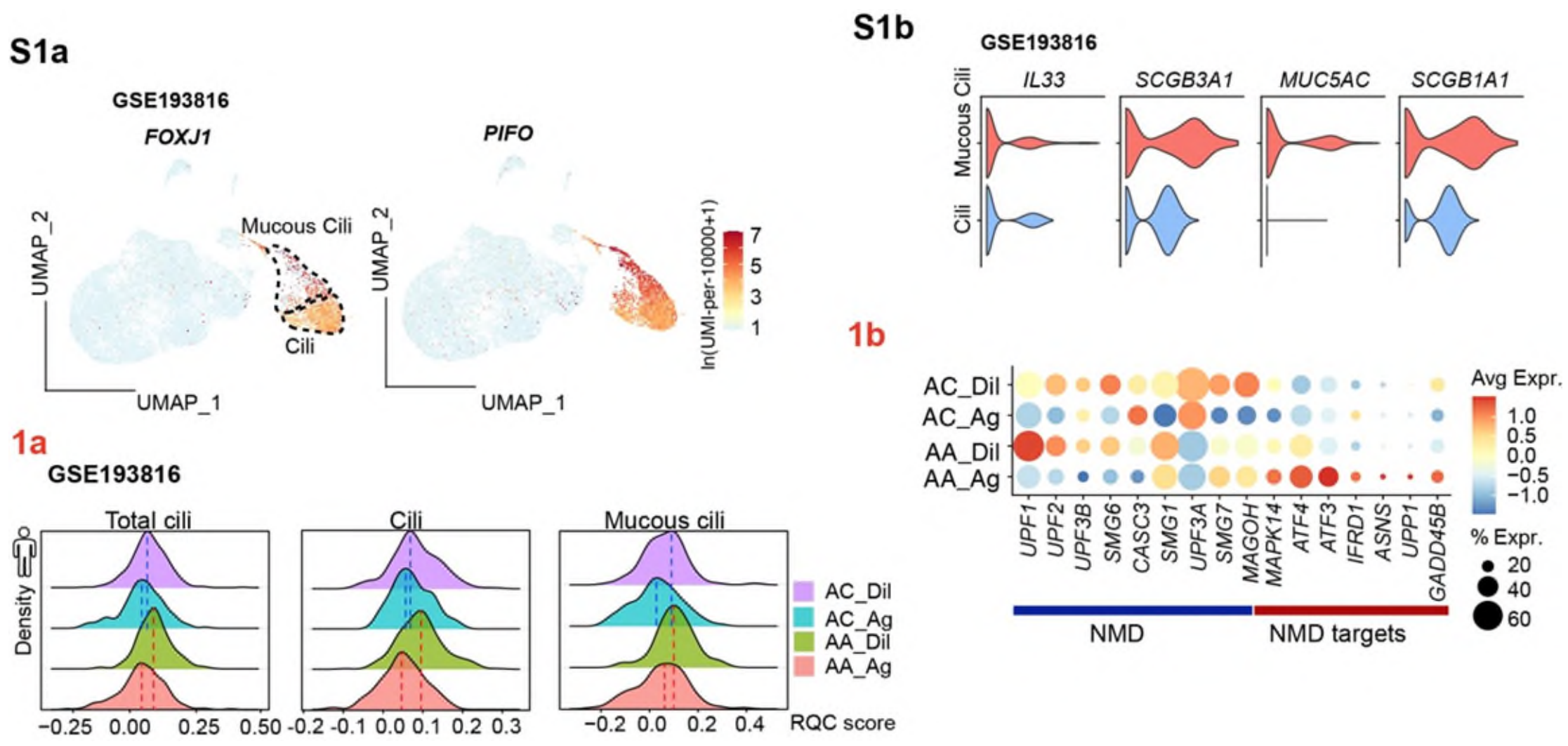

Response Figure 2 | **S1a**, UMAP plots showing marker gene expressions of ciliated cells in scRNA-seq dataset of airway mucosa from allergic asthmatic individuals under allergen or diluent exposure (GSE193816). Color intensity was proportional to its normalized unique molecular identifier (UMI). **S1b**, Violin plots showing differential gene expressions between mucous Cili and Cili subpopulation. **1a**, Histogram plots comparing the RQC signature scores in ciliated cells from subjects with exposure of allergen or diluent (GSE193816). **1b**, Dot plots comparing expressions of NMD-related genes and NMD targets across ciliated cell subpopulations from subjects with allergen or diluent exposure. Ag: allergen, Dil: diluent.

- In the introduction non-T2 asthma is identified as a target. However the animal models used are allergic models TDP-43 is only significantly induced by the eosinophilic models OVA and HDM, but not by the neutrophilic cockroach model.

Response: Thanks for the constructive comment. In the initial submission, the airway Tdp-43 upregulation was obvious but not significant in the cockroach antigen (CA) induced asthmatic mice, potentially due to small sample size and high intra-group

variation. In the revised manuscript, we validated whether CA could significantly induce airway Tdp-43 expression in a new batch of mice with CA-induced asthma. Quantitative IF analysis of Tdp-43 in lung tissues from six mice per group (CTRL and CA) now confirms its significant elevation in the CA group compared to CTRL group (Response Figure 3: 1g,h). In parallel, we isolated primary AECs from CA-treated and control mice. The results from both western blot and IF assays collectively demonstrated that Tdp-43 expression was significantly elevated in the CA group compared to control (Response Figure 3: 1i, S2d-e).

Does airway epithelial-specific knockout of Tdp-43 have a therapeutic effect in CA-induced neutrophilic asthma? In the revised manuscript, we observed that *Tardbp*$^{\Delta Scgb1a1}$ mice manifested significantly alleviated pulmonary pathologies, which was dose-dependently correlated to the extent of TDP-43 loss (Response Figure 3: 3a). Flow cytometric results revealed profoundly reduced Th2 infiltrations in the lungs and neutrophil infiltrations in both lungs and BALF from HO-*Tardbp*$^{\Delta Scgb1a1}$ mice with CA exposure (Response Figure 3: 3b,c,e,f).

We administered the Tdp-43 targeting LNP-ASO to a CA-induced non-T2 asthmatic mouse model to further validate its therapeutic efficacy. The results unambiguously demonstrated the LNP-ASO group exhibited a marked reduction in pulmonary pathologies and immune cell infiltration, particularly neutrophils (Response Figure 3: 7i,j).

Collectively, our findings revealed that *Tardbp*$^{\Delta Scgb1a1}$ mice were hyposensitive to either CA-induced neutrophilic (non-T2) or OVA-induced T2 eosinophilic (T2)

asthmatic modeling. The OVA model was employed more extensively in our study due to its broader application in murine asthma modeling[18, 19, 20].

Response Figure 3

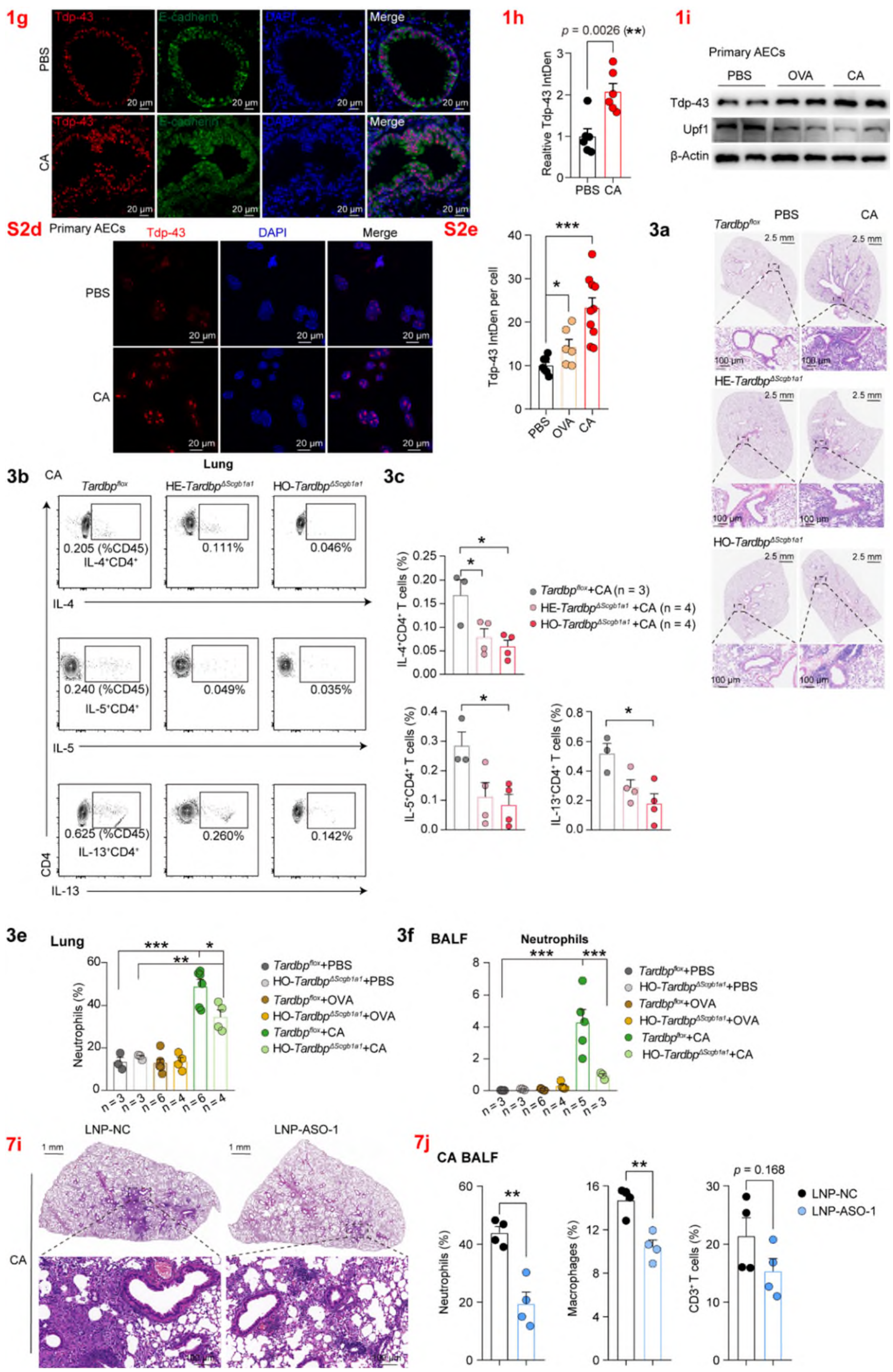

Response Figure 3 | **1g,** Representative IF images of Tdp-43 in the lungs from CA-induced asthmatic mice and control. **1h,** Quantitative IF results of Tdp-43 in (**1g**). **1i,** immunoblotting of Tdp-43 and Upf1 in the AECs isolated from CA/OVA-induced mice and control mice. **S2d**, Representative IF images of Tdp-43 in the AECs isolated from CA induced mice and control mice. **S2e**, Quantitative IF results of TDP-43 in (**S2d**). **3a**, H&E staining comparing pathologies between CA-induced $\textit{Tardbp}^{\Delta Scgb1a1}$ and $\textit{Tardbp}^{flox}$ mice. **3b**, Representative flow plots showing pulmonary infiltrations of Th2 across CA-induced HO-$\textit{Tardbp}^{\Delta Scgb1a1}$, HE-$\textit{Tardbp}^{\Delta Scgb1a1}$ and $\textit{Tardbp}^{flox}$ mice. **3c**, Bar plots summarizing the flow cytometric results in (**3b**). **3e** and **3f**, Bar plots showing flow cytometric results of neutrophil infiltrations in the lung (**3e**) and BALF specimens (**3f**) from $\textit{Tardbp}^{\Delta Scgb1a1}$ and $\textit{Tardbp}^{flox}$ mice. **7i**, H&E staining of pulmonary pathologies in CA-induced mice receiving i.n. LNP-ASO-1 intervention and control. **7j**, Bar plots showing flow cytometric results of immune cell infiltrations in the BALF of CA-induced mice receiving i.n. LNP-ASO-1 intervention and control.

- Figure 1 F would benefit from staining controls (epithelial cell marker, isotype controls for TDP-43 mouse and human tissue.

Response: We are grateful to the reviewer for the constructive comment. As suggested, we performed co-localization staining of Tdp-43 and E-Cadherin (a marker for epithelial cells) in mouse and human tissues, with IgG serving as a negative control for Tdp-43 staining. The results showed that Tdp-43 is primarily expressed in E-Cadherin positive cells (Response Figure 4: S2b).

Response Figure 4

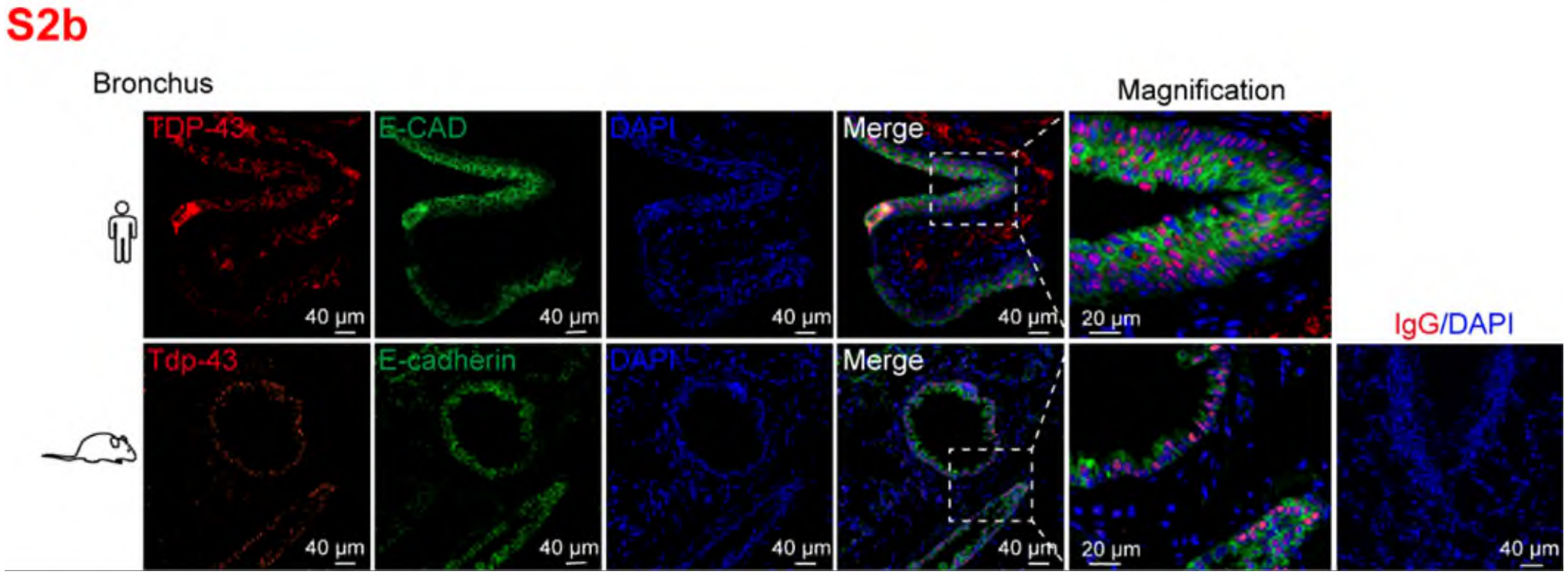

Response Figure 4 | **S2b**, Representative IF images of Tdp-43 and E-Cadherin in the lungs from C57BL/6J mice and bronchoscopic mucosal specimens from healthy subjects.

- The human data is on increased TDP-43 expression associated with asthma is only shown for lung samples from patients with asthma-COPD overlap syndrome and not asthma alone.

Response: We appreciate the reviewer's constructive feedback. We recollected bronchoscopic mucosal specimens from patients with asthma (without comorbidity of COPD) and relatively healthy subjects, followed by IF co-staining of TDP-43 and E-Cadherin. Quantitative analysis of TDP-43 expression within E-Cadherin-positive regions revealed a significantly higher level of TDP-43 in the airway epithelium of patients with asthma compared to the control group (Response Figure 5: 1j, k).

Response Figure 5

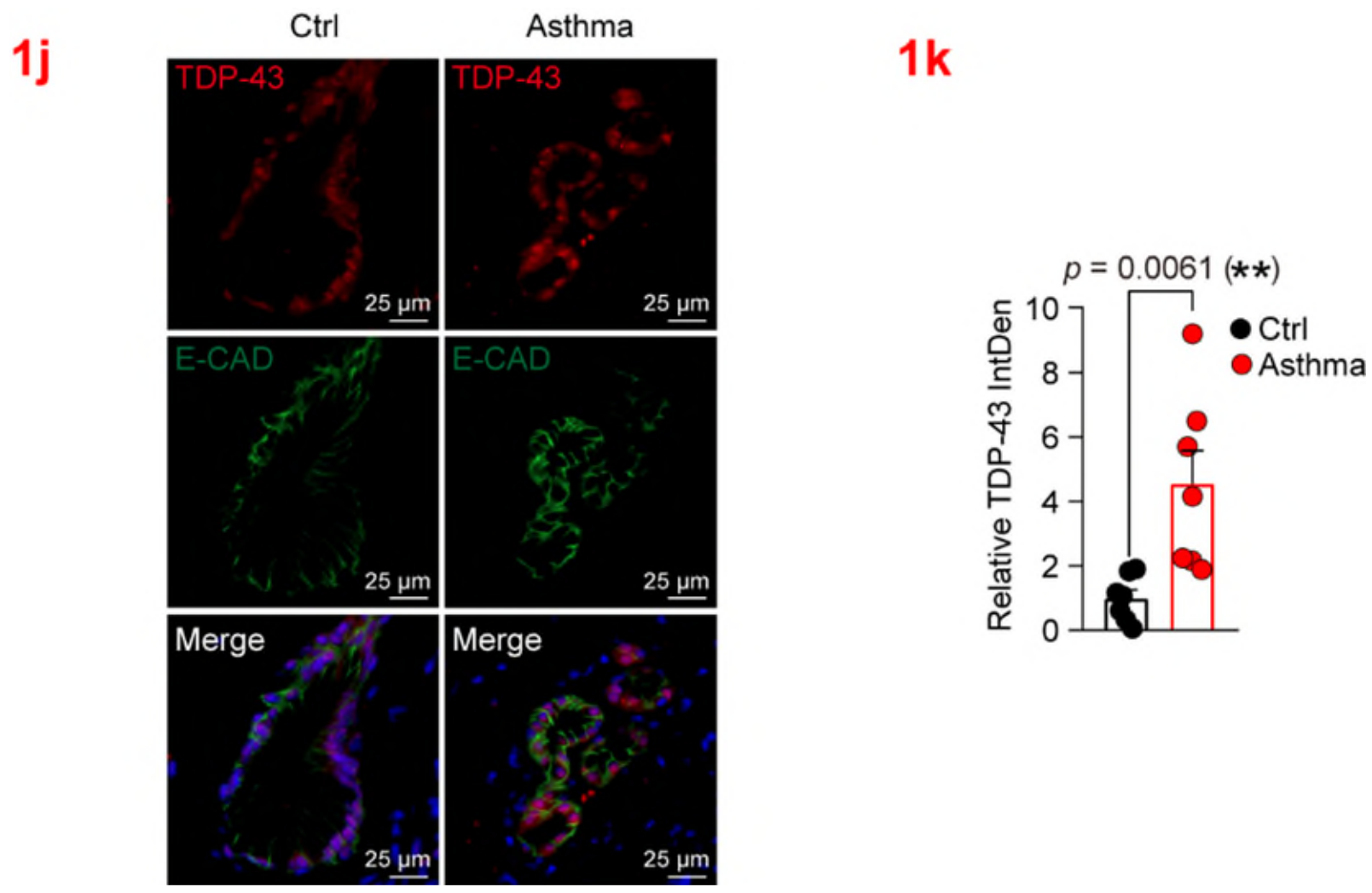

Response Figure 5 | **1j**, Representative IF staining of TDP-43 and E-cadherin in the bronchoscopic mucosal specimens from patients with asthma and relatively healthy subjects. **1k**, Quantitative IF results of TDP-43 within E-Cadherin-positive regions in (**1j**).

Reviewer #2 (Remarks to the Author):

Yuan and colleagues have performed a series of experiments in transgenic mice and the human bronchial epithelial cell line BEAS2B. This is linked to published transcriptomic data from a human allergen challenge clinical study to assess a role for TDP-43 in asthma pathogenesis. There is also an assessment of the role of TDP-43 in influenza virus infection.

Response: We would like to express our sincere gratitude to Reviewer #2 for the insightful and constructive comments.

Background

Two or the 3 epithelial alarmins are mentioned (IL-33 and TSLP)-why not also mention IL-25?

Response: Thank you for the comment. IL-25 is also an important alarmin, alongside IL-33 and TSLP. As suggested, we have included IL-25 in the **Introduction** accordingly.

In transcripts from asthma patients, specific AS events in related to T2 cytokines have been reported, including $\text{IL-33}^{\Delta E3\&4}$, $\text{IL-4}^{\Delta E2}$ and $\text{IL-5}^{\Delta E2}$ 16-18, which are closely associated with asthma severity. What does closely associated to asthma severity mean – closely is subjective and how was asthma severity measured?

Response: Thank you for the constructive comment. It would be more precise to say that these AS events of IL-33, IL-4 and IL-5 in asthmatic individuals were correlated with T2 inflammations[34, 35, 36].

In the revised manuscript, we have removed these citations from the **Introduction**, as their connection to NMD is tenuous. This adjustment was made to streamline the narrative, which now focuses on how allergen challenge triggers tissue perturbations, leading to cellular stress and subsequent NMD impairment, before introducing dysregulated TDP-43 expression as a potential sensor of such stress that may drive NMD dysregulation and contribute to asthmatic airway inflammation.

Hope these modifications make sense to you.

Not clear why you think TDP-43 is potentially involved..? How do you speculate the preceding evidence in neurodegenerative diseases leads to question in asthma

specifically? Do you have a hypothesis related to role of TDP-43 in asthma?

Response: Thank you very much for the valuable comment. We refined the core scientific question for a more acceptable transition as below.

Based on the pathological cascade: **allergen exposure triggered tissue perturbations → cellular stress response in AECs → dysregulated TDP-43 expression → compromised NMD activity→ asthmatic airway inflammation**, this study aims to investigate the potential role of TDP-43 dysregulation in allergen-induced airway asthmatic inflammatory response.

We reorganized the **Introduction** section accordingly:

"Asthmatic airway inflammation is triggered by a wide array of allergens, including pollens, house dust mites (HDM), cockroach antigen (CA) and mould spores, which share minimal structural or biological commonality[21]. Despite their diversity, these allergens share the capacity to induce tissue perturbations on AECs. It remains unclear how AECs sense and translate these tissue perturbations to activate asthmatic airway response. Nevertheless, allergen-mediated tissue perturbations are increasingly recognized to induce cellular stress in AECs: Zhu *et al*. demonstrated that allergen exposure activates cellular stress in AECs, leading to stress granule formation and subsequent IL-33 maturation and secretion[22]; endoplasmic reticulum (ER) stress is closely associated with asthmatic airway response, as evidenced in both allergen-induced asthmatic murine models and airway epithelial brushing specimens from asthmatic patients[6, 7]; preclinical studies also showed that suppression of ER stress

significantly diminished asthmatic airway response[8].

In eukaryotic cells, aberrantly processed mRNAs are inspected and eliminated by RNA quality control (RQC) machineries, such as nonsense-mediated mRNA decay (NMD) triggered by premature termination codons (PTCs), thereby maintaining transcriptome integrity[23]. Deteriorated NMD activity contributes to the pathogenesis of ageing, genetic diseases and cancers[23]. While cellular stress frequently compromises NMD activity and causes the accumulation of stress-related and NMD-sensitive transcripts[24, 25, 26], the mechanisms through which cellular stress impairs NMD remain elusive. It is reasonable to hypothesize that allergen-induced tissue perturbations and associated cellular stress may also disrupt NMD in AECs, playing a role in the onset of asthmatic airway reactions.

TAR DNA-Binding Protein-43 (TDP-43, encoded by the *TARDBP* gene) is a master regulator in RNA metabolism and performs a crucial role in RQC[27]. In motor neurons (MNs), TDP-43 represses cryptic exon splicing, ensuring correct processing of pre-mRNAs, such as *STMN2* and *UNC13A*, which are essential for neural development[28, 29]. The nuclear clearance of TDP-43 in MNs is a defining characteristic of neurodegeneration in TDP-43 proteinopathies including amyotrophic lateral sclerosis and frontotemporal dementia[30]. While proper expression level and correct nuclear distribution of TDP-43 is required for its functions in RNA splicing, cellular stress frequently induces dysregulated TDP-43 expression, nuclear condensation and cytoplasmic aggregation, directly effecting RQC[12, 13]. Ectopic overexpression of TDP-43 in murine neurons dampened NMD, leading to cytotoxicity and survival issues[31, 32]. ”

Here we reported that allergen-induced TDP-43 expression in AECs drives asthma pathogenesis by inducing UPF1-mediated NMD impairment, leading to accumulation of pathogenic transcripts containing upstream open reading frames (uORFs) in their 5'untranslated regions (5'UTRs) and long 3'UTRs, such as the transcripts encoding cholinergic receptor muscarinic 1 (CHRM1), whose expression on AECs plays essential role in asthmatic response through recognition of the vagal neurotransmitter acetylcholine (Ach).

This long sentence ties together disparate findings and in doing so loses accuracy. There is no data on asthma pathogenesis- and what does asthma pathogenesis mean? Inception of disease, progression, exacerbation frequency, disease severity, responsiveness to therapy etc this can only be assessed by longitudinal clinical study and cannot be deduced from mouse experiments.

Response: Thank you for your insightful comment. We agree that the animal studies in the manuscript can only give insights into the role of TDP-43 in AEC acute response to allergen rather than asthma pathogenesis.

The sentence has been revised in the **Introduction** as below:

"Here we reported that allergen exposure-induced TDP-43 expression in AECs drives asthmatic airway inflammation by dampening UPF1-mediated NMD activity, leading to accumulation of NMD-sensitive transcripts encoding pathogenic molecules including cholinergic receptor muscarinic 1 (CHRM1), whose expression on AECs plays essential role in asthmatic response through recognition of the vagal

neurotransmitter acetylcholine (Ach)."

General comment for background-the description of asthma is incomplete and inaccurate in places. Asthma is a clinical umbrella term covering multiple endotypes and when referring to pathogenesis this should be acknowledged and specific pathogenic mechanisms identified.

Response: Thank you for the valuable feedback. We agree that this study focuses on allergen-induced acute inflammatory response in AECs rather than asthma pathogenesis. The **Background** section has been updated in accordance with your suggestion. The title has also been changed into "**TDP-43 drives allergen-induced inflammation and influenza A virus replication in airway epithelium**".

Results

Figure 1 What allergic individuals? Do they have asthma - this is not clear. What allergen are they sensitive to/challenged with? Were all subjects exposed to the same allergen? What is meant by pathogenic asthma state?

Response: Thank you for this insightful comment, which is valuable for highlighting the clinical relevance of our study.

As you suggested, we gave more details about the scRNA-seq dataset of human airway mucosa from allergic individuals as below:

Participant characteristics:

	Allergic controls	Allergic Asthmatics

	(**AC**)	(**AA**)
History of asthma	×	✓
Allergic symptoms to HDM and/or cat	✓	✓
Skin prick test (**SPT**)	Positive	Positive
Lung function	Baseline FEV_1 and FVC normal	Baseline FEV1≥75% predicted
Methacholine challenge	Negative	Positive

The same stock of allergen was used for both SPT and segmental allergen challenge (SAC): standardized **HDM** [Dermatophagoides pteronyssinus; Greer Laboratories, 10,000 allergy units (AU) per ml] and/or **cat hair extract** [Felis catis; Greer Laboratories, 10,000 bioequivalent allergy units (BAU) per ml].

For the SAC procedures:

1) Diluent (**Dil**) was then administered to the anterior segment of the right upper lobe followed by allergen (**Ag**) to the lateral segment of the right middle lobe.

2) 24 hours later, endobronchial brush samples were obtained from the Dil- and Ag-challenged segmental airways for scRNA-seq.

Two subpopulations of ciliated cells (Cili) were identified (Response Figure 2: S1a) and the mucous Cili subpopulation highly expressed *IL33*, *SCGB3A1*, *MUC5AC* and

SCGB1A1 Response Figure 2: S1b). To infer the potential involvement of RQC in allergen-induced asthmatic response, we inquired a RQC signature based on RNA splicing and surveillance-related genes in ciliated cells. Among both allergic controls (AC) and allergic asthmatic s(AA) subjects, Cili and mucous Cili with allergen exposure (Ag) displayed substantially decreased RQC scores compared to that of diluent exposure (Dil) (Response Figure 2: 1a). Of note, compared to Dil-Cili, Ag-Cili under-expressed genes encoding components of NMD machinery (*UPF1*, *UPF2*, *UPF3B*, *SMG6* and *SMG1*) in both AC and AA individuals (Response Figure 2: 1b). However, accumulated expressions of NMD targets were only observed in Ag-Cili from AA subjects (Response Figure 2: 1b).

These results suggest that AECs from allergic asthmatics may possess compromised NMD activity.

Response Figure 2

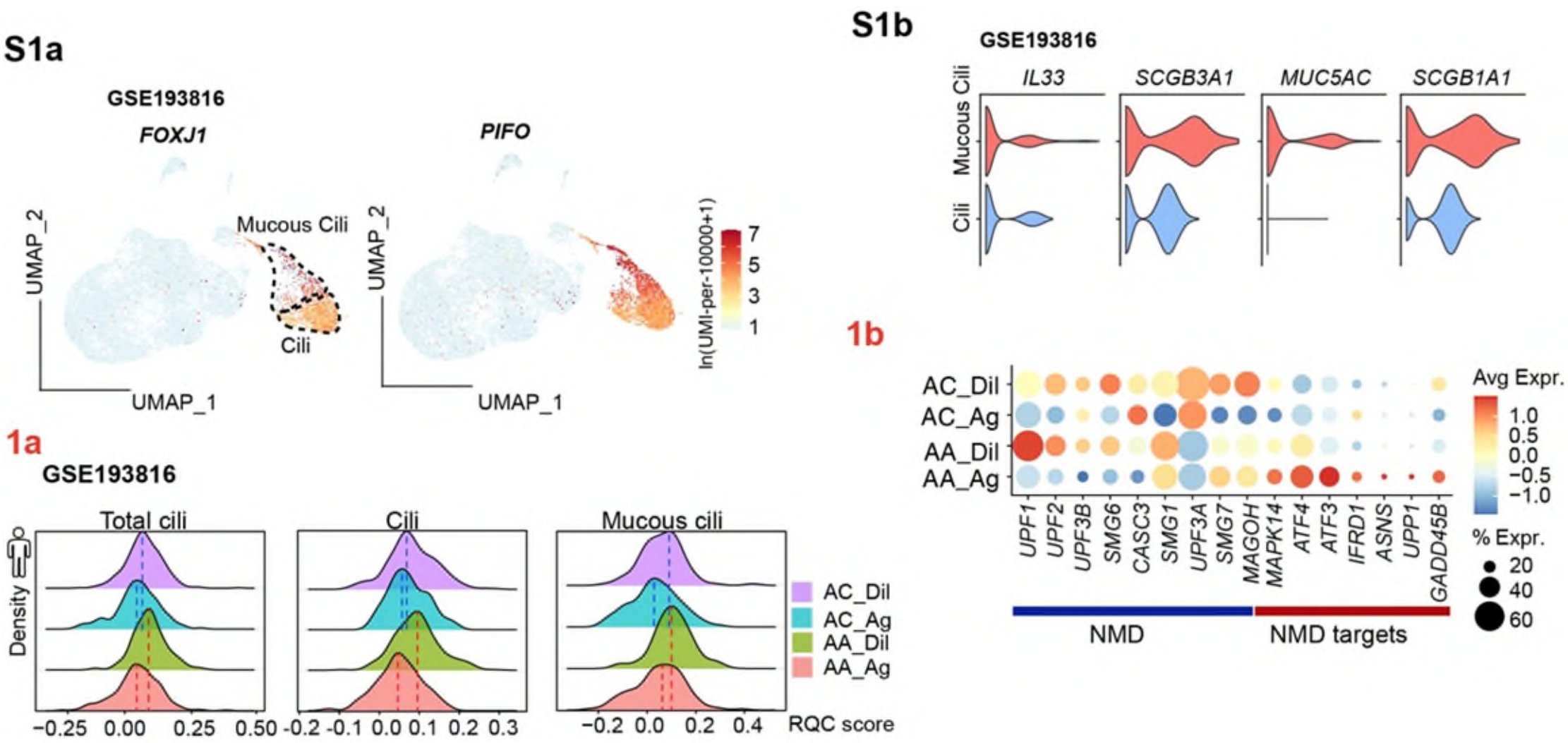

Response Figure 2 | **S1a**, UMAP plots showing marker gene expressions of ciliated cells in scRNA-seq dataset of airway mucosa from allergic asthmatic individuals under allergen or diluent exposure (GSE193816). Color intensity was proportional to its

normalized unique molecular identifier (UMI). **S1b**, Violin plots showing differential gene expressions between mucous Cili and Cili subpopulation. **1a**, Histogram plots comparing the RQC signature scores in ciliated cells from subjects with exposure of allergen or diluent (GSE193816). **1b**, Dot plots comparing expressions of NMD-related genes and NMD targets across ciliated cell subpopulations from subjects with allergen or diluent exposure. Ag: allergen, Dil: diluent.

Why were 3 different models initially assessed and the OVA only examined further - could argue that OVA is least clinically informative. OVA is a "clean" model of type 2 allergic inflammation-allergic airways disease.

Response: Thanks for the constructive advice. In addition to the OVA-induced model, we employed the CA-induced neutrophilic asthmatic model, which is more clinically informative.

We validated whether CA could significantly induce airway Tdp-43 expression in a new batch of mice with CA-induced asthma. Quantitative IF analysis of Tdp-43 in lung tissues from six mice per group (CTRL and CA) now confirms its significant elevation in the CA group compared to CTRL group (Response Figure 3: 1g,h). In parallel, we isolated primary AECs from CA-treated and control mice. The results from both western blot and IF assays collectively demonstrated that Tdp-43 expression was significantly elevated in the CA group (Response Figure 3: 1i, S2d-e).

In the revised manuscript, we observed that *Tardbp*$^{\Delta Scgb1a1}$ mice manifested significantly alleviated pulmonary pathologies, which was dose-dependently correlated

to the extent of TDP-43 loss (Response Figure 3: 3a). Flow cytometric results revealed profoundly reduced Th2 infiltrations in the lungs and neutrophil infiltrations in both lungs and BALF from HO-*Tardbp*$^{\Delta Scgb1a1}$ mice with CA exposure (Response Figure 3: 3b,c,e,f).

We administered the Tdp-43 targeting LNP-ASO to a CA-induced non-T2 asthmatic mouse model to further validate its therapeutic efficacy. The results unambiguously demonstrated the LNP-ASO group exhibited a marked reduction in pulmonary pathologies and immune cell infiltration, particularly neutrophils (Response Figure 3: 7i,j).

The OVA model was employed more extensively in our study due to its broader application in murine asthma modeling[18, 19, 20]. Importantly, in both CA and OVA mouse models, we consistently observed that Tdp-43 inhibition alleviated asthmatic airway response.

Response Figure 3

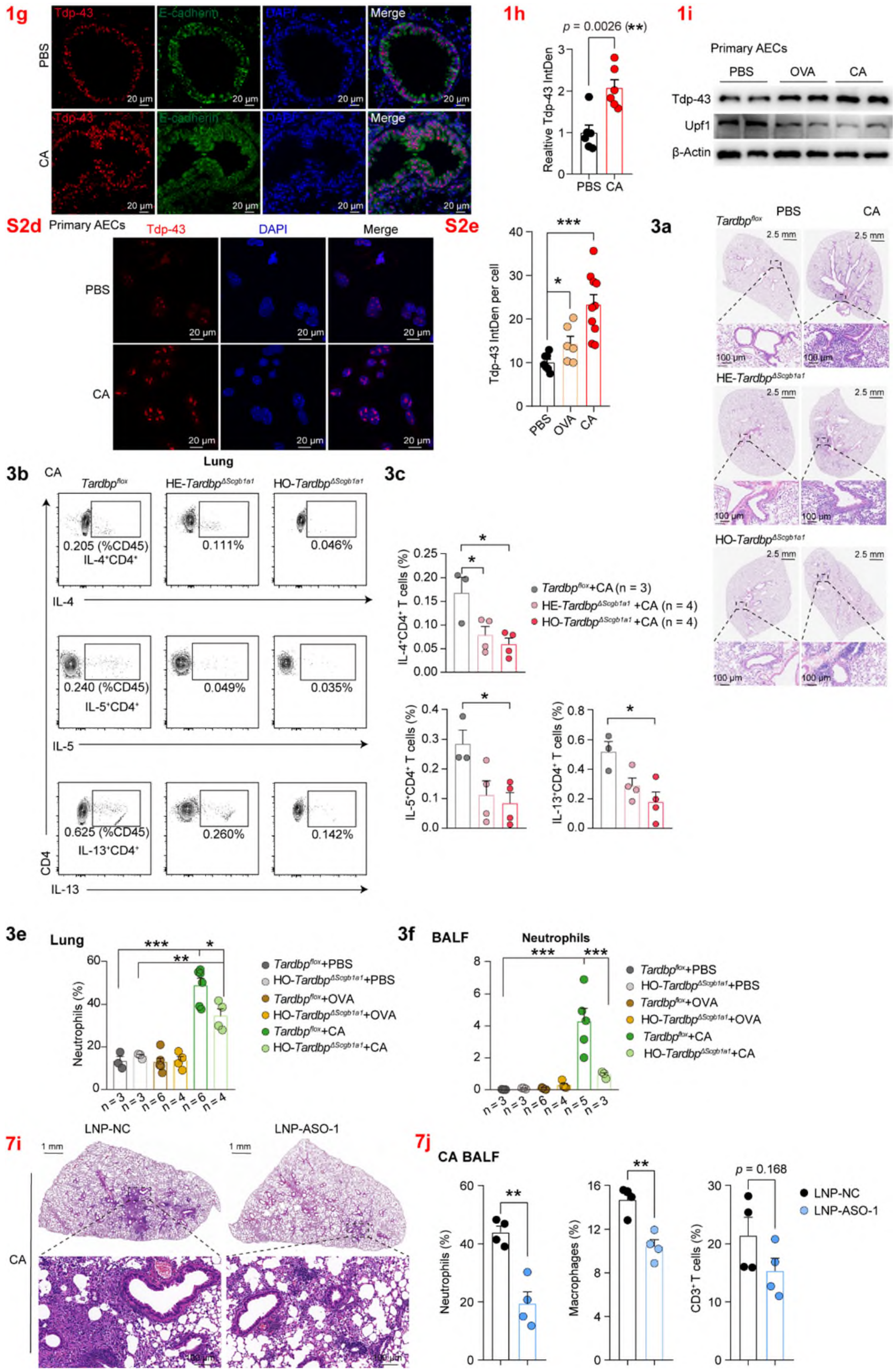

Response Figure 3 | **1g,** Representative IF images of Tdp-43 in the lungs from CA-induced asthmatic mice and control. **1h,** Quantitative IF results of Tdp-43 in (**1g**). **1i,**

Immunoblotting of Tdp-43 and Upf1 in the AECs isolated from CA/OVA-induced mice and control mice. **S2d**, Representative IF images of Tdp-43 in the AECs isolated from CA-induced mice and control mice. **S2e**, Quantitative IF results of TDP-43 in (**S2d**). **3a**, H&E staining comparing pathologies between CA-induced *Tardbp*$^{\Delta Scgb1a1}$ and *Tardbp*^{flox} mice. **3b**, Representative flow plots showing pulmonary infiltrations of Th2 across CA-induced HO-*Tardbp*$^{\Delta Scgb1a1}$, HE-*Tardbp*$^{\Delta Scgb1a1}$ and *Tardbp*^{flox} mice. **3c**, Bar plots summarizing the flow cytometric results in (**3b**). **3e** and **3f**, Bar plots showing flow cytometric results of neutrophil infiltrations in the lung (**3e**) and BALF specimens (**3f**) from *Tardbp*$^{\Delta Scgb1a1}$ and *Tardbp*^{flox} mice. **7i**, H&E staining of pulmonary pathologies in CA-induced mice receiving i.n. LNP-ASO-1 intervention and control. **7j**, Bar plots showing flow cytometric results of immune cell infiltrations in the BALF of CA-induced mice receiving i.n. LNP-ASO-1 intervention and control.

To investigate the expression patterns of RQC-related genes, we performed full-length transcript sequencing (smart-seq2) with purified AECs from OVA-induced asthmatic mice. Gene Set Enrichment Analysis (GSEA) results revealed that AECs from asthmatic mice had increased expression of genes enriched in asthma Such as…? Were they consistent with allergen challenged people?

Response: Thank you very much for the useful comment. KEGG term "**asthma mmu05310**" contained genes involved in asthmatic response. The full list of genes of mmu05310 on the KEGG website are: *H2-Ea, Epx, Fcer1a, Ms4a2, Fcer1g, H2-Aa, H2-Ab1*, *H2-Eb1*, *H2-DMa*, *H2-DMb1*, *H2-DMb2*, *H2-Oa*, *H2-Ob*, *Il10*, *Il13*, *Il3*, *Il4*,

Il5, *Il9*, *Prg2*, *Ccl11*, *Tnf*, *Cd40*, *Cd40lg* and *H2-Eb2* (Adapted from https://www.genome.jp/dbget-bin/www_bget?pathway+mmu05310). These genes are evolutionarily conserved in humans and mediate immune responses in asthma.

Among these genes, AECs from asthmatic mice had increased expression of genes encoding MHC class II components (*H2-Aa*, *H2-Ab1*, *H2-Eb1*, *H2-DMb1*, *H2-DMa*), inflammatory cytokines (*Tnf*, *Il10*), high affinity IgE receptors (*Fcer1g*, *Fcer1a*) (Response Figure 7: S2a, 4a). Besides, asthmatic AECs overexpressed *Tslp* (encoding one of the alarmins), *Csf3* and several genes encoding chemokines for granulocytes or monocytes (*Cxcl5*, *Cxcl3*, *Cxcl2*, *Cxcl14*, *Cxcl1*, *Ccl8*) and mucin-secretion related genes (*Muc13*, *Muc5ac*, *Muc5b*, *Tff1* and *Tff2*) (Response Figure 7: 4a). However, AECs from *Tardbp*$^{\Delta Scgb1a1}$ mice with OVA stimulation failed to launch these expressional changes (Response Figure 7: S4b, 4a), indicating that Tdp-43 play an important role in allergen-induced asthmatic airway response.

Response Figure 7

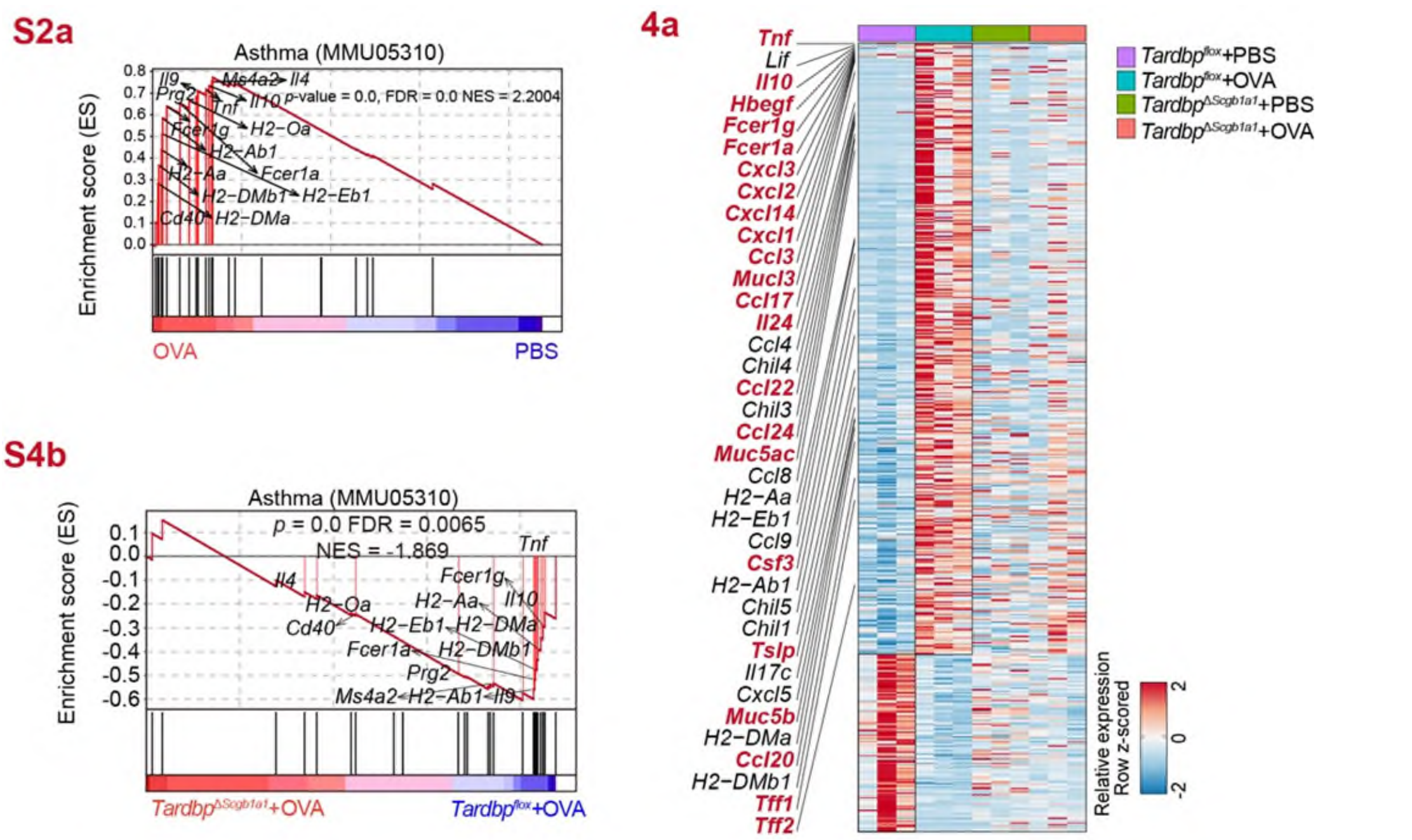

Response Figure 7 | **S2a**, GSEA analysis of asthma (MMU05310) signaling in AECs from OVA- vs PBS-induced mice. **4a**, Heatmap showing that AECs from allergen-stimulated *Tardbp*$^{\Delta Scgb1a1}$ mice showed blunted expression of AHR-related genes. **S4b**, GESA analysis revealing that AECs from asthmatic *Tardbp*$^{\Delta Scgb1a1}$ mice had decreased expressions of genes enriched in asthma (MMU05310).

AECs from asthmatic mice showed increased expression of genes involved in asthmatic response, such as Mucl3, Ccl22, Rnase2a, Retnla, etc. These are not commonly reported 'asthma signature' genes reported in humans. This reinforces that mice do not have asthma and AECs from mice acutely sensitised and challenged with allergen will not recapitulate the intrinsic disease phenotype of AECs from adult asthmatics. As a result, the analysis of mouse AECs in this context is of limited value here.

Response: We are grateful to the reviewer for the constructive comment.

We revisited the RNA-seq data of mouse asthmatic AECs and found that AECs from OVA-induced asthmatic mice exhibited upregulated expression of Tslp (encoding the epithelial alarmin TSLP), high-affinity IgE receptor genes (*Fcer1g*, *Fcer1a*), as well as numerous chemokine genes involved in recruiting granulocytes and monocytes (*Cxcl5*, *Cxcl3*, *Cxcl2*, *Cxcl14*, *Cxcl1*, *Ccl8*) and genes associated with mucin secretion (*Mucl3*, *Muc5ac*, *Muc5b*, *Tff1*, *Tff2*) (Response Figure 8: 4a). These genes correspond to a common "asthma signature" also observed in human studies.

We agree with the reviewer that murine models do not fully recapitulate all aspects of human chronic asthma. However, the OVA-induced acute allergic inflammation

model is a well-established and valuable tool for studying specific, conserved immunological pathways, such as the TSLP-alarmin response, IgE receptor signaling, and chemokine-driven granulocyte recruitment, that are central to asthma pathogenesis in both species. The significant upregulation of these hallmark genes in our model demonstrates its relevance for investigating these core mechanistic pathways. Therefore, while acknowledging the limitations, we believe the analysis of mouse AECs in this context provides meaningful insights into the epithelial-initiated immune responses that are shared between mice and humans.

Response Figure 8

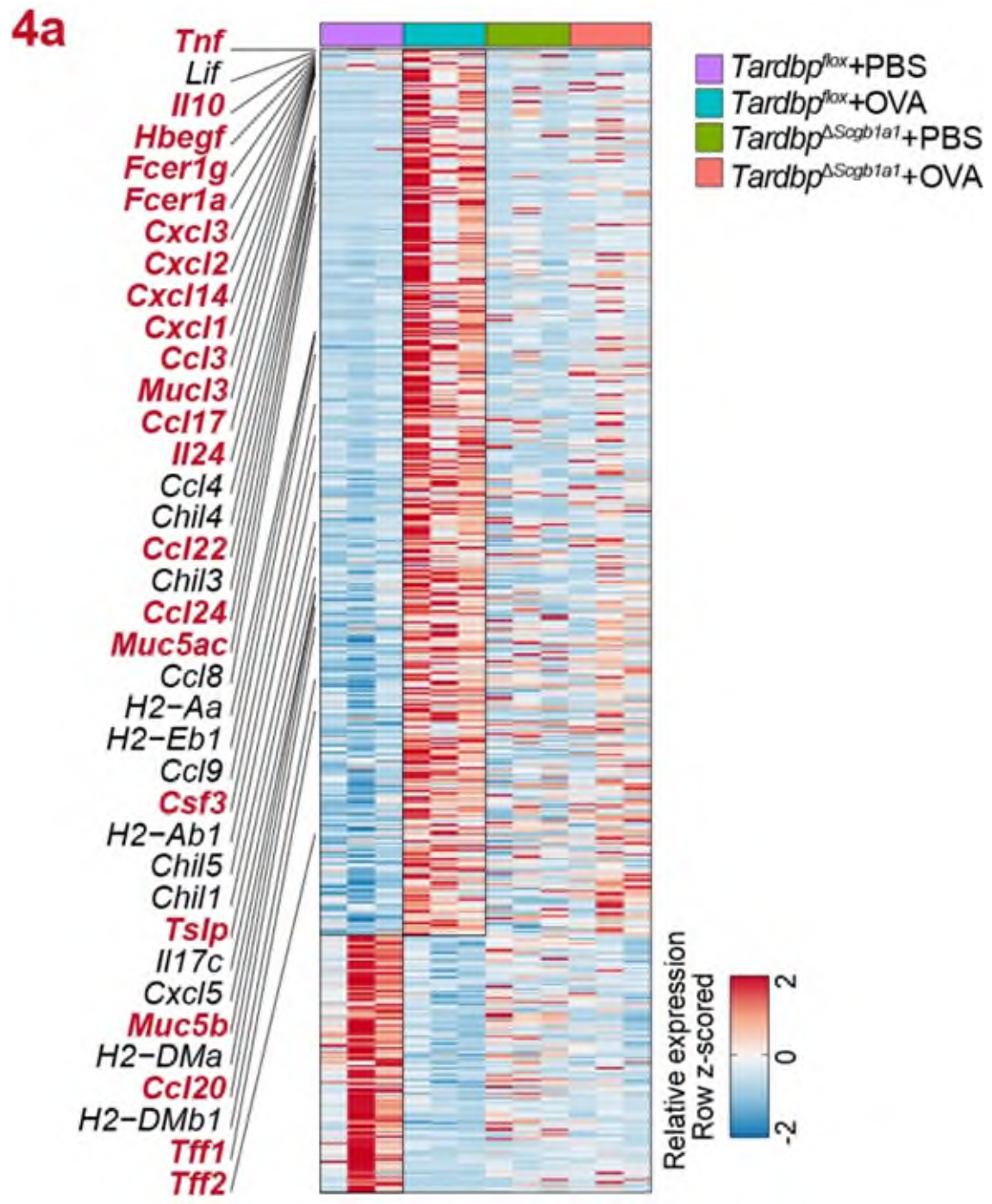

Response Figure 8 | **4a**, Heatmap showing that AECs from allergen-stimulated *Tardbp*$^{\Delta Scgb1a1}$ mice showed blunted expression of AHR-related genes.

Fig 1J/K the data is not convincing-the images for healthy vs ACOS do not look comparable to me.

Response: Thank you for the comment. We recollected airway tissues from patients with asthma (without comorbidity of COPD) and relatively healthy control subjects, followed by IF co-staining of TDP-43 and E-Cadherin (a marker for epithelial cells). Quantitative analysis of TDP-43 expression within E-Cadherin-positive populations revealed a significantly higher level of TDP-43 in the airways of asthma patients compared to the control group (Response Figure 5: 1j, k).

Response Figure 5.

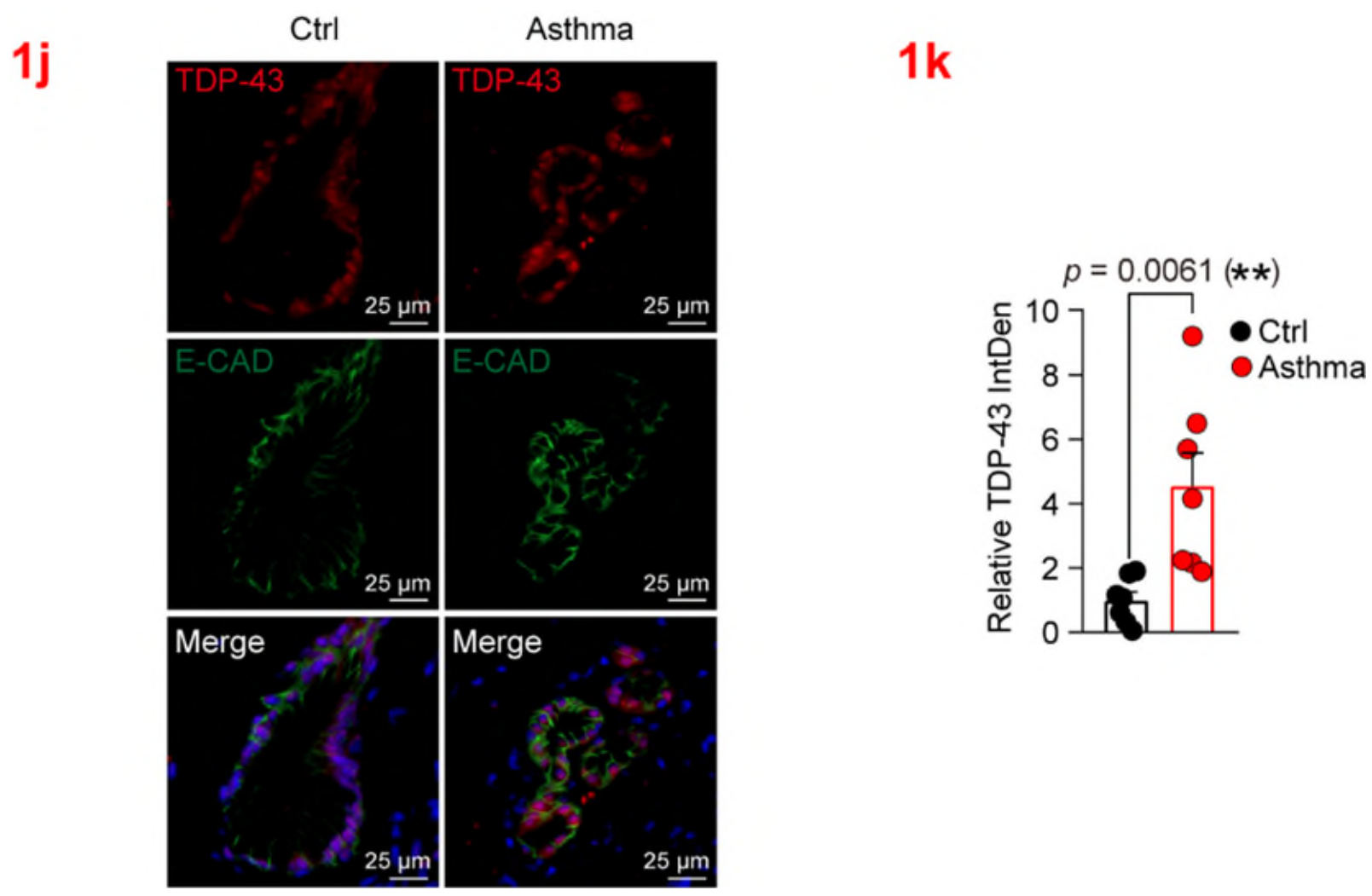

Response Figure 5 | **1j**, Representative IF staining of TDP-43 and E-cadherin in the bronchoscopic mucosal specimens from patients with asthma and relatively healthy subjects. **1k**, Quantitative IF results of TDP-43 within E-Cadherin-positive regions in (**1j**).

Fig 2 The data generated from silenced TARDBP in cell line BEAS-2B do NOT indicate that TDP-43 plays a crucial role in mediating inflammatory responses in AECs. This is a non-differentiated cell line so provides little insight in AEC biology.

Response: Thank you for pointing this out. We agreed that the BEAS-2B cell line is

non-differentiated and thus it is a suboptimal model for illustrating airway epithelium-mediated inflammatory response. Meanwhile, the RNA sequencing data was generated on BEAS-2B cells with LPS stimulation, which is not optimal for studying type 2 innate inflammatory response.

For these considerations, we removed these data in the revised manuscript.

Characterises TDP-43 knockout mice - comparing parental (flox), heterozygous ko (HE) and homozygous ko (HO). Please indicate how the data was confirmed -

Response: Thank you for pointing this out. We quantified the airway expression of Tdp-43 via IF staining in flox, HE and HO groups (Response Figure 9: 2a). The results showed that the expression of Tdp-43 was significantly lower in both the HE and HO groups than in control group, and it was even lower in the HO group compared to the HE group (Response Figure 9: 2b). Moreover, staining and quantification were performed on lung tissues from at least six mice per group to ensure reliability.

To assess the TDP-43 knockout efficiency and the targeted cell types, we conducted scRNA-seq using lung tissues from OVA-challenged mice. The data showed a significant reduction of Tdp-43 expression specifically within the club cells and ciliated cells of the airway epithelium in *Tardbp*$^{\Delta Scgb1a1}$ mice relative to controls (Response Figure 9: 2d, e).

ScRNA-seq analysis also drew the following findings:

- **An alleviation of airway metaplasia in the OVA-*Tardbp*^{flox} group.** Secretory-pro, club, and alveolar cells isolated from OVA-*Tardbp*$^{\Delta Scgb1a1}$ mice exhibited a

reduced capacity of mucin secretion compared to those from OVA-*Tardbp*^{flox} mice (lower expression of *Muc5ac*, *Spdef*, *Tff2*, *Fcgbp*, *Clca1*) (Response Figure 9: 2f).

- **OVA-*Tardbp*$^{\Delta Scgb1a1}$ mice exhibited an attenuated myeloid inflammatory response**: Notably reduced mast cell infiltrations (*Fcer1g* and *Mcpt8*) were observed in the lungs of OVA-*Tardbp*$^{\Delta Scgb1a1}$ mice (Response Figure 9: S3f, 2g). Furthermore, mast cells from OVA-*Tardbp*$^{\Delta Scgb1a1}$ group also under-expressed genes related to granulocyte inflammation (*Tnf*, *Il13*, *Il1rl1*, *Il6*, *S100a6*, *Serpinb1a*, *F2r*, *P2rx4*) (Response Figure 9: 2h). The lungs of OVA-*Tardbp*$^{\Delta Scgb1a1}$ mice exhibited an increased abundance of M2-like macrophages, which possess immunomodulatory and tissue-repair properties (*Mertk*, *Chil3*, *Arg1*, *Mrc1*) (Response Figure 9: S3f, 2g).
- **The acquisition of the Th2-like program in T cells was significantly blunted in the OVA-*Tardbp*$^{\Delta Scgb1a1}$ mice**. Scart1^{+} γδT17 cells are mucosal-resident lymphocytes characterized by a Th17-like transcriptional signature that is essential for maintaining mucosal homeostasis[37]. In OVA-*Tardbp*^{flox} mice, this population underwent a trans-differentiation from its steady-state mucosal-γδT17 identity (marked by lower expression of *Sox13*, *Rorc*, *Scart1*, *Il17re*, and *Il17a*) toward an activated, Th2-like state, evidenced by elevated expression of *Cd69*, *Il2ra*, *Gata3*, *Il1rl1*, *Klrg1* and *Runx1*(Response Figure 9: S3g, 2i, 2j)[38]. Interestingly, a population of immature γδT cells (imm-γδT) (*Dntt*$^{+}$*Rag1*$^{+}$*Ptcra*$^{+}$) was specifically detected in the lungs from OVA-*Tardbp*^{flox} mice (Response

Figure 9: S3g, 2i). Notably, both these imm-γδT cells and naïve T cells in OVA-*Tardbp*^{flox} mice similarly acquired a Th2-like program (Fig. 2j). In sharp contrast, the acquisition of the Th2-program, observed across Scart1^{+}γδT17, imm-γδT and naïve T cells in OVA-*Tardbp*^{flox} mice, was blunted in the OVA-*Tardbp*$^{\Delta Scgb1a1}$ mice Response Figure 9: 2j).

Taken together, the scRNA-seq analysis revealed that, upon allergen exposure, *Tardbp*$^{\Delta Scgb1a1}$ mice exhibited an attenuated asthmatic airway microenvironment compared with *Tardbp*^{flox} mice.

Response Figure 9

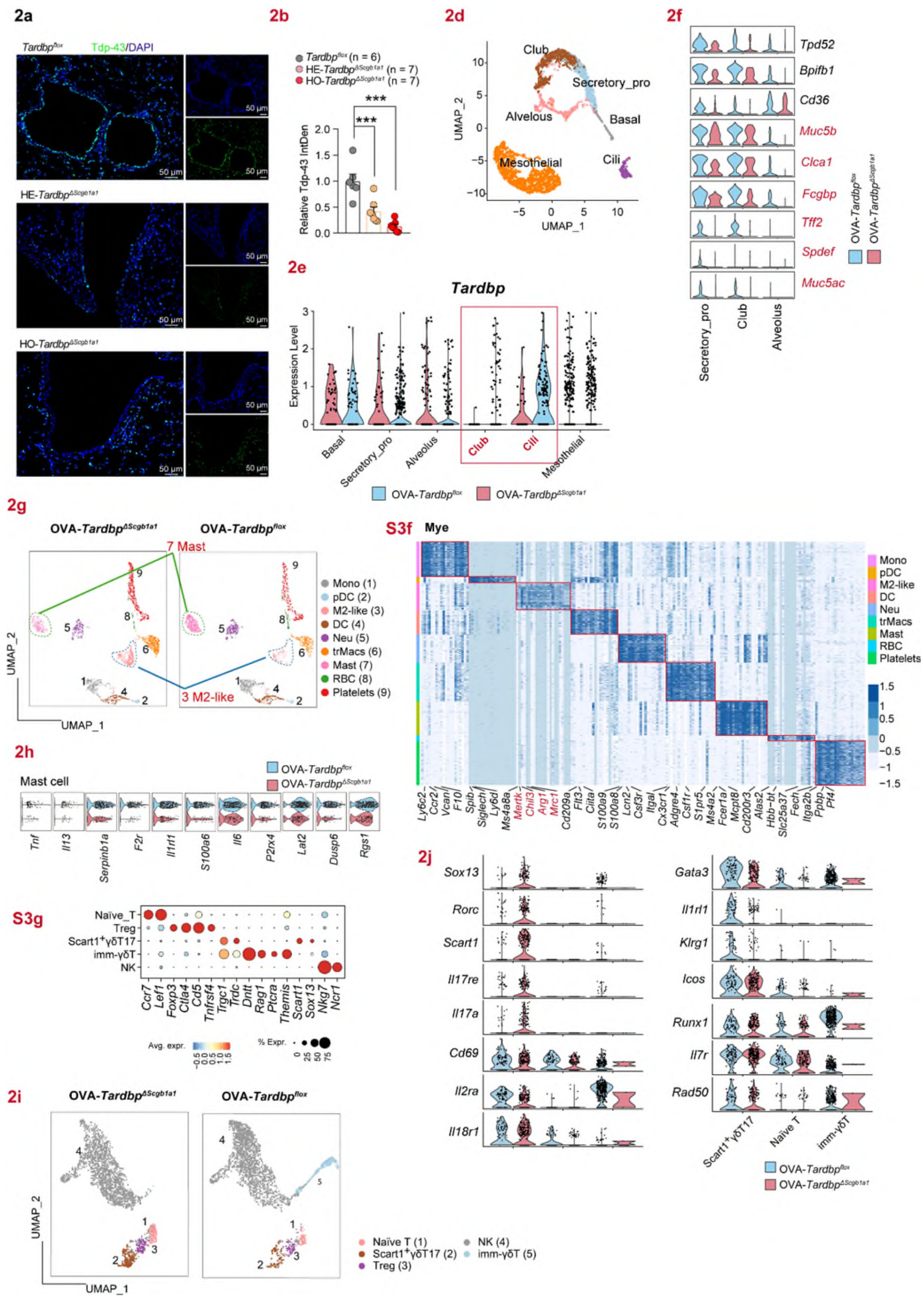

Response Figure 9 | **2a**, Representative IF images of Tdp-43 expression in the lung tissues from *Tardbp*$^{\Delta Scgb1a1}$ and *Tardbp*^{flox} mice. **2b**, Bar plots comparing the Integrated Density of TDP-43 signals in (**2a**). **2d**, UMAP plot showing the cell types of epithelium

were displayed separately according to OVA-stimulated *Tardbp*$^{\Delta Scgb1a1}$ and *Tardbp*^{flox} mice. **2e**, Violin plot showing the expression of *Tardbp* between OVA-stimulated *Tardbp*$^{\Delta Scgb1a1}$ and *Tardbp*^{flox} mice in epithelial cell types. **2f**, Violin plot showing the expression of gene about mucin secretion in epithelial cell types. **2g**, UMAP plot showing the cell types of myeloid cells were displayed separately according to OVA-stimulated *Tardbp*$^{\Delta Scgb1a1}$ and *Tardbp*^{flox} mice. **S3f,** Heatmap showing expression of the top 20 differential genes in different myeloid cell types. Each row represents an individual cell. **2h**, Violin plot showing the expression of gene related to granulocyte inflammation in mast cell. **S3g,** Dotplots showing expression level of marker genes of various cell types in B cells and T cells. **2i**, UMAP plot showing the cell types of T cells were displayed separately according to OVA-stimulated *Tardbp*$^{\Delta Scgb1a1}$ and *Tardbp*^{flox} mice. **2j**, Violin plot showing the expression of gene related to type 2 inflammatory stimulation in T cell types.

Please indicate repeat experiments, in the figure legend.

Response: Thank you for your helpful reminder. In accordance with your kind advice, the details regarding the number of experimental replicates have now been included in the figure legends.

The description of the mouse data is very superficial.

Response: Thank you for the insightful comments on the description of the mouse data. In the revised manuscript, we elaborated the murine results in more details:

- We revealed that Tdp-43 ablation in AECs alleviated the asthmatic airway microenvironment upon OVA stimulation by performing single cell RNA sequencing analysis (please refer to Response Figure 9).
- We confirmed that genes encoding epithelial alarmins were significantly downregulated in AECs from allergen-induced *Tardbp*$^{\Delta Scgb1a1}$ mice (please refer to Response Figure 19).
- The results of pulmonary pathology and flow cytometry confirmed that *Tardbp*$^{\Delta Scgb1a1}$ mice were resistant to either OVA-induced eosinophilic (T2) or CA-induced neutrophilic (non-T2) asthmatic airway response (please refer to Response Figure 3).

What day post allergen challenge is assessed?

Response: Thank you very much for the constructive comment. As schematically illustrated in Response Figure 10: S1d, mice were sacrificed for tissue collection on day 18 of the entire modeling protocol, which was 24 hours after the final administration of allergen. This experimental model was adapted from the widely used protocol[18, 39].

Response Figure 10

S1d

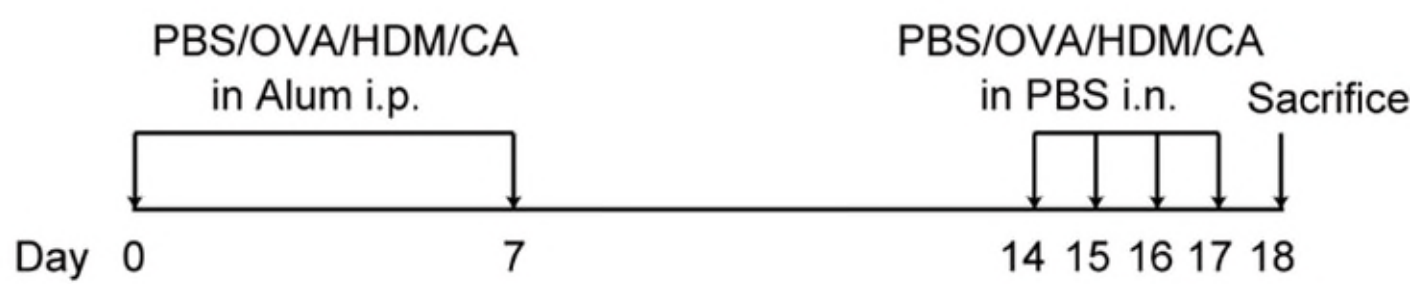

Response Figure 10 | **S1d**, Schematic diagram illustrating construction of allergen-induced asthmatic mouse models.

Figure 3 To gain mechanistic insights of TDP-43 involvement in asthma pathogenesis, we performed smart-seq2-seq on AECs isolated from *Tardbp*$^{\Delta Scgb1a1}$ and *Tardbp*^{flox} mice under OVA-induced asthmatic state.

This approach does not give insight into asthma pathogenesis - this will give insight into the role of TDP-43 in AEC acute response to allergen.

Response: We are grateful to the reviewer for the insightful comment. We agreed that RNA-seq on AECs isolated from *Tardbp*$^{\Delta Scgb1a1}$ and *Tardbp*^{flox} mice under OVA-induced asthmatic state can only give insight into the role of Tdp-43 in AEC acute response to allergen rather than asthma pathogenesis.

The sentence has been rephrased in the revised manuscript as follows: "…To gain mechanistic insights of TDP-43 involvement in allergen-induced asthmatic airway response …".

The data in Figure 3 is qualitative so difficult to discern the value.

Response: Thank you for your inquiry. The Figure 3 in the initial submission displays the RNA-seq results and therefore only provides qualitative evidence for the negative regulatory role of Tdp-43 on NMD.

To validate the RNA-seq results, we examined the expression of *UPF1*, encoding the RNA-dependent helicase UPF1 (the rate-limiting component of NMD). We found that knockdown of TDP-43 significantly promoted the expression of UPF1, whereas overexpression of TDP-43 markedly suppressed it (Response Figure 11: 4f-i). Mechanistically, we discovered that TDP-43 binds to the mRNA of Upf1 and negatively

regulates its stability (Response Figure 11: 4j-m). Consistently, in primary airway epithelial cells from CA-treated mice, we observed an upregulation of Tdp-43 and a concomitant downregulation of Upf1 (Response Figure 11: 1i). Furthermore, the RNA stability of Upf1 was significantly reduced in the CA-treated group (Response Figure 11: 4n).

Response Figure 11

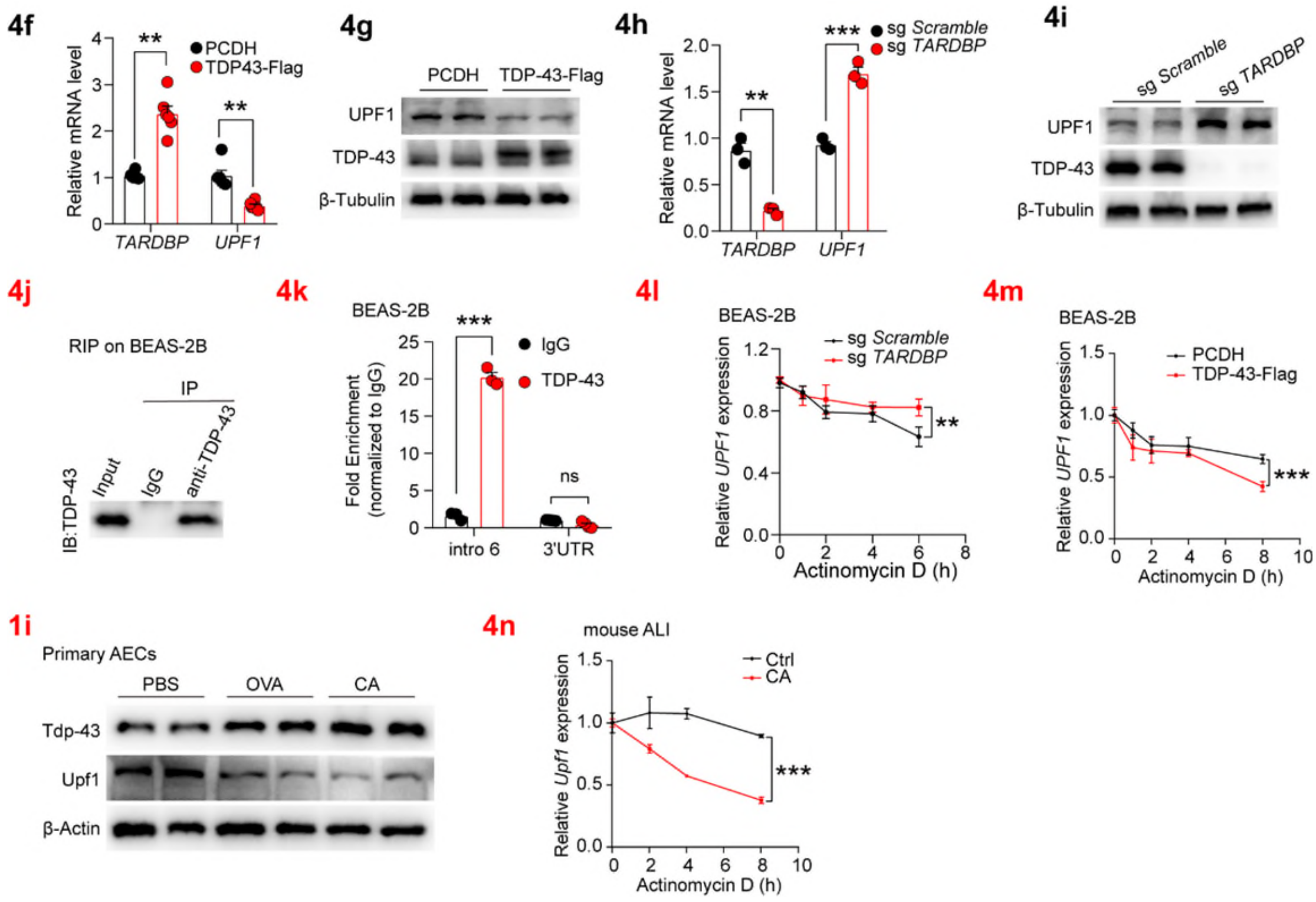

Response Figure 11 | **4f**, mRNA levels of TARDBP and UPF1 in TDP-43-overexpressed BEAS-2B cells. **4g**, Immunoblotting of TDP-43 and UPF1 in TDP-43-overexpressed BEAS-2B cells. **4h**, mRNA levels of TARDBP and UPF1 in TDP-43-knockdown BEAS-2B cells. **4i**, Immunoblotting of TDP-43 and UPF1 in TDP-43-knockdown BEAS-2B cells. **4j**, Immunoblotting of TDP-43 after RIP assay with TDP-43 antibody in BEAS-2B cells. **4k**, qPCR analysis of RNA pulled down by TDP-43 antibody in

BEAS-2B cells, using primers targeting the intron 6 or 3’UTR of UPF1. **4l**, mRNA level of UPF1 in TDP-43-knockdown BEAS-2B cells treated with 10 μg/ml Actinomycin D for 1 h, 2 h, 4 h, and 6 h. **4m**, qPCR results showing *UPF1* expression in TDP-43-overexpressed BEAS-2B cells treated with 10 μg/ml Actinomycin D for 1 h, 2 h, 4 h, and 8 h. **1i**, Immunoblotting of TDP-43 and UPF1 in primary airway epithelial cells from CA-treated mice and control mice. **4n**, mRNA expression of *Upf1* in allergen (CA)-induced and control AECs under air-liquid interface conditions, followed by treated with 10 μg/ml Actinomycin D for 2 h, 4 h and 8 h.

Figure 4 The link between TDP-43 and CHRM1 mRNA expression is interesting. At a molecular level in this synthetic system it is a compelling narrative relevant to asthma. However caution is needed with over interpreting the mechanistic link to asthma in the absence of any data that actually does so.

Response: We appreciate the reviewer for the constructive comment. To validate the hypothesis that NMD mediates *CHRM1* degradation through uORFs, we performed site-directed mutagenesis on the start codons of predicted uORFs of *CHRM1*'s with strong activity (Response Figure 12: 5h). Mutating the start codon of uORF11 markedly enhanced the capacity of *CHRM1*'s 5'UTR in driving the expression of luciferase reporter (Response Figure 12: 5j). Furthermore, knockdown of UPF1 significantly upregulated the expression of CHRM1, confirming this regulatory relationship (Response Figure 12: S5h). These findings conclusively identify *CHRM1* as a target of the NMD pathway.

Response Figure 12

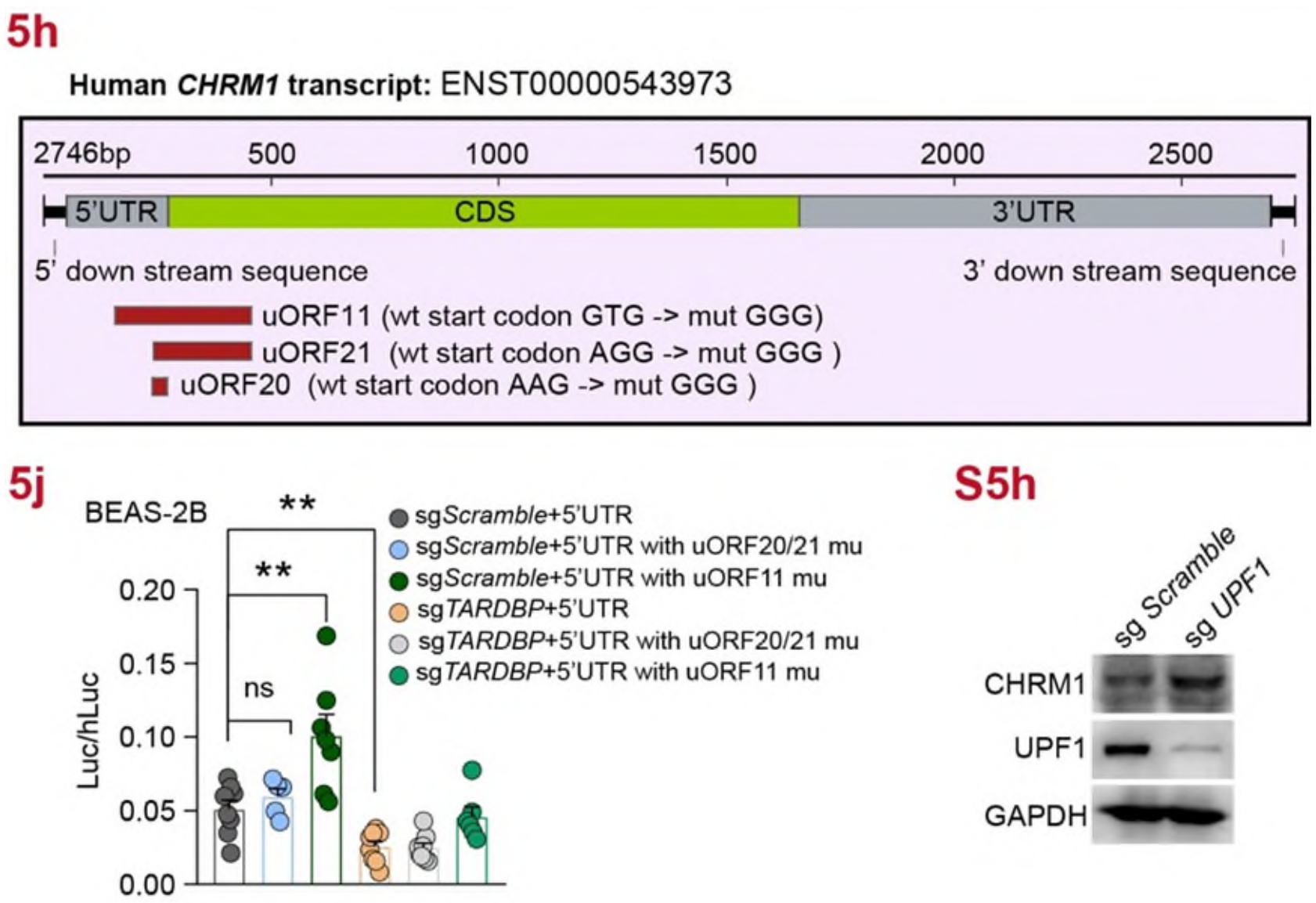

Response Figure 12 | **5h**, The locations of uORFs within the 5'UTR of human CHRM1 transcript. The red arrow indicates the potential location of exon junction complex (EJC). **5j**, Bar charts comparing relative Luc activities (Luc/hLuc) directed by 5'UTR between site-directed mutagenesis on the start codons of predicted uORFs of human CHRM1's in BEAS-2B cells. **S5h**, Immunoblotting of CHRM1 and UPF1 in UPF1-knockdown BEAS-2B cells.

Figure 5 I wonder why influenza was chosen as rhinovirus is the most common cause of asthma exacerbations.

Response: We appreciate the reviewer's comment. While investigating the function of TDP-43 in asthmatic airway response, we came across an article published in ***Nucleic Acids Res*** (Dupont *et al*., 2024)[40] reporting that TDP-43 as a critical host factor required for influenza A virus (IAV) replication, facilitating viral mRNA expression and

infectious virion production. Wondering the role of airway TDP-43 in IAV infection and influenza complicated asthma exacerbations (IAV-AE) *in vivo*, we performed the experiments of subjecting *Tardbp*$^{\Delta Scgb1a1}$ mice to IAV infection and IAV-AE modeling.

This is a secondary observation of this study and we believe that the potential involvement of TDP-43 in rhinovirus infection and its associated asthma exacerbations warrant further independent investigation.

The BEAS-2B data is limited - a timecourse of virus replication would be more informative - these are cell lines so presumably you are not limited by cell number. As such the data related to control of viral replication and anti-viral interferon is very limited.

Response: Thank you very much for the insightful comment. As suggested, we infected both TDP-43-overexpressing and control BEAS-2B cells with a single dose of PR8 virus in a parallel comparison. Cells were then collected at the respective time points of 24, 48, 72, and 96 hours post-infection for the assessment of NP protein levels. The results demonstrated that overexpression of TDP-43 significantly facilitated PR8 infection, as it was associated with a delayed viral clearance (Response Figure 13: 6c, 6d, S7a).

Response Figure 13

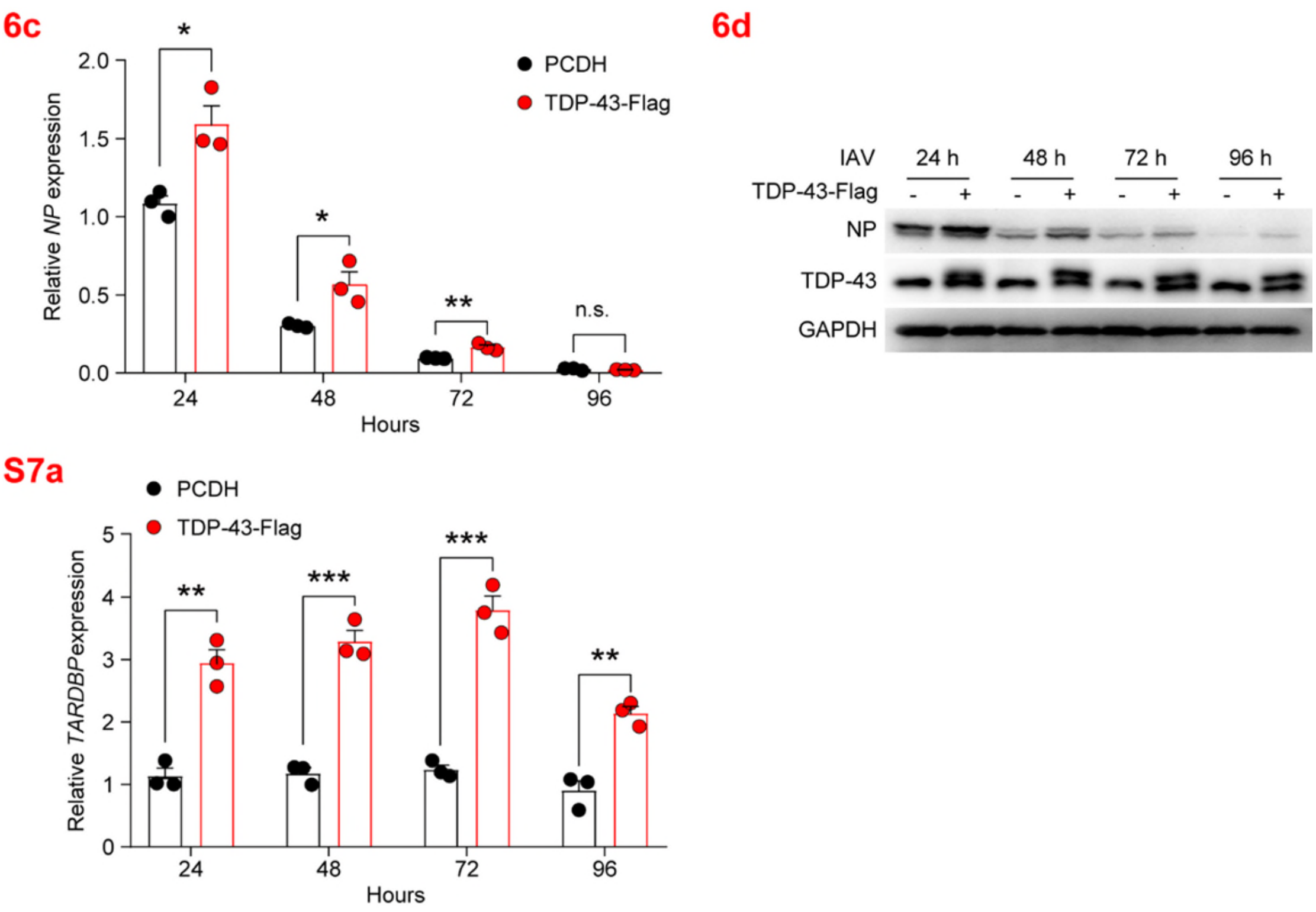

Response Figure 13 | **6c**, qPCR results showing *NP* expression in TDP-43-overexpressed BEAS-2B cells infected with PR8 at different time. **6d**, Immunoblotting of NP and TDP-43 in TDP-43-overexpressing BEAS-2B cells infected with PR8 at different time. **S7a**, qPCR results showing *TARDBP* expression in TDP-43-overexpressed BEAS-2B cells infected with PR8 at different time.

Regarding mouse influenza infection model - again a timecourse tracking lung viral load would be more informative. As it is the only data to support the conclusion is NP immunofluorescence (which is quantified) at a single timepoint.

Response: Thanks for the constructive advice. To assess viral infection and replication, *Tardbp*$^{\Delta Scgb1a1}$ and *Tardbp*^{flox} mice were infected with the PR8 virus. Lung tissues were harvested at 3, 6, 10, and 15 days post-infection and subjected to IF staining for the viral NP protein. The result show that NP protein levels in the lungs of *Tardbp*$^{\Delta Scgb1a1}$

group were significantly lower than those in the *Tardbp*^{flox} group at each time point (3, 6, 10, and 15 day) (Response Figure 14: S7b).

Response Figure 14

S7b

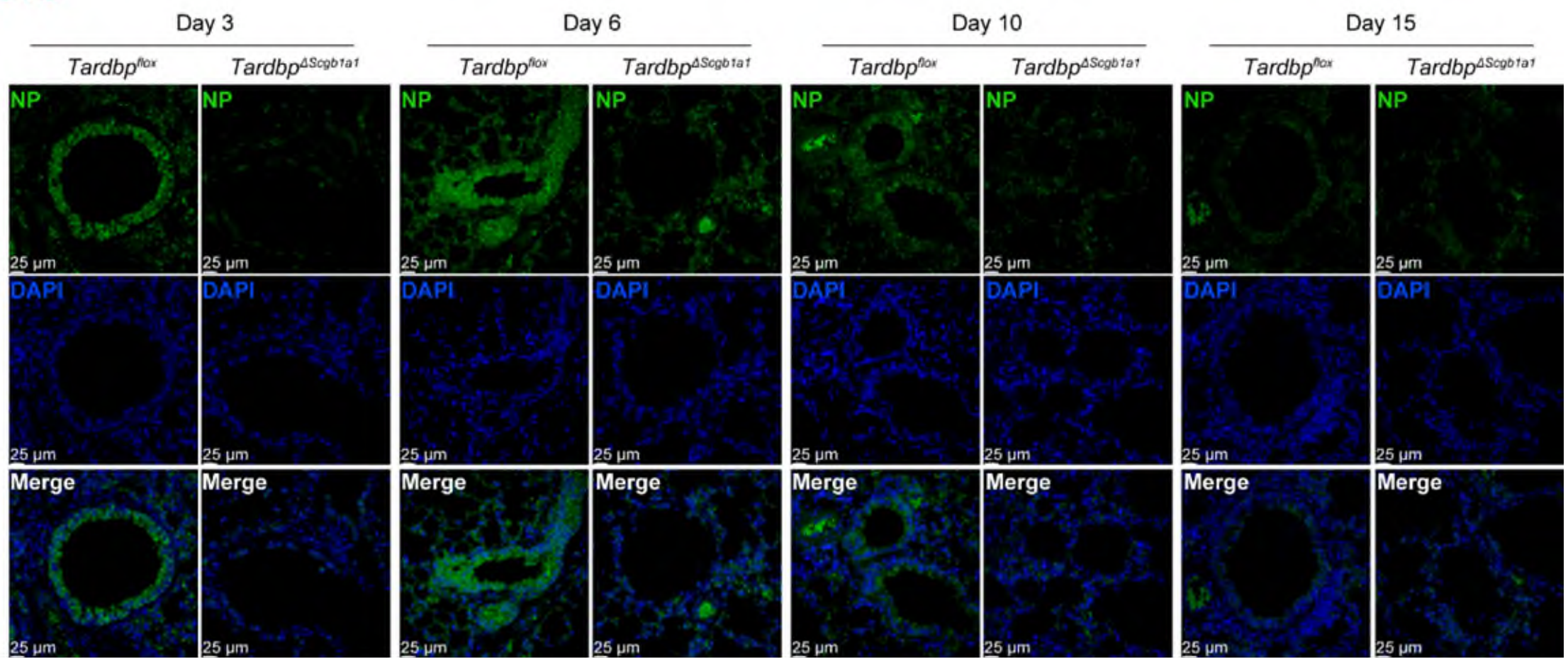

Response Figure 14 | **S7b**, Representative IF images of NP in lungs from *Tardbp*$^{\Delta Scgb1a1}$ and *Tardbp*^{flox} mice infected with the PR8 virus.

Figure 6 The title of this section really highlights misunderstanding of asthma - application of ASO-LNP targeting TDP-43 ameliorates asthmatic symptoms.

There is no assessment of anything related to symptoms. LNP ASO is delivered intranasally. This is done during the allergen sensitisation and challenge phase of the experiment - so cannot conclude if the treatment is preventing sensitisation or response to allergen in established sensitisation. This relevant to the research question - do the authors hypothesises that TDP-43 plays a role in asthma development, or allergen induced inflammation in established disease?

Response: Thanks for the constructive comment. We have revised the title of this section to “Application of ASO-LNP targeting TDP-43 ameliorates asthmatic airway

inflammations”.

- Our initial therapeutic strategy involved delivering LNP-ASO throughout the OVA sensitization and challenge phases, this protocol is designated as the prophylactic regimen (Response Figure 15: 7d). The results of pulmonary pathologies and flowcytometry revealed alleviated asthmatic airway inflammations in the lung tissues of mice from LNP-ASO group (Response Figure 15: 7f-g).
- To evaluate the therapeutic effect of LNP-ASO on established CA-induced sensitization, C57BL/6 mice were first sensitized via intraperitoneal injection over a two-week period. Subsequently, during the intranasal challenge phase, they were concurrently treated with LNP-ASO. This is called **therapeutic regimen** **(**Response Figure 15: 7h**)**. The results revealed that the LNP-ASO group exhibited a marked reduction in pulmonary pathologies and immune cell infiltration, particularly neutrophils (Response Figure 15: 7i-j).

These results imply a broader pathogenic role for dysregulated TDP-43 in AECs. Its promoting effect appears to extend from the early phase of allergen-induced inflammation to the later establishment of asthmatic airway response.

Response Figure 15

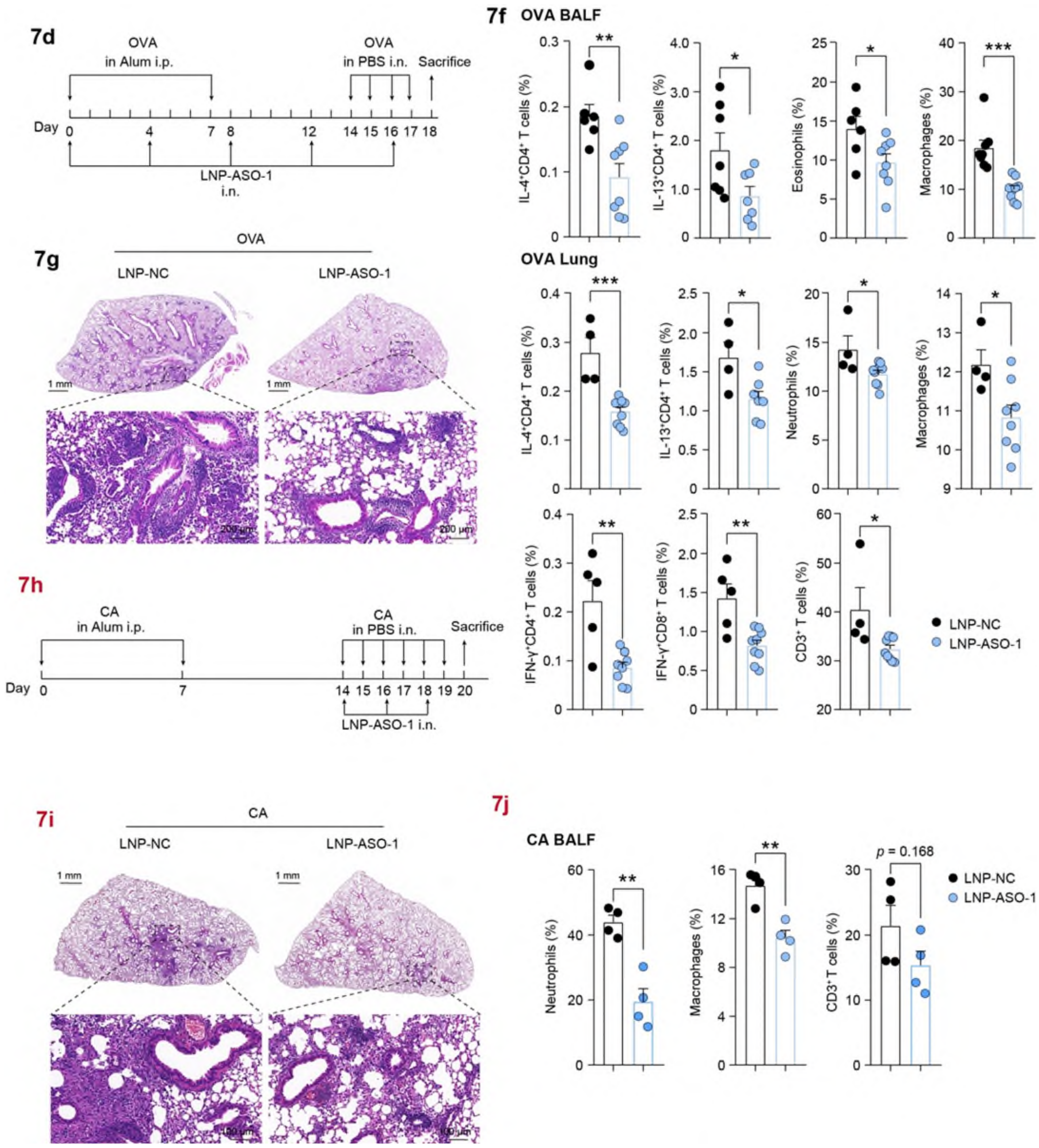

Response Figure 15 | **7d**, Schematic diagram illustrating therapeutic strategy of LNP-ASO targeting Tdp-43 in OVA-induced asthmatic mice. **7f**, Bar plots showing flow cytometric results of immune cell infiltrations in the lung tissues and BALF of OVA-induced mice receiving i.n. LNP-ASO-1 intervention. **7g**, H&E staining of pulmonary pathologies in OVA-induced mice receiving i.n. LNP-ASO-1 intervention. **7h**, Schematic diagram illustrating therapeutic strategy of LNP-ASO targeting Tdp-43 in CA-induced asthmatic mice. **7i**, H&E staining of pulmonary pathologies in CA-induced mice receiving i.n. LNP-ASO-1 intervention. **7j**, Bar plots showing flow cytometric

results of immune cell infiltrations in the BALF of CA-induced mice receiving i.n. LNP-ASO-1 intervention.

Reviewer #3 (Remarks to the Author):

This manuscript identifies TDP-43 as a novel epithelial-intrinsic regulator of asthma pathogenesis via suppression of nonsense-mediated decay (NMD). The authors propose that allergen-induced TDP-43 expression in airway epithelial cells inhibits UPF1-mediated RNA quality control, leading to accumulation of pathogenic transcripts such as CHRM1 and promoting airway inflammation. They further show that TDP-43 facilitates influenza A virus (IAV) replication, and that intranasal delivery of antisense oligonucleotides (ASO) encapsulated in lipid nanoparticles (LNPs) reduces asthma-like pathology in mice.

The study is mechanistically intriguing, linking RNA surveillance to airway mucosal biology. However, several key mechanistic and interpretive issues need to be addressed.

Response: We deeply appreciate the reviewer's acknowledgment of our study's scientific significance and his/her valuable professional comments. We have carefully considered each point, performed extensive experiments where needed, and have addressed all questions. These revisions have been carefully integrated into the revised manuscript, significantly enhancing its overall scientific rigor and clarity.

Major Comments

1. Mechanistic link between TDP-43 and UPF1 repression is not established. Please provide direct evidence of regulation (e.g., RIP-qPCR, ChIP, or promoter assay).

Response: Thank you for the insightful comment. While correct nuclear localization of TDP-43 is required for its physiological functions in RNA metabolism, cellular stress induces the abnormal formation of its RNA-depleted nuclear condensates of TDP-43, directly disrupting RQC[12, 13].

We proposed that allergen-induced airway perturbations would disrupt the normal nuclear distribution of TDP-43 in AECs and this could ultimately contribute to the initiation of allergic inflammation. Indeed, we found that in allergen-induced asthmatic AECs, the nuclear distribution of TDP-43 was dispersed into several smaller condensates (Response Figure 16: 1l, 1m). Meanwhile, markedly elevated Tdp-43 but decreased Upf1 expression was observed in AECs from allergen-induced asthmatic mice (Response Figure 16: 1i). We also observed that UPF1 was downregulated upon TDP-43 overexpression in BEAS-2B cells (Response Figure 16: 4f, 4g). Given the fact that TDP-43 autoregulates its mRNA level in an exosome-dependent fashion[14], the accumulation of TDP-43 in asthmatic AECs may result from the breakdown of this autoregulatory feedback loop caused by allergen-induced alteration of nuclear distribution of TDP-43.

In intron6 of *UPF1*, there are 4 tandem UG repeats for potential binding of TDP-43[15] (Response Figure 16: S4g), which was further verified by performing RNA immunoprecipitation coupled with quantitative real-time polymerase chain reaction (RIP-qPCR) with anti-TDP-43 antibody on BEAS-2B cells (Response Figure 16: 4j, k).

In mammals, *UPF1* has two isoforms derived from conserved alternative 5’ splice site usage in exon7, generating *UPF1* with different regulatory loop lengths (*UPF1*$_{SL}$ and *UPF1*$_{LL}$ with 33-nt longer) (Response Figure 16: S4h)[16]. Under steady state, *UPF1*$_{LL}$ accounts for only 15-25% of total *UPF1* mRNA[17]. As the TDP-43-binding site in intron6 is in proximity to the alternative 5’ splice site in exon7, we wondered whether excess TDP-43 expression could regulate alternative splicing of *UPF1*. We performed minigene splicing assay by subcloning the partial intron6-exon7-intron7 region of *UPF1* into the pET01 construct, flanked by exons A and B (Response Figure 16: S4g)[18]. The minigene construct was transfected into BEAS-2B cells for 24 hours and the splicing patterns of the inserted *UPF1* genomic segment were analyzed by RT-PCR using indicated primers (Response Figure 16: S4g). The minigene splicing analysis demonstrated that both the short (331-nt) and long (364-nt) isoforms were detectable in BEAS-2B cells, with the short isoform predominating (Response Figure 16: 4o). Overexpression of TDP-43 enhanced the inclusion of the 33-nt fragment, thereby increasing the proportion of the long isoform (please refer to Response Figure 16: 4o,p).

Response Figure 16

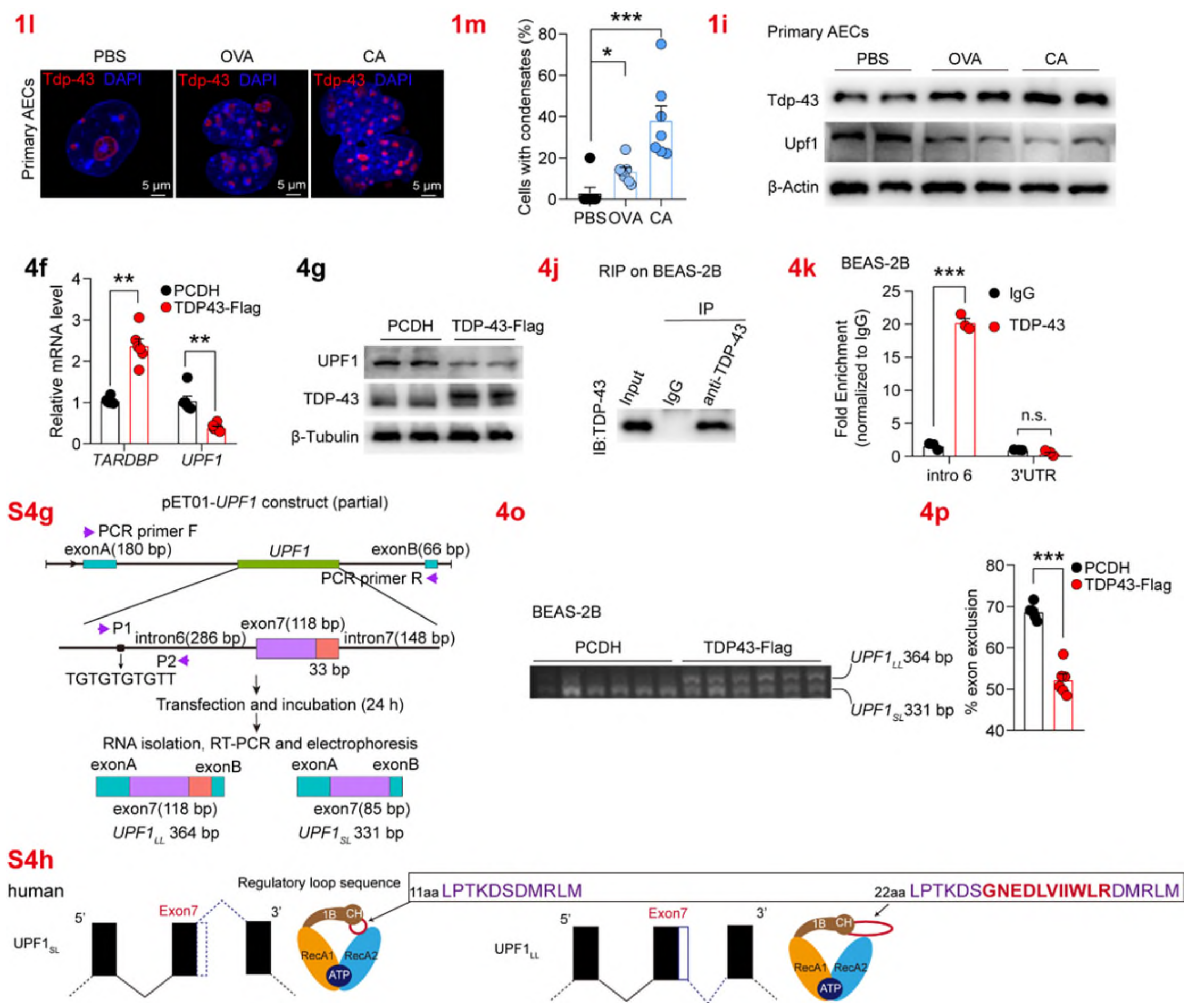

Response Figure 16 | 1l, Representative IF images of Tdp-43 in the AECs isolated from CA/OVA-induced mice and control mice. **1m**, Quantify the percentage of cells containing small Tdp-43 condensates in (**1l**). **1i**, Immunoblotting of Tdp-43 and Upf1 in the AECs isolated from CA/OVA-induced asthmatic mice and control. **4f**, mRNA levels of *TARDBP* and *UPF1* in TDP-43-overexpressed BEAS-2B cells. **4g**, Immunoblotting of TDP-43 and UPF1 in TDP-43-overexpressed BEAS-2B cells. **4j**, Immunoblotting of TDP-43 after RIP assay with TDP-43 antibody in BEAS-2B cells. **4k,** qPCR analysis of RNA pulled down by TDP-43 antibody in BEAS-2B cells, using primers targeting the intron 6 or 3'UTR of *UPF1*. **S4g,** Schematic diagram of the assay for UPF1 alternative splicing. **S4h,** A schematic diagram comparing the differences between the two transcript isoforms of gene UPF1. **4o**, Agarose gel separation of long

and short minigene splicing variants in RT-PCR products. **4p**, Quantitative results of long and short variants in (4o).

2. Specificity of airway epithelial TDP-43 deletion needs further validation. Validation with FoxJ1-Cre (ciliated cell–specific) or Krt5-CreER (basal) models would strengthen the claim that epithelial TDP-43 is the key pathogenic driver. Or Single-cell RNA-seq demonstrating TDP-43 ablation across epithelial subsets would also be convincing.

Response: Thank you for the constructive comment. In a previous elegantly performed lineage tracing study, Rawlins *et al* demonstrated that Scgb1a1/Cc10^{+} club cells significantly contribute into the long-term maintenance and repair of airway epithelium but not alveolar epithelium[17]. To further assess the target cell specificity of *Scgb1a1-Cre* mice, we conducted single-cell RNA sequencing (scRNA-seq) using lung tissues from *Tardbp*$^{\Delta Scgb1a1}$ and *Tardbp*^{flox} mice. In *Tardbp*$^{\Delta Scgb1a1}$ mice, *Tardbp* expression was affected specifically in club cells and ciliated cells but remained intact in basal cells and alveolar epithelium (Response Figure 17: 2d, e), consistent with the findings of Rawlins *et al*[17].

For the scRNA-seq data, we comprehensively compared different cell compartments between OVA- *Tardbp*$^{\Delta Scgb1a1}$ and OVA-*Tardbp*^{flox}.

- **An alleviation of airway metaplasia in the OVA-*Tardbp*^{flox} group:** secretory-pro, club, and alveolar cells isolated from OVA-*Tardbp*$^{\Delta Scgb1a1}$ mice exhibited a reduced capacity of mucin secretion compared to those from OVA-*Tardbp*^{flox}

mice (lower expression of *Muc5ac*, *Spdef*, *Tff2*, *Fcgbp*, *Clca1*) (Response Figure 17: 2d, S3e, 2e).

- Notably reduced mast cell infiltrations (*Fcer1g* and *Mcpt8*) were observed in the lungs of OVA-*Tardbp*$^{\Delta Scgb1a1}$ mice (Response Figure 17: S3f, 2g). Furthermore, mast cells from OVA-*Tardbp*$^{\Delta Scgb1a1}$ group also under-expressed genes related to granulocyte inflammation (*Tnf*, *Il13*, *Il1rl1*, *Il6*, *S100a6*, *Serpinb1a*, *F2r*, *P2rx4*) (Response Figure 17: 2h). The lungs of OVA-*Tardbp*$^{\Delta Scgb1a1}$ mice exhibited an increased abundance of M2-like macrophages, which possess immunomodulatory and tissue-repair properties (*Mertk*, *Chil3*, *Arg1*, *Mrc1*) (Response Figure 17: S3f, 2g).
- **The acquisition of the Th2-like program in T cells was significantly blunted in the OVA-*Tardbp*$^{\Delta Scgb1a1}$ mice**. Scart1^{+} γδT17 cells are mucosal-resident lymphocytes characterized by a Th17-like transcriptional signature that is essential for maintaining mucosal homeostasis[37]. In OVA-*Tardbp*^{flox} mice, this population underwent a trans-differentiation from its steady-state mucosal-γδT17 identity (marked by lower expression of *Sox13*, *Rorc*, *Scart1*, *Il17re*, and *Il17a*) toward an activated, Th2-like state, evidenced by elevated expression of *Cd69*, *Il2ra*, *Gata3*, *Il1rl1*, *Klrg1* and *Runx1*(Response Figure 17: S3g, 2i, 2j)[38]. Interestingly, a population of immature γδT cells (imm-γδT) (*Dntt*$^{+}$*Rag1*$^{+}$*Ptcra*$^{+}$) was specifically detected in the lungs from OVA-*Tardbp*^{flox} mice (Response Figure 17: S3g, 2i). Notably, both these imm-γδT cells and naïve T cells in OVA-*Tardbp*^{flox} mice similarly acquired a Th2-like program (Fig. 2j). In sharp contrast,

the acquisition of the Th2-program, observed across Scart1^{+}γδT17, imm-γδT and naïve T cells in OVA-*Tardbp*^{flox} mice, was blunted in the OVA-*Tardbp*$^{\Delta Scgb1a1}$ mice Response Figure 17: 2j).

Taken together, the scRNA-seq analysis revealed that, upon allergen exposure, *Tardbp*$^{\Delta Scgb1a1}$ mice exhibited an attenuated asthmatic airway response compared with *Tardbp*^{flox} mice.

Response Figure 17

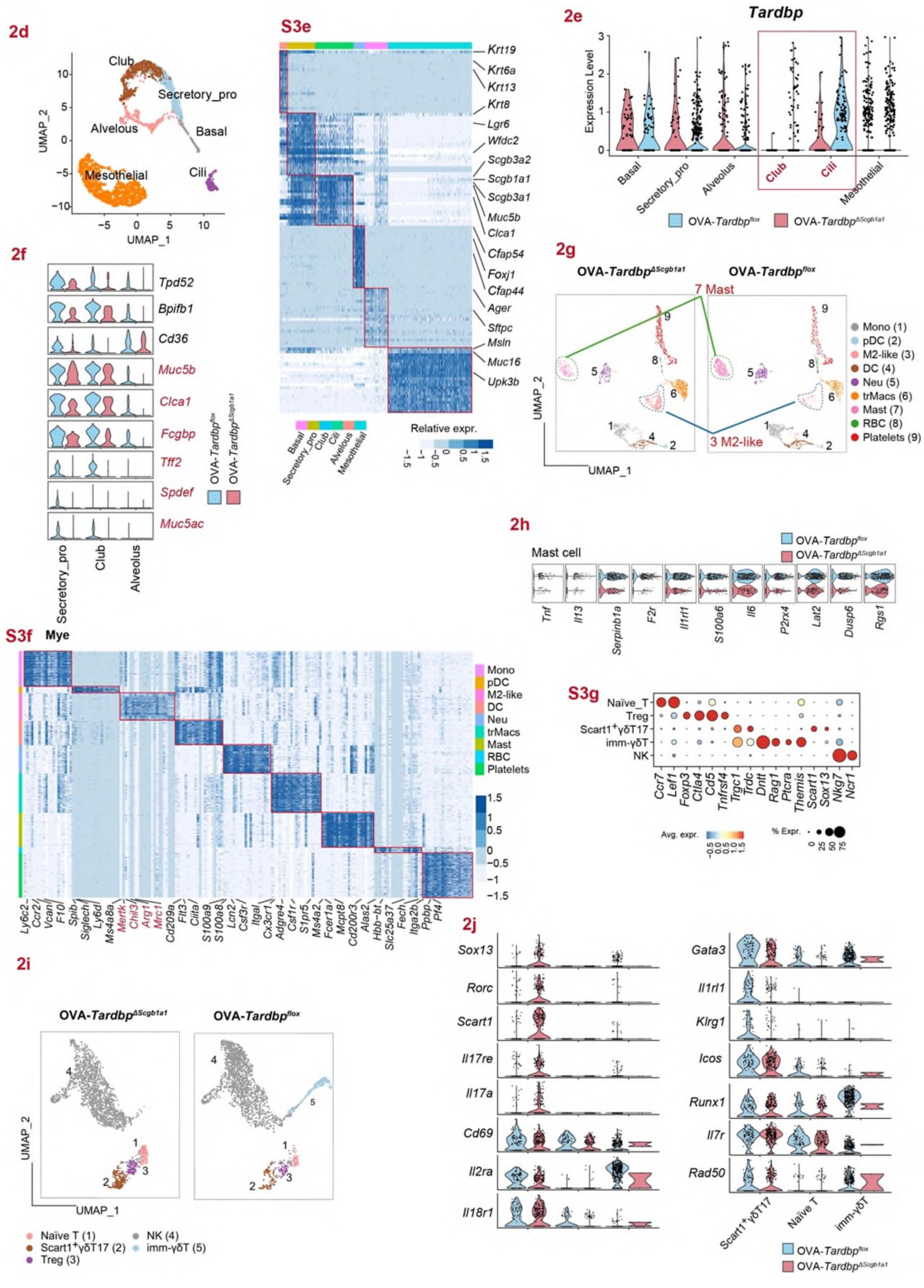

Response Figure 17 | **2d,** UMAP plot showing the cell types of epithelium were displayed separately according to OVA-stimulated *Tardbp*$^{\Delta Scgb1a1}$ and *Tardbp*^{flox} mice. **S3e,** Heatmap showing expression of the top 20 differential genes in multiple distinct epithelial cell types. Each column represents an individual cell. **2e**, Violin plot showing

the expression of *Tardbp* between OVA-stimulated *Tardbp*$^{\Delta Scgb1a1}$ and *Tardbp*^{flox} mice in epithelial cell types. **2f**, Violin plot showing the expression of gene about mucin secretion in epithelial cell types. **2g**, UMAP plot showing the cell types of myeloid cells were displayed separately according to OVA-stimulated *Tardbp*$^{\Delta Scgb1a1}$ and *Tardbp*^{flox} mice. **S3f,** Heatmap showing expression of the top 20 differential genes in different myeloid cell types. Each row represents an individual cell. **2h**, Violin plot showing the expression of gene related to granulocyte inflammation in mast cell. **2i,** UMAP plot showing the cell types of T cells were displayed separately according to OVA-stimulated *Tardbp*$^{\Delta Scgb1a1}$ and *Tardbp*^{flox} mice. **S3g,** Dotplots showing expression level of marker genes of various cell types in B cells and T cells. **2j,** Violin plot showing the expression of gene related to type 2 inflammatory stimulation in T cell types.

3. The AAV-Chrm1 rescue experiment may not be epithelium-specific. Quantify transgene expression in airway vs non-airway tissues and test pharmacologic antagonists.

Response: Thank you for raising this concern. In the AAV construct, Chrm1 expression was directed by the promotor of *Scgb1a1*/*Cc10* and the C-terminal of Chrm1 was Flag tagged (Chrm1-Flag). To evaluate the cell-type specificity of ectopically expressed Chrm1-Flag in the airways, we performed co-IF staining for the Flag tag and the epithelial marker E-cadherin on lung sections from mice following intranasal AAV-Chrm1 infection. For quantitative analysis, Chrm1-Flag signals co-localizing with E-cadherin were identified as epithelium-specific, while those that did not co-localize

were classified as non-epithelial.

Across examined fields, we consistently observed robust co-localization of Chrm1-Flag and E-cadherin signals (Response Figure 18: S5i). Quantitative analysis confirmed a marked enrichment, with Chrm1-Flag signal intensity in E-cadherin positive regions exceeding that in E-cadherin negative regions by approximately 30-fold (Response Figure 18: S5j), confirming the specificity of Chrm1-Flag expression in the airway epithelium.

Response Figure 18

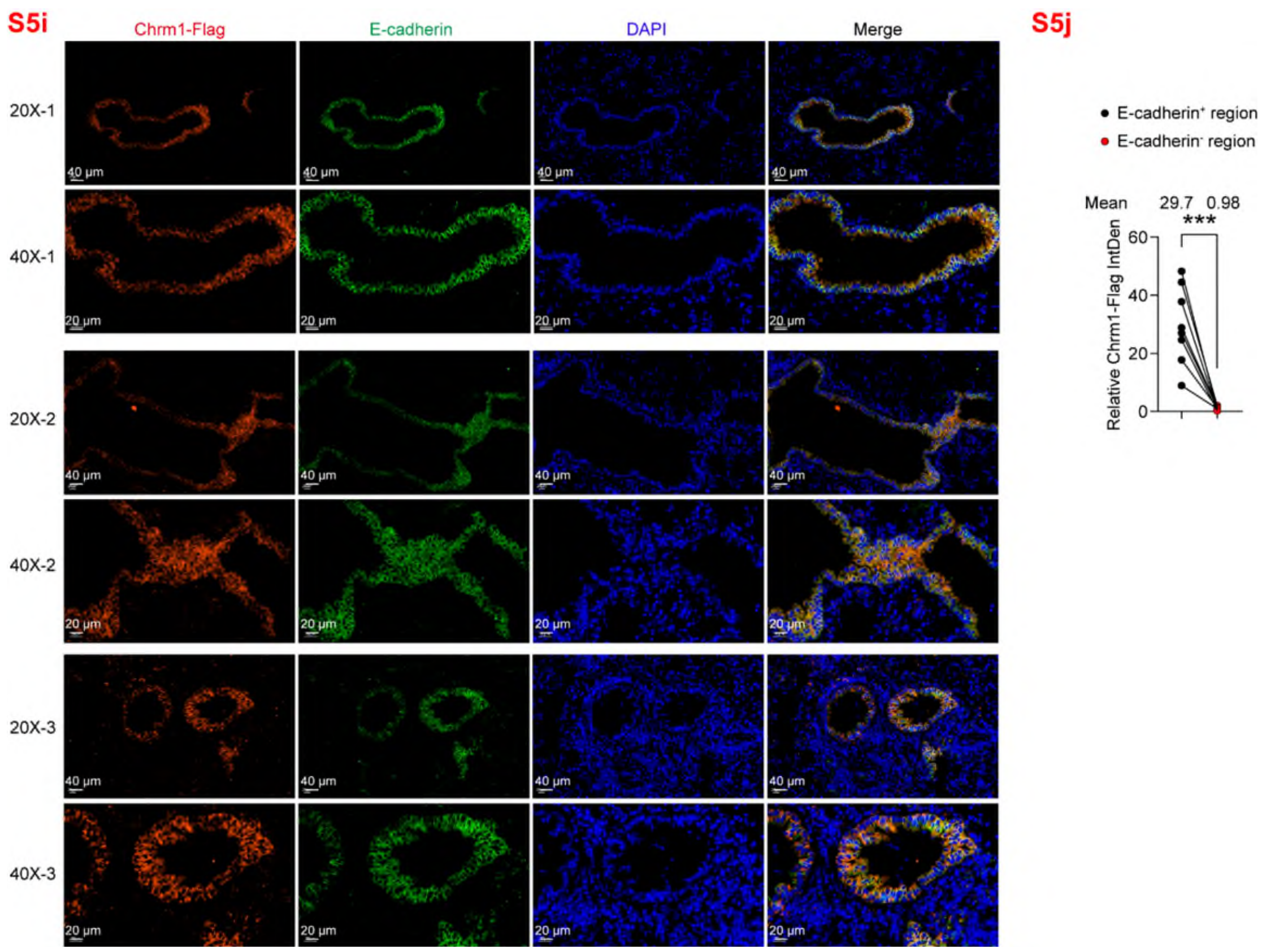

Response Figure 18 | **S5i,** Representative IF images of Flag and E-cadherin in lungs from OVA-induced *Tardbp*$^{\Delta Scgb1a1}$ and *Tardbp*^{flox} mice receiving i.n. infection of AAV-Ctrl or AAV-Chrm1. **S5j,** Bar plots comparing the Integrated Density of Flag signals between E-cadherin positive regions and E-cadherin negative regions.

Regarding the use of pharmacological antagonists of Chrm1, we agree that they represent a powerful approach for elucidating receptor function and have given serious consideration to your suggestion.

However, a primary challenge in interpreting *in vivo* inhibition studies with Chrm1 antagonists in an allergen-induced asthma model arises from the cellular pleiotropy of Chrm1 expression. Chrm1 is expressed not only in AECs but also in neuronal ganglia (where it may amplify cholinergic signaling)[41], as well as in various immune cells (T cells, B cells, mast cells, neutrophils, eosinophils and macrophages/monocytes)[1, 2]. Consequently, administration of a Chrm1 antagonist would likely affect all these cell types simultaneously. The resulting phenotypes, such as alterations in airway inflammation, would therefore reflect a complex aggregate of effects, making it difficult to dissect the specific contribution originating from the AEC compartment. This confounding factor could obscure the precise epithelial role that is the focus of our investigation.

4. Link between epithelial TDP-43/CHRM1 and Th2 activation is unclear. Examine whether TDP-43 or CHRM1 alters epithelial alarmins (IL-33, TSLP, IL-25) that drive Th2/ILC2 responses.

Response: Thank you for the insightful comment. Our RNA-seq data on mouse primary AECs showed that Tslp expression was significantly upregulated in AECs from OVA-*Tardbp*^{flox} mice compared to those from PBS-*Tardbp*^{flox} mice. TSLP is a key epithelial

alarmin that activates ILC2s by binding to its receptor complex composed of TSLPR and IL-7Rα[33]. However, *Tslp* expression in AECs from OVA-$Tardbp^{\Delta Scgb1a1}$ mice was significantly downregulated compared to those from OVA-$Tardbp^{flox}$ mice (Response Figure 19**:** A). Consistent results were obtained for the two alarmin genes *Il25* and *Tslp* in both lung tissues and purified primary AECs from a CA-induced asthma model, as measured by qPCR (Response Figure 19: 3g, h). These reductions were also recapitulated *ex vivo*, where ALI cultures derived from steady-state $Tardbp^{\Delta Scgb1a1}$ mice showed diminished *Il33*, *Il25* and *Tslp* induction upon Il13 challenge compared to ALIs derived from $Tardbp^{flox}$ mice (Response Figure 19: 3i). These findings indicate that dysregulated TDP-43 drives asthmatic airway responses by inducing the expression of alarmins in AECs.

Response Figure 19

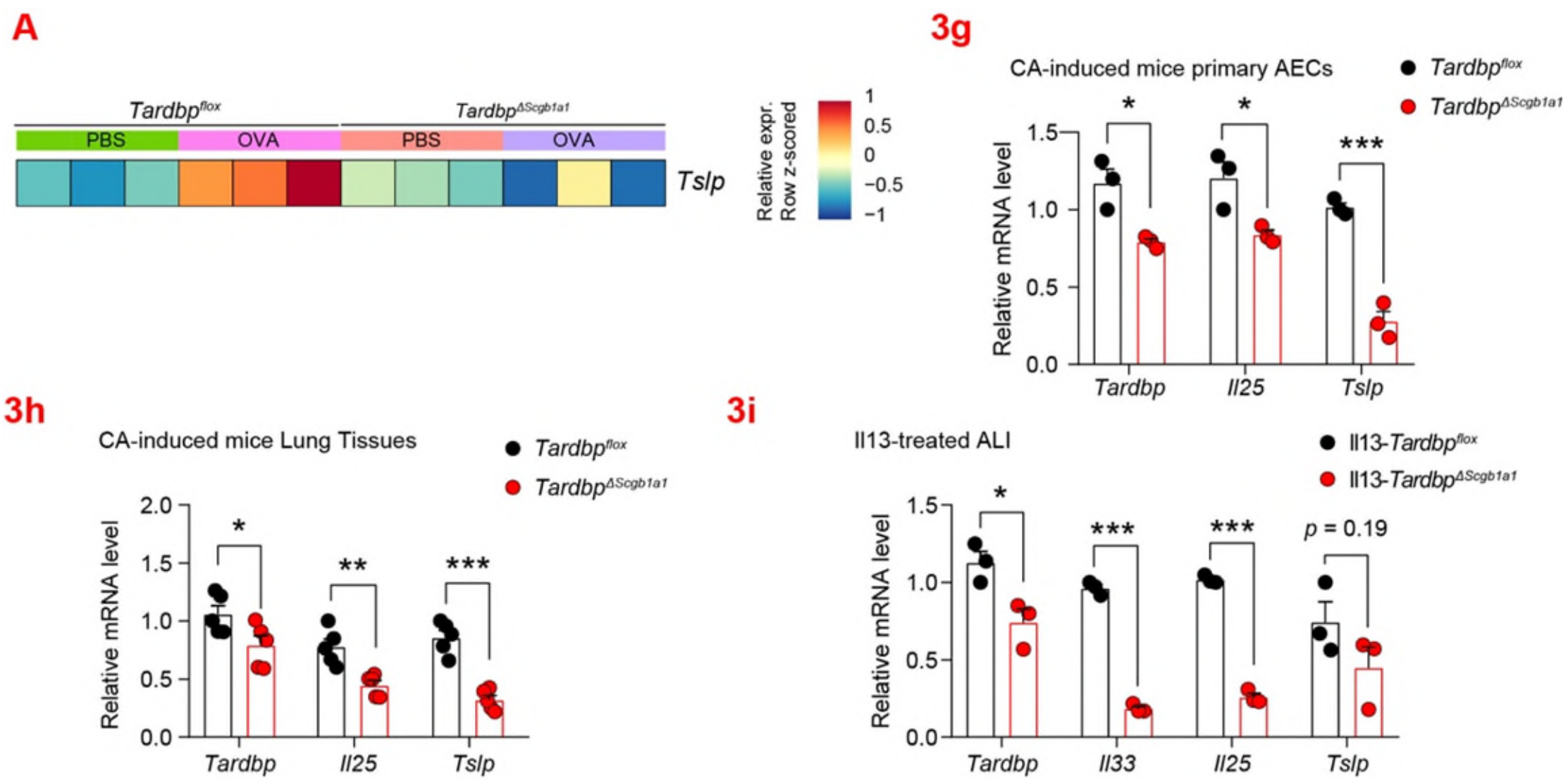

Response Figure 19 | **A,** Heatmap showing expression of *Tslp*, *Tnf*, *Lif* on mouse primary AECs from allergen-stimulated $Tardbp^{\Delta Scgb1a1}$ mice and $Tardbp^{flox}$ mice. **3g-h,** mRNA levels of *Tardbp*, *Il25* and *Tslp* expression in purified primary AECs (**3g**) or lung

tissues (**3h**) from a CA-induced asthma model. **3i**, Comparison of mRNA levels of *Tardbp, Il33, Il25* and *Tslp* between Il13-treated ALIs derived from *Tardbp*$^{\Delta Scgb1a1}$ and *Tardbp*^{flox} mice.

5. NMD dysregulation beyond CHRM1 is not explored. Analyze other NMD-sensitive transcripts to broaden mechanistic insight.

Response: Thank you very much for the insightful comments. Airway allergic inflammation can be triggered by a wide array of allergens, including pollens, house dust mites, cockroach antigen and mould spores, which share little structural or biological commonality[21]. Despite their diversity, these allergens share the capacity to induce airway barrier perturbations. It remains unclear how AECs sense and translate these tissue perturbations to initiate the asthmatic airway response. In this study, we proposed that allergen-exposure elicits cellular stress in AECs, which in turn impairs UPF1-mediated NMD activity, leading to uncontrolled accumulation of target transcripts of NMD, which encode pathogenic molecules transducing signals in allergen-induced asthmatic airway response. We agree that analyzing other NMD-sensitive transcripts would be helpful to broaden mechanistic insight. In addition to *CHRM1*, we investigated *ATF4* and *MAPK14.*

- ***ATF4*:** ATF4 is a master transcription factor in endoplasmic reticulum (ER) stress. Accumulating evidence indicates that ER stress is closely associated with asthmatic airway response, as evidenced in both allergen-induced murine models of asthma and airway epithelial brushing samples from asthmatic patients[6, 7, 42, 43].

Bioinformatic analysis revealed that *ATF4* has several uORFs in 5'UTR (Response Figure 20: S6a). ATF4 is a well-documented NMD target[9, 44]. Preclinical therapeutic studies showed that suppression of ATF4-involved ER stress significantly diminished asthmatic airway response[8, 45, 46]. Accumulated ATF4 expression was observed upon UPF1 knockdown in BEAS-2B cells (Response Figure 20: S6b). In CA-treated mouse lung tissues, we observed a significant upregulation of ATF4 signal compared to control lung tissues (Response Figure 20: S6c,d). Furthermore, *Tardbp*$^{\Delta Scgb1a1}$ mouse lungs showed a marked downregulation of ATF4 relative to *Tardbp*^{flox} controls (Response Figure 20: S6e,f). In primary AECs isolated from CA-stimulated *Tardbp*$^{\Delta Scgb1a1}$ mice, *ATF4* mRNA level was also significantly downregulated compared to control (Response Figure 20: S6g). *TARDBP*-knockdown in BEAS-2B cells markedly reduced ATF4 expression at both mRNA and protein level (Response Figure 20: S6h,i); conversely, *TARDBP* overexpression significantly increased *ATF4* expression (Response Figure 20: S6j).

Response Figure 20

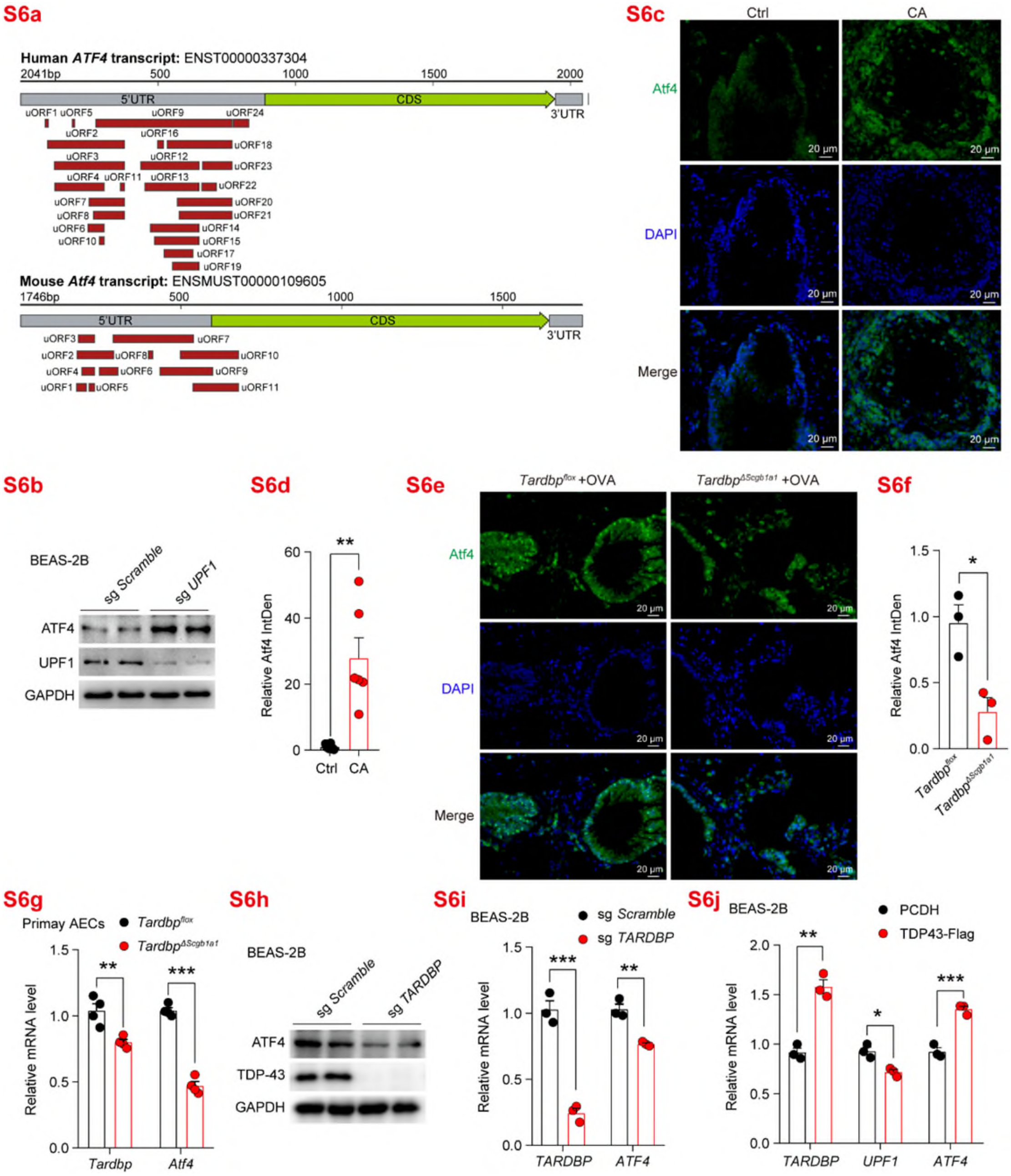

Response Figure 20 | **S6a**, The locations of potential uORFs with strong Kozak strength in human and mouse *Atf4* transcripts. **S6b,** Immunoblotting of ATF4 and UPF1 in UPF1-knockdown BEAS-2B cells. **S6c,** Representative IF images of Atf4 in CA-treated mouse lung tissues and control. **S6d,** Bar plots comparing the Integrated Density of Atf4 signals in (**S6c**). **S6e,** Representative IF images of Atf4 in OVA-induced *Tardbp*$^{\Delta Scgb1a1}$ and *Tardbp*^{flox} mouse lungs. **S6f,** Bar plots comparing the Integrated Density of Atf4 signals in (**S6e**). **S6g,** mRNA levels of *Tardbp* and *Atf4* in primary AECs

isolated from CA-induced $Tardbp^{\Delta Scgb1a1}$ and $Tardbp^{flox}$ mice. **S6h,** Immunoblotting of ATF4 and TDP-43 in TDP-43-knockdown BEAS-2B cells. **S6i,** mRNA levels of *TARDBP* and *ATF4* in TDP-43-knockdown BEAS-2B cells. **S6j,** mRNA levels of *TARDBP*, *UPF1* and *ATF4* in TDP-43-overexpressed BEAS-2B cells.

- ***MAPK14***: *MAPK14* encodes p38a that plays important roles in allergic airway inflammation[10, 11]. Bioinformatic analysis identified several uORFs within the 5'UTR of *MAPK14* (Response Figure 21: A). RNA decay results showed *MAPK14* mRNA stability increased upon TDP-43 overexpression in BEAS-2B cells (Response Figure 21: B). *MAPK14* expression level was significantly decreased in BEAS-2B cell with TDP-43 knockdown (Response Figure 21: C).

- ***ATF3***: *ATF3* is another NMD target[9]. *Atf3* expression was markedly downregulated in AECs from CA-induced $Tardbp^{\Delta Scgb1a1}$ mice (Response Figure 21: D), while in BEAS-2B cells with TDP-43 overexpression, *UPF1* expression was decreased but ATF3 expression was increased (Response Figure 21: E).

Response Figure 21

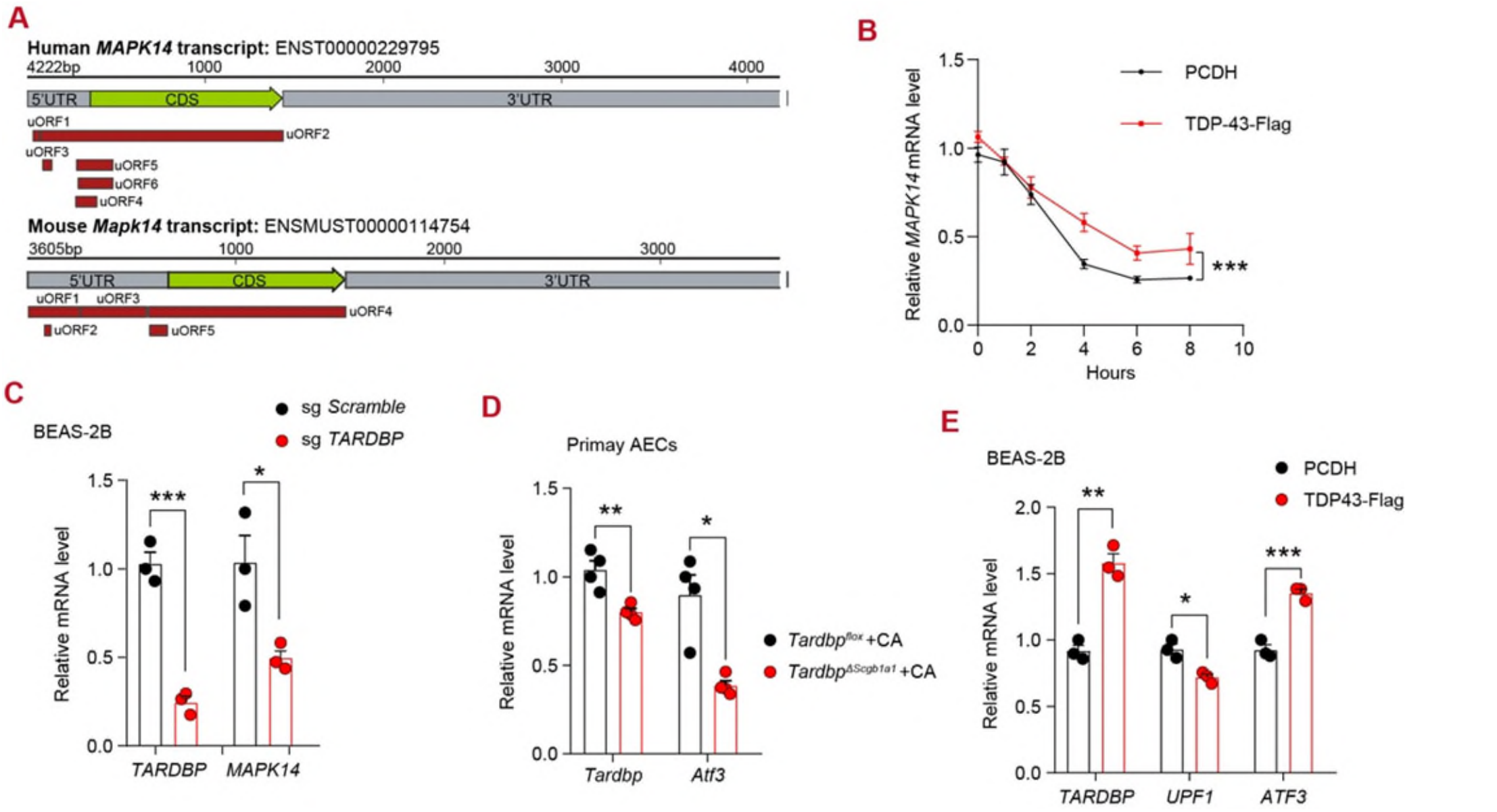

Response Figure 21 | *MAPK14* and *ATF3* are NMD targets. **A,** The locations of potential uORFs with strong Kozak strength in human and mouse *MAPK14* transcripts. **B**, mRNA level of *MAPK14* in TDP-43-overexpressed BEAS-2B cells treated with 10 μg/ml Actinomycin D for 1 h, 2 h, 4 h, 6 h and 8 h. **C**, mRNA levels of *TARDBP* and *MAPK14* in TDP-43-knockdown BEAS-2B cells. **D**, mRNA levels of *Tardbp* and *Atf3* in primary AECs isolated from *Tardbp*$^{\Delta Scgb1a1}$ and *Tardbp*^{flox} mice. **E**, mRNA levels of *TARDBP*, *UPF1* and *ATF3* in TDP-43-overexpression BEAS-2B cells.

Taken together, these results demonstrate that allergen-exposure impairs NMD activity in AECs, leading to uncontrolled accumulation of NMD-sensitive transcripts including *CHRM1*, *ATF4* and *MAPK14*, which encode pathogenic molecules mediating asthmatic airway response to allergens.

We placed greater emphasis on *CHRM1* for the following considerations:

✓ Close association of CHRM1 to the vagal neurotransmitter Ach, the active player in asthma development.

- ✓ Exact role of CHRM1 on AECs during asthmatic airway response has not been previously reported.
- ✓ *CHRM1*'s status as a target of NMD remained unreported in the literature.

6. Discussion lacks mechanistic integration. Expand on how TDP-43–NMD suppression fits within epithelial stress or immune-regulatory pathways. Broader context could be strengthened. Consider linking epithelial TDP-43 biology to its known functions in neurodegeneration or systemic RNA surveillance.

Response: We really appreciate the reviewer for the constructive comments on discussion writing. We have fully considered your suggestions, investigated the cutting-edge developments of TDP-43 biology in neurodegenerative disorders, and integrated our findings on allergen-induced type 2 innate immune responses in the airways. These elements have been carefully incorporated into the revised manuscript. The updated discussion section is provided below:

"**TDP-43 may act as a stress sensor in AECs to detect tissue perturbations elicited by allergen exposure**. The divergence between T2 and type I/III (T1/3) immunity originates at the initial stage of allergen or pathogen sensing [6, 45]. T1/T3 responses are primed by innate inflammation triggered when pattern recognition receptors (PRRs) detect conserved pathogen-associated molecular patterns (PAMPs), whereas the innate inflammation elicited by structurally diverse allergens is thought to operate through a distinct mechanism: by inducing common tissue perturbations, such

as epithelial membrane perforation and Ca^{2+} influx[46], that activate cellular stress in mucosal epithelial cells. This highlights a critical, unanswered question: how the cellular stress induced by tissue perturbations is sensed and translated into a T2 innate immune response in AECs during the initiation of allergen-induced asthma. In this study, we identify TDP-43 as a key sensor for this cellular stress. Firstly, the nuclear distribution of TDP-43 may be disrupted by cellular stress, leading to the abnormal formation of its RNA-depleted nuclear condensates or cytoplasmic aggregation as reported in neuron cells[19, 20]. Normally, TDP-43 maintains homeostasis by binding to its own 3'UTR to autoregulate its protein levels, however, under the circumstances of neurodegenerative disorders, nuclear mislocalization of TDP-43 breakdowns this autoregulation loop, leading to uncontrolled accumulation of TDP-43 [15]. It shall be similar in asthmatic airway epithelium, wherein TDP-43 expression level is significantly increased. Secondly, dysregulated TDP-43 level further suppresses UPF1 expression by directly destabilizing *UPF1* transcripts, leading to dampened NMD activity and subsequent accumulation of NMD-sensitive transcripts, which encode pathogenic molecules, such as CHRM1, MAPK14/p38a, ATF4. The final step is the execution of the type 2 innate inflammatory response: ATF4 initiates a transcriptional program that facilitates IL-33 maturation and release[7, 47]; p38α activates downstream signaling to drive the expression of type 2 inflammatory genes; and CHRM1 transduces ACh signaling.

TDP-43 restrains NMD activity by regulating alternative splicing and stability of *UPF1* transcripts. In neuron cells with TDP-43 loss, NMD is activated to degrade

RNAs containing PTCs introduced by aberrant cryptic exons[48]. These cryptic exons become fully detectable only when NMD is inhibited[49]. This indicates that UPF1 expression or activity is not reduced, and may even be enhanced, by TDP-43 loss. Meanwhile, overexpression of wildtype TDP-43 in rodent neurons impair UPF1-mediated NMD and leads to cytotoxicity, which can be rescued by co-overexpression of UPF1[21, 22]. In consistency, we also observed that TDP-43 overexpression suppresses UPF1 expression, and *UPF1* transcripts are destabilized in AECs with allergen exposure. To date, the underlying mechanism by which TDP-43 suppresses UPF1 expression is unknown. For the first time, we revealed that TDP-43 directly binds to *UPF1* transcript and regulate its alternative splicing. It can be speculated that under physiological conditions, AECs with proper expression level and correct nuclear distribution of TDP-43 may be recruited to the $(UG)_4$ site and prevent the inclusion of the so-called 33-nt cryptic exon, thereby generating $UPF1_{SL}$. In contrast, in allergen-induced AECs, dysregulated nuclear distribution and expression of TDP-43 might disrupt this splicing regulation, leading to the generation of $UPF1_{LL}$. The regulatory mechanism between TDP-43 and *UPF1* in asthmatic airway response may also be relevant to the neuropathological field."

Minor Comments

1. The Abstract slightly overstates therapeutic claims; suggest changing to "ameliorated asthma-like pathology in mice."

Response: Thank you very much for pointing this out. We have changed the expression

into “Intranasal application of lipid nanoparticles carrying antisense oligonucleotides targeting TDP-43 effectively ameliorated asthma-like pathologies in mice” in the Abstract accordingly.

2. Indicate sex and age of animals in all experiments.

Response: We appreciate the reviewer’s nice reminder. We have indicated the sex and age of animals in all experiments in the Mouse model section of Materials and Methods.

3. Specify statistical tests and adjustments for multiple comparisons.

Response: Thank you for this important comment. We have provided a detailed description of the statistical tests about every data generated by differential analysis in the figure legends.

We used post hoc Tukey multiple comparisons test in one-way ANOVA (normally distributed data), and Dunn's multiple comparisons test in Kruskal-Wallis test (non-normally distributed data) for pairwise comparisons of difference between three or more groups, and the details has been added to the Statistical analysis section of the Materials and Methods.

4. Include verification of Cre recombination efficiency and Tardbp deletion at the RNA level.

Response: Thank you very much. As suggested, we isolated primary airway epithelial cells from $Tardbp^{\Delta Scgb1a1}$ and $Tardbp^{flox}$ mice following the protocol established by

Hilaire *et al*[51]. Subsequent qPCR assessment of Tdp-43 expression confirmed its significant knockdown in AECs derived from *Tardbp*$^{\Delta Scgb1a1}$ mice compared to control mice (Response Figure 22: 2c).

Response Figure 22

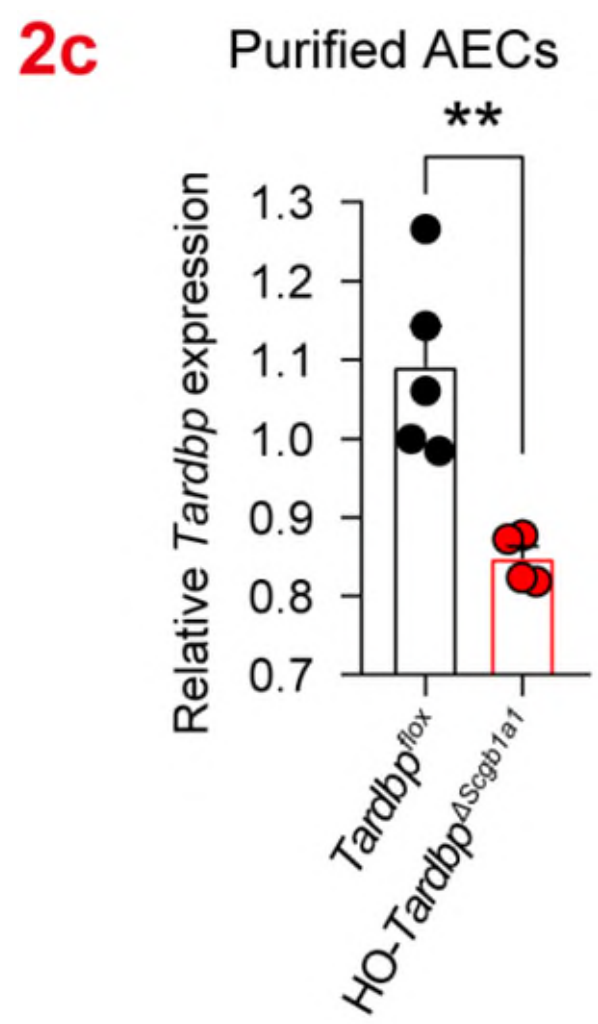

Response Figure 22 | **2c**, mRNA level of *Tardbp* in primary airway epithelial cells from *Tardbp*$^{\Delta Scgb1a1}$ and *Tardbp*^{flox} mice.

References

1. Davis BE, Cropper KJ, Cockcroft DWC. Muscarinic Receptor Antagonism and Allergen Induced Airway Responses in Allergic Asthma: A Scoping Review. *Clin Invest Med* **45**, E10-20 (2022).
2. Gosens R, Zaagsma J, Meurs H, Halayko AJ. Muscarinic receptor signaling in the pathophysiology of asthma and COPD. *Respir Res* **7**, 73 (2006).
3. Wang Y, Wang J, Yan Z, Liu S, Xu W. Potential drug targets for asthma identified in the plasma and brain through Mendelian randomization analysis. *Front Immunol* **14**, 1240517 (2023).
4. Metzen J, Bittinger F, Kirkpatrick CJ, Kilbinger H, Wessler I. Proliferative effect of acetylcholine on rat trachea epithelial cells is mediated by nicotinic receptors and

muscarinic receptors of the M1-subtype. *Life Sci* **72**, 2075-2080 (2003).

5. Koyama S, Rennard SI, Robbins RA. Acetylcholine stimulates bronchial epithelial cells to release neutrophil and monocyte chemotactic activity. *Am J Physiol* **262**, L466-471 (1992).

6. Bhakta NR, *et al.* IFN-stimulated Gene Expression, Type 2 Inflammation, and Endoplasmic Reticulum Stress in Asthma. *Am J Respir Crit Care Med* **197**, 313-324 (2018).

7. Hoffman SM, *et al.* Endoplasmic reticulum stress mediates house dust mite-induced airway epithelial apoptosis and fibrosis. *Respir Res* **14**, 141 (2013).

8. Nakada EM, *et al.* Conjugated bile acids attenuate allergen-induced airway inflammation and hyperresponsiveness by inhibiting UPR transducers. *JCI Insight* **4**, (2019).

9. Mendell JT, Sharifi NA, Meyers JL, Martinez-Murillo F, Dietz HC. Nonsense surveillance regulates expression of diverse classes of mammalian transcripts and mutes genomic noise. *Nat Genet* **36**, 1073-1078 (2004).

10. Duan W, *et al.* Inhaled p38alpha mitogen-activated protein kinase antisense oligonucleotide attenuates asthma in mice. *Am J Respir Crit Care Med* **171**, 571-578 (2005).

11. Shi K, *et al.* Epithelial cell membrane perforation induces allergic airway inflammation. *Nature* **645**, 475-483 (2025).

12. Huang WP, *et al.* Stress-induced TDP-43 nuclear condensation causes splicing loss of function and STMN2 depletion. *Cell Rep* **43**, 114421 (2024).

13. Perez-Berlanga M, *et al.* Loss of TDP-43 oligomerization or RNA binding elicits distinct aggregation patterns. *EMBO J* **42**, e111719 (2023).

14. Ayala YM, *et al.* TDP-43 regulates its mRNA levels through a negative feedback loop. *EMBO J* **30**, 277-288 (2011).

15. Gowravaram M, *et al.* A conserved structural element in the RNA helicase UPF1 regulates its catalytic activity in an isoform-specific manner. *Nucleic Acids Res* **46**, 2648-2659 (2018).

16. Nicholson P, Josi C, Kurosawa H, Yamashita A, Muhlemann O. A novel phosphorylation-independent interaction between SMG6 and UPF1 is essential for human NMD. *Nucleic Acids Res* **42**, 9217-9235 (2014).

17. Rawlins EL, *et al.* The role of Scgb1a1+ Clara cells in the long-term maintenance and repair of lung airway, but not alveolar, epithelium. *Cell Stem Cell* **4**, 525-534 (2009).

18. Lu Y, *et al.* Eosinophil extracellular traps drive asthma progression through neuro-immune signals. *Nat Cell Biol* **23**, 1060-1072 (2021).

19. Tsai CH, *et al.* Neutrophil extracellular trap production and CCL4L2 expression influence corticosteroid response in asthma. *Sci Transl Med* **15**, eadf3843 (2023).

20. Zou X, *et al.* Hypoxia-inducible factor 2alpha promotes pathogenic polarization of stem-like Th2 cells via modulation of phospholipid metabolism. *Immunity* **57**, 2808-2826 e2808 (2024).

21. Kopp EB, Agaronyan K, Licona-Limon I, Nish SA, Medzhitov R. Modes of type 2 immune response initiation. *Immunity* **56**, 687-694 (2023).

22. Chen W, *et al.* Allergen protease-activated stress granule assembly and gasdermin D fragmentation control interleukin-33 secretion. *Nat Immunol* **23**, 1021-1030 (2022).

23. Kurosaki T, Popp MW, Maquat LE. Quality and quantity control of gene expression by nonsense-mediated mRNA decay. *Nat Rev Mol Cell Biol* **20**, 406-420 (2019).

24. Li Z, Vuong JK, Zhang M, Stork C, Zheng S. Inhibition of nonsense-mediated RNA decay by ER stress. *RNA* **23**, 378-394 (2017).

25. Martin L, Gardner LB. Stress-induced inhibition of nonsense-mediated RNA decay regulates intracellular cystine transport and intracellular glutathione through regulation of the cystine/glutamate exchanger SLC7A11. *Oncogene* **34**, 4211-4218 (2015).

26. Gardner LB. Hypoxic inhibition of nonsense-mediated RNA decay regulates gene expression and the integrated stress response. *Mol Cell Biol* **28**, 3729-3741 (2008).

27. Tziortzouda P, Van Den Bosch L, Hirth F. Triad of TDP43 control in neurodegeneration: autoregulation, localization and aggregation. *Nat Rev Neurosci* **22**, 197-208 (2021).

28. Baughn MW, *et al.* Mechanism of STMN2 cryptic splice-polyadenylation and its correction for TDP-43 proteinopathies. *Science* **379**, 1140-1149 (2023).

29. Brown AL, *et al.* TDP-43 loss and ALS-risk SNPs drive mis-splicing and depletion of UNC13A. *Nature* **603**, 131-137 (2022).

30. Neumann M, *et al.* Ubiquitinated TDP-43 in frontotemporal lobar degeneration and amyotrophic lateral sclerosis. *Science* **314**, 130-133 (2006).

31. Barmada SJ, *et al.* Amelioration of toxicity in neuronal models of amyotrophic lateral sclerosis by hUPF1. *Proc Natl Acad Sci U S A* **112**, 7821-7826 (2015).

32. Jackson KL, *et al.* Preservation of forelimb function by UPF1 gene therapy in a rat model of TDP-43-induced motor paralysis. *Gene Ther* **22**, 20-28 (2015).

33. Roan F, Obata-Ninomiya K, Ziegler SF. Epithelial cell-derived cytokines: more than just signaling the alarm. *J Clin Invest* **129**, 1441-1451 (2019).

34. Gordon ED, *et al.* Alternative splicing of interleukin-33 and type 2 inflammation in asthma. *Proc Natl Acad Sci U S A* **113**, 8765-8770 (2016).

35. Seah GT, Gao PS, Hopkin JM, Rook GA. Interleukin-4 and its alternatively spliced variant (IL-4delta2) in patients with atopic asthma. *Am J Respir Crit Care Med* **164**, 1016-1018 (2001).

36. Shilovskiy I, *et al.* Identification of a novel splice variant for mouse and human interleukin-5. *Heliyon* **6**, e03586 (2020).

37. Tan L, *et al.* Single-Cell Transcriptomics Identifies the Adaptation of Scart1(+) Vgamma6(+) T Cells to Skin Residency as Activated Effector Cells. *Cell Rep* **27**, 3657-3671 e3654 (2019).

38. Spidale NA, *et al.* Interleukin-17-Producing gammadelta T Cells Originate from SOX13(+) Progenitors that Are Independent of gammadeltaTCR Signaling. *Immunity* **49**, 857-872 e855 (2018).

39. Cunha AA, *et al.* Extracellular DNA traps in bronchoalveolar fluid from a murine eosinophilic pulmonary response. *Allergy* **69**, 1696-1700 (2014).

40. Dupont M, *et al.* The RBPome of influenza A virus NP-mRNA reveals a role for TDP-43 in viral replication. *Nucleic Acids Res* **52**, 7188-7210 (2024).

41. Sabbir MG. Cholinergic Receptor Muscarinic 1 Co-Localized with Mitochondria in Cultured Dorsal Root Ganglion Neurons, and Its Deletion Disrupted Mitochondrial Ultrastructure in Peripheral Neurons: Implications in Alzheimer's Disease. *J Alzheimers Dis* **98**, 247-264 (2024).

42. Hoffman SM, *et al.* Protein disulfide isomerase-endoplasmic reticulum resident protein 57 regulates allergen-induced airways inflammation, fibrosis, and hyperresponsiveness. *J Allergy Clin Immunol* **137**, 822-832 e827 (2016).

43. Pathinayake PS, Hsu AC, Nichol KS, Horvat JC, Hansbro PM, Wark PAB. Endoplasmic reticulum stress enhances the expression of TLR3-induced TSLP by airway epithelium. *Am J Physiol Lung Cell Mol Physiol* **326**, L618-L626 (2024).

44. Kamelgarn M, Chen J, Kuang L, Jin H, Kasarskis EJ, Zhu H. ALS mutations of FUS suppress protein translation and disrupt the regulation of nonsense-mediated decay. *Proc Natl Acad Sci U S A* **115**, E11904-E11913 (2018).

45. Liu F, Zhang H, Feng Y. SIRT6 Attenuates ER Stress-Associated Mucus Hypersecretion via Regulation of NRF2 in Asthma Models. *FASEB J* **39**, e70926 (2025).

46. Makhija L*, et al.* Chemical chaperones mitigate experimental asthma by attenuating endoplasmic reticulum stress. *Am J Respir Cell Mol Biol* **50**, 923-931 (2014).

47. Iwasaki A, Medzhitov R. Control of adaptive immunity by the innate immune system. *Nat Immunol* **16**, 343-353 (2015).

48. Xiao Y*, et al.* Endoplasmic reticulum stress drives macrophages to produce IL-33 to favor Th2 polarization in the airways. *J Leukoc Biol* **115**, 893-901 (2024).

49. Ling JP, Pletnikova O, Troncoso JC, Wong PC. TDP-43 repression of nonconserved cryptic exons is compromised in ALS-FTD. *Science* **349**, 650-655 (2015).

50. Sinha IR*, et al.* Inhibition of nonsense-mediated decay in TDP-43 deficient neurons reveals novel cryptic exons. *bioRxiv*, (2025).

51. Lam HC, Choi AM, Ryter SW. Isolation of mouse respiratory epithelial cells and exposure to experimental cigarette smoke at air liquid interface. *J Vis Exp*, (2011).

Response to reviewers' comments

Reviewer #1 (Remarks to the Author):

The authors have addressed the issues that were identified in the prior review and have added supporting data and information to the revised manuscript.

Response: We greatly appreciate your constructive comments during the review process and are delighted that you have approved our revisions.

Minor comment:

It should be noted that the data with the human epithelial cells was done with BEAS-2b cells and not with epithelial cells differentiated at ALI.

Response: Thank you very much for the nice reminder. Where necessary, we have added annotations to explicitly state whether the results were obtained using BEAS-2B cells or primary AECs differentiated under ALI.

Reviewer #2 (Remarks to the Author):

I would like to thank the authors for the additional work undertaken and data provided to address this reviewer's questions. For me there does remain several outstanding concerns:

Response: We truly appreciate your professional comments, which are very helpful in strengthening our study. We have carefully digested the comments and performed additional experiments where needed. We hope the revised manuscript will address the

concerns.

Response figure 5 (updated figure 1) – the representative image does not appear to be relate to the quantification. What is '5 independent biological repeats'? You should indicate how many donors and how many sections/fields per section were analyzed.

Response: We are grateful to the reviewer for the constructive comment. The integrated density (IntDen) of TDP-43 was quantified as per cell, and the representative image shown in (Response Figure 1: 1j and k) was selected as the one with TDP-43 IntDen per cell closest to the mean value of each group. We apologize for not providing a detailed description in the original figure and figure legend.

We collected bronchoscopic mucosal biopsies from 7 patients with asthma and 7 control subjects (from the hospital's histopathology bank) for TDP-43 staining, with one section per patient. For each section, 1~5 fields were randomly selected based on section size and staining uniformity. For quantification, the accumulated TDP-43 IntDen signals were calculated within E-CAD$^+$ cells in each field to determine the TDP-43 IntDen per cell in that specific field. For each patient, the mean value (TDP-43 IntDen per cell) across fields was then calculated. Subsequently, the group mean was calculated across subjects within the same group. The field of view with TDP-43 IntDen per cell closest to the group mean was chosen as the representative image for that group. Finally, the TDP-43 IntDen per cell for each patient were normalized to the mean value of the control group.

The detailed results of each step in the quantitative analysis were summarized in **Table 1** as following.

For clarity, we have added detailed descriptions in both the figure legend and the Human samples section of the Methods.

Table 1 Intermediate data for quantifying TDP-43 expression levels in bronchoscopic mucosal specimens using mIF detection.

<table>
<tr><th>Section</th><th>field</th><th>IntDen</th><th>Cell number</th><th>IntDen per cell</th><th>Average IntDen per section</th><th>Average IntDen per group</th><th>Normalization</th></tr>
<tr><td>Asthma-1</td><td>field-1</td><td>15615711</td><td>104</td><td>150151.07</td><td rowspan="2">112588.37</td><td rowspan="12">78909.01</td><td rowspan="2">6.495</td></tr>
<tr><td></td><td>field-2</td><td>3901335</td><td>52</td><td>75025.67</td></tr>
<tr><td>Asthma-2</td><td>field-1</td><td>3870819</td><td>104</td><td>37219.41</td><td rowspan="3">72170.64</td><td rowspan="3">4.163</td></tr>
<tr><td></td><td>field-2</td><td>3825904</td><td>38</td><td>100681.68</td></tr>
<tr><td>Representative image</td><td>field-3</td><td>3380266</td><td>43</td><td>78610.84</td></tr>
<tr><td>Asthma-3</td><td>field-1</td><td>1559359</td><td>31</td><td>50301.90</td><td rowspan="2">37509.20</td><td rowspan="2">2.164</td></tr>
<tr><td></td><td>field-2</td><td>1235825</td><td>50</td><td>24716.50</td></tr>
<tr><td>Asthma-4</td><td>field-1</td><td>4470288</td><td>137</td><td>32629.84</td><td>32629.84</td><td>1.882</td></tr>
<tr><td>Asthma-5</td><td>field-1</td><td>4588233</td><td>181</td><td>25349.35</td><td rowspan="2">39046.11</td><td rowspan="2">2.252</td></tr>
<tr><td></td><td>field-2</td><td>4166686</td><td>79</td><td>52742.86</td></tr>
<tr><td>Asthma-6</td><td>field-1</td><td>11649307</td><td>73</td><td>159579.55</td><td>159579.55</td><td>9.205</td></tr>
<tr><td>Asthma-7</td><td>field-1</td><td>9604456</td><td>70</td><td>137206.51</td><td rowspan="2">98839.34</td><td rowspan="2">5.702</td></tr>
<tr><td></td><td>field-2</td><td>5684384</td><td>94</td><td>60472.17</td></tr>
<tr><td></td><td></td><td></td><td></td><td></td><td></td><td></td><td></td></tr>
<tr><td>Ctrl-1</td><td>field-1</td><td>16471389</td><td>300</td><td>54904.63</td><td rowspan="3">31917.45</td><td rowspan="6">17335.66</td><td rowspan="3">1.841</td></tr>
<tr><td>Representative image</td><td>field-2</td><td>976295</td><td>69</td><td>14149.20</td></tr>
<tr><td></td><td>field-3</td><td>6247453</td><td>234</td><td>26698.52</td></tr>
<tr><td>Ctrl-2</td><td>field-1</td><td>4139235</td><td>125</td><td>33113.88</td><td>33113.88</td><td>1.910</td></tr>
<tr><td>Ctrl-3</td><td>field-1</td><td>741335</td><td>70</td><td>10590.50</td><td>10590.50</td><td>0.611</td></tr>
<tr><td>Ctrl-4</td><td>field-1</td><td>211926</td><td>62</td><td>3418.16</td><td>18763.07</td><td>1.082</td></tr>
</table>

	field-2	848239	116	7312.41			
	field-3	7881643	173	45558.63			
Ctrl-5	field-1	174004	112	1553.61	1081.38		0.062
	field-2	78581	129	609.16			
Ctrl-6	field-1	1643664	78	21072.62	20253.27		1.168
	field-2	920621	103	8938.07			
	field-3	3672217	175	20984.10			
	field-4	893374	64	13958.97			
	field-5	2396632	66	36312.61			
Ctrl-7	field-1	517967	92	5630.08	5630.08		0.325

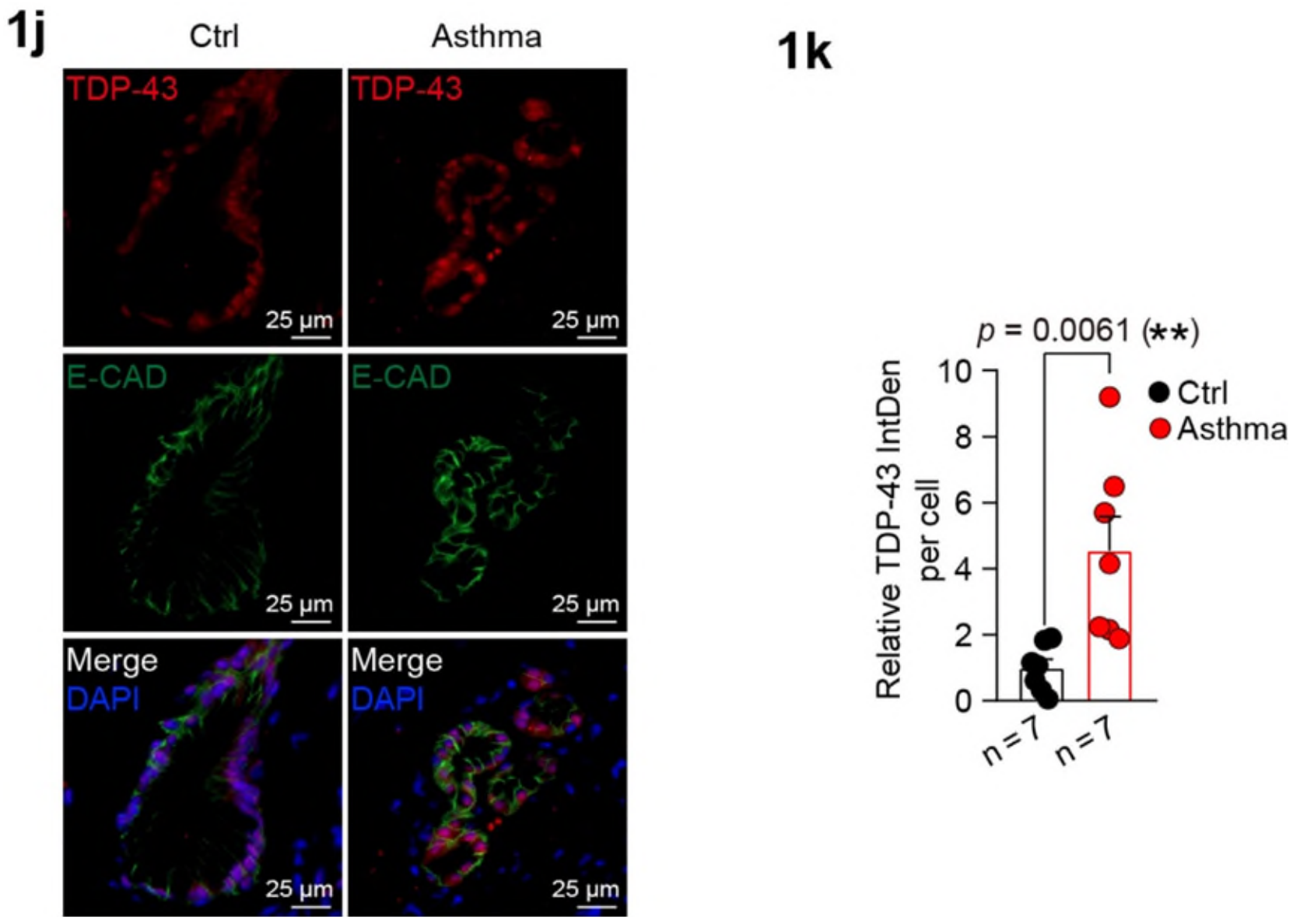

Response Figure 1 | **1j**, Representative IF staining of TDP-43 and E-cadherin in the bronchoscopic mucosal specimens from patients with asthma (n = 7) and relatively healthy subjects (n = 7). **1k**, Quantitative results of TDP-43 IntDen per cell within E-Cadherin-positive regions in (**1j**), each dot corresponds to the field-averaged value for a single individual, normalized to the mean of the control group.

I am not convinced (in the absence of studying this process using differentiated BECs from patients with asthma) allergen induced tissue perturbations – unclear what this means. The primary driver of airway pathology is allergen induced inflammation caused by T2 immunity. Direct epithelial damage by allergen is a less important secondary response and relies on inherent abnormality of asthmatic airway epithelial cells (otherwise everyone would be susceptible to allergen induced tissue perturbation). Only some allergens (with protease activity for example) have been documented to directly damage epithelium – and this is mostly in mouse models with high level of allergen exposure. OVA as allergen is used to study T2 immunity in mouse models of allergic airways disease. OVA does not possess protease activity and does not damage epithelium. Therefore the use of OVA to understand how allergen induced epithelial damage drives airway inflammation is not appropriate.

The authors state “Importantly, in both CA and OVA mouse models, we consistently observed that Tdp-43 inhibition alleviated asthmatic airway response”. I think this data undermines the main conclusion. OVA does not exhibit protease activity, and it is fundamentally different from protease containing allergens such as house dust mite (HDM; e.g., Der p 1) or cockroach allergens.

Fundamental to supporting the conclusions of this research is the use of ALI-differentiated BECs from patients with asthma. Using mouse ALI-BECs treated with IL-13 does not recapitulate the inherent abnormality of asthmatic BECs necessary to recapitulate the allergen-induced tissue perturbations and signaling pathway central the hypothesis:

Allergen exposure triggered tissue perturbations cellular stress response in AECs dysregulated TDP-43 expression compromised NMD activity asthmatic airway inflammation, this study aims to investigate the potential role of TDP 43 dysregulation in allergen induced airway asthmatic inflammatory response.

Response: We appreciate the reviewer’s thoughtful comment regarding this concern.

We agree with the reviewer on the opinion that ‘The primary driver of airway pathology is allergen induced inflammation caused by T2 immunity’ That is why at the

beginning of asthmatic mouse modeling, mice are sensitized by intraperitoneal injection of allergens emulsified with adjuvant (aluminum hydroxide). This step activates dendritic cells (DCs) to prime pro-Th2 response and promote B cell IgE class-switching. Post-sensitization, mice have IgE pre-coated on granulocytes without overt allergic symptoms. During sensitization, airway abnormalities may already begin to develop, as circulating type 2 cytokines produced by pro-Th2 can act on AECs to initiate pathological changes[1, 2]. Although OVA lacks protease activity and does not directly damage the epithelium, it can predispose the epithelium to barrier disruption by establishing type 2 immunity[1, 3], thereby facilitating allergen penetration during the challenge phase. This functionally resembles the intrinsic barrier defects observed in populations susceptible to allergen-induced asthma[3, 4, 5].

In a broad sense, airway tissue perturbations arise from both the direct actions of allergens that possess protease activity (e.g., HDM or CA, which combine both antigenic and immunogenic properties within the same entity[6, 7]) on the mucosal barrier, and the indirect consequences of Th2 immunity primed by a wider range of allergens including OVA. Because OVA act through this indirect pathway, the OVA-induced asthma model remains employed for studying airway barrier dysfunction[8, 9, 10]. Therefore, using the OVA-induced asthmatic mouse model to study how allergen-triggered airway damage drives airway inflammation does not necessarily invalidate this broader concept of tissue perturbation. Nevertheless, we also conducted the phenotypic and therapeutic experiments in CA-induced asthmatic mouse model.

In this context, although being recognized as a secondary response, allergen-induced

airway barrier abnormalities release epithelial alarmins (IL-25, IL-33, TSLP), which are essential for fully activating type 2 effector cells (ILC2s, Th2, etc.) and triggering asthmatic responses. The resulting type 2 cytokines further impair the airway barrier, establishing a vicious cycle[11, 12]. This positive feedback loop persists throughout the challenge phase, mirroring repeated allergen exposure that precipitates disease onset in asthma-prone individuals. **Thus, it seems that allergen-induced airway tissue disturbance is not confined to the early phase but continues across the entire duration, and TDP-43 might serve as an important contributor that helps promote this process**, as supported by the findings that AECs purified from both OVA- and CA-induced asthmatic mice exhibited induced Tdp-43 expression (Response Figure 2: 1i) and targeting Tdp-43 in AECs substantially attenuated airway inflammation and improved airway integrity in both OVA- and CA-challenged mice. We have accordingly modified the expressions in the introduction and discussion part in the revised manuscript.

As advised, we performed validations in ALI-differentiated AECs from patients with asthma. Owing to the significant challenges in recruiting patients for invasive bronchoscopic brushing, we acquired mucosal samples from a limited number of subjects: 2 with asthma, 1 with COPD (whose airway epithelial cells also exhibit intrinsic abnormalities), and 2 healthy controls for ALI culturing. After differentiation, the ALIs were treated with OVA or CA. Successful differentiation of all the 5 donor-derived ALIs was confirmed by H&E staining and acetylated α-tubulin (ACTUB) immunofluorescence (Response Figure 2: S3a). qPCR results demonstrated that the

expression levels of *IL33* and *TSLP* were markedly higher in ALI-cultured AECs from asthmatic patients compared with control subjects (Response Figure 2: S3b), indicating inherent abnormalities of AECs from asthmatic individuals.

TDP-43 expression was higher in the primary ALIs derived from patients with asthma and COPD than from controls (Response Figure 2: S3c). CA treatment further promoted TDP-43 expression in ALIs from the asthma/COPD group but not from controls (Response Figure 2: S3d and e), supporting that inherent AEC abnormality is a prerequisite for AEC responsiveness to allergen exposure. Direct OVA treatment on ALIs did not trigger TDP-43 upregulation (Response Figure 2: S3d). In OVA-induced asthmatic mice, however, Tdp-43 overexpression is driven by type 2 immunity-related airway epithelial damage, consistent with the finding that Il-13 stimulation induces Tdp-43 expression in the mouse ALIs (Response Figure 2: S2g and h).

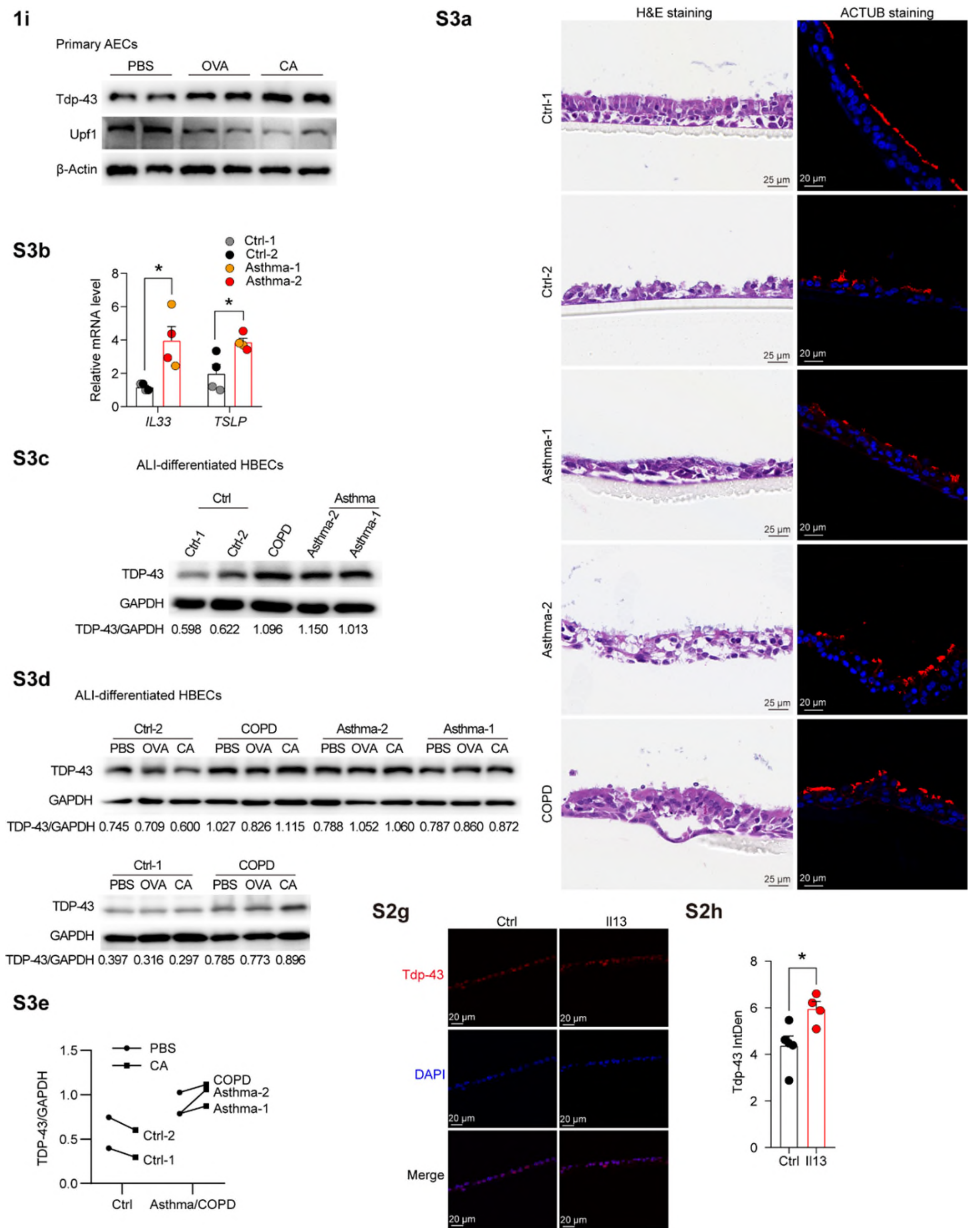

Response Figure 2 | 1i, Immunoblotting of Tdp-43 and Upf1 in the AECs isolated from CA/OVA-induced asthmatic mice and control mice. **S3a,** Representative images of H&E staining and ACTUB IF staining in Asthma, COPD and control patient HBECs under ALI conditions. **S3b,** mRNA levels of *IL33* and *TSLP* in Asthma and control patient HBECs under ALI conditions, each dot denotes one independent ALI biological

replicate. **S3c,** Immunoblotting of TDP-43 in Asthma, COPD and control patient HBECs under ALI conditions. **S3d,** Immunoblotting of TDP-43 in Asthma and control patient AECs under ALI conditions and treated with CA (200 μg/ml), OVA (400 μg/ml) or PBS for 24 h. **S3e,** Quantitative analysis of TDP-43 expression normalized to GAPDH in (**S3d**). **S2g,** Representative IF images of Tdp-43 in Il13-stimulated and control AECs under ALI conditions. **S2h,** Quantitative IF results of Tdp-43 in (**S2g**).

Figure 6c – I see no evidence of infection and replication of IAV – there is no increase (above input) of NP – this data does not add anything to the main conclusions of the study. Similarly, the assessment of influenza infection and replication time-course in vivo is qualitative (IF). The response states significantly low NP in KO mice. A quantitative measure of viral load (qPCR, infectivity) would be more convincing.

Response: Thanks for the constructive advice. To validate the success of PR8 infection, uninfected cells were included as a negative control in our previous assay, with NP CT values in these cells being greater than 34 or undetectable. Following your suggestion, we have replotted *NP* expression, including uninfected cells as a control. A marked increase in NP expression relative to input can be clearly observed. We have moved the revised Figure 6c to Figure S8a.

To quantitatively assess the enhanced resistance of *Tardbp*$^{\Delta Scgb1a1}$ mice to PR8 infection compared with *Tardbp*^{flox}, we used real-time PCR to measure *NP* expression in lung tissues from *Tardbp*^{flox} and *Tardbp*$^{\Delta Scgb1a1}$ mice at 3, 6, 10, and 15 days post-PR8 infection. NP expression was undetectable in the lungs of uninfected mice. At day 3 and day 6 post-infection, *NP* levels were significantly lower in the lungs of

Tardbp$^{\Delta Scgb1a1}$ mice than in *Tardbp*^{flox} mice. By day 10, pulmonary *NP* levels had decreased substantially in both groups, and no significant difference remained between them. By day 15, CT values of *NP* exceeded 30, indicating that PR8 virus had been completely cleared from the lungs (Response Figure 3: 6h). Western blot analysis of lung tissues from mice on day 6 post-PR8 infection also demonstrated that NP protein expression was significantly lower in *Tardbp*$^{\Delta Scgb1a1}$ mice than in *Tardbp*^{flox} mice (Response Figure 3: S8d and e).

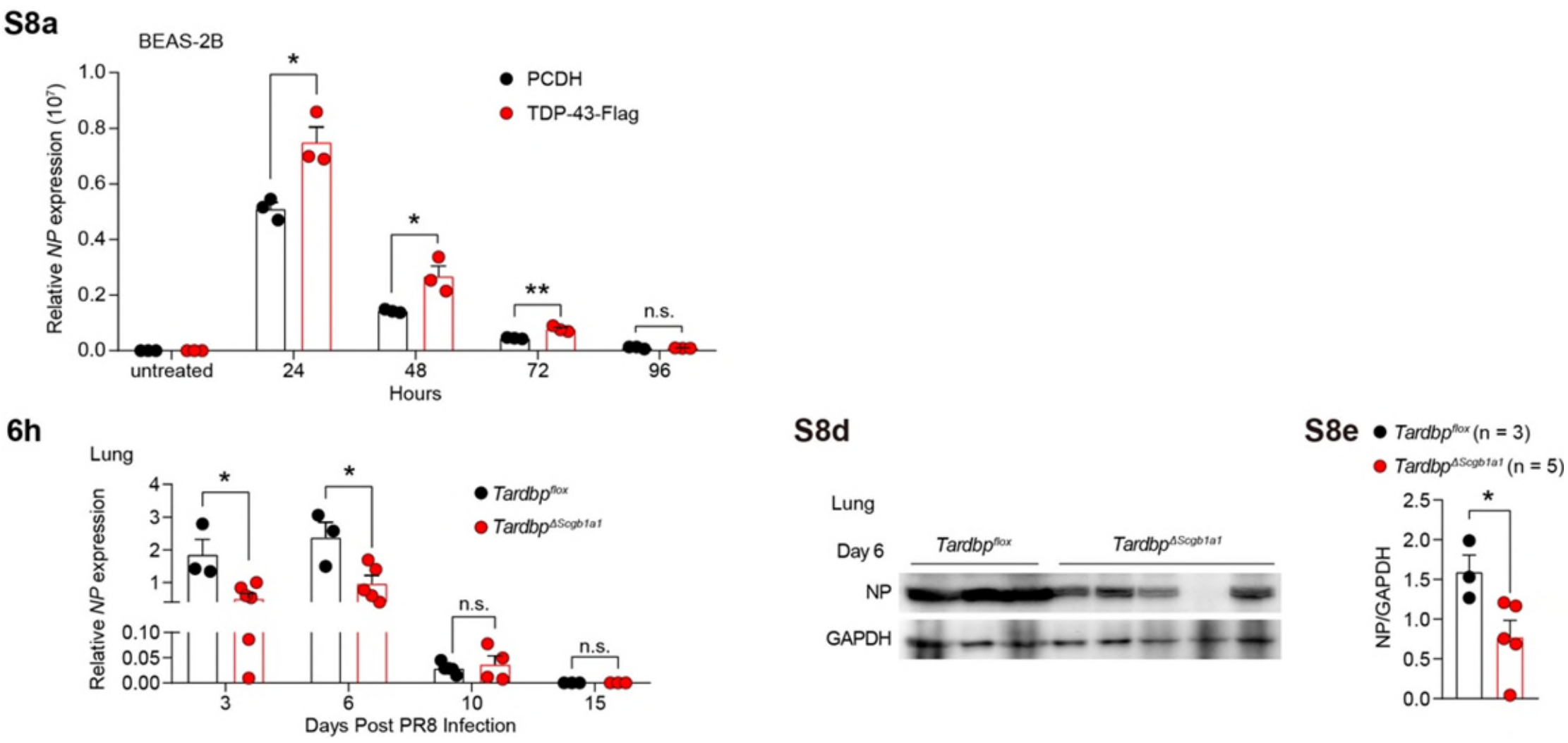

Response Figure 3 | **S8a**, qPCR results showing *NP* expression in TDP-43-overexpressed BEAS-2B cells infected with PR8 at different time points, untreated indicates no PR8 infection. **6h**, qPCR results showing *NP* expression in lungs from *Tardbp*$^{\Delta Scgb1a1}$ and *Tardbp*^{flox} mice at 3, 6, 10, and 15 days post-infection with PR8 virus. **S8d**, Immunoblotting of NP in lungs from *Tardbp*$^{\Delta Scgb1a1}$ and *Tardbp*^{flox} mice infected with the PR8 virus for 6 days. **S8e**, Quantitative analysis of NP expression in (**S8d**), each dot represents an individual mouse.

Reviewer #3 (Remarks to the Author):

The authors have adequately addressed all of my concerns. Specifically, the addition of scRNA-seq and mechanistic data (RIP-qPCR/minigene assay) has significantly strengthened the manuscript. I have no further comments and recommend the paper for publication.

Response: We greatly appreciate the reviewer's professional comments and are delighted that our revisions were accepted.

1. Moonwiriyakit A, *et al.* GPR120/FFAR4 stimulation attenuates airway remodeling and suppresses IL-4- and IL-13-induced airway epithelial injury via inhibition of STAT6 and Akt. *Biomed Pharmacother* **168**, 115774 (2023).

2. Raby KL, Michaeloudes C, Tonkin J, Chung KF, Bhavsar PK. Mechanisms of airway epithelial injury and abnormal repair in asthma and COPD. *Front Immunol* **14**, 1201658 (2023).

3. Holgate ST, Roberts G, Arshad HS, Howarth PH, Davies DE. The role of the airway epithelium and its interaction with environmental factors in asthma pathogenesis. *Proc Am Thorac Soc* **6**, 655-659 (2009).

4. Hackett TL, *et al.* Intrinsic phenotypic differences of asthmatic epithelium and its inflammatory responses to respiratory syncytial virus and air pollution. *Am J Respir Cell Mol Biol* **45**, 1090-1100 (2011).

5. Portelli MA, *et al.* Elevated PLAUR is observed in the airway epithelium of asthma patients and blocking improves barrier integrity. *Clin Transl Allergy* **13**, e12293 (2023).

6. Agaronyan K, *et al.* Tissue remodeling by an opportunistic pathogen triggers allergic inflammation. *Immunity* **55**, 895-911 e810 (2022).

7. Sokol CL, Barton GM, Farr AG, Medzhitov R. A mechanism for the initiation of allergen-induced T helper type 2 responses. *Nat Immunol* **9**, 310-318 (2008).

8. Jin J, *et al.* Fallopia japonica Root Extract Ameliorates Ovalbumin-Induced Airway Inflammation in a CARAS Mouse Model by Modulating the IL-33/TSLP/NF-kappaB Signaling Pathway. *Int J Mol Sci* **24**, (2023).

9. Xu Y, Feng Y, Wang L, Xu X, Xu L, Wang B. Benzo[a]pyrene aggravated ovalbumin-induced epithelial tight junction disruption via ROS driven-NLRP3/Caspase-1 signaling pathway in asthmatic mice. *Int J Mol Med* **56**, (2025).

10. Luo X, *et al.* Nebulization of Hypoxic hUCMSC-EVs Attenuates Airway Epithelial Barrier Defects in Chronic Asthma Mice by Transferring CAV-1. *Int J Nanomedicine* **19**, 10941-10959 (2024).

11. Roan F, Obata-Ninomiya K, Ziegler SF. Epithelial cell-derived cytokines: more than just signaling the alarm. *J Clin Invest* **129**, 1441-1451 (2019).

12. Kopp EB, Agaronyan K, Licona-Limon I, Nish SA, Medzhitov R. Modes of type 2 immune response initiation. *Immunity* **56**, 687-694 (2023).

REVIEWERS' COMMENTS

Reviewer #2 (Remarks to the Author):

I thank the authors for the substantial additional data. All outstanding concerns have been addressed.

Response: We sincerely appreciate your positive feedback and the acknowledgment that our revisions have adequately addressed all concerns. We are delighted to reach this stage. Thank you again for your time and guidance.
